# Sociodemographic Variation in Gratitude Using a Cross-National Analysis with 22 Countries

**DOI:** 10.1007/s41042-025-00254-w

**Published:** 2025-10-13

**Authors:** Sakurako S Okuzono, Richard G Cowden, Noah Padgett, George Yancey, Byron R Johnson, Tyler J VanderWeele

**Affiliations:** 1 Department of Social and Behavioral Sciences, Harvard T.H. Chan School of Public Health, Boston, United States; 2 Human Flourishing Program, Institute for Quantitative Social Science, Harvard University, Cambridge, United States; 3 Department of Epidemiology, Harvard T.H. Chan School of Public Health, Boston, United States; 4 Institute for Studies of Religion, Baylor University, Waco, United States; 5 Institute for Global Human Flourishing, Baylor University, Waco, United States

**Keywords:** Gratitude, Disparities, Multinational, Population well-being, Global flourishing study

## Abstract

We used nationally representative data from the first wave of the Global Flourishing Study (*N* = 202,898) to (1) explore the distribution of gratitude in 22 geographically and culturally diverse countries and (2) identify potential differences in mean gratitude across nine sociodemographic characteristics, including age, gender, marital status, employment status, years of education, immigrant status, frequency of religious service attendance, religious affiliation, and racial/ethnic identity. Our descriptive analysis supported substantial variation in gratitude across countries. The highest mean gratitude was in Indonesia (M = 8.93, SD = 1.76), whereas the lowest was in Japan (M = 5.81, SD = 2.25). We estimated country-level descriptive statistics for gratitude in each sociodemographic category and then performed a series of random-effects meta-analyses to aggregate results for the seven sociodemographic variables that were assessed consistently across countries. When pooled across countries, the categories for each sociodemographic characteristic with the highest mean gratitude included those who are older, women, married, immigrants, retired or self-employed, have completed 16 + years of education, and attend religious services more than once a week. Sociodemographic differences in gratitude varied to some extent by country. Our findings enhance existing knowledge about the distribution of gratitude and sociodemographic disparities in gratitude, and provide preliminary evidence about which groups might be strategic targets for interventions to promote gratitude.

**Supplementary Information:**

The online version contains supplementary material available at 10.1007/s41042-025-00254-w.

## Introduction

Gratitude is the expression of appreciation for what one has in life (Wood et al., 2010). An abundance of research has shown that gratitude is a psychological asset that has the potential to improve population health and well-being, with a range of psychological interventions available to cultivate individual experiences of gratitude (Wood et al., 2010). While it may be important for people to experience moments of gratitude, health and well-being are likely to be most positively affected if people are able to experience gratitude with greater consistency and intensity (Cowden et al., 2021). The generalization of gratitude across time and situations in daily life is often referred to as dispositional gratitude (Cowden et al., 2021; Mccullough et al., 2002).

A significant amount of research has provided evidence of connections between higher gratitude and various positive psychosocial outcomes, including better mental well-being (e.g., life satisfaction, positive affect) (Lin & Yeh, 2014; Wood et al., 2010), lower mental distress (e.g., lower depressive symptoms, less suicidal ideation) (McGuire et al., 2021), better health behaviors (e.g., less substance use, less alcohol use), more frequent religious participation (Portocarrero et al., 2020), and increased social support (Wood et al., 2008). In addition, higher gratitude is associated with various physical health outcomes, such as lower mortality(Chen et al., 2024), higher self-rated health (Hill et al., 2013), lower obesity (Chen et al., 2016), less sleep disturbance (O’Connell et al., 2016), and better cardiovascular health (Huffman et al., 2016; Moieni et al., 2019). In addition, interventions to improve gratitude have shown promise (Boggiss et al., 2020; Folk & Dunn, 2023; Jans-Beken et al., 2020). For instance, two recent intervention studies found that writing gratitude messages was associated with an increase in positive affect over the short term (Davis et al., 2016; Folk & Dunn, 2023). Cultivating gratitude as a public health strategy could have important population-level benefits to health and well-being.

Numerous research studies have used multinational data to describe gratitude across cultures (Heintz et al., 2019; McGrath, 2015; Park et al., 2006; Srirangarajan et al., 2020). For instance, one study with more than 50 nations identified gratitude as one of the top five character strengths, with the ranking of gratitude ranging from 3 (e.g., United Arab Emirates, mean: 4.03) to 17 (e.g., Uzbekistan, mean: 3.51) out of the 24 character strengths assessed (Park et al., 2006). In another study involving 75 nations, Estonia had the lowest gratitude, with a mean gratitude score of 3.48 (gratitude ranked 20th out of the 24 character strengths in Estonia), whereas the highest mean gratitude was in the United States (mean = 3.99, gratitude ranked 6th out of the 24 character strengths in the United States) (McGrath, 2015). These cross-country variations in gratitude levels are likely attributable to a range of sociocultural factors. For instance, in cultures where social harmony is highly valued, expressing gratitude is often deeply ingrained as a social norm. Cultural values also play a role in shaping whether gratitude is primarily associated with personal gain (individualism) or group harmony (collectivism). Research suggests similarities in the distribution of gratitude between the United States and the United Kingdom, both considered individualistic cultures (Morgan et al., 2014). The dominant religion within a country can also influence gratitude levels, as some religions explicitly teach and promote gratitude as a virtue (Portocarrero et al., 2020; Rosmarin et al., 2011). In addition, economic conditions may contribute to between-country differences in gratitude, though further research is needed to explore this relationship.

Beyond broad cultural differences, research suggests that demographic factors might be related to gratitude, and that these relationships can vary across cultures. Studies have found differences in the distribution of gratitude based on gender (Heintz et al., 2019; Sommers & Kosmitzki, 1988) and age (Heintz et al., 2019). Others have found variations related to educational status or personality characteristics (e.g., extraverted vs. introverted) between countries like Japan and the United States (Robustelli & Whisman, 2018) or variations related to age and sex in Germany and the United States. Interestingly, some studies have even found differences in associations between gratitude and other emotions in samples that share a relatively similar cultural orientation. For example, research comparing Japan and Thailand found differences in how gratitude expression correlates with emotions like indebtedness or prosocial motivation (Naito et al., 2005). Even in the case of the United States and the United Kingdom, which share some cultural commonalities, evidence of differences in gratitude illustrates how the socially constructed elements of gratitude can differ even within seemingly similar contexts (Morgan et al., 2014). These findings suggest that understanding demographic variations in gratitude requires considering the specific cultural contexts in which these factors operate.

Socioecological systems theory provides one useful framework for grasping these complex interactions. This theory posits that individual development, including the development of traits like gratitude, is shaped by multiple, interconnected socioecological systems (Bronfenbrenner & Others, 1994). These range from individual-level factors (e.g., personality, demographics) to interpersonal relationships, macro-level influences (e.g., cultural norms, societal values, religious institutions), and even the historical context. For instance, marital status may affect gratitude through supporting partner relationships (e.g., microsystem), or immigration status may shape gratitude via community support, cultural integration, or exposure to discrimination (e.g., meso- or macrosystem) (Mccullough et al., 2002; Wood et al., 2010). While personal experiences and relationships remain influential, the broader sociocultural environment can impinge on a person’s experience of gratitude (Heintz et al., 2019). Societies that weave gratitude into their fabric—through social norms, cultural narratives, and institutions—are likely to cultivate a more grateful populace overall.

Despite the important contributions of prior research, existing cross-cultural studies have at least four limitations. First, most relevant cross-cultural studies have relied on convenience samples, leaving open questions about the generalizability of findings. Second, some studies examining the distribution of gratitude have aggregated data from multiple years, and the temporal variation within a country may have been masked (McGrath, 2015). This would prevent us from proposing public health policies to improve population gratitude, since it is difficult to determine what the magnitude of gratitude is at a particular point in time and how gratitude may have changed over time in that specific context. Third, while many studies have primarily focused on differences in gender or comparisons between individualistic and collectivist cultures (Heintz et al., 2019), further exploration of sociodemographic factors other than gender is needed. For instance, we might hypothesize that individuals with higher levels of religious participation would report higher levels of gratitude, given the potential role of religion in shaping such perspectives. Furthermore, education may contribute to improving gratitude; indeed, some research suggests a positive correlation between educational attainment and gratitude levels (Hartanto et al., 2019). Investigating how gratitude differs based on these sociodemographic factors across cultures could provide a more nuanced understanding of gratitude in different cultures and subpopulations, which could lead to more effective and culturally sensitive policy implications. Fourth, the country-specific samples included in prior cross-national studies are often too small to represent one nation’s average (e.g., less than 50 people in some countries), raising questions about the representativeness of the results(Park et al., 2006). Existing research or data may not be sufficient to monitor the population distribution of gratitude over time and identify disparities.

This study addresses some of these existing gaps in knowledge using data from the Global Flourishing Study (GFS). The GFS contains nationally representative data from more than 200,000 individuals across 22 geographically and culturally diverse countries. We aim to explore the distribution of gratitude levels in each country and test for potential sociodemographic differences in gratitude levels. Specifically, we examine the distribution of gratitude across key sociodemographic characteristics (i.e., age, gender, marital status, employment status, years of education, immigration status, frequency of religious service attendance, religious affiliation, race/ethnicity). Based on the existing evidence, we anticipated that being a woman (Heintz et al., 2019; Sommers & Kosmitzki, 1988), being married, and more frequent religious participation would be associated with higher levels of gratitude (Chen et al., 2024). Further, we expected to observe meaningful cross-national variation in the distribution of gratitude, as suggested by previous literature. Although we anticipated that there would be some sociodemographic differences in gratitude across all countries, on the basis of socioecological system theory (Bronfenbrenner & Others, 1994), we expected some cross-national variation in sociodemographic differences because of the diverse sociocultural contexts of the included countries.

## Methods

The methods described below have been adapted from VanderWeele et al.(VanderWeele, 2024). Further methodological details are available elsewhere (Johnson et al., 2023; Lomas et al., 2025; R. Noah Padgett, Bradshaw et al., 2025; Padgett et al., 2025; Ritter et al., 2024).

### Participants

The Global Flourishing Study (GFS) is a study of 202,898 participants from 22 geographically and culturally diverse countries, with nationally representative sampling within each country, concerning the distribution of determinants of well-being. Wave 1 of the data included the following countries and territories: Argentina, Australia, Brazil, Egypt, Germany, Hong Kong (Special Administrative Region of China), India, Indonesia, Israel, Japan, Kenya, Mexico, Nigeria, the Philippines, Poland, South Africa, Spain, Sweden, Tanzania, Türkiye, the United Kingdom, and the United States. The countries were selected to (a) maximize coverage of the world’s population, (b) ensure geographic, cultural, and religious diversity, and (c) prioritize feasibility and existing data collection infrastructure. Data collection was carried out by Gallup Inc. Data for Wave 1 were collected principally during 2023, with some countries beginning data collection in 2022 and exact dates varying by country (Johnson et al., 2023; Lomas et al., 2025; Ritter et al., 2024). Plans are in place to collect four additional annual waves of panel data on the participants from 2024 to 2027. The precise sampling design to ensure nationally representative samples varied by country, and further details are available elsewhere (Padgett et al., 2025; Ritter et al., 2024). Survey items included aspects of well-being such as happiness, health, meaning, character, relationships, and financial stability (VanderWeele, 2017), along with other demographic, social, economic, political, religious, personality, childhood, community, health, and well-being variables. The data are publicly available through the Center for Open Science (COS) (https://www.cos.io/gfs). During the translation process, Gallup adhered to the TRAPD model (translation, review, adjudication, pretesting, and documentation) for cross-cultural survey research (Padgett et al., 2025).

### Measures

#### Sociodemographic Variables

Seven sociodemographic variables were assessed consistently across the 22 countries based on theoretical frameworks, expert knowledge, and feasibility (Lomas et al., 2025): age, gender, marital status, employment status, education status, frequency of religious service attendance, and immigration status. Continuous age was classified as 18–24, 25–29, 30–39, 40–49, 50–59, 60–69, 70–79, and 80 or older. Gender was assessed as man, woman, or other. Marital status was assessed as single/never married, married, separated, divorced, widowed, or domestic partner. Employment was assessed as employed, self-employed, retired, student, homemaker, unemployed and searching, and other. Education was assessed as up to 8 years, 9–15 years, and 16 + years. Frequency of religious service attendance was assessed as more than once a week, once a week, one to three times/month, a few times/year, or never. Immigration status was dichotomously assessed with an item asking participants to report whether they had been born in the country where data collection was taking place.

Religious affiliation was also assessed in all countries, but there were considerable cross-country differences in the response categories that were endorsed by participants because some religious affiliations are only applicable in certain countries and not others, and precise response categories varied by country (Johnson et al., 2023). The categories of religious affiliation included Christianity, Islam, Hinduism, Buddhism, Judaism, Sikhism, Baha’i, Jainism, Shinto, Taoism, Confucianism, Primal/Animist/Folk religion, Spiritism, African-derived, some other religion, or no religion/atheist/agnostic. Racial/ethnic identity was assessed in most (18/22) countries, and response categories varied across countries. For additional details about these variables, see the GFS codebook (https://osf.io/cg76b) or Crabtree et al. (Crabtree et al., 2021/2025).

#### Outcome Variable

Overall dispositional gratitude was assessed with a single item adapted from the well-validated Gratitude Questionnaire-Six Item Form (GQ-6) (Mccullough et al., 2002): “If I had to list everything that I felt grateful for, it would be a very long list.” The decision to select this item was informed by prior validation work, input from subject experts, and considerations of cross-cultural applicability. Cognitive interviews and pilot testing were used to ensure clarity and cultural relevance across the countries included in the GFS (Crabtree et al., 2021/2025; Lomas et al., 2025). Response options were anchored at 0 (*Strongly disagree*) and 10 (*Strongly agree*). We used this indicator as a continuous variable in the analysis.

### Analysis

Descriptive statistics for the full sample, weighted to be nationally representative within each country, were estimated for each of the demographic variables. Nationally representative means for gratitude were estimated separately for each country (and ordered from highest to lowest), with 95% confidence intervals, and standard deviations. We further reported the Gini coefficients for each country to descriptively provide the levels of inequality in the gratitude scores across participants in each country.

Variations in means of gratitude across demographic categories were estimated, with all analyses initially conducted by country (online Supplemental File). Primary results consisted of random-effects meta-analyses of country-specific means of gratitude in each specific demographic category (Borenstein et al., 2010; Hunter & Schmidt, 2000) along with 95% confidence intervals, standard errors, and lower and upper limits of a 95% prediction interval across countries. We used random-effects meta-analysis over multi-level modeling since random-effects meta-analysis does not presume cross-cultural measurement equivalence of the measures, which is important because (1) most constructs were assessed using a single item and (2) cognitive testing during the survey development process suggested some variation in the interpretation of items across countries (Mathur & VanderWeele, 2020). In addition to obtaining the overall mean gratitude within each demographic category across countries, we calculated a “tau” heterogeneity estimate (*τ*) representing the estimated standard deviation of the means across countries, and *I*^2^ representing the proportion of variability due to heterogeneity across countries versus sampling variability. These statistics were used to assess variation across countries within each demographic category (Mathur & VanderWeele, 2020). A description of these methods and a more detailed discussion of the rationale underpinning the choice of a meta-analytic approach (over multilevel modeling) can be found in Mathur et al. (Mathur & VanderWeele, 2020). Forest plots of estimates are available in the online Supplemental File. All meta-analyses were conducted in R (*R: A Language and Environment for Statistical Computing. R Foundation for Statistical Computing*, n.d.) using the metafor package (Viechtbauer, 2010). Within each country, a global test of variation of outcome across levels of each particular demographic variable was conducted, and a pooled *p*-value across countries reported evidence of variation within at least one country (i.e., global *p*-value) (Wilson, 2019). We provide Bonferroni corrected *p*-value thresholds based on the seven sociodemographic variables (i.e., age, gender, marital status, employment status, education status, frequency of religious service attendance, and immigration status) that were included in the meta-analyses: *p* =.05/7 = 0.007 (Abdi, 2007; Mathur & VanderWeele, 2020). Religious affiliation and race/ethnicity were not included in the meta-analyses since these variables were assessed inconsistently across the countries. As a supplementary analysis, population-weighted meta-analyses were also conducted. All analyses were pre-registered prior to data access (https://osf.io/mnx4s); all code to reproduce analyses is openly available in an online repository (Padgett et al., 2024).

#### Missing Data

Missing data on all variables was imputed using multivariate imputation by chained equations, and five imputed datasets were used (Acock, 2005; Rubin, 1996; Sterne et al., 2009; van Buuren, 2021). To account for variation in the assessment of certain variables across countries (e.g., religious affiliation and race/ethnicity), the imputation process was conducted separately in each country. This within-country imputation approach ensured that the imputation models accurately reflected country-specific contexts and assessment methods. Sampling weights were included in the imputation models to account for specific variable missingness that may have been related to the probability of inclusion in the study.

#### Accounting for Complex Sampling Design

The GFS used different sampling schemes across countries based on the availability of existing panels and recruitment needs (Padgett et al., 2025). All analyses accounted for the complex survey design components by including weights, primary sampling units, and strata. Additional methodological detail, including accounting for the complex sampling design, is provided elsewhere (R. Noah Padgett, Bradshaw et al., 2025; Padgett et al., 2025).

## Results

Table 1 shows the number and percentage of people in each demographic group in the observed sample. Most individuals were middle-aged (30–39 years: 20%; 40–49 years: 17%; 50–59 years: 16%), about half were women (51%) and married (52%), about one-third were employed for an employer (39%), and most were born in the country in which the survey was conducted (94%). The countries with the greatest number of participants were the United States (19%) and Japan (10%), whereas the countries with the lowest number of participants were Türkiye (0.7%) and South Africa (1.3%). Tables S1a to S22a in the Supplemental File show the number and percentage of people in each demographic group in each of the 22 countries.

**Table 1:** Nationally representative descriptive statistics of the observed sample (*n* = 202,898)

Characteristic	*N* = 202,8981
Age group	
18–24	27,007 (13%)
25–29	20,700 (10%)
30–39	40,256 (20%)
40–49	34,464 (17%)
50–59	31,793 (16%)
60–69	27,763 (14%)
70–79	16,776 (8.3%)
80 or older	4,119 (2.0%)
Missing	20 (< 0.1%)
Gender	
Man	98,411 (49%)
Woman	103,488 (51%)
Other	602 (0.3%)
Missing	397 (0.2%)
Marital status	
Married	107,354 (53%)
Separated	5,195 (2.6%)
Divorced	11,654 (5.7%)
Widowed	9,823 (4.8%)
Never	52,115 (26%)
Domestic Partner	14,931 (7.4%)
Missing	1,826 (0.9%)
Employment	
Employed for an employer	78,815 (39%)
Self-employed	36,362 (18%)
Retired	29,303 (14%)
Student	10,726 (5.3%)
Homemaker	21,677 (11%)
Unemployed and looking for a job	16,790 (8.3%)
None of these/other	8,431 (4.2%)
Missing	793 (0.4%)
Religious service attendance	
> 1/week	26,537 (13%)
1/week	39,157 (19%)
1–3/month	19,749 (9.7%)
A few times a year	41,436 (20%)
Never	75,297 (37%)
Missing	722 (0.4%)
Education	
up to 8 years	45,078 (22%)
9–15 years	115,097 (57%)
16 + years	42,578 (21%)
Missing	146 (< 0.1%)
Immigration	
Born in this country	190,998 (94%)
Born in another country	9,791 (4.8%)
Missing	2,110 (1.0%)
COUNTRY	
Argentina	6,724 (3.3%)
Australia	3,844 (1.9%)
Brazil	13,204 (6.5%)
Egypt	4,729 (2.3%)
Germany	9,506 (4.7%)
India	12,765 (6.3%)
Indonesia	6,992 (3.4%)
Israel	3,669 (1.8%)
Japan	20,543 (10%)
Kenya	11,389 (5.6%)
Mexico	5,776 (2.8%)
Nigeria	6,827 (3.4%)
Philippines	5,292 (2.6%)
Poland	10,389 (5.1%)
South Africa	2,651 (1.3%)
Spain	6,290 (3.1%)
Tanzania	9,075 (4.5%)
Türkiye	1,473 (0.7%)
United Kingdom	5,368 (2.6%)
United States	38,312 (19%)
Sweden	15,068 (7.4%)
Hong Kong (S.A.R. of China)	3,012 (1.5%)

*n* = sampling weight adjusted count (i.e., effective sample size), % = sampling weight adjusted proportion, *S.A.R. *Special Administrative Region

Table 2 shows the ordered mean of gratitude with standard deviation and Gini coefficients to assess the dispersion or inequality in gratitude across participants in each country. The countries with the greatest mean gratitude were Indonesia (8.93, SD = 1.76), Mexico (8.84, SD = 1.75), and the Philippines (8.64, SD = 2.0), whereas the countries with the lowest mean gratitude were Japan (5.81, SD = 2.25), Türkiye (6.96, SD = 2.73), and Hong Kong (6.97, SD = 2.22). The Gini coefficient for gratitude was highest in Türkiye (0.22) and Japan (0.21) and lowest in Indonesia and Mexico (both 0.09).

**Table 2:** Ordered means of gratitude with standard deviations and Gini coefficients

Country	Mean	95% CI	Standard Deviation	Gini Coefficient
Indonesia	8.93	(8.86, 9.00)	1.76	0.09
Mexico	8.84	(8.78, 8.90)	1.75	0.09
Philippines	8.64	(8.57, 8.71)	2.00	0.11
Brazil	8.61	(8.57, 8.66)	2.05	0.11
Argentina	8.53	(8.47, 8.60)	1.99	0.11
Nigeria	8.41	(8.32, 8.50)	2.11	0.12
United States	8.14	(8.08, 8.20)	2.2	0.14
Kenya	8.08	(7.99, 8.16)	2.73	0.17
Israel	7.91	(7.71, 8.10)	2.03	0.14
South Africa	7.85	(7.71, 7.98)	2.42	0.16
Tanzania	7.85	(7.71, 7.99)	2.88	0.19
Australia	7.83	(7.73, 7.93)	2.23	0.15
Spain	7.79	(7.72, 7.86)	2.09	0.14
Egypt	7.75	(7.64, 7.85)	2.52	0.17
India	7.71	(7.62, 7.79)	3.13	0.2
Germany	7.63	(7.58, 7.69)	2.17	0.15
Sweden	7.48	(7.44, 7.53)	2.24	0.16
United Kingdom	7.41	(7.32, 7.50)	2.36	0.17
Poland	7.35	(7.25, 7.46)	1.82	0.13
Hong Kong (S.A.R. of China)	6.97	(6.86, 7.07)	2.22	0.18
Türkiye	6.96	(6.79, 7.14)	2.73	0.22
Japan	5.81	(5.78, 5.85)	2.24	0.21

*S.A.R. *Special Administrative Region, *CI* confidence interval

Table 3 shows the random-effects meta-analyses of mean gratitude by demographic group across the 22 countries. Across countries, mean gratitude was highest in the oldest age group (80 or older: 8.22, 95%CI: 7.93, 8.51), among women (7.96, 95%CI: 7.67, 8.25), those who are married (8.02, 95%CI: 7.73, 8.31), those who are retired (7.95, 95%CI: 7.65, 8.25) or self-employed (7.95, 95%CI: 7.65, 8.25), those with 16 years or more of education (7.98, 95%CI: 7.68, 8.29), those who attend religious services more than once a week (8.54, 95%CI: 8.28, 8.80), and those born in another country (7.91, 95%CI: 7.58, 8.23). When comparing the highest and lowest mean gratitude within each demographic category, the largest group differences in gratitude were found for frequency of religious service attendance (mean difference: 1.08). Specifically, those who attend religious services more than once a week (8.54, 95%CI: 8.28, 8.80) reported the highest mean gratitude, while those who never attend religious services had the lowest mean gratitude (7.46, 95%CI: 7.16, 7.76). The smallest difference was observed for immigration status (mean difference: 0.07), with a slightly lower mean gratitude reported among those who were born in the country where data collection occurred (7.84, 95%CI: 7.54, 8.14). Global *p*-values for each sociodemographic characteristic were *p* <.001 after Bonferroni correction, indicating that there was evidence of differences in mean gratitude for each sociodemographic variable in at least one country.

**Table 3:** Random-effects meta-analysis of gratitude means by demographic category

					Prediction Interval				
Variable	Category	Est	95% CI	SE	LL	UL	Heterogeneity (τ)	I^2	Global p-value
Age group									< 0.001**
	18–24	7.69	(7.38,7.99)	0.15	5.87	8.95	0.71	98.4	
	25–29	7.77	(7.45,8.10)	0.17	5.51	9.00	0.77	98.3	
	30–39	7.76	(7.43,8.10)	0.17	5.46	9.05	0.80	99.2	
	40–49	7.80	(7.48,8.13)	0.17	5.46	8.96	0.78	99.0	
	50–59	7.89	(7.54,8.23)	0.17	5.56	9.15	0.81	99.1	
	60–69	7.90	(7.60,8.21)	0.16	5.96	8.94	0.71	98.7	
	70–79	7.94	(7.66,8.21)	0.14	6.44	9.04	0.61	97.3	
	80 or older	8.22	(7.93,8.51)	0.15	6.84	9.02	0.59	89.7	
Gender									< 0.001**
	Man	7.72	(7.41,8.03)	0.16	5.55	8.92	0.73	99.5	
	Woman	7.96	(7.67,8.25)	0.15	6.05	8.97	0.70	99.6	
	Other	7.00	(6.39,7.61)	0.31	5.26	8.88	1.05	80.0	
Marital status									< 0.001**
	Married	8.02	(7.73,8.31)	0.15	6.02	9.05	0.69	99.6	
	Separated	7.60	(7.26,7.94)	0.17	5.91	8.78	0.76	93.8	
	Divorced	7.71	(7.34,8.08)	0.19	5.68	9.16	0.83	97.3	
	Widowed	8.00	(7.68,8.31)	0.16	6.29	8.97	0.72	96.0	
	Domestic partner	7.83	(7.43,8.24)	0.21	5.65	9.67	0.90	98.8	
	Single, never married	7.54	(7.16,7.91)	0.19	5.19	8.96	0.89	99.4	
Employment status									< 0.001**
	Employed for an employer	7.85	(7.55,8.16)	0.16	5.65	8.93	0.73	99.5	
	Self-employed	7.95	(7.65,8.25)	0.15	6.06	8.99	0.71	98.8	
	Retired	7.95	(7.65,8.25)	0.15	6.11	8.97	0.69	98.6	
	Student	7.72	(7.41,8.04)	0.16	6.29	9.15	0.74	97.1	
	Homemaker	7.84	(7.51,8.17)	0.17	6.16	9.02	0.77	98.3	
	Unemployed and looking for a job	7.35	(6.90,7.79)	0.23	4.65	8.87	1.05	98.6	
	None of these/other	7.50	(7.09,7.92)	0.21	5.55	8.92	0.94	96.8	
Education									< 0.001**
	Up to 8 years	7.79	(7.44,8.13)	0.18	5.14	8.89	0.81	98.6	
	9–15 years	7.81	(7.50,8.13)	0.16	5.67	8.98	0.75	99.7	
	16 + years	7.98	(7.68,8.29)	0.15	6.29	9.01	0.71	99.3	
Religious service attendance									< 0.001**
	> 1/week	8.54	(8.28,8.80)	0.13	7.39	9.47	0.60	98.0	
	1/week	8.18	(7.94,8.42)	0.12	7.06	8.99	0.57	98.2	
	1–3/month	7.93	(7.66,8.21)	0.14	6.14	8.87	0.65	97.5	
	A few times a year	7.84	(7.54,8.14)	0.15	6.12	8.85	0.71	99.1	
	Never	7.46	(7.16,7.76)	0.15	5.67	8.76	0.71	99.3	
Immigration status									< 0.001**
	Born in this country	7.84	(7.54,8.14)	0.15	5.81	8.93	0.72	99.7	
	Born in another country	7.91	(7.58,8.23)	0.17	6.11	9.54	0.73	95.4	

Note. CI = confidence interval, *SE* = standard error, τ = tau, *I*^2^ = I-squared statistic

Est: Estimated mean of gratitude in the sociodemographic category

*SE* Standard error analogue

Prediction interval: Variation in country-specific gratitude in the sociodemographic category

Lower: Lower limit of the approximately 95% prediction interval

Upper: Upper limit of the approximately 95% prediction interval

τ:The standard deviation of the distribution of mean gratitude across countries, an indicator of cross-national heterogeneity. Additional information on heterogeneity is available in the forest plots for each estimate (see Figures S1-S34)

*I*^2^:An estimate of the variability in mean for gratitude due to heterogeneity across countries versus sampling variability. Given that the sample sizes for each country are large, the *I*^2^ is high

Global *p*-value: A test of the null hypothesis that there are no differences in mean for gratitude between the categories for the sociodemographic characteristic in any of the 22 countries

All *p*-values passed the Bonferroni-corrected threshold of* p* =.007 (*p* =.05/7)

The “tau” estimate shows cross-country variation in gratitude for each of the sociodemographic categories. There was some evidence of cross-national heterogeneity in gratitude for all sociodemographic categories, although variation was greater for some categories than others. The categories that had the highest tau estimates for each demographic variable was the age category “50–59” (0.81), the “other” gender category (1.05), the marital status category “domestic partner” (0.90), the employment status category “unemployed and looking for a job” (1.05), the education category “up to 8 years” (0.81), the religious service attendance categories “a few times a year” and “never” (0.71), and the immigration status category “born in another country” (0.73). This suggests particularly strong variation in mean gratitude for some sociodemographic categories. Further, I^2^ values are above 95% across all demographic categories, further suggesting that variability in mean gratitude across countries is likely due to heterogeneity rather than sampling variability.

Drawing on the heterogeneity estimate (“tau”) and global *p*-values shown in Table 3 (Tables S1b-S22b in the Supplemental File), we examine the variation in the mean gratitude for each sociodemographic group separately by country (forest plots in Figures S1 to S34 also provide a visual display of country-specific mean gratitude for each sociodemographic category). For instance, we found that those who attended religious services more than once a week had the highest mean gratitude in all but two countries (i.e., the Philippines and Egypt). The differences between the lowest and highest mean gratitude within this category (i.e., frequency of religious attendance) across countries range from 0.22 to 2.79, with the highest difference found in Hong Kong (Never: 6.20, 95%CI: 6.04, 6.36, > 1/week: 8.99, 95%CI: 8.77, 9.20). Although the differences in means gratitude across levels of religious participation within each country were not large, we found that, in most countries, the mean gratitude tends to increase with age in most countries except for India, Indonesia, Israel, the Philippines, and Tanzania, which show the highest mean gratitude in younger age groups below the age of 40.

We also found that the pattern of differences in mean gratitude by educational category was mostly consistent across the countries, except for Nigeria and Hong Kong. While the higher education groups (9–15 years or 16 + years) had the highest mean gratitude in most countries, the highest education group (16 + years) had the lowest mean gratitude in four countries (Nigeria: 8.33, Hong Kong: 6.8, Türkiye: 6.51, and Argentina: 8.32). However, the difference in mean gratitude across the educational attainment categories in each country was not large (ranging from 0.09 in Mexico to 1.18 in Japan). In terms of employment, while the mean gratitude for the retired or self-employed categories showed the highest mean gratitude in many countries, the retired category had the lowest mean gratitude in India (retired: 6.90), Indonesia (retired: 8.79), and the Philippines (retired: 8.27). The levels of gratitude for those who are looking for a job or are unemployed were consistently lower, except for Türkiye and Sweden. Students had the highest mean gratitude in India (8.1), Indonesia (9.16), Israel (8.41), Japan (6.72), Kenya (8.28), and the Philippines (8.76). The difference in mean gratitude between the highest category and the lowest in each country ranged from 0.36 (Kenya) to 2.22 (United Kingdom).

Additional information on the magnitude of differences can be found in the Supplemental File, where the standardized pairwise differences between groups for each demographic variable are reported for the pooled estimates and by country (see Figures S36-115). For instance, pooled cross-country estimates showed that the difference in standardized mean gratitude across categories for the demographic variables ranged from − 0.24 SD (age group comparison of 18–24 vs. 80 or older) to 0.49 SD (religious service attendance group comparison of more than once a week vs. never). The standardized mean differences in gratitude were smaller for several age group comparisons (18–24 vs. 30–39: −0.03 SD; 18–24 vs. 40–49: −0.04 SD; 25–29 vs. 30–39: −0.01 SD; 25–29 vs. 40–49: −0.02 SD; 30–39 vs. 40–49: −0.02 SD), three marital status comparisons (married vs. widowed: 0.01 SD; separated vs. single: 0.01 SD; divorced vs. domestic: −0.01 SD), and two employment status comparisons (employed vs. homemaker: −0.00 SD; self-employed vs. retired: −0.01 SD), while three frequency of religious participation comparisons (1/week vs. never: 0.32 SD; >1/week vs. never: 0.49 SD; >1/week vs. a few times a year: 0.31 SD), one employment status comparison (retired vs. unemployed: 0.28 SD), and two gender comparisons (female vs. other: 0.41 SD; male vs. other: 0.28 SD) showed somewhat larger standardized mean differences in gratitude. Standardized mean differences between groups for each demographic variable showed some variation across countries. For example, the largest standardized difference between those who attended religious services more than once a week and those who attended once a week (see Figure S102) was observed in Hong Kong (0.59 SD), while standardized mean differences between these two groups were more negligible in Indonesia (0.03 SD), Kenya (0.01 SD), and the Philippines (−0.05 SD).

Table 3 shows pooled results for the sociodemographic characteristics that were measured consistently across countries; religious affiliation and race/ethnicity were not included, as these variables were not assessed consistently across the countries. The mean gratitude across religious affiliation categories and race/ethnicity can be found in Tables S1b-S22b in the Supplemental File. Although we found some evidence of differences in mean gratitude by categories of these variables, there were no specific patterns across countries. Respondents with Christian affiliation are present in all 22 countries and display relatively high mean scores in 10 countries, often above 8, particularly in the United States (8.58), Mexico (8.92), Indonesia (9.08), India (8.70), the Philippines (8.64), and Kenya (8.12). Among these 22 countries, 3 countries showed the highest gratitude for Christianity, including Japan, Indonesia, and India. Respondents with Muslim affiliation are present in 16 countries, and 6 countries showed mean scores higher than 8 among this group, notably in Argentina (9.86), Indonesia (8.92), Mexico (10), Nigeria (8.24), the Philippines (8.58), and the United Kingdom (8.36). Some minority religious affiliations showed the highest gratitude scores in some countries. For instance, Shinto presented the highest mean gratitude in the United States, South Africa, and Hong Kong. The group of people who have no religious affiliation had the highest mean gratitude score (8.86) in Nigeria. We did not find consistent patterns for race/ethnicity across the countries. For instance, there is no specific race/ethnicity that had the highest mean gratitude across countries.

Table S23 in the Supplemental File provides analyses that complement the analyses presented in Table 3. While Table 3 shows a random-effects meta-analysis that treats each person in the 22 countries equally by assuming that the proportion in each country was drawn from the underlying distribution of the 22 countries included in the study, Table S23 shows population-weighted meta-analyses in which each country’s results were weighted by the actual 2023 population size. Results of the random-effects and population-weighted meta-analyses are mostly aligned. For instance, both sets of results suggested that higher age, being a women, higher education, more frequent religious service attendance, and being married are associated with higher mean gratitude. The mean gratitude was consistently higher for the population-weighted meta-analysis for education. However, we observed some differences between the two approaches, such as the mean gratitude for those who were born in the country, which had a higher mean gratitude in the population-weighted approach (7.91) compared to the random-effects approach (7.84). In the category of religious service attendance, two categories (1–3 times/week and never) had higher mean gratitude in the population-weighted meta-analytic results, whereas other categories had lower mean gratitude for the population-weighted results compared to the random-effects results.

## Discussion

In this study, we used a nationally representative dataset that includes 202,898 individuals from 22 countries to explore the distribution of mean gratitude scores across countries and key demographic groups within and across countries. Our main findings are threefold. First, we found that the distribution of gratitude varies across countries. The highest mean gratitude was in Indonesia, while the lowest was in Japan. Second, when estimates are pooled across countries, those with higher socioeconomic status (i.e., higher educational status), older age groups (e.g., 60–69, 70–79, or 80 or older), women, those who are married, retired/self-employed, and those who attend religious services more regularly tend to report the highest gratitude compared to their counterparts among 22 countries. Third, we found evidence of cross-country variability in the distribution of gratitude for each sociodemographic variable, suggesting that the pattern of sociodemographic differences varies to some extent across countries.

### Cross-national Variation in Gratitude Levels

Our findings concerning the distribution of gratitude in 22 countries diverged somewhat from previous studies. For instance, while we found that Indonesia, Mexico, and the Philippines had the greatest mean gratitude, previous research indicates otherwise (McGrath, 2015; Park et al., 2006). The differences may come from differences in the representation of the data or methodological factors, including differences in sampling strategies (e.g., convenient sampling vs. random sampling), sample, measurement approaches (e.g., single item vs. multi-item measures), and timing of data collection. For instance, the sample size of one study ranged from 20 (Poland) to 8357 (United States) across countries, which raises questions about the representativeness of such findings (McGrath, 2015; Park et al., 2006). To our knowledge, the current study is the first to leverage multinational data collected within a relatively short window of time to provide nationally representative benchmarks of gratitude in 22 countries. As subsequent waves of data collection for the GFS are completed, such benchmarking could be used to evaluate population-level changes in gratitude that local policymakers and public health experts might draw on in their efforts to promote gratitude.

One possible explanation for the cross-country variation in mean gratitude that we observed is cross-country differences in religiosity (Cowden et al., 2025). For instance, although Indonesia and Japan are both island Asian countries, the mean gratitude was highest in Indonesia and lowest in Japan. While 61% of the Japanese sample reported having no religion and a majority (77%) never attend religious services, 92% of those in the Indonesian sample are adherents of Islam, 7.2% identify as Christian, and only a small fraction (4.8%) never attend religious services. Many religions encourage gratefulness as a virtue and emphasize expressing thankfulness to a higher power, such as God, for the everyday blessings they receive (Portocarrero et al., 2020; Rosmarin et al., 2011). Religious participation might influence gratitude through the experiences that take place in sacred worship spaces (Counted et al., 2024; Krause, 2009; Rosmarin et al., 2011), which could partly explain differences in mean gratitude across countries.

Alternatively, cross-country variations in mean gratitude that were observed may be due to differences in population demographics, sociocultural factors, or cultural factors, as supported by socioecological system theory, which explains that human development is shaped by the interaction of several systems, including individual-, family-, neighborhood-, culture, or political-levels (Bronfenbrenner & Others, 1994). Although speculative, variation in mean gratitude across countries might also be explained by the racial/ethnic diversity of these countries. While Indonesia consists of multiple races and ethnicities, Japan is a monoracial country. It may be that gratitude is an important factor in creating cohesiveness in the population, especially when individuals do not share the same cultural norms due to their racial or ethnic backgrounds (Rosmarin et al., 2011).

It is also important to note that differences across countries may arise due to translations and cultural differences in the interpretation of the items. For example, people in different cultures may interpret and respond to the gratitude item differently (Cowden et al., 2024). One possible influencing factor is religious beliefs and practices, which may color or impact the way a person approaches the gratitude item. In countries that are more religious, it may be that responses to the gratitude item are more strongly influenced by religion. Variation in gratitude across countries may also be impacted by differences in how people in different cultures typically respond to rating-scale survey items. For instance, in Asian countries, especially Japan, the distribution of responses to 0–10 scales tends to be concentrated in the middle rather than spread across a wider range (Heine et al., 2002).

### Sociodemographic Differences in Gratitude Levels

Results from the random-effects meta-analyses provided evidence of differences in mean gratitude across the 22 countries for each sociodemographic characteristic. Specifically, we found that those with higher socioeconomic status (i.e., more years of education), older age groups (e.g., 60–69, 70–79, or 80 or older), women, retired/self-employed, those who are married, and those who attend religious services more regularly reported higher gratitude. This trend somewhat corresponds with previous research. One framework through which to consider the findings of this study is socioecological systems theory, which emphasizes the complex interplay of multiple factors across different layers of the socioecological system (e.g., microsystem, mesosystem) in shaping individual experiences (including gratitude) (Bronfenbrenner & Others, 1994). For example, married individuals may receive more social support from their partner, while those with higher educational status or those who are employed may receive better support (e.g., provide resources or information, provide emotional support) as they are more likely to be surrounded by individuals with a similar socioeconomic position (e.g., microsystem) (Mccullough et al., 2002; Wood et al., 2010).

Prior research exploring differences in gratitude by sociodemographic factors suggests that higher gratitude is associated with being older, a woman, more highly educated, and employed among Dutch adults (Jans-Beken et al., 2018), which aligns with our findings. Gender differences in gratitude may be linked to differing expectations in expressing gratitude (Canary & Dindia, n.d.), as other studies have suggested that gender differences may stem from varying social-emotional skills and diverse social expectations (Rosmarin et al., 2011). Specifically, one prior study pointed out that there may be preferred characteristics in society by gender (Grusec P, n.d.; Kashdan et al., 2009). For example, men prefer to be strong or aggressive, while women are expected to maintain social bonds by being kind and expressing gratefulness (Kashdan et al., 2009). Another study suggested that gender differences may arise from the interpretation of items used in the study (Heintz et al., 2019). Further, our meta-analytic results indicate there are substantial heterogeneities across the countries for each sociodemographic category (Heintz et al., 2019), suggesting that the levels of gratitude may be determined by cultural factors.

### Cross-national Variation in Sociodemographic Differences

We observed some cross-country variation in sociodemographic differences. Specifically, the distribution of gratitude by frequency of religious service attendance varied somewhat by country. We found that while the main trend is that individuals who attend religious services more than once a week generally report higher levels of gratitude, Egypt and the Philippines showed different patterns. In Egypt, individuals who never attended religious services reported the highest levels of gratitude (7.91, 95%CI = 7.79, 8.02), while in the Philippines, those who attended religious services once a week reported the highest gratitude (8.74, 95%CI: 8.65, 8.85). The Philippines is a predominantly Christian country (93%), but only 16% of the population participates in religious services more than once a week, while the majority (62%) attend once a week or 1–3 times per month. Although the *p*-value testing the null hypothesis that the mean gratitude for each category is the same is significant for the Philippines, the means for most categories (except for the group that never attends religious services) are fairly similar. In contrast to the Philippines, Egypt may have other mechanisms that explain the contradictory distribution of gratitude by the frequency of religious attendance. In Egypt, where the majority of individuals reported Islam as their religious affiliation, a significant portion (44%) reported never attending religious services. Other factors, such as lifestyle, neighborhood conditions, or the political climate, might also influence their levels of gratitude. Similarly, religious involvement outside of worship services might affect their gratitude; however, our single-item measure of religious service attendance may have overlooked these aspects (Rosmarin et al., 2011).

Aside from religious service attendance, our findings concerning the overall trend of mean gratitude distribution across various sociodemographic characteristics—such as educational attainment, marital status, and age—were consistent with a considerable body of research on character, virtue, and psychological well-being (Diener & Biswas-Diener, 2011; Diener et al., 1999; VanderWeele, 2017). However, these trends did not hold true for certain countries once we examined them individually. For instance, the random-effects meta-analyses indicated that older people tend to report higher gratitude, whereas country-specific results showed that this trend did not remain for some countries, including Israel, the Philippines, Tanzania, and India. There was no specific distribution of age or education to explain the discrepancies compared with other countries. The discrepancies may be due to socioeconomic and labor market realities or cultural norms about the expression of gratitude that we did not explore in our analysis. However, the differences in mean gratitude between each category in specific countries were small compared to the range of gratitude responses (i.e., 0 to 10), suggesting the differences are more negligible.

Nevertheless, our pooled results suggest that those with more frequent religious participation exhibit higher gratitude, indicating that the cultivation of gratitude may be closely tied to religious beliefs, practices, and behaviors they learn through religious practices (Emmons & Kneezel, 2005; Portocarrero et al., 2020). Further, it is worth noting that in Japan, where the majority of the population has no specific religion, a similar trend was observed: those who participate in religious activities more than once a week and those with Christianity display higher gratitude, providing some support for theorizing that religious service attendance and religiosity are related to gratitude (Rosmarin et al., 2011). Although more detailed analyses are necessary to investigate the possible causes of the observed cross-national variation and sociodemographic differences, our findings resonate with the idea that gratitude is influenced by the characteristics of the sociocultural context in which individuals live (Bronfenbrenner & Others, 1994).

### Strengths and Limitations

Our findings should be interpreted in light of several methodological limitations. First, although gratitude was assessed with an item from a validated and widely used measure of gratitude (Mccullough et al., 2002), cognitive interviews and pilot testing that was performed during the survey development phase of the GFS suggested that responses from specific countries (e.g., Indonesia) tended to gravitate toward the upper response options (Crabtree et al., 2021/2025), signaling potential cross-sectional response biases or measurement non-equivalence (Cowden et al., 2024). Further, while single items are commonly used in large-scale epidemiologic studies like the GFS to reduce test-taker fatigue and provide a cost-effective way of assessing a wide range of constructs, there are limitations to using single items that should be considered when interpreting the findings of this study (Allen et al., 2022). For example, our measure may capture the breadth of dispositional gratitude while potentially overlooking aspects such as intensity, frequency, or temporal stability. Future research examining different dimensions of gratitude (e.g., momentary experiences or specific facets) will provide more nuanced insights into population-level gratitude. Second, caution is needed in interpreting cross-national differences, as these may be influenced by matters of translation, different modes of assessment, variation in interpretation of items and response patterns, and seasonal effects arising from data being collected in different countries at different times of the year. Third, the first wave of the GFS included 22 countries, offering a geographically and culturally diverse multinational sample. Although Wave 1 of the GFS represents around half of the global population, many cultures and contexts may not be represented in the study. Further research is needed to assess whether our findings can be generalized to countries not included in this study. Fourth, our results are descriptive and used cross-sectional data and therefore do not provide evidence of causality. Further exploration of causal questions will be possible as subsequent waves of the GFS become available. Fifth, we did not consider country-level predictors of gratitude levels in this study. Future studies could build on our findings by examining country-level predictors, including economic indicators, healthcare quality, gender inequity, or individualism.

Despite these limitations, our study has several strengths that help to advance the empirical literature on gratitude. For example, this is the first study to use large samples that were weighted to be approximately nationally representative, enabling us to estimate the distribution of gratitude across multiple countries and identify subpopulations that may be especially likely to benefit from resources and supports to promote gratitude. Our findings could inform the development of targeted public health initiatives aimed at promoting gratitude. By assessing gratitude levels across various countries and population subgroups, we can identify those who would benefit most from such programs. For instance, our study suggests that prioritizing interventions to promote gratitude among individuals with lower socioeconomic status, younger age groups, men, the unemployed or students, single or divorced individuals, and those who do not attend religious services regularly may be the most beneficial and effective approach to improving gratitude levels across the population.

In conclusion, this multinational study provides evidence on the distribution of gratitude in 22 geographically and culturally diverse countries around the world, highlighting sociodemographic variations both within and across these nations. Our research establishes the groundwork for understanding gratitude from an epidemiological perspective, though additional investigation is needed to understand the causes and consequences of gratitude across diverse global populations. As the GFS cohort is established through subsequent years of data collection, the panel data will provide a valuable opportunity to assess changes in gratitude over time. Such evidence will provide important insight into developing public health strategies and policies oriented toward the promotion of gratitude within different populations, monitoring progress against targets, and making well-informed decisions about modifications that may be needed to improve the effectiveness of public health efforts to promote gratitude.

## Electronic supplementary material

Below is the link to the electronic supplementary material.

Supplementary Material 1

## Data Availability

Data are publicly available through the Center for Open Science (https://www.cos.io/gfs). The research questions, variables, and analytic plan for the current study were preregistered with the Center for Open Science prior to accessing data (https://osf.io/zugyn). All code to reproduce analyses is openly available in an online repository
