# Supplementary material for: Sociodemographic Variation in Gratitude Using a Cross-National Analysis with 22 Countries
Source: Int J Appl Posit Psychol. 2025 Oct 13;10(4):60. doi: 10.1007/s41042-025-00254-w (PMC12518394; doi:10.1007/s41042-025-00254-w)

Sociodemographic Variation in Gratitude Across 22 Countries using Cross-National Analysis

**SUPPLEMENTAL FILE**

Table S1a-S22a. Nationally Representative Descriptive Statistics for Each Country.

Table S1b-S22b. Mean of Gratitude by Sociodemographic Categories for Each Country.

Table S23. Population Weighted Meta-Analyses for the Mean Gratitude by Sociodemographic Category.

Figure S1-S34. The Means of Gratitude for Each Sociodemographic Category in All 22 Countries.

Figure S35-S115. Forest plots of standardized effect sizes (standardized mean differences) of pairwise differences in gratitude means across countries.

Table S1a. Nationally representative descriptive statistics for Argentina

| **Characteristic** | **N = 6,724**^1^ |
| --- | --- |
| **Age group** |  |
| 18-24 | 1,108 (16%) |
| 25-29 | 719 (11%) |
| 30-39 | 1,432 (21%) |
| 40-49 | 1,254 (19%) |
| 50-59 | 1,014 (15%) |
| 60-69 | 730 (11%) |
| 70-79 | 356 (5.3%) |
| 80 or older | 112 (1.7%) |
| Missing | 0 (0%) |
| **Gender** |  |
| Male | 3,143 (47%) |
| Female | 3,542 (53%) |
| Other | 21 (0.3%) |
| Missing | 18 (0.3%) |
| **Marital status** |  |
| Married | 1,565 (23%) |
| Separated | 455 (6.8%) |
| Divorced | 321 (4.8%) |
| Widowed | 401 (6.0%) |
| Never | 2,381 (35%) |
| Domestic Partner | 1,514 (23%) |
| Missing | 88 (1.3%) |
| **Employment** |  |
| Employed for an employer | 2,440 (36%) |
| Self-employed | 1,748 (26%) |
| Retired | 773 (11%) |
| Student | 354 (5.3%) |
| Homemaker | 639 (9.5%) |
| Unemployed and looking for a job | 569 (8.5%) |
| None of these/other | 179 (2.7%) |
| Missing | 22 (0.3%) |
| **Religious service attendance** |  |
| >1/week | 532 (7.9%) |
| 1/week | 773 (12%) |
| 1-3/month | 461 (6.8%) |
| A few times a year | 1,949 (29%) |
| Never | 2,982 (44%) |
| Missing | 27 (0.4%) |
| **Education** |  |
| up to 8 years | 2,263 (34%) |
| 9-15 years | 3,823 (57%) |
| 16+years | 635 (9.4%) |
| Missing | 3 (<0.1%) |
| **Immigration** |  |
| Born in this country | 6,346 (94%) |
| Born in another country | 348 (5.2%) |
| Missing | 29 (0.4%) |
| **Religious affiliation** |  |
| Christianity | 4,992 (74%) |
| Islam | 9 (0.1%) |
| Hinduism | 6 (<0.1%) |
| Buddhism | 35 (0.5%) |
| Judaism | 40 (0.6%) |
| Sikhism | 0 (<0.1%) |
| Taoism | 2 (<0.1%) |
| Confucianism | 0 (<0.1%) |
| Primal, Animist, or Folk religion | 19 (0.3%) |
| Some other religion | 156 (2.3%) |
| No religion/Atheist/Agnostic | 1,352 (20%) |
| Missing | 111 (1.7%) |
| **Race and ethnicity** |  |
| Asian | 43 (0.6%) |
| Black | 95 (1.4%) |
| Indigenous | 129 (1.9%) |
| Mestizo(a) | 1,801 (27%) |
| Mullato(a) | 75 (1.1%) |
| Other | 104 (1.5%) |
| White | 3,406 (51%) |
| Missing | 1,070 (16%) |
| ^1^n (%) | |

Table S1b. Means by demographic category for Argentina

| Variable | Category | Mean | SE | 95% CI | Global p-value |
| --- | --- | --- | --- | --- | --- |
| Age group | 18-24 | 8.08 | 0.10 | (7.88, 8.27) | <0.001 |
|  | 25-29 | 8.42 | 0.12 | (8.19, 8.65) |  |
|  | 30-39 | 8.47 | 0.08 | (8.33, 8.62) |  |
|  | 40-49 | 8.66 | 0.07 | (8.52, 8.79) |  |
|  | 50-59 | 8.82 | 0.07 | (8.68, 8.96) |  |
|  | 60-69 | 8.68 | 0.10 | (8.49, 8.87) |  |
|  | 70-79 | 8.73 | 0.16 | (8.40, 9.05) |  |
|  | 80 or older | 9.00 | 0.24 | (8.52, 9.48) |  |
| Gender | Male | 8.31 | 0.05 | (8.20, 8.42) | <0.001 |
|  | Female | 8.73 | 0.04 | (8.65, 8.81) |  |
|  | Other | 9.03 | 0.42 | (8.15, 9.91) |  |
| Marital status | Married | 8.76 | 0.07 | (8.63, 8.89) | <0.001 |
|  | Separated | 8.63 | 0.11 | (8.41, 8.86) |  |
|  | Divorced | 8.69 | 0.13 | (8.43, 8.95) |  |
|  | Widowed | 8.70 | 0.14 | (8.43, 8.97) |  |
|  | Never | 8.29 | 0.06 | (8.16, 8.41) |  |
|  | Domestic Partner | 8.59 | 0.07 | (8.45, 8.72) |  |
| Employment | Employed for an employer | 8.48 | 0.05 | (8.37, 8.58) | <0.001 |
|  | Self-employed | 8.53 | 0.07 | (8.39, 8.67) |  |
|  | Retired | 8.80 | 0.10 | (8.61, 8.99) |  |
|  | Student | 7.99 | 0.16 | (7.67, 8.31) |  |
|  | Homemaker | 8.91 | 0.11 | (8.70, 9.12) |  |
|  | Unemployed and looking for a job | 8.45 | 0.12 | (8.21, 8.70) |  |
|  | None of these/other | 8.20 | 0.28 | (7.66, 8.75) |  |
| Religious service attendance | >1/week | 9.48 | 0.09 | (9.31, 9.64) | <0.001 |
|  | 1/week | 8.98 | 0.08 | (8.81, 9.14) |  |
|  | 1-3/month | 8.52 | 0.14 | (8.24, 8.79) |  |
|  | A few times a year | 8.64 | 0.06 | (8.53, 8.76) |  |
|  | Never | 8.18 | 0.06 | (8.07, 8.29) |  |
| Education | up to 8 years | 8.78 | 0.07 | (8.63, 8.92) | <0.001 |
|  | 9-15 years | 8.42 | 0.04 | (8.35, 8.50) |  |
|  | 16+years | 8.32 | 0.09 | (8.15, 8.49) |  |
| Immigration status | Born in this country | 8.53 | 0.03 | (8.46, 8.60) | 0.998 |
|  | Born in another country | 8.57 | 0.19 | (8.20, 8.93) |  |
| Religious affiliation | Christianity | 8.69 | 0.04 | (8.62, 8.77) | <0.001 |
|  | Islam | 9.86 | 0.11 | (9.59, 10.13) |  |
|  | Hinduism | 7.45 | 0.64 | (5.61, 9.30) |  |
|  | Buddhism | 8.32 | 0.45 | (7.39, 9.24) |  |
|  | Judaism | 7.53 | 0.48 | (6.55, 8.50) |  |
|  | Taoism | 5.95 |  |  |  |
|  | Primal, Animist, or Folk religion | 8.32 | 0.50 | (7.26, 9.39) |  |
|  | Some other religion | 8.90 | 0.19 | (8.53, 9.27) |  |
|  | No religion/Atheist/Agnostic | 7.95 | 0.09 | (7.78, 8.12) |  |
| Race and ethnicity | Asian | 8.36 | 0.47 | (7.42, 9.31) | 0.992 |
|  | Black | 8.85 | 0.38 | (8.09, 9.60) |  |
|  | Indigenous | 8.34 | 0.28 | (7.78, 8.90) |  |
|  | Mestizo(a) | 8.53 | 0.07 | (8.40, 8.66) |  |
|  | Mullato(a) | 8.62 | 0.35 | (7.91, 9.34) |  |
|  | Other | 8.36 | 0.34 | (7.66, 9.06) |  |
|  | White | 8.54 | 0.04 | (8.45, 8.62) |  |

Table S2a. Nationally representative descriptive statistics for Australia

| **Characteristic** | **N = 3,844**^1^ |
| --- | --- |
| **Age group** |  |
| 18-24 | 345 (9.0%) |
| 25-29 | 282 (7.3%) |
| 30-39 | 641 (17%) |
| 40-49 | 618 (16%) |
| 50-59 | 691 (18%) |
| 60-69 | 589 (15%) |
| 70-79 | 498 (13%) |
| 80 or older | 178 (4.6%) |
| Missing | 2 (<0.1%) |
| **Gender** |  |
| Male | 1,861 (48%) |
| Female | 1,941 (50%) |
| Other | 36 (0.9%) |
| Missing | 6 (0.2%) |
| **Marital status** |  |
| Married | 1,797 (47%) |
| Separated | 158 (4.1%) |
| Divorced | 332 (8.6%) |
| Widowed | 215 (5.6%) |
| Never | 855 (22%) |
| Domestic Partner | 450 (12%) |
| Missing | 38 (1.0%) |
| **Employment** |  |
| Employed for an employer | 1,881 (49%) |
| Self-employed | 380 (9.9%) |
| Retired | 912 (24%) |
| Student | 190 (5.0%) |
| Homemaker | 137 (3.6%) |
| Unemployed and looking for a job | 134 (3.5%) |
| None of these/other | 206 (5.4%) |
| Missing | 4 (0.1%) |
| **Religious service attendance** |  |
| >1/week | 162 (4.2%) |
| 1/week | 299 (7.8%) |
| 1-3/month | 135 (3.5%) |
| A few times a year | 656 (17%) |
| Never | 2,584 (67%) |
| Missing | 7 (0.2%) |
| **Education** |  |
| up to 8 years | 70 (1.8%) |
| 9-15 years | 2,434 (63%) |
| 16+years | 1,330 (35%) |
| Missing | 10 (0.3%) |
| **Immigration** |  |
| Born in this country | 2,953 (77%) |
| Born in another country | 885 (23%) |
| Missing | 6 (0.2%) |
| **Religious affiliation** |  |
| Christianity | 1,592 (41%) |
| Islam | 45 (1.2%) |
| Hinduism | 31 (0.8%) |
| Buddhism | 36 (0.9%) |
| Judaism | 26 (0.7%) |
| Sikhism | 8 (0.2%) |
| Baha’i | 7 (0.2%) |
| Taoism | 5 (0.1%) |
| Primal, Animist, or Folk religion | 23 (0.6%) |
| Some other religion | 39 (1.0%) |
| No religion/Atheist/Agnostic | 2,020 (53%) |
| Missing | 15 (0.4%) |
| **Race and ethnicity** |  |
| Aboriginal | 53 (1.4%) |
| Australian | 1,946 (51%) |
| Australian British/European | 1,047 (27%) |
| Chinese | 75 (1.9%) |
| Indian | 58 (1.5%) |
| Japanese | 1 (<0.1%) |
| Malay | 11 (0.3%) |
| New Zealander | 91 (2.4%) |
| Other | 163 (4.2%) |
| Other European | 357 (9.3%) |
| Russian | 7 (0.2%) |
| Samoan | 4 (0.1%) |
| Sinhalese | 1 (<0.1%) |
| Spanish | 2 (<0.1%) |
| Sri Lankan Moor | 1 (<0.1%) |
| Sri Lankan Tamil | 7 (0.2%) |
| Vietnamese | 7 (0.2%) |
| Missing | 14 (0.4%) |
| ^1^n (%) | |

Table S2b. Means by demographic category for Australia

| Variable | Category | Mean | SE | 95% CI | Global p-value |
| --- | --- | --- | --- | --- | --- |
| Age group | 18-24 | 7.34 | 0.20 | (6.94, 7.74) | <0.001 |
|  | 25-29 | 7.55 | 0.19 | (7.17, 7.93) |  |
|  | 30-39 | 7.49 | 0.14 | (7.21, 7.78) |  |
|  | 40-49 | 7.77 | 0.14 | (7.49, 8.05) |  |
|  | 50-59 | 7.71 | 0.11 | (7.49, 7.93) |  |
|  | 60-69 | 8.06 | 0.09 | (7.89, 8.24) |  |
|  | 70-79 | 8.43 | 0.10 | (8.23, 8.63) |  |
|  | 80 or older | 8.69 | 0.15 | (8.40, 8.98) |  |
| Gender | Male | 7.54 | 0.07 | (7.40, 7.69) | <0.001 |
|  | Female | 8.11 | 0.07 | (7.98, 8.24) |  |
|  | Other | 7.80 | 0.49 | (6.79, 8.80) |  |
| Marital status | Married | 8.26 | 0.06 | (8.15, 8.37) | <0.001 |
|  | Separated | 6.68 | 0.27 | (6.14, 7.22) |  |
|  | Divorced | 7.86 | 0.16 | (7.54, 8.18) |  |
|  | Widowed | 8.51 | 0.14 | (8.22, 8.80) |  |
|  | Never | 7.05 | 0.12 | (6.81, 7.29) |  |
|  | Domestic Partner | 7.66 | 0.16 | (7.34, 7.98) |  |
| Employment | Employed for an employer | 7.80 | 0.07 | (7.67, 7.93) | <0.001 |
|  | Self-employed | 8.18 | 0.14 | (7.90, 8.47) |  |
|  | Retired | 8.25 | 0.08 | (8.09, 8.41) |  |
|  | Student | 7.44 | 0.27 | (6.91, 7.98) |  |
|  | Homemaker | 7.59 | 0.35 | (6.89, 8.28) |  |
|  | Unemployed and looking for a job | 6.64 | 0.39 | (5.86, 7.41) |  |
|  | None of these/other | 6.92 | 0.27 | (6.39, 7.46) |  |
| Religious service attendance | >1/week | 9.03 | 0.15 | (8.73, 9.33) | <0.001 |
|  | 1/week | 8.67 | 0.14 | (8.39, 8.96) |  |
|  | 1-3/month | 8.34 | 0.20 | (7.94, 8.74) |  |
|  | A few times a year | 7.96 | 0.11 | (7.75, 8.18) |  |
|  | Never | 7.60 | 0.06 | (7.48, 7.72) |  |
| Education | up to 8 years | 8.33 | 0.40 | (7.53, 9.12) | <0.001 |
|  | 9-15 years | 7.65 | 0.07 | (7.51, 7.79) |  |
|  | 16+years | 8.14 | 0.06 | (8.02, 8.26) |  |
| Immigration status | Born in this country | 7.78 | 0.06 | (7.67, 7.90) | <0.001 |
|  | Born in another country | 8.00 | 0.09 | (7.81, 8.18) |  |
| Religious affiliation | Christianity | 8.25 | 0.06 | (8.13, 8.37) | <0.001 |
|  | Islam | 7.64 | 0.62 | (6.39, 8.90) |  |
|  | Hinduism | 8.07 | 0.46 | (7.13, 9.00) |  |
|  | Buddhism | 8.02 | 0.51 | (6.98, 9.05) |  |
|  | Judaism | 8.20 | 0.41 | (7.34, 9.05) |  |
|  | Sikhism | 5.16 | 1.37 | (1.65, 8.66) |  |
|  | Baha’i | 9.25 | 0.49 | (7.97, 10.54) |  |
|  | Taoism | 9.65 | 0.31 | (8.52, 10.77) |  |
|  | Primal, Animist, or Folk religion | 7.80 | 0.67 | (6.41, 9.19) |  |
|  | Some other religion | 7.45 | 0.54 | (6.35, 8.55) |  |
|  | No religion/Atheist/Agnostic | 7.50 | 0.08 | (7.36, 7.65) |  |
| Race and ethnicity | Other | 7.97 | 0.26 | (7.45, 8.48) | <0.001 |
|  | Aboriginal | 6.66 | 0.63 | (5.38, 7.93) |  |
|  | Australian | 7.86 | 0.07 | (7.72, 7.99) |  |
|  | Australian British/European | 7.84 | 0.09 | (7.66, 8.02) |  |
|  | Chinese | 7.50 | 0.33 | (6.85, 8.16) |  |
|  | Indian | 7.89 | 0.46 | (6.96, 8.82) |  |
|  | Japanese | 8.91 |  |  |  |
|  | Malay | 7.71 | 0.86 | (5.77, 9.65) |  |
|  | New Zealander | 8.08 | 0.34 | (7.40, 8.75) |  |
|  | Other European | 7.87 | 0.13 | (7.60, 8.13) |  |
|  | Russian | 7.45 | 0.49 | (6.17, 8.74) |  |
|  | Samoan | 5.11 | 2.69 | (-4.83, 15.06) |  |
|  | Sinhalese | 6.57 |  |  |  |
|  | Spanish | 9.52 |  |  |  |
|  | Sri Lankan Moor | 4.32 |  |  |  |
|  | Sri Lankan Tamil | 7.26 | 0.88 | (5.00, 9.52) |  |
|  | Vietnamese | 7.18 | 0.89 | (4.90, 9.46) |  |

Table S3a. Nationally representative descriptive statistics for Brazil

| **Characteristic** | **N = 13,204**^1^ |
| --- | --- |
| **Age group** |  |
| 18-24 | 1,986 (15%) |
| 25-29 | 1,468 (11%) |
| 30-39 | 2,908 (22%) |
| 40-49 | 2,638 (20%) |
| 50-59 | 2,131 (16%) |
| 60-69 | 1,435 (11%) |
| 70-79 | 510 (3.9%) |
| 80 or older | 126 (1.0%) |
| Missing | 0 (0%) |
| **Gender** |  |
| Male | 6,320 (48%) |
| Female | 6,820 (52%) |
| Other | 35 (0.3%) |
| Missing | 30 (0.2%) |
| **Marital status** |  |
| Married | 4,646 (35%) |
| Separated | 594 (4.5%) |
| Divorced | 865 (6.5%) |
| Widowed | 408 (3.1%) |
| Never | 4,347 (33%) |
| Domestic Partner | 2,081 (16%) |
| Missing | 263 (2.0%) |
| **Employment** |  |
| Employed for an employer | 3,756 (28%) |
| Self-employed | 2,918 (22%) |
| Retired | 1,536 (12%) |
| Student | 624 (4.7%) |
| Homemaker | 1,305 (9.9%) |
| Unemployed and looking for a job | 2,419 (18%) |
| None of these/other | 448 (3.4%) |
| Missing | 199 (1.5%) |
| **Religious service attendance** |  |
| >1/week | 2,386 (18%) |
| 1/week | 2,272 (17%) |
| 1-3/month | 1,398 (11%) |
| A few times a year | 3,978 (30%) |
| Never | 3,110 (24%) |
| Missing | 61 (0.5%) |
| **Education** |  |
| up to 8 years | 3,139 (24%) |
| 9-15 years | 7,665 (58%) |
| 16+years | 2,390 (18%) |
| Missing | 10 (<0.1%) |
| **Immigration** |  |
| Born in this country | 12,688 (96%) |
| Born in another country | 153 (1.2%) |
| Missing | 363 (2.7%) |
| **Religious affiliation** |  |
| Christianity | 9,911 (75%) |
| Islam | 6 (<0.1%) |
| Hinduism | 1 (<0.1%) |
| Buddhism | 37 (0.3%) |
| Judaism | 31 (0.2%) |
| Baha’i | 2 (<0.1%) |
| Jainism | 2 (<0.1%) |
| Shinto | 1 (<0.1%) |
| Taoism | 1 (<0.1%) |
| Confucianism | 6 (<0.1%) |
| Primal, Animist, or Folk religion | 15 (0.1%) |
| Spiritism | 696 (5.3%) |
| Umbanda, Candomblé, and other African-derived religions | 525 (4.0%) |
| Some other religion | 144 (1.1%) |
| No religion/Atheist/Agnostic | 1,712 (13%) |
| Missing | 113 (0.9%) |
| **Race and ethnicity** |  |
| Amarela | 238 (1.8%) |
| Branca | 5,169 (39%) |
| Indígena | 131 (1.0%) |
| Other | 61 (0.5%) |
| Parda | 5,125 (39%) |
| Preta | 1,615 (12%) |
| Missing | 865 (6.6%) |
| ^1^n (%) | |

Table S3b. Means by demographic category for Brazil

| Variable | Category | Mean | SE | 95% CI | Global p-value |
| --- | --- | --- | --- | --- | --- |
| Age group | 18-24 | 8.03 | 0.06 | (7.90, 8.15) | <0.001 |
|  | 25-29 | 8.33 | 0.07 | (8.18, 8.47) |  |
|  | 30-39 | 8.57 | 0.05 | (8.48, 8.66) |  |
|  | 40-49 | 8.76 | 0.05 | (8.67, 8.85) |  |
|  | 50-59 | 8.95 | 0.05 | (8.85, 9.05) |  |
|  | 60-69 | 8.94 | 0.07 | (8.80, 9.09) |  |
|  | 70-79 | 8.74 | 0.17 | (8.41, 9.08) |  |
|  | 80 or older | 8.78 | 0.25 | (8.29, 9.28) |  |
| Gender | Male | 8.48 | 0.04 | (8.41, 8.55) | <0.001 |
|  | Female | 8.73 | 0.03 | (8.67, 8.79) |  |
|  | Other | 8.11 | 0.53 | (7.03, 9.20) |  |
| Marital status | Married | 8.93 | 0.03 | (8.86, 9.00) | <0.001 |
|  | Separated | 8.53 | 0.11 | (8.30, 8.75) |  |
|  | Divorced | 8.74 | 0.09 | (8.56, 8.92) |  |
|  | Widowed | 8.99 | 0.12 | (8.75, 9.22) |  |
|  | Never | 8.20 | 0.05 | (8.11, 8.29) |  |
|  | Domestic Partner | 8.64 | 0.05 | (8.54, 8.75) |  |
| Employment | Employed for an employer | 8.65 | 0.04 | (8.58, 8.72) | <0.001 |
|  | Self-employed | 8.72 | 0.04 | (8.63, 8.80) |  |
|  | Retired | 8.81 | 0.08 | (8.66, 8.97) |  |
|  | Student | 8.07 | 0.10 | (7.86, 8.27) |  |
|  | Homemaker | 8.90 | 0.07 | (8.76, 9.04) |  |
|  | Unemployed and looking for a job | 8.29 | 0.07 | (8.16, 8.42) |  |
|  | None of these/other | 8.52 | 0.15 | (8.23, 8.82) |  |
| Religious service attendance | >1/week | 9.19 | 0.05 | (9.10, 9.28) | <0.001 |
|  | 1/week | 8.77 | 0.05 | (8.67, 8.87) |  |
|  | 1-3/month | 8.75 | 0.06 | (8.64, 8.87) |  |
|  | A few times a year | 8.63 | 0.04 | (8.55, 8.70) |  |
|  | Never | 7.97 | 0.06 | (7.85, 8.09) |  |
| Education | up to 8 years | 8.64 | 0.06 | (8.54, 8.75) | <0.001 |
|  | 9-15 years | 8.55 | 0.03 | (8.49, 8.61) |  |
|  | 16+years | 8.75 | 0.04 | (8.67, 8.83) |  |
| Immigration status | Born in this country | 8.61 | 0.02 | (8.56, 8.65) | 0.687 |
|  | Born in another country | 8.74 | 0.18 | (8.38, 9.10) |  |
| Religious affiliation | Christianity | 8.75 | 0.03 | (8.70, 8.80) | <0.001 |
|  | Islam | 6.91 | 0.99 | (4.17, 9.65) |  |
|  | Hinduism | 8.13 |  |  |  |
|  | Buddhism | 8.64 | 0.37 | (7.89, 9.39) |  |
|  | Judaism | 8.06 | 0.69 | (6.65, 9.47) |  |
|  | Baha’i | 9.68 |  |  |  |
|  | Jainism | 4.61 |  |  |  |
|  | Shinto | 3.34 |  |  |  |
|  | Taoism | 6.48 |  |  |  |
|  | Confucianism | 8.02 | 0.93 | (5.45, 10.59) |  |
|  | Primal, Animist, or Folk religion | 8.53 | 0.63 | (7.18, 9.88) |  |
|  | Spiritism | 8.88 | 0.08 | (8.71, 9.04) |  |
|  | Umbanda, Candomblé, and other African-derived religions | 8.43 | 0.12 | (8.19, 8.68) |  |
|  | Some other religion | 8.79 | 0.23 | (8.34, 9.25) |  |
|  | No religion/Atheist/Agnostic | 7.78 | 0.08 | (7.63, 7.93) |  |
| Race and ethnicity | Other | 9.15 | 0.30 | (8.53, 9.77) | <0.001 |
|  | Amarela | 8.29 | 0.20 | (7.90, 8.68) |  |
|  | Branca | 8.56 | 0.04 | (8.49, 8.63) |  |
|  | Indígena | 8.61 | 0.30 | (8.02, 9.19) |  |
|  | Parda | 8.66 | 0.04 | (8.60, 8.73) |  |
|  | Preta | 8.62 | 0.06 | (8.49, 8.75) |  |

Table S4a. Nationally representative descriptive statistics for Egypt

| **Characteristic** | **N = 4,729**^1^ |
| --- | --- |
| **Age group** |  |
| 18-24 | 960 (20%) |
| 25-29 | 607 (13%) |
| 30-39 | 1,204 (25%) |
| 40-49 | 897 (19%) |
| 50-59 | 613 (13%) |
| 60-69 | 387 (8.2%) |
| 70-79 | 54 (1.1%) |
| 80 or older | 7 (0.2%) |
| Missing | 0 (0%) |
| **Gender** |  |
| Male | 2,394 (51%) |
| Female | 2,334 (49%) |
| Other | 0 (0%) |
| Missing | 0 (<0.1%) |
| **Marital status** |  |
| Married | 3,387 (72%) |
| Separated | 39 (0.8%) |
| Divorced | 101 (2.1%) |
| Widowed | 238 (5.0%) |
| Never | 947 (20%) |
| Domestic Partner | 0 (0%) |
| Missing | 17 (0.4%) |
| **Employment** |  |
| Employed for an employer | 1,267 (27%) |
| Self-employed | 892 (19%) |
| Retired | 253 (5.4%) |
| Student | 297 (6.3%) |
| Homemaker | 1,772 (37%) |
| Unemployed and looking for a job | 224 (4.7%) |
| None of these/other | 21 (0.4%) |
| Missing | 3 (<0.1%) |
| **Religious service attendance** |  |
| >1/week | 839 (18%) |
| 1/week | 960 (20%) |
| 1-3/month | 368 (7.8%) |
| A few times a year | 458 (9.7%) |
| Never | 2,091 (44%) |
| Missing | 12 (0.3%) |
| **Education** |  |
| up to 8 years | 2,486 (53%) |
| 9-15 years | 1,599 (34%) |
| 16+years | 643 (14%) |
| Missing | 1 (<0.1%) |
| **Immigration** |  |
| Born in this country | 4,713 (100%) |
| Born in another country | 16 (0.3%) |
| Missing | 1 (<0.1%) |
| **Religious affiliation** |  |
| Christianity | 120 (2.5%) |
| Islam | 4,607 (97%) |
| Taoism | 0 (<0.1%) |
| Missing | 1 (<0.1%) |
| **Race and ethnicity** |  |
| Arab | 4,585 (97%) |
| Bedouin Arab | 4 (<0.1%) |
| Greek | 1 (<0.1%) |
| Nubian | 27 (0.6%) |
| Turkish | 9 (0.2%) |
| Missing | 102 (2.2%) |
| ^1^n (%) | |

Table S4b. Means by demographic category for Egypt

| Variable | Category | Mean | SE | 95% CI | Global p-value |
| --- | --- | --- | --- | --- | --- |
| Age group | 18-24 | 7.43 | 0.11 | (7.21, 7.65) | <0.001 |
|  | 25-29 | 7.70 | 0.13 | (7.45, 7.95) |  |
|  | 30-39 | 7.74 | 0.09 | (7.57, 7.91) |  |
|  | 40-49 | 7.87 | 0.10 | (7.68, 8.06) |  |
|  | 50-59 | 7.95 | 0.12 | (7.72, 8.19) |  |
|  | 60-69 | 8.01 | 0.18 | (7.65, 8.36) |  |
|  | 70-79 | 7.55 | 0.56 | (6.42, 8.68) |  |
|  | 80 or older | 9.20 | 0.41 | (8.15, 10.25) |  |
| Gender | Male | 7.47 | 0.08 | (7.32, 7.62) | <0.001 |
|  | Female | 8.03 | 0.06 | (7.92, 8.14) |  |
| Marital status | Married | 7.83 | 0.06 | (7.72, 7.94) | <0.001 |
|  | Separated | 8.25 | 0.54 | (7.17, 9.34) |  |
|  | Divorced | 8.11 | 0.27 | (7.58, 8.64) |  |
|  | Widowed | 8.38 | 0.17 | (8.04, 8.72) |  |
|  | Never | 7.23 | 0.12 | (6.98, 7.47) |  |
| Employment | Employed for an employer | 7.63 | 0.10 | (7.42, 7.83) | <0.001 |
|  | Self-employed | 7.66 | 0.12 | (7.41, 7.90) |  |
|  | Retired | 7.78 | 0.21 | (7.37, 8.18) |  |
|  | Student | 7.15 | 0.20 | (6.77, 7.54) |  |
|  | Homemaker | 8.02 | 0.07 | (7.88, 8.15) |  |
|  | Unemployed and looking for a job | 7.37 | 0.24 | (6.90, 7.85) |  |
|  | None of these/other | 8.24 | 0.51 | (7.17, 9.32) |  |
| Religious service attendance | >1/week | 7.88 | 0.12 | (7.64, 8.11) | <0.001 |
|  | 1/week | 7.52 | 0.13 | (7.27, 7.77) |  |
|  | 1-3/month | 7.55 | 0.17 | (7.22, 7.87) |  |
|  | A few times a year | 7.43 | 0.17 | (7.09, 7.76) |  |
|  | Never | 7.91 | 0.06 | (7.79, 8.02) |  |
| Education | up to 8 years | 7.90 | 0.08 | (7.75, 8.06) | <0.001 |
|  | 9-15 years | 7.56 | 0.07 | (7.43, 7.69) |  |
|  | 16+years | 7.60 | 0.12 | (7.36, 7.84) |  |
| Immigration status | Born in this country | 7.74 | 0.05 | (7.64, 7.84) | <0.001 |
|  | Born in another country | 9.58 | 0.16 | (9.23, 9.93) |  |
| Religious affiliation | Christianity | 7.10 | 0.39 | (6.33, 7.87) | 0.008 |
|  | Islam | 7.76 | 0.05 | (7.66, 7.87) |  |
| Race and ethnicity | Arab | 7.75 | 0.05 | (7.65, 7.85) | <0.001 |
|  | Bedouin Arab | 3.71 | 0.00 | (3.71, 3.71) |  |
|  | Nubian | 7.28 | 0.09 | (7.09, 7.48) |  |
|  | Turkish | 8.55 | 0.62 | (7.10, 10.00) |  |

Table S5a. Nationally representative descriptive statistics for Germany

| **Characteristic** | **N = 9,506**^1^ |
| --- | --- |
| **Age group** |  |
| 18-24 | 829 (8.7%) |
| 25-29 | 774 (8.1%) |
| 30-39 | 1,438 (15%) |
| 40-49 | 1,494 (16%) |
| 50-59 | 1,729 (18%) |
| 60-69 | 1,915 (20%) |
| 70-79 | 1,137 (12%) |
| 80 or older | 190 (2.0%) |
| Missing | 0 (0%) |
| **Gender** |  |
| Male | 4,641 (49%) |
| Female | 4,843 (51%) |
| Other | 11 (0.1%) |
| Missing | 11 (0.1%) |
| **Marital status** |  |
| Married | 4,784 (50%) |
| Separated | 219 (2.3%) |
| Divorced | 767 (8.1%) |
| Widowed | 409 (4.3%) |
| Never | 2,627 (28%) |
| Domestic Partner | 619 (6.5%) |
| Missing | 81 (0.9%) |
| **Employment** |  |
| Employed for an employer | 4,950 (52%) |
| Self-employed | 712 (7.5%) |
| Retired | 2,480 (26%) |
| Student | 605 (6.4%) |
| Homemaker | 251 (2.6%) |
| Unemployed and looking for a job | 288 (3.0%) |
| None of these/other | 204 (2.1%) |
| Missing | 14 (0.2%) |
| **Religious service attendance** |  |
| >1/week | 285 (3.0%) |
| 1/week | 424 (4.5%) |
| 1-3/month | 550 (5.8%) |
| A few times a year | 2,362 (25%) |
| Never | 5,876 (62%) |
| Missing | 9 (<0.1%) |
| **Education** |  |
| up to 8 years | 235 (2.5%) |
| 9-15 years | 6,094 (64%) |
| 16+years | 3,164 (33%) |
| Missing | 13 (0.1%) |
| **Immigration** |  |
| Born in this country | 8,722 (92%) |
| Born in another country | 744 (7.8%) |
| Missing | 40 (0.4%) |
| **Religious affiliation** |  |
| Christianity | 5,052 (53%) |
| Islam | 351 (3.7%) |
| Hinduism | 12 (0.1%) |
| Buddhism | 51 (0.5%) |
| Judaism | 19 (0.2%) |
| Sikhism | 5 (<0.1%) |
| Baha’i | 3 (<0.1%) |
| Shinto | 2 (<0.1%) |
| Taoism | 0 (<0.1%) |
| Confucianism | 4 (<0.1%) |
| Primal, Animist, or Folk religion | 34 (0.4%) |
| Some other religion | 60 (0.6%) |
| No religion/Atheist/Agnostic | 3,815 (40%) |
| Missing | 99 (1.0%) |
| **Race and ethnicity** |  |
| Missing | 9,506 (100%) |
| ^1^n (%) | |

Table S5b. Means by demographic category for Germany

| Variable | Category | Mean | SE | 95% CI | Global p-value |
| --- | --- | --- | --- | --- | --- |
| Age group | 18-24 | 7.44 | 0.11 | (7.22, 7.66) | <0.001 |
|  | 25-29 | 7.73 | 0.09 | (7.54, 7.91) |  |
|  | 30-39 | 7.58 | 0.07 | (7.45, 7.72) |  |
|  | 40-49 | 7.55 | 0.07 | (7.42, 7.68) |  |
|  | 50-59 | 7.45 | 0.07 | (7.31, 7.59) |  |
|  | 60-69 | 7.74 | 0.06 | (7.62, 7.87) |  |
|  | 70-79 | 7.84 | 0.08 | (7.68, 8.00) |  |
|  | 80 or older | 8.41 | 0.16 | (8.09, 8.72) |  |
| Gender | Male | 7.50 | 0.04 | (7.42, 7.59) | <0.001 |
|  | Female | 7.76 | 0.04 | (7.69, 7.84) |  |
|  | Other | 5.08 | 0.48 | (3.98, 6.18) |  |
| Marital status | Married | 7.82 | 0.04 | (7.75, 7.90) | <0.001 |
|  | Separated | 7.45 | 0.18 | (7.09, 7.81) |  |
|  | Divorced | 7.49 | 0.11 | (7.29, 7.70) |  |
|  | Widowed | 7.68 | 0.15 | (7.38, 7.98) |  |
|  | Never | 7.30 | 0.06 | (7.19, 7.41) |  |
|  | Domestic Partner | 7.80 | 0.09 | (7.61, 7.98) |  |
| Employment | Employed for an employer | 7.60 | 0.04 | (7.52, 7.68) | <0.001 |
|  | Self-employed | 7.93 | 0.10 | (7.74, 8.13) |  |
|  | Retired | 7.76 | 0.05 | (7.65, 7.86) |  |
|  | Student | 7.57 | 0.12 | (7.33, 7.81) |  |
|  | Homemaker | 7.26 | 0.17 | (6.92, 7.60) |  |
|  | Unemployed and looking for a job | 7.12 | 0.18 | (6.77, 7.46) |  |
|  | None of these/other | 7.31 | 0.21 | (6.90, 7.72) |  |
| Religious service attendance | >1/week | 8.33 | 0.14 | (8.07, 8.60) | <0.001 |
|  | 1/week | 8.08 | 0.12 | (7.85, 8.31) |  |
|  | 1-3/month | 8.15 | 0.10 | (7.95, 8.36) |  |
|  | A few times a year | 7.96 | 0.05 | (7.86, 8.06) |  |
|  | Never | 7.39 | 0.04 | (7.31, 7.46) |  |
| Education | up to 8 years | 7.41 | 0.16 | (7.10, 7.72) | <0.001 |
|  | 9-15 years | 7.50 | 0.04 | (7.43, 7.58) |  |
|  | 16+years | 7.90 | 0.05 | (7.81, 7.99) |  |
| Immigration status | Born in this country | 7.63 | 0.03 | (7.57, 7.69) | 0.596 |
|  | Born in another country | 7.71 | 0.10 | (7.51, 7.92) |  |
| Religious affiliation | Christianity | 7.77 | 0.04 | (7.70, 7.84) | <0.001 |
|  | Islam | 7.60 | 0.17 | (7.26, 7.95) |  |
|  | Hinduism | 7.48 | 0.72 | (5.86, 9.09) |  |
|  | Buddhism | 7.94 | 0.32 | (7.29, 8.59) |  |
|  | Judaism | 7.26 | 0.59 | (6.01, 8.51) |  |
|  | Sikhism | 8.90 | 0.82 | (6.28, 11.53) |  |
|  | Baha’i | 9.00 |  |  |  |
|  | Confucianism | 7.41 | 0.34 | (5.91, 8.91) |  |
|  | Primal, Animist, or Folk religion | 7.63 | 0.74 | (6.12, 9.14) |  |
|  | Some other religion | 7.71 | 0.57 | (6.56, 8.85) |  |
|  | No religion/Atheist/Agnostic | 7.45 | 0.05 | (7.36, 7.54) |  |

Table S6a. Nationally representative descriptive statistics for Hong Kong

| **Characteristic** | **N = 3,012**^1^ |
| --- | --- |
| **Age group** |  |
| 18-24 | 217 (7.2%) |
| 25-29 | 198 (6.6%) |
| 30-39 | 507 (17%) |
| 40-49 | 580 (19%) |
| 50-59 | 711 (24%) |
| 60-69 | 620 (21%) |
| 70-79 | 164 (5.5%) |
| 80 or older | 15 (0.5%) |
| Missing | 0 (0%) |
| **Gender** |  |
| Male | 1,390 (46%) |
| Female | 1,620 (54%) |
| Other | 2 (<0.1%) |
| Missing | 0 (0%) |
| **Marital status** |  |
| Married | 2,080 (69%) |
| Separated | 21 (0.7%) |
| Divorced | 105 (3.5%) |
| Widowed | 45 (1.5%) |
| Never | 723 (24%) |
| Domestic Partner | 37 (1.2%) |
| Missing | 1 (<0.1%) |
| **Employment** |  |
| Employed for an employer | 2,056 (68%) |
| Self-employed | 245 (8.1%) |
| Retired | 423 (14%) |
| Student | 55 (1.8%) |
| Homemaker | 114 (3.8%) |
| Unemployed and looking for a job | 62 (2.0%) |
| None of these/other | 39 (1.3%) |
| Missing | 18 (0.6%) |
| **Religious service attendance** |  |
| >1/week | 237 (7.9%) |
| 1/week | 567 (19%) |
| 1-3/month | 332 (11%) |
| A few times a year | 543 (18%) |
| Never | 1,332 (44%) |
| Missing | 1 (<0.1%) |
| **Education** |  |
| up to 8 years | 433 (14%) |
| 9-15 years | 2,031 (67%) |
| 16+years | 547 (18%) |
| Missing | 0 (0%) |
| **Immigration** |  |
| Born in this country | 2,637 (88%) |
| Born in another country | 321 (11%) |
| Missing | 53 (1.8%) |
| **Religious affiliation** |  |
| Christianity | 757 (25%) |
| Islam | 86 (2.8%) |
| Hinduism | 20 (0.7%) |
| Buddhism | 349 (12%) |
| Judaism | 10 (0.3%) |
| Sikhism | 2 (<0.1%) |
| Baha’i | 3 (<0.1%) |
| Jainism | 1 (<0.1%) |
| Shinto | 19 (0.6%) |
| Taoism | 97 (3.2%) |
| Confucianism | 11 (0.4%) |
| Primal, Animist, or Folk religion | 27 (0.9%) |
| Chinese folk/traditional religion | 106 (3.5%) |
| Some other religion | 4 (0.1%) |
| No religion/Atheist/Agnostic | 1,518 (50%) |
| Missing | 5 (0.2%) |
| **Race and ethnicity** |  |
| Chinese (Cantonese) | 1,930 (64%) |
| Chinese (Chaoshan) | 201 (6.7%) |
| Chinese (Fujianese) | 117 (3.9%) |
| Chinese (Hakka) | 121 (4.0%) |
| Chinese (Other ethnicity) | 264 (8.8%) |
| Chinese (Shanghainese) | 89 (2.9%) |
| East Asian (Korean, Japanese) | 10 (0.3%) |
| Other | 4 (0.1%) |
| South Asian (Indian, Nepalese, Pakistani) | 17 (0.6%) |
| Southeast Asian (Filipino, Indonesian, Thailand) | 46 (1.5%) |
| Taiwanese | 14 (0.4%) |
| White | 15 (0.5%) |
| Missing | 184 (6.1%) |
| ^1^n (%) | |

Table S6b. Means by demographic category for Hong Kong

| Variable | Category | Mean | SE | 95% CI | Global p-value |
| --- | --- | --- | --- | --- | --- |
| Age group | 18-24 | 7.02 | 0.14 | (6.76, 7.29) | <0.001 |
|  | 25-29 | 6.66 | 0.24 | (6.18, 7.15) |  |
|  | 30-39 | 6.52 | 0.11 | (6.30, 6.74) |  |
|  | 40-49 | 7.06 | 0.11 | (6.84, 7.28) |  |
|  | 50-59 | 7.15 | 0.10 | (6.96, 7.34) |  |
|  | 60-69 | 7.07 | 0.14 | (6.79, 7.35) |  |
|  | 70-79 | 7.06 | 0.36 | (6.34, 7.78) |  |
|  | 80 or older | 7.41 | 0.51 | (6.30, 8.52) |  |
| Gender | Male | 6.92 | 0.08 | (6.76, 7.08) | 0.969 |
|  | Female | 7.01 | 0.07 | (6.86, 7.15) |  |
|  | Other | 6.91 |  |  |  |
| Marital status | Married | 7.31 | 0.06 | (7.18, 7.43) | <0.001 |
|  | Separated | 6.69 | 0.77 | (5.08, 8.30) |  |
|  | Divorced | 6.89 | 0.37 | (6.14, 7.63) |  |
|  | Widowed | 5.78 | 0.60 | (4.56, 6.99) |  |
|  | Never | 6.12 | 0.11 | (5.90, 6.34) |  |
|  | Domestic Partner | 6.15 | 0.35 | (5.43, 6.86) |  |
| Employment | Employed for an employer | 7.08 | 0.05 | (6.97, 7.18) | <0.001 |
|  | Self-employed | 7.40 | 0.23 | (6.95, 7.85) |  |
|  | Retired | 6.78 | 0.20 | (6.40, 7.17) |  |
|  | Student | 6.38 | 0.26 | (5.85, 6.91) |  |
|  | Homemaker | 6.28 | 0.29 | (5.71, 6.85) |  |
|  | Unemployed and looking for a job | 5.59 | 0.42 | (4.74, 6.43) |  |
|  | None of these/other | 5.47 | 1.01 | (3.43, 7.51) |  |
| Religious service attendance | >1/week | 8.99 | 0.11 | (8.77, 9.20) | <0.001 |
|  | 1/week | 7.69 | 0.12 | (7.44, 7.93) |  |
|  | 1-3/month | 7.49 | 0.11 | (7.28, 7.71) |  |
|  | A few times a year | 6.90 | 0.13 | (6.63, 7.16) |  |
|  | Never | 6.20 | 0.08 | (6.04, 6.36) |  |
| Education | up to 8 years | 7.25 | 0.20 | (6.86, 7.64) | 0.021 |
|  | 9-15 years | 6.95 | 0.06 | (6.83, 7.07) |  |
|  | 16+years | 6.80 | 0.11 | (6.59, 7.01) |  |
| Immigration status | Born in this country | 7.00 | 0.05 | (6.89, 7.10) | 0.193 |
|  | Born in another country | 6.73 | 0.23 | (6.27, 7.18) |  |
| Religious affiliation | Christianity | 7.38 | 0.11 | (7.18, 7.59) | <0.001 |
|  | Islam | 7.61 | 0.58 | (6.46, 8.75) |  |
|  | Hinduism | 7.63 | 0.41 | (6.76, 8.50) |  |
|  | Buddhism | 7.78 | 0.14 | (7.51, 8.06) |  |
|  | Judaism | 6.85 | 0.52 | (5.64, 8.06) |  |
|  | Sikhism | 8.45 |  |  |  |
|  | Baha’i | 8.19 |  |  |  |
|  | Shinto | 8.73 | 0.23 | (8.25, 9.21) |  |
|  | Taoism | 7.42 | 0.29 | (6.84, 8.00) |  |
|  | Confucianism | 7.46 | 0.25 | (6.89, 8.03) |  |
|  | Primal, Animist, or Folk religion | 6.93 | 0.28 | (6.35, 7.51) |  |
|  | Chinese folk/traditional religion | 7.64 | 0.21 | (7.22, 8.06) |  |
|  | Some other religion | 5.49 | 3.18 | (-11.73, 22.70) |  |
|  | No religion/Atheist/Agnostic | 6.43 | 0.07 | (6.28, 6.57) |  |
| Race and ethnicity | Other | 7.04 | 0.79 | (2.32, 11.76) | <0.001 |
|  | White | 7.20 | 0.49 | (6.13, 8.26) |  |
|  | Chinese (Cantonese) | 6.89 | 0.07 | (6.76, 7.02) |  |
|  | Chinese (Chaoshan) | 6.94 | 0.22 | (6.51, 7.36) |  |
|  | Chinese (Fujianese) | 7.10 | 0.24 | (6.63, 7.56) |  |
|  | Chinese (Hakka) | 6.82 | 0.27 | (6.27, 7.36) |  |
|  | Chinese (Other ethnicity) | 7.10 | 0.18 | (6.74, 7.46) |  |
|  | Chinese (Shanghainese) | 7.65 | 0.33 | (7.00, 8.31) |  |
|  | East Asian (Korean, Japanese) | 7.17 | 0.50 | (5.87, 8.48) |  |
|  | South Asian (Indian, Nepalese, Pakistani) | 7.77 | 0.76 | (6.14, 9.39) |  |
|  | Southeast Asian (Filipino, Indonesian, Thailand) | 8.46 | 0.52 | (7.40, 9.51) |  |
|  | Taiwanese | 5.83 | 0.76 | (4.18, 7.49) |  |

Table S7a. Nationally representative descriptive statistics for India

| **Characteristic** | **N = 12,765**^1^ |
| --- | --- |
| **Age group** |  |
| 18-24 | 2,543 (20%) |
| 25-29 | 1,640 (13%) |
| 30-39 | 3,109 (24%) |
| 40-49 | 2,275 (18%) |
| 50-59 | 1,574 (12%) |
| 60-69 | 1,188 (9.3%) |
| 70-79 | 370 (2.9%) |
| 80 or older | 67 (0.5%) |
| Missing | 0 (0%) |
| **Gender** |  |
| Male | 6,473 (51%) |
| Female | 6,292 (49%) |
| Other | 0 (0%) |
| Missing | 0 (0%) |
| **Marital status** |  |
| Married | 9,848 (77%) |
| Separated | 45 (0.4%) |
| Divorced | 25 (0.2%) |
| Widowed | 445 (3.5%) |
| Never | 2,065 (16%) |
| Domestic Partner | 269 (2.1%) |
| Missing | 69 (0.5%) |
| **Employment** |  |
| Employed for an employer | 2,660 (21%) |
| Self-employed | 3,401 (27%) |
| Retired | 286 (2.2%) |
| Student | 532 (4.2%) |
| Homemaker | 4,221 (33%) |
| Unemployed and looking for a job | 902 (7.1%) |
| None of these/other | 715 (5.6%) |
| Missing | 48 (0.4%) |
| **Religious service attendance** |  |
| >1/week | 2,875 (23%) |
| 1/week | 3,166 (25%) |
| 1-3/month | 2,740 (21%) |
| A few times a year | 2,090 (16%) |
| Never | 1,823 (14%) |
| Missing | 71 (0.6%) |
| **Education** |  |
| up to 8 years | 11,422 (89%) |
| 9-15 years | 1,194 (9.4%) |
| 16+years | 145 (1.1%) |
| Missing | 4 (<0.1%) |
| **Immigration** |  |
| Born in this country | 12,629 (99%) |
| Born in another country | 110 (0.9%) |
| Missing | 26 (0.2%) |
| **Religious affiliation** |  |
| Christianity | 306 (2.4%) |
| Islam | 1,555 (12%) |
| Hinduism | 10,362 (81%) |
| Buddhism | 230 (1.8%) |
| Sikhism | 127 (1.0%) |
| Jainism | 10 (<0.1%) |
| Shinto | 1 (<0.1%) |
| Primal, Animist, or Folk religion | 30 (0.2%) |
| Some other religion | 67 (0.5%) |
| No religion/Atheist/Agnostic | 13 (0.1%) |
| Missing | 62 (0.5%) |
| **Race and ethnicity** |  |
| General | 3,538 (28%) |
| Other backward caste | 4,177 (33%) |
| Schedule caste | 3,599 (28%) |
| Schedule tribe | 1,185 (9.3%) |
| Missing | 267 (2.1%) |
| ^1^n (%) | |

Table S7b. Means by demographic category for India

| Variable | Category | Mean | SE | 95% CI | Global p-value |
| --- | --- | --- | --- | --- | --- |
| Age group | 18-24 | 7.82 | 0.08 | (7.66, 7.99) | 0.017 |
|  | 25-29 | 7.81 | 0.10 | (7.62, 8.00) |  |
|  | 30-39 | 7.70 | 0.07 | (7.55, 7.84) |  |
|  | 40-49 | 7.77 | 0.08 | (7.62, 7.92) |  |
|  | 50-59 | 7.68 | 0.10 | (7.49, 7.87) |  |
|  | 60-69 | 7.37 | 0.14 | (7.11, 7.64) |  |
|  | 70-79 | 7.39 | 0.22 | (6.95, 7.83) |  |
|  | 80 or older | 7.33 | 0.60 | (6.14, 8.52) |  |
| Gender | Male | 7.62 | 0.05 | (7.52, 7.72) | <0.001 |
|  | Female | 7.80 | 0.06 | (7.68, 7.91) |  |
| Marital status | Married | 7.71 | 0.05 | (7.61, 7.81) | 0.652 |
|  | Separated | 6.77 | 0.66 | (5.43, 8.11) |  |
|  | Divorced | 6.89 | 0.96 | (4.89, 8.88) |  |
|  | Widowed | 7.48 | 0.21 | (7.06, 7.89) |  |
|  | Never | 7.75 | 0.09 | (7.58, 7.92) |  |
|  | Domestic Partner | 7.80 | 0.30 | (7.21, 8.39) |  |
| Employment | Employed for an employer | 7.71 | 0.09 | (7.54, 7.88) | <0.001 |
|  | Self-employed | 7.69 | 0.08 | (7.54, 7.85) |  |
|  | Retired | 7.50 | 0.31 | (6.90, 8.11) |  |
|  | Student | 8.10 | 0.14 | (7.83, 8.38) |  |
|  | Homemaker | 7.78 | 0.06 | (7.66, 7.91) |  |
|  | Unemployed and looking for a job | 7.34 | 0.15 | (7.04, 7.63) |  |
|  | None of these/other | 7.54 | 0.14 | (7.26, 7.82) |  |
| Religious service attendance | >1/week | 7.91 | 0.07 | (7.77, 8.04) | <0.001 |
|  | 1/week | 7.76 | 0.08 | (7.61, 7.91) |  |
|  | 1-3/month | 7.77 | 0.07 | (7.62, 7.92) |  |
|  | A few times a year | 7.41 | 0.09 | (7.24, 7.58) |  |
|  | Never | 7.54 | 0.10 | (7.34, 7.74) |  |
| Education | up to 8 years | 7.66 | 0.05 | (7.57, 7.76) | <0.001 |
|  | 9-15 years | 8.07 | 0.09 | (7.89, 8.24) |  |
|  | 16+years | 8.20 | 0.20 | (7.81, 8.60) |  |
| Immigration status | Born in this country | 7.71 | 0.05 | (7.62, 7.80) | 0.836 |
|  | Born in another country | 7.53 | 0.31 | (6.92, 8.14) |  |
| Religious affiliation | Christianity | 8.70 | 0.20 | (8.31, 9.10) | <0.001 |
|  | Islam | 7.67 | 0.12 | (7.43, 7.91) |  |
|  | Hinduism | 7.70 | 0.05 | (7.61, 7.80) |  |
|  | Buddhism | 7.07 | 0.23 | (6.62, 7.52) |  |
|  | Sikhism | 7.88 | 0.24 | (7.42, 8.35) |  |
|  | Jainism | 6.84 | 0.55 | (5.57, 8.11) |  |
|  | Primal, Animist, or Folk religion | 7.57 | 0.59 | (6.37, 8.77) |  |
|  | Some other religion | 6.78 | 0.42 | (5.95, 7.61) |  |
|  | No religion/Atheist/Agnostic | 7.53 | 1.04 | (5.24, 9.83) |  |
| Race and ethnicity | General | 7.81 | 0.07 | (7.66, 7.95) | 0.336 |
|  | Other backward caste | 7.69 | 0.07 | (7.55, 7.82) |  |
|  | Schedule caste | 7.63 | 0.08 | (7.47, 7.78) |  |
|  | Schedule tribe | 7.72 | 0.11 | (7.49, 7.94) |  |

Table S8a. Nationally representative descriptive statistics for Indonesia

| **Characteristic** | **N = 6,992**^1^ |
| --- | --- |
| **Age group** |  |
| 18-24 | 1,216 (17%) |
| 25-29 | 849 (12%) |
| 30-39 | 1,591 (23%) |
| 40-49 | 1,576 (23%) |
| 50-59 | 1,169 (17%) |
| 60-69 | 490 (7.0%) |
| 70-79 | 83 (1.2%) |
| 80 or older | 17 (0.2%) |
| Missing | 0 (0%) |
| **Gender** |  |
| Male | 3,461 (50%) |
| Female | 3,513 (50%) |
| Other | 7 (<0.1%) |
| Missing | 11 (0.2%) |
| **Marital status** |  |
| Married | 4,846 (69%) |
| Separated | 81 (1.2%) |
| Divorced | 196 (2.8%) |
| Widowed | 425 (6.1%) |
| Never | 1,381 (20%) |
| Domestic Partner | 18 (0.3%) |
| Missing | 45 (0.6%) |
| **Employment** |  |
| Employed for an employer | 1,323 (19%) |
| Self-employed | 2,187 (31%) |
| Retired | 78 (1.1%) |
| Student | 272 (3.9%) |
| Homemaker | 2,138 (31%) |
| Unemployed and looking for a job | 529 (7.6%) |
| None of these/other | 448 (6.4%) |
| Missing | 18 (0.3%) |
| **Religious service attendance** |  |
| >1/week | 2,667 (38%) |
| 1/week | 2,529 (36%) |
| 1-3/month | 786 (11%) |
| A few times a year | 659 (9.4%) |
| Never | 332 (4.8%) |
| Missing | 18 (0.3%) |
| **Education** |  |
| up to 8 years | 3,079 (44%) |
| 9-15 years | 3,491 (50%) |
| 16+years | 419 (6.0%) |
| Missing | 2 (<0.1%) |
| **Immigration** |  |
| Born in this country | 6,958 (100%) |
| Born in another country | 34 (0.5%) |
| Missing | 0 (0%) |
| **Religious affiliation** |  |
| Christianity | 504 (7.2%) |
| Islam | 6,406 (92%) |
| Hinduism | 73 (1.0%) |
| Buddhism | 3 (<0.1%) |
| Taoism | 1 (<0.1%) |
| Some other religion | 1 (<0.1%) |
| Missing | 4 (<0.1%) |
| **Race and ethnicity** |  |
| Bali | 69 (1.0%) |
| Banjar/Melayu Banjar | 320 (4.6%) |
| Batak | 165 (2.4%) |
| Betawi | 251 (3.6%) |
| Bugis | 243 (3.5%) |
| Jawa | 2,846 (41%) |
| Madura | 262 (3.7%) |
| Makasar | 91 (1.3%) |
| Minangkabau | 273 (3.9%) |
| Other | 1,262 (18%) |
| Sunda/Parahyangan | 1,172 (17%) |
| Missing | 38 (0.5%) |
| ^1^n (%) | |

Table S8b. Means by demographic category for Indonesia

| Variable | Category | Mean | SE | 95% CI | Global p-value |
| --- | --- | --- | --- | --- | --- |
| Age group | 18-24 | 8.96 | 0.07 | (8.82, 9.09) | <0.001 |
|  | 25-29 | 9.01 | 0.07 | (8.86, 9.15) |  |
|  | 30-39 | 9.06 | 0.05 | (8.96, 9.15) |  |
|  | 40-49 | 8.93 | 0.07 | (8.80, 9.06) |  |
|  | 50-59 | 8.79 | 0.08 | (8.65, 8.94) |  |
|  | 60-69 | 8.76 | 0.11 | (8.56, 8.97) |  |
|  | 70-79 | 8.36 | 0.38 | (7.60, 9.11) |  |
|  | 80 or older | 8.14 | 0.86 | (6.30, 9.99) |  |
| Gender | Male | 8.92 | 0.05 | (8.83, 9.01) | 0.015 |
|  | Female | 8.94 | 0.04 | (8.86, 9.03) |  |
|  | Other | 6.83 | 1.03 | (4.11, 9.55) |  |
| Marital status | Married | 8.94 | 0.04 | (8.86, 9.01) | <0.001 |
|  | Separated | 8.79 | 0.32 | (8.15, 9.43) |  |
|  | Divorced | 8.97 | 0.18 | (8.61, 9.32) |  |
|  | Widowed | 8.71 | 0.13 | (8.45, 8.97) |  |
|  | Never | 8.97 | 0.08 | (8.82, 9.12) |  |
|  | Domestic Partner | 9.70 | 0.16 | (9.36, 10.04) |  |
| Employment | Employed for an employer | 8.93 | 0.06 | (8.82, 9.05) | <0.001 |
|  | Self-employed | 8.99 | 0.06 | (8.88, 9.10) |  |
|  | Retired | 8.79 | 0.18 | (8.43, 9.15) |  |
|  | Student | 9.16 | 0.09 | (8.98, 9.34) |  |
|  | Homemaker | 8.89 | 0.06 | (8.76, 9.02) |  |
|  | Unemployed and looking for a job | 8.88 | 0.12 | (8.64, 9.12) |  |
|  | None of these/other | 8.74 | 0.12 | (8.51, 8.97) |  |
| Religious service attendance | >1/week | 9.01 | 0.05 | (8.92, 9.11) | 0.003 |
|  | 1/week | 8.92 | 0.05 | (8.82, 9.03) |  |
|  | 1-3/month | 8.79 | 0.09 | (8.61, 8.97) |  |
|  | A few times a year | 8.85 | 0.09 | (8.68, 9.02) |  |
|  | Never | 8.79 | 0.14 | (8.51, 9.07) |  |
| Education | up to 8 years | 8.86 | 0.07 | (8.73, 8.99) | 0.067 |
|  | 9-15 years | 8.98 | 0.03 | (8.92, 9.04) |  |
|  | 16+years | 9.01 | 0.07 | (8.88, 9.15) |  |
| Immigration status | Born in this country | 8.93 | 0.04 | (8.86, 9.00) | 0.659 |
|  | Born in another country | 8.68 | 0.35 | (7.96, 9.39) |  |
| Religious affiliation | Christianity | 9.08 | 0.16 | (8.76, 9.39) | 0.015 |
|  | Islam | 8.92 | 0.04 | (8.85, 8.99) |  |
|  | Hinduism | 8.67 | 0.25 | (8.16, 9.17) |  |
|  | Buddhism | 6.25 | 1.32 | (-5.88, 18.38) |  |
| Race and ethnicity | Other | 9.11 | 0.08 | (8.95, 9.26) | <0.001 |
|  | Bali | 8.55 | 0.25 | (8.04, 9.05) |  |
|  | Banjar/Melayu Banjar | 8.91 | 0.15 | (8.62, 9.21) |  |
|  | Batak | 9.07 | 0.07 | (8.93, 9.22) |  |
|  | Betawi | 9.06 | 0.12 | (8.81, 9.30) |  |
|  | Bugis | 8.98 | 0.10 | (8.77, 9.18) |  |
|  | Jawa | 8.93 | 0.04 | (8.84, 9.02) |  |
|  | Madura | 8.57 | 0.39 | (7.81, 9.33) |  |
|  | Makasar | 9.11 | 0.16 | (8.79, 9.43) |  |
|  | Minangkabau | 8.52 | 0.13 | (8.27, 8.77) |  |
|  | Sunda/Parahyangan | 8.87 | 0.09 | (8.69, 9.05) |  |

Table S9a. Nationally representative descriptive statistics for Israel

| **Characteristic** | **N = 3,669**^1^ |
| --- | --- |
| **Age group** |  |
| 18-24 | 553 (15%) |
| 25-29 | 407 (11%) |
| 30-39 | 666 (18%) |
| 40-49 | 616 (17%) |
| 50-59 | 542 (15%) |
| 60-69 | 469 (13%) |
| 70-79 | 336 (9.2%) |
| 80 or older | 79 (2.2%) |
| Missing | 0 (0%) |
| **Gender** |  |
| Male | 1,791 (49%) |
| Female | 1,872 (51%) |
| Other | 0 (<0.1%) |
| Missing | 6 (0.2%) |
| **Marital status** |  |
| Married | 2,056 (56%) |
| Separated | 48 (1.3%) |
| Divorced | 258 (7.0%) |
| Widowed | 212 (5.8%) |
| Never | 834 (23%) |
| Domestic Partner | 193 (5.3%) |
| Missing | 69 (1.9%) |
| **Employment** |  |
| Employed for an employer | 1,793 (49%) |
| Self-employed | 424 (12%) |
| Retired | 576 (16%) |
| Student | 388 (11%) |
| Homemaker | 211 (5.7%) |
| Unemployed and looking for a job | 148 (4.0%) |
| None of these/other | 118 (3.2%) |
| Missing | 10 (0.3%) |
| **Religious service attendance** |  |
| >1/week | 649 (18%) |
| 1/week | 495 (14%) |
| 1-3/month | 374 (10%) |
| A few times a year | 1,014 (28%) |
| Never | 1,122 (31%) |
| Missing | 14 (0.4%) |
| **Education** |  |
| up to 8 years | 224 (6.1%) |
| 9-15 years | 1,517 (41%) |
| 16+years | 1,926 (52%) |
| Missing | 2 (<0.1%) |
| **Immigration** |  |
| Born in this country | 2,796 (76%) |
| Born in another country | 868 (24%) |
| Missing | 5 (0.1%) |
| **Religious affiliation** |  |
| Christianity | 39 (1.1%) |
| Islam | 656 (18%) |
| Judaism | 2,897 (79%) |
| Baha’i | 2 (<0.1%) |
| Taoism | 1 (<0.1%) |
| Primal, Animist, or Folk religion | 1 (<0.1%) |
| Some other religion | 5 (0.1%) |
| No religion/Atheist/Agnostic | 64 (1.7%) |
| Missing | 4 (0.1%) |
| **Race and ethnicity** |  |
| Arab | 674 (18%) |
| Jewish | 2,926 (80%) |
| Other | 39 (1.1%) |
| Missing | 30 (0.8%) |
| ^1^n (%) | |

Table S9b. Means by demographic category for Israel

| Variable | Category | Mean | SE | 95% CI | Global p-value |
| --- | --- | --- | --- | --- | --- |
| Age group | 18-24 | 8.28 | 0.12 | (8.05, 8.51) | <0.001 |
|  | 25-29 | 8.12 | 0.15 | (7.83, 8.42) |  |
|  | 30-39 | 7.87 | 0.13 | (7.61, 8.12) |  |
|  | 40-49 | 7.87 | 0.14 | (7.60, 8.14) |  |
|  | 50-59 | 7.87 | 0.13 | (7.60, 8.13) |  |
|  | 60-69 | 7.78 | 0.18 | (7.42, 8.13) |  |
|  | 70-79 | 7.49 | 0.19 | (7.12, 7.87) |  |
|  | 80 or older | 7.52 | 0.32 | (6.88, 8.17) |  |
| Gender | Male | 7.89 | 0.10 | (7.69, 8.10) | 0.995 |
|  | Female | 7.92 | 0.12 | (7.69, 8.14) |  |
| Marital status | Married | 8.17 | 0.09 | (7.98, 8.35) | <0.001 |
|  | Separated | 7.24 | 0.23 | (6.77, 7.71) |  |
|  | Divorced | 6.88 | 0.27 | (6.35, 7.41) |  |
|  | Widowed | 7.27 | 0.24 | (6.79, 7.75) |  |
|  | Never | 7.88 | 0.13 | (7.64, 8.13) |  |
|  | Domestic Partner | 7.47 | 0.27 | (6.94, 8.00) |  |
| Employment | Employed for an employer | 8.00 | 0.11 | (7.78, 8.22) | <0.001 |
|  | Self-employed | 7.83 | 0.14 | (7.56, 8.11) |  |
|  | Retired | 7.66 | 0.19 | (7.30, 8.03) |  |
|  | Student | 8.41 | 0.14 | (8.14, 8.67) |  |
|  | Homemaker | 7.61 | 0.22 | (7.18, 8.04) |  |
|  | Unemployed and looking for a job | 6.99 | 0.24 | (6.51, 7.47) |  |
|  | None of these/other | 7.95 | 0.17 | (7.63, 8.28) |  |
| Religious service attendance | >1/week | 9.12 | 0.08 | (8.97, 9.27) | <0.001 |
|  | 1/week | 8.26 | 0.16 | (7.94, 8.57) |  |
|  | 1-3/month | 7.75 | 0.16 | (7.43, 8.07) |  |
|  | A few times a year | 7.66 | 0.18 | (7.30, 8.02) |  |
|  | Never | 7.32 | 0.12 | (7.09, 7.55) |  |
| Education | up to 8 years | 7.33 | 0.27 | (6.81, 7.86) | <0.001 |
|  | 9-15 years | 7.88 | 0.11 | (7.67, 8.08) |  |
|  | 16+years | 7.99 | 0.11 | (7.78, 8.21) |  |
| Immigration status | Born in this country | 8.10 | 0.08 | (7.93, 8.26) | <0.001 |
|  | Born in another country | 7.29 | 0.19 | (6.93, 7.66) |  |
| Religious affiliation | Christianity | 6.58 | 0.51 | (5.55, 7.61) | <0.001 |
|  | Islam | 7.30 | 0.18 | (6.95, 7.64) |  |
|  | Judaism | 8.08 | 0.11 | (7.87, 8.30) |  |
|  | Baha’i | 10.00 |  |  |  |
|  | Some other religion | 8.35 | 0.79 | (5.79, 10.90) |  |
|  | No religion/Atheist/Agnostic | 6.85 | 0.33 | (6.19, 7.52) |  |
| Race and ethnicity | Other | 7.34 | 0.33 | (6.68, 8.01) | <0.001 |
|  | Arab | 7.29 | 0.17 | (6.95, 7.62) |  |
|  | Jewish | 8.06 | 0.12 | (7.83, 8.29) |  |

Table S10a. Nationally representative descriptive statistics for Japan

| **Characteristic** | **N = 20,543**^1^ |
| --- | --- |
| **Age group** |  |
| 18-24 | 1,589 (7.7%) |
| 25-29 | 806 (3.9%) |
| 30-39 | 2,851 (14%) |
| 40-49 | 3,363 (16%) |
| 50-59 | 3,770 (18%) |
| 60-69 | 4,118 (20%) |
| 70-79 | 3,554 (17%) |
| 80 or older | 493 (2.4%) |
| Missing | 0 (0%) |
| **Gender** |  |
| Male | 9,847 (48%) |
| Female | 10,602 (52%) |
| Other | 28 (0.1%) |
| Missing | 66 (0.3%) |
| **Marital status** |  |
| Married | 11,837 (58%) |
| Separated | 190 (0.9%) |
| Divorced | 2,126 (10%) |
| Widowed | 1,179 (5.7%) |
| Never | 5,004 (24%) |
| Domestic Partner | 144 (0.7%) |
| Missing | 64 (0.3%) |
| **Employment** |  |
| Employed for an employer | 10,853 (53%) |
| Self-employed | 1,748 (8.5%) |
| Retired | 2,535 (12%) |
| Student | 491 (2.4%) |
| Homemaker | 1,276 (6.2%) |
| Unemployed and looking for a job | 622 (3.0%) |
| None of these/other | 2,983 (15%) |
| Missing | 36 (0.2%) |
| **Religious service attendance** |  |
| >1/week | 316 (1.5%) |
| 1/week | 348 (1.7%) |
| 1-3/month | 862 (4.2%) |
| A few times a year | 3,112 (15%) |
| Never | 15,788 (77%) |
| Missing | 117 (0.6%) |
| **Education** |  |
| up to 8 years | 567 (2.8%) |
| 9-15 years | 14,893 (72%) |
| 16+years | 5,083 (25%) |
| Missing | 0 (0%) |
| **Immigration** |  |
| Born in this country | 19,548 (95%) |
| Born in another country | 158 (0.8%) |
| Missing | 837 (4.1%) |
| **Religious affiliation** |  |
| Christianity | 381 (1.9%) |
| Islam | 10 (<0.1%) |
| Hinduism | 5 (<0.1%) |
| Buddhism | 6,709 (33%) |
| Judaism | 10 (<0.1%) |
| Sikhism | 6 (<0.1%) |
| Baha’i | 2 (<0.1%) |
| Jainism | 11 (<0.1%) |
| Shinto | 469 (2.3%) |
| Taoism | 7 (<0.1%) |
| Confucianism | 17 (<0.1%) |
| Primal, Animist, or Folk religion | 19 (<0.1%) |
| Some other religion | 46 (0.2%) |
| No religion/Atheist/Agnostic | 12,497 (61%) |
| Missing | 355 (1.7%) |
| **Race and ethnicity** |  |
| Missing | 20,543 (100%) |
| ^1^n (%) | |

Table S10b. Means by demographic category for Japan

| Variable | Category | Mean | SE | 95% CI | Global p-value |
| --- | --- | --- | --- | --- | --- |
| Age group | 18-24 | 5.86 | 0.07 | (5.73, 6.00) | <0.001 |
|  | 25-29 | 5.50 | 0.09 | (5.31, 5.68) |  |
|  | 30-39 | 5.46 | 0.05 | (5.36, 5.56) |  |
|  | 40-49 | 5.45 | 0.05 | (5.35, 5.55) |  |
|  | 50-59 | 5.56 | 0.04 | (5.48, 5.64) |  |
|  | 60-69 | 5.96 | 0.04 | (5.88, 6.03) |  |
|  | 70-79 | 6.44 | 0.04 | (6.36, 6.51) |  |
|  | 80 or older | 6.82 | 0.11 | (6.61, 7.03) |  |
| Gender | Male | 5.55 | 0.03 | (5.50, 5.60) | <0.001 |
|  | Female | 6.05 | 0.03 | (6.00, 6.10) |  |
|  | Other | 5.79 | 0.43 | (4.91, 6.67) |  |
| Marital status | Married | 6.02 | 0.02 | (5.97, 6.06) | <0.001 |
|  | Separated | 5.85 | 0.19 | (5.47, 6.24) |  |
|  | Divorced | 5.67 | 0.07 | (5.54, 5.80) |  |
|  | Widowed | 6.65 | 0.08 | (6.49, 6.82) |  |
|  | Never | 5.18 | 0.03 | (5.12, 5.25) |  |
|  | Domestic Partner | 5.57 | 0.24 | (5.09, 6.06) |  |
| Employment | Employed for an employer | 5.65 | 0.03 | (5.60, 5.70) | <0.001 |
|  | Self-employed | 6.05 | 0.07 | (5.92, 6.18) |  |
|  | Retired | 6.10 | 0.04 | (6.02, 6.19) |  |
|  | Student | 6.27 | 0.11 | (6.07, 6.48) |  |
|  | Homemaker | 6.16 | 0.06 | (6.05, 6.27) |  |
|  | Unemployed and looking for a job | 4.64 | 0.10 | (4.45, 4.82) |  |
|  | None of these/other | 6.02 | 0.04 | (5.94, 6.10) |  |
| Religious service attendance | >1/week | 7.45 | 0.14 | (7.17, 7.73) | <0.001 |
|  | 1/week | 7.03 | 0.13 | (6.77, 7.29) |  |
|  | 1-3/month | 6.12 | 0.08 | (5.96, 6.29) |  |
|  | A few times a year | 6.12 | 0.04 | (6.03, 6.20) |  |
|  | Never | 5.67 | 0.02 | (5.63, 5.71) |  |
| Education | up to 8 years | 5.11 | 0.12 | (4.87, 5.35) | <0.001 |
|  | 9-15 years | 5.67 | 0.02 | (5.63, 5.71) |  |
|  | 16+years | 6.29 | 0.04 | (6.22, 6.36) |  |
| Immigration status | Born in this country | 5.81 | 0.02 | (5.77, 5.84) | 0.061 |
|  | Born in another country | 6.05 | 0.19 | (5.68, 6.43) |  |
| Religious affiliation | Christianity | 7.00 | 0.14 | (6.74, 7.27) | <0.001 |
|  | Islam | 6.09 | 0.87 | (4.04, 8.14) |  |
|  | Hinduism | 6.10 | 0.83 | (3.45, 8.75) |  |
|  | Buddhism | 6.16 | 0.03 | (6.10, 6.22) |  |
|  | Judaism | 5.53 | 1.05 | (3.06, 8.01) |  |
|  | Sikhism | 5.97 | 0.39 | (4.84, 7.09) |  |
|  | Baha’i | 6.62 |  |  |  |
|  | Jainism | 4.77 | 1.32 | (1.78, 7.77) |  |
|  | Shinto | 6.17 | 0.14 | (5.90, 6.44) |  |
|  | Taoism | 6.95 | 0.70 | (5.16, 8.75) |  |
|  | Confucianism | 6.15 | 0.51 | (5.05, 7.25) |  |
|  | Primal, Animist, or Folk religion | 6.87 | 0.55 | (5.71, 8.03) |  |
|  | Some other religion | 5.29 | 0.55 | (4.18, 6.39) |  |
|  | No religion/Atheist/Agnostic | 5.57 | 0.02 | (5.53, 5.62) |  |

Table S11a. Nationally representative descriptive statistics for Kenya

| **Characteristic** | **N = 11,389**^1^ |
| --- | --- |
| **Age group** |  |
| 18-24 | 2,868 (25%) |
| 25-29 | 2,035 (18%) |
| 30-39 | 2,564 (23%) |
| 40-49 | 1,708 (15%) |
| 50-59 | 1,072 (9.4%) |
| 60-69 | 710 (6.2%) |
| 70-79 | 360 (3.2%) |
| 80 or older | 67 (0.6%) |
| Missing | 5 (<0.1%) |
| **Gender** |  |
| Male | 5,567 (49%) |
| Female | 5,813 (51%) |
| Other | 2 (<0.1%) |
| Missing | 7 (<0.1%) |
| **Marital status** |  |
| Married | 6,626 (58%) |
| Separated | 467 (4.1%) |
| Divorced | 111 (1.0%) |
| Widowed | 464 (4.1%) |
| Never | 3,531 (31%) |
| Domestic Partner | 146 (1.3%) |
| Missing | 43 (0.4%) |
| **Employment** |  |
| Employed for an employer | 1,467 (13%) |
| Self-employed | 3,630 (32%) |
| Retired | 319 (2.8%) |
| Student | 1,136 (10.0%) |
| Homemaker | 1,537 (13%) |
| Unemployed and looking for a job | 3,153 (28%) |
| None of these/other | 138 (1.2%) |
| Missing | 9 (<0.1%) |
| **Religious service attendance** |  |
| >1/week | 2,774 (24%) |
| 1/week | 6,063 (53%) |
| 1-3/month | 1,219 (11%) |
| A few times a year | 855 (7.5%) |
| Never | 465 (4.1%) |
| Missing | 13 (0.1%) |
| **Education** |  |
| up to 8 years | 4,485 (39%) |
| 9-15 years | 6,115 (54%) |
| 16+years | 783 (6.9%) |
| Missing | 6 (<0.1%) |
| **Immigration** |  |
| Born in this country | 11,270 (99%) |
| Born in another country | 117 (1.0%) |
| Missing | 2 (<0.1%) |
| **Religious affiliation** |  |
| Christianity | 10,334 (91%) |
| Islam | 918 (8.1%) |
| Buddhism | 1 (<0.1%) |
| Judaism | 3 (<0.1%) |
| Baha’i | 1 (<0.1%) |
| Jainism | 1 (<0.1%) |
| Confucianism | 3 (<0.1%) |
| Primal, Animist, or Folk religion | 7 (<0.1%) |
| Some other religion | 5 (<0.1%) |
| No religion/Atheist/Agnostic | 108 (0.9%) |
| Missing | 9 (<0.1%) |
| **Race and ethnicity** |  |
| Embu | 197 (1.7%) |
| Kalenjin | 1,377 (12%) |
| Kamba | 1,299 (11%) |
| Kenyan Somali/Somali | 396 (3.5%) |
| Kikuyu | 2,119 (19%) |
| Kisii | 789 (6.9%) |
| Luhya | 1,943 (17%) |
| Luo | 1,120 (9.8%) |
| Maasai | 237 (2.1%) |
| Meru | 630 (5.5%) |
| Miji Kenda tribes | 708 (6.2%) |
| Other | 548 (4.8%) |
| Missing | 27 (0.2%) |
| ^1^n (%) | |

Table S11b. Means by demographic category for Kenya

| Variable | Category | Mean | SE | 95% CI | Global p-value |
| --- | --- | --- | --- | --- | --- |
| Age group | 18-24 | 8.07 | 0.06 | (7.95, 8.19) | 0.005 |
|  | 25-29 | 8.18 | 0.07 | (8.06, 8.31) |  |
|  | 30-39 | 8.07 | 0.07 | (7.94, 8.20) |  |
|  | 40-49 | 7.85 | 0.09 | (7.66, 8.04) |  |
|  | 50-59 | 8.08 | 0.12 | (7.84, 8.32) |  |
|  | 60-69 | 8.37 | 0.15 | (8.08, 8.67) |  |
|  | 70-79 | 8.07 | 0.25 | (7.59, 8.55) |  |
|  | 80 or older | 8.18 | 0.56 | (7.07, 9.29) |  |
| Gender | Male | 8.04 | 0.05 | (7.94, 8.15) | <0.001 |
|  | Female | 8.11 | 0.05 | (8.00, 8.21) |  |
|  | Other | 10.00 |  |  |  |
| Marital status | Married | 8.07 | 0.05 | (7.97, 8.17) | 0.357 |
|  | Separated | 7.81 | 0.16 | (7.50, 8.13) |  |
|  | Divorced | 7.82 | 0.30 | (7.22, 8.43) |  |
|  | Widowed | 7.97 | 0.21 | (7.56, 8.38) |  |
|  | Never | 8.15 | 0.06 | (8.04, 8.26) |  |
|  | Domestic Partner | 7.92 | 0.29 | (7.34, 8.49) |  |
| Employment | Employed for an employer | 8.13 | 0.09 | (7.96, 8.31) | <0.001 |
|  | Self-employed | 7.97 | 0.07 | (7.84, 8.10) |  |
|  | Retired | 7.99 | 0.23 | (7.55, 8.44) |  |
|  | Student | 8.28 | 0.08 | (8.12, 8.44) |  |
|  | Homemaker | 8.20 | 0.10 | (8.00, 8.39) |  |
|  | Unemployed and looking for a job | 8.06 | 0.06 | (7.93, 8.18) |  |
|  | None of these/other | 7.92 | 0.32 | (7.29, 8.55) |  |
| Religious service attendance | >1/week | 8.20 | 0.07 | (8.06, 8.33) | <0.001 |
|  | 1/week | 8.17 | 0.05 | (8.08, 8.27) |  |
|  | 1-3/month | 7.79 | 0.11 | (7.57, 8.01) |  |
|  | A few times a year | 7.63 | 0.13 | (7.39, 7.88) |  |
|  | Never | 7.66 | 0.17 | (7.33, 7.99) |  |
| Education | up to 8 years | 8.06 | 0.07 | (7.93, 8.19) | 0.544 |
|  | 9-15 years | 8.07 | 0.05 | (7.98, 8.16) |  |
|  | 16+years | 8.23 | 0.12 | (7.99, 8.47) |  |
| Immigration status | Born in this country | 8.08 | 0.04 | (7.99, 8.16) | 0.996 |
|  | Born in another country | 8.14 | 0.28 | (7.58, 8.70) |  |
| Religious affiliation | Christianity | 8.12 | 0.04 | (8.03, 8.20) | 0.001 |
|  | Islam | 7.69 | 0.15 | (7.39, 7.99) |  |
|  | Judaism | 8.34 |  |  |  |
|  | Primal, Animist, or Folk religion | 6.89 | 0.82 | (4.76, 9.01) |  |
|  | Some other religion | 8.20 | 0.88 | (5.46, 10.93) |  |
|  | No religion/Atheist/Agnostic | 7.74 | 0.33 | (7.09, 8.40) |  |
| Race and ethnicity | Other | 8.19 | 0.15 | (7.91, 8.48) | <0.001 |
|  | Embu | 8.42 | 0.19 | (8.04, 8.79) |  |
|  | Kalenjin | 8.32 | 0.10 | (8.12, 8.53) |  |
|  | Kamba | 8.34 | 0.09 | (8.15, 8.52) |  |
|  | Kenyan Somali/Somali | 7.43 | 0.25 | (6.94, 7.93) |  |
|  | Kikuyu | 8.07 | 0.10 | (7.87, 8.27) |  |
|  | Kisii | 7.22 | 0.13 | (6.97, 7.47) |  |
|  | Luhya | 8.24 | 0.07 | (8.10, 8.38) |  |
|  | Luo | 7.65 | 0.13 | (7.40, 7.91) |  |
|  | Maasai | 8.64 | 0.22 | (8.21, 9.06) |  |
|  | Meru | 8.10 | 0.14 | (7.83, 8.37) |  |
|  | Miji Kenda tribes | 8.29 | 0.18 | (7.94, 8.64) |  |

Table S12a. Nationally representative descriptive statistics for Mexico

| **Characteristic** | **N = 5,776**^1^ |
| --- | --- |
| **Age group** |  |
| 18-24 | 986 (17%) |
| 25-29 | 623 (11%) |
| 30-39 | 1,312 (23%) |
| 40-49 | 1,027 (18%) |
| 50-59 | 873 (15%) |
| 60-69 | 611 (11%) |
| 70-79 | 277 (4.8%) |
| 80 or older | 68 (1.2%) |
| Missing | 0 (0%) |
| **Gender** |  |
| Male | 2,755 (48%) |
| Female | 2,997 (52%) |
| Other | 3 (<0.1%) |
| Missing | 21 (0.4%) |
| **Marital status** |  |
| Married | 2,089 (36%) |
| Separated | 403 (7.0%) |
| Divorced | 230 (4.0%) |
| Widowed | 347 (6.0%) |
| Never | 1,432 (25%) |
| Domestic Partner | 1,109 (19%) |
| Missing | 166 (2.9%) |
| **Employment** |  |
| Employed for an employer | 1,921 (33%) |
| Self-employed | 1,091 (19%) |
| Retired | 386 (6.7%) |
| Student | 247 (4.3%) |
| Homemaker | 1,257 (22%) |
| Unemployed and looking for a job | 564 (9.8%) |
| None of these/other | 169 (2.9%) |
| Missing | 141 (2.4%) |
| **Religious service attendance** |  |
| >1/week | 609 (11%) |
| 1/week | 1,261 (22%) |
| 1-3/month | 676 (12%) |
| A few times a year | 2,054 (36%) |
| Never | 1,134 (20%) |
| Missing | 43 (0.7%) |
| **Education** |  |
| up to 8 years | 1,291 (22%) |
| 9-15 years | 3,180 (55%) |
| 16+years | 1,304 (23%) |
| Missing | 1 (<0.1%) |
| **Immigration** |  |
| Born in this country | 5,517 (96%) |
| Born in another country | 108 (1.9%) |
| Missing | 151 (2.6%) |
| **Religious affiliation** |  |
| Christianity | 4,844 (84%) |
| Islam | 2 (<0.1%) |
| Hinduism | 3 (<0.1%) |
| Buddhism | 6 (0.1%) |
| Judaism | 7 (0.1%) |
| Baha’i | 1 (<0.1%) |
| Jainism | 1 (<0.1%) |
| Shinto | 2 (<0.1%) |
| Taoism | 4 (<0.1%) |
| Confucianism | 1 (<0.1%) |
| Primal, Animist, or Folk religion | 20 (0.3%) |
| Some other religion | 41 (0.7%) |
| No religion/Atheist/Agnostic | 770 (13%) |
| Missing | 75 (1.3%) |
| **Race and ethnicity** |  |
| Black | 108 (1.9%) |
| Indigenous | 594 (10%) |
| Mestizo | 2,762 (48%) |
| Mulatto | 63 (1.1%) |
| Other | 339 (5.9%) |
| White | 1,116 (19%) |
| Missing | 794 (14%) |
| ^1^n (%) | |

Table S12b. Means by demographic category for Mexico

| Variable | Category | Mean | SE | 95% CI | Global p-value |
| --- | --- | --- | --- | --- | --- |
| Age group | 18-24 | 8.51 | 0.07 | (8.38, 8.65) | <0.001 |
|  | 25-29 | 8.70 | 0.09 | (8.53, 8.88) |  |
|  | 30-39 | 8.77 | 0.07 | (8.63, 8.90) |  |
|  | 40-49 | 8.97 | 0.07 | (8.84, 9.09) |  |
|  | 50-59 | 9.15 | 0.06 | (9.03, 9.27) |  |
|  | 60-69 | 8.93 | 0.10 | (8.73, 9.13) |  |
|  | 70-79 | 9.07 | 0.13 | (8.81, 9.32) |  |
|  | 80 or older | 8.69 | 0.22 | (8.24, 9.14) |  |
| Gender | Male | 8.69 | 0.05 | (8.60, 8.79) | <0.001 |
|  | Female | 8.98 | 0.04 | (8.91, 9.04) |  |
|  | Other | 7.91 | 0.58 | (4.65, 11.18) |  |
| Marital status | Married | 9.05 | 0.04 | (8.97, 9.13) | <0.001 |
|  | Separated | 8.79 | 0.10 | (8.58, 8.99) |  |
|  | Divorced | 9.18 | 0.12 | (8.94, 9.42) |  |
|  | Widowed | 8.83 | 0.14 | (8.54, 9.11) |  |
|  | Never | 8.52 | 0.06 | (8.39, 8.64) |  |
|  | Domestic Partner | 8.82 | 0.07 | (8.68, 8.96) |  |
| Employment | Employed for an employer | 8.75 | 0.06 | (8.63, 8.86) | <0.001 |
|  | Self-employed | 8.97 | 0.06 | (8.85, 9.09) |  |
|  | Retired | 8.98 | 0.10 | (8.78, 9.18) |  |
|  | Student | 8.19 | 0.15 | (7.90, 8.48) |  |
|  | Homemaker | 9.03 | 0.06 | (8.91, 9.14) |  |
|  | Unemployed and looking for a job | 8.65 | 0.09 | (8.47, 8.83) |  |
|  | None of these/other | 8.94 | 0.14 | (8.67, 9.21) |  |
| Religious service attendance | >1/week | 9.17 | 0.10 | (8.99, 9.36) | <0.001 |
|  | 1/week | 9.00 | 0.06 | (8.89, 9.11) |  |
|  | 1-3/month | 8.88 | 0.08 | (8.73, 9.03) |  |
|  | A few times a year | 8.85 | 0.05 | (8.76, 8.94) |  |
|  | Never | 8.45 | 0.08 | (8.29, 8.60) |  |
| Education | up to 8 years | 8.90 | 0.07 | (8.77, 9.03) | 0.802 |
|  | 9-15 years | 8.81 | 0.04 | (8.74, 8.89) |  |
|  | 16+years | 8.85 | 0.06 | (8.73, 8.97) |  |
| Immigration status | Born in this country | 8.85 | 0.03 | (8.79, 8.91) | <0.001 |
|  | Born in another country | 8.28 | 0.23 | (7.81, 8.74) |  |
| Religious affiliation | Christianity | 8.92 | 0.03 | (8.86, 8.98) | <0.001 |
|  | Islam | 10.00 |  |  |  |
|  | Hinduism | 8.17 | 1.19 | (-5.72, 22.06) |  |
|  | Buddhism | 8.84 | 1.52 | (1.48, 16.19) |  |
|  | Judaism | 9.49 | 0.32 | (8.62, 10.36) |  |
|  | Shinto | 7.33 |  |  |  |
|  | Taoism | 7.12 | 0.16 | (6.51, 7.74) |  |
|  | Confucianism | 8.00 |  |  |  |
|  | Primal, Animist, or Folk religion | 8.13 | 0.77 | (6.50, 9.75) |  |
|  | Some other religion | 8.93 | 0.28 | (8.35, 9.50) |  |
|  | No religion/Atheist/Agnostic | 8.36 | 0.09 | (8.18, 8.55) |  |
| Race and ethnicity | Black | 8.39 | 0.26 | (7.88, 8.90) | <0.001 |
|  | Indigenous | 8.83 | 0.10 | (8.62, 9.04) |  |
|  | Other | 9.12 | 0.09 | (8.94, 9.31) |  |
|  | White | 8.93 | 0.06 | (8.82, 9.05) |  |
|  | Mestizo | 8.80 | 0.04 | (8.72, 8.88) |  |
|  | Mulatto | 8.50 | 0.34 | (7.81, 9.19) |  |

Table S13a. Nationally representative descriptive statistics for Nigeria

| **Characteristic** | **N = 6,827**^1^ |
| --- | --- |
| **Age group** |  |
| 18-24 | 1,533 (22%) |
| 25-29 | 1,193 (17%) |
| 30-39 | 1,943 (28%) |
| 40-49 | 1,059 (16%) |
| 50-59 | 619 (9.1%) |
| 60-69 | 296 (4.3%) |
| 70-79 | 133 (2.0%) |
| 80 or older | 50 (0.7%) |
| Missing | 0 (0%) |
| **Gender** |  |
| Male | 3,371 (49%) |
| Female | 3,456 (51%) |
| Other | 0 (<0.1%) |
| Missing | 0 (0%) |
| **Marital status** |  |
| Married | 4,065 (60%) |
| Separated | 117 (1.7%) |
| Divorced | 71 (1.0%) |
| Widowed | 231 (3.4%) |
| Never | 2,289 (34%) |
| Domestic Partner | 12 (0.2%) |
| Missing | 42 (0.6%) |
| **Employment** |  |
| Employed for an employer | 699 (10%) |
| Self-employed | 3,898 (57%) |
| Retired | 178 (2.6%) |
| Student | 650 (9.5%) |
| Homemaker | 499 (7.3%) |
| Unemployed and looking for a job | 684 (10%) |
| None of these/other | 211 (3.1%) |
| Missing | 8 (0.1%) |
| **Religious service attendance** |  |
| >1/week | 4,049 (59%) |
| 1/week | 1,895 (28%) |
| 1-3/month | 531 (7.8%) |
| A few times a year | 254 (3.7%) |
| Never | 77 (1.1%) |
| Missing | 20 (0.3%) |
| **Education** |  |
| up to 8 years | 2,575 (38%) |
| 9-15 years | 4,120 (60%) |
| 16+years | 130 (1.9%) |
| Missing | 2 (<0.1%) |
| **Immigration** |  |
| Born in this country | 6,779 (99%) |
| Born in another country | 47 (0.7%) |
| Missing | 1 (<0.1%) |
| **Religious affiliation** |  |
| Christianity | 3,476 (51%) |
| Islam | 3,302 (48%) |
| Shinto | 1 (<0.1%) |
| Confucianism | 0 (<0.1%) |
| Primal, Animist, or Folk religion | 24 (0.3%) |
| Some other religion | 1 (<0.1%) |
| No religion/Atheist/Agnostic | 15 (0.2%) |
| Missing | 9 (0.1%) |
| **Race and ethnicity** |  |
| Edo | 116 (1.7%) |
| Efik | 48 (0.7%) |
| Fulani | 266 (3.9%) |
| Hausa | 2,342 (34%) |
| Ibibio | 180 (2.6%) |
| Idoma | 61 (0.9%) |
| Igala | 77 (1.1%) |
| Igbo (Ibo) | 1,111 (16%) |
| Ijaw | 110 (1.6%) |
| Kanuri | 31 (0.5%) |
| Other | 1,014 (15%) |
| Tiv | 198 (2.9%) |
| Urhobo | 38 (0.6%) |
| Yoruba | 1,230 (18%) |
| Missing | 4 (<0.1%) |
| ^1^n (%) | |

Table S13b. Means by demographic category for Nigeria

| Variable | Category | Mean | SE | 95% CI | Global p-value |
| --- | --- | --- | --- | --- | --- |
| Age group | 18-24 | 8.34 | 0.08 | (8.19, 8.49) | 0.006 |
|  | 25-29 | 8.31 | 0.07 | (8.18, 8.44) |  |
|  | 30-39 | 8.35 | 0.06 | (8.23, 8.47) |  |
|  | 40-49 | 8.53 | 0.11 | (8.31, 8.74) |  |
|  | 50-59 | 8.78 | 0.16 | (8.48, 9.09) |  |
|  | 60-69 | 8.46 | 0.23 | (8.00, 8.91) |  |
|  | 70-79 | 8.21 | 0.57 | (7.08, 9.34) |  |
|  | 80 or older | 8.65 | 0.25 | (8.15, 9.15) |  |
| Gender | Male | 8.35 | 0.07 | (8.22, 8.48) | 0.006 |
|  | Female | 8.48 | 0.05 | (8.38, 8.58) |  |
| Marital status | Married | 8.35 | 0.06 | (8.23, 8.46) | 0.022 |
|  | Separated | 8.40 | 0.29 | (7.82, 8.98) |  |
|  | Divorced | 8.57 | 0.32 | (7.93, 9.21) |  |
|  | Widowed | 8.90 | 0.21 | (8.48, 9.32) |  |
|  | Never | 8.47 | 0.06 | (8.36, 8.59) |  |
|  | Domestic Partner | 8.74 | 0.83 | (6.88, 10.61) |  |
| Employment | Employed for an employer | 8.50 | 0.10 | (8.31, 8.69) | 0.249 |
|  | Self-employed | 8.47 | 0.05 | (8.37, 8.57) |  |
|  | Retired | 8.49 | 0.32 | (7.86, 9.12) |  |
|  | Student | 8.41 | 0.13 | (8.15, 8.67) |  |
|  | Homemaker | 8.12 | 0.17 | (7.79, 8.45) |  |
|  | Unemployed and looking for a job | 8.23 | 0.13 | (7.99, 8.48) |  |
|  | None of these/other | 8.34 | 0.24 | (7.86, 8.82) |  |
| Religious service attendance | >1/week | 8.60 | 0.05 | (8.51, 8.70) | <0.001 |
|  | 1/week | 8.23 | 0.08 | (8.08, 8.38) |  |
|  | 1-3/month | 7.73 | 0.17 | (7.38, 8.07) |  |
|  | A few times a year | 8.36 | 0.19 | (7.98, 8.73) |  |
|  | Never | 7.71 | 0.45 | (6.82, 8.60) |  |
| Education | up to 8 years | 8.37 | 0.10 | (8.18, 8.56) | 0.946 |
|  | 9-15 years | 8.44 | 0.04 | (8.36, 8.53) |  |
|  | 16+years | 8.33 | 0.19 | (7.94, 8.71) |  |
| Immigration status | Born in this country | 8.41 | 0.04 | (8.33, 8.50) | 0.838 |
|  | Born in another country | 8.19 | 0.40 | (7.38, 8.99) |  |
| Religious affiliation | Christianity | 8.57 | 0.05 | (8.47, 8.67) | <0.001 |
|  | Islam | 8.24 | 0.07 | (8.10, 8.39) |  |
|  | Primal, Animist, or Folk religion | 8.43 | 0.69 | (6.99, 9.88) |  |
|  | No religion/Atheist/Agnostic | 8.86 | 0.79 | (7.14, 10.57) |  |
| Race and ethnicity | Other | 8.39 | 0.10 | (8.20, 8.58) | <0.001 |
|  | Edo | 8.55 | 0.30 | (7.96, 9.13) |  |
|  | Efik | 8.59 | 0.43 | (7.73, 9.45) |  |
|  | Fulani | 8.24 | 0.19 | (7.87, 8.62) |  |
|  | Hausa | 8.23 | 0.10 | (8.04, 8.42) |  |
|  | Ibibio | 8.50 | 0.33 | (7.85, 9.16) |  |
|  | Idoma | 8.24 | 0.42 | (7.40, 9.07) |  |
|  | Igala | 8.03 | 0.29 | (7.44, 8.61) |  |
|  | Igbo (Ibo) | 8.79 | 0.07 | (8.65, 8.93) |  |
|  | Ijaw | 8.82 | 0.51 | (7.82, 9.82) |  |
|  | Kanuri | 8.21 | 0.42 | (7.34, 9.08) |  |
|  | Tiv | 8.25 | 0.21 | (7.85, 8.66) |  |
|  | Urhobo | 8.82 | 0.28 | (8.24, 9.40) |  |
|  | Yoruba | 8.46 | 0.09 | (8.27, 8.64) |  |

Table S14a. Nationally representative descriptive statistics for Philippines

| **Characteristic** | **N = 5,292**^1^ |
| --- | --- |
| **Age group** |  |
| 18-24 | 1,073 (20%) |
| 25-29 | 695 (13%) |
| 30-39 | 1,160 (22%) |
| 40-49 | 972 (18%) |
| 50-59 | 732 (14%) |
| 60-69 | 495 (9.4%) |
| 70-79 | 143 (2.7%) |
| 80 or older | 23 (0.4%) |
| Missing | 0 (0%) |
| **Gender** |  |
| Male | 2,625 (50%) |
| Female | 2,643 (50%) |
| Other | 13 (0.2%) |
| Missing | 11 (0.2%) |
| **Marital status** |  |
| Married | 2,385 (45%) |
| Separated | 249 (4.7%) |
| Divorced | 9 (0.2%) |
| Widowed | 274 (5.2%) |
| Never | 1,206 (23%) |
| Domestic Partner | 1,152 (22%) |
| Missing | 16 (0.3%) |
| **Employment** |  |
| Employed for an employer | 1,350 (26%) |
| Self-employed | 1,379 (26%) |
| Retired | 158 (3.0%) |
| Student | 585 (11%) |
| Homemaker | 1,049 (20%) |
| Unemployed and looking for a job | 658 (12%) |
| None of these/other | 113 (2.1%) |
| Missing | 0 (0%) |
| **Religious service attendance** |  |
| >1/week | 844 (16%) |
| 1/week | 1,929 (36%) |
| 1-3/month | 1,374 (26%) |
| A few times a year | 929 (18%) |
| Never | 210 (4.0%) |
| Missing | 6 (0.1%) |
| **Education** |  |
| up to 8 years | 1,188 (22%) |
| 9-15 years | 3,722 (70%) |
| 16+years | 381 (7.2%) |
| Missing | 1 (<0.1%) |
| **Immigration** |  |
| Born in this country | 5,284 (100%) |
| Born in another country | 8 (0.1%) |
| Missing | 0 (0%) |
| **Religious affiliation** |  |
| Christianity | 4,914 (93%) |
| Islam | 297 (5.6%) |
| Buddhism | 4 (<0.1%) |
| Judaism | 4 (<0.1%) |
| Baha’i | 1 (<0.1%) |
| Primal, Animist, or Folk religion | 5 (<0.1%) |
| Some other religion | 35 (0.7%) |
| No religion/Atheist/Agnostic | 23 (0.4%) |
| Missing | 9 (0.2%) |
| **Race and ethnicity** |  |
| Aeta | 1 (<0.1%) |
| Badjao | 2 (<0.1%) |
| Bicolano/Bikolano | 300 (5.7%) |
| Cebuano | 656 (12%) |
| Chinese-Filipino | 3 (<0.1%) |
| Igorot | 42 (0.8%) |
| Ilocano/Ilokano | 429 (8.1%) |
| Ilonggo/Hiligaynon | 428 (8.1%) |
| Kapampangan | 107 (2.0%) |
| Maguindanaoan | 84 (1.6%) |
| Mangyan | 2 (<0.1%) |
| Maranao | 39 (0.7%) |
| Masbateno | 54 (1.0%) |
| Other | 244 (4.6%) |
| Pangasinense | 107 (2.0%) |
| Tagalog | 1,691 (32%) |
| Tausug | 94 (1.8%) |
| Visayan/Bisaya | 739 (14%) |
| Waray | 216 (4.1%) |
| Zamboangueno | 51 (1.0%) |
| Missing | 3 (<0.1%) |
| ^1^n (%) | |

Table S14b. Means by demographic category for Philippines

| Variable | Category | Mean | SE | 95% CI | Global p-value |
| --- | --- | --- | --- | --- | --- |
| Age group | 18-24 | 8.75 | 0.07 | (8.62, 8.88) | <0.001 |
|  | 25-29 | 8.87 | 0.10 | (8.67, 9.07) |  |
|  | 30-39 | 8.86 | 0.06 | (8.74, 8.98) |  |
|  | 40-49 | 8.41 | 0.08 | (8.25, 8.57) |  |
|  | 50-59 | 8.53 | 0.09 | (8.36, 8.71) |  |
|  | 60-69 | 8.26 | 0.16 | (7.95, 8.57) |  |
|  | 70-79 | 8.24 | 0.20 | (7.84, 8.63) |  |
|  | 80 or older | 8.47 | 0.41 | (7.62, 9.33) |  |
| Gender | Male | 8.47 | 0.06 | (8.35, 8.58) | <0.001 |
|  | Female | 8.81 | 0.04 | (8.74, 8.88) |  |
|  | Other | 7.77 | 0.73 | (6.13, 9.40) |  |
| Marital status | Married | 8.60 | 0.05 | (8.49, 8.70) | <0.001 |
|  | Separated | 8.33 | 0.15 | (8.03, 8.63) |  |
|  | Divorced | 7.59 | 0.90 | (5.46, 9.72) |  |
|  | Widowed | 8.49 | 0.16 | (8.17, 8.81) |  |
|  | Never | 8.58 | 0.07 | (8.44, 8.73) |  |
|  | Domestic Partner | 8.89 | 0.06 | (8.77, 9.01) |  |
| Employment | Employed for an employer | 8.63 | 0.07 | (8.48, 8.78) | 0.154 |
|  | Self-employed | 8.67 | 0.06 | (8.55, 8.80) |  |
|  | Retired | 8.27 | 0.24 | (7.80, 8.74) |  |
|  | Student | 8.76 | 0.08 | (8.60, 8.93) |  |
|  | Homemaker | 8.70 | 0.07 | (8.57, 8.83) |  |
|  | Unemployed and looking for a job | 8.49 | 0.13 | (8.24, 8.74) |  |
|  | None of these/other | 8.43 | 0.23 | (7.97, 8.89) |  |
| Religious service attendance | >1/week | 8.64 | 0.10 | (8.45, 8.83) | <0.001 |
|  | 1/week | 8.74 | 0.05 | (8.64, 8.85) |  |
|  | 1-3/month | 8.57 | 0.07 | (8.42, 8.71) |  |
|  | A few times a year | 8.65 | 0.07 | (8.51, 8.78) |  |
|  | Never | 8.05 | 0.24 | (7.58, 8.52) |  |
| Education | up to 8 years | 8.30 | 0.10 | (8.10, 8.50) | <0.001 |
|  | 9-15 years | 8.72 | 0.04 | (8.65, 8.79) |  |
|  | 16+years | 8.90 | 0.10 | (8.70, 9.10) |  |
| Immigration status | Born in this country | 8.64 | 0.04 | (8.57, 8.71) | 0.785 |
|  | Born in another country | 8.20 | 0.70 | (6.44, 9.96) |  |
| Religious affiliation | Christianity | 8.64 | 0.04 | (8.57, 8.71) | <0.001 |
|  | Islam | 8.58 | 0.15 | (8.29, 8.86) |  |
|  | Buddhism | 9.80 | 0.39 | (5.89, 13.71) |  |
|  | Judaism | 10.00 |  |  |  |
|  | Primal, Animist, or Folk religion | 8.20 | 0.76 | (5.74, 10.66) |  |
|  | Some other religion | 8.81 | 0.28 | (8.23, 9.39) |  |
|  | No religion/Atheist/Agnostic | 8.12 | 0.66 | (6.75, 9.50) |  |
| Race and ethnicity | Other | 8.57 | 0.13 | (8.31, 8.82) | <0.001 |
|  | Aeta | 10.00 |  |  |  |
|  | Badjao | 9.46 |  |  |  |
|  | Bicolano/Bikolano | 8.68 | 0.12 | (8.45, 8.91) |  |
|  | Cebuano | 8.52 | 0.10 | (8.32, 8.72) |  |
|  | Chinese-Filipino | 9.60 | 0.27 | (7.74, 11.45) |  |
|  | Igorot | 8.47 | 0.28 | (7.91, 9.03) |  |
|  | Ilocano/Ilokano | 8.50 | 0.15 | (8.21, 8.78) |  |
|  | Ilonggo/Hiligaynon | 8.72 | 0.10 | (8.52, 8.92) |  |
|  | Kapampangan | 8.70 | 0.22 | (8.25, 9.14) |  |
|  | Maguindanaoan | 8.23 | 0.17 | (7.88, 8.58) |  |
|  | Mangyan | 7.27 |  |  |  |
|  | Maranao | 8.30 | 0.46 | (7.38, 9.23) |  |
|  | Masbateno | 7.86 | 0.29 | (7.27, 8.45) |  |
|  | Pangasinense | 9.04 | 0.13 | (8.77, 9.30) |  |
|  | Tagalog | 8.85 | 0.05 | (8.75, 8.96) |  |
|  | Tausug | 8.98 | 0.19 | (8.61, 9.35) |  |
|  | Visayan/Bisaya | 8.46 | 0.10 | (8.26, 8.66) |  |
|  | Waray | 8.17 | 0.23 | (7.73, 8.62) |  |
|  | Zamboangueno | 8.33 | 0.26 | (7.80, 8.87) |  |

Table S15a. Nationally representative descriptive statistics for Poland

| **Characteristic** | **N = 10,389**^1^ |
| --- | --- |
| **Age group** |  |
| 18-24 | 955 (9.2%) |
| 25-29 | 761 (7.3%) |
| 30-39 | 2,159 (21%) |
| 40-49 | 1,956 (19%) |
| 50-59 | 1,670 (16%) |
| 60-69 | 1,909 (18%) |
| 70-79 | 833 (8.0%) |
| 80 or older | 145 (1.4%) |
| Missing | 1 (<0.1%) |
| **Gender** |  |
| Male | 4,974 (48%) |
| Female | 5,387 (52%) |
| Other | 3 (<0.1%) |
| Missing | 26 (0.2%) |
| **Marital status** |  |
| Married | 6,065 (58%) |
| Separated | 111 (1.1%) |
| Divorced | 529 (5.1%) |
| Widowed | 990 (9.5%) |
| Never | 1,811 (17%) |
| Domestic Partner | 504 (4.8%) |
| Missing | 379 (3.6%) |
| **Employment** |  |
| Employed for an employer | 5,837 (56%) |
| Self-employed | 686 (6.6%) |
| Retired | 2,434 (23%) |
| Student | 515 (5.0%) |
| Homemaker | 338 (3.3%) |
| Unemployed and looking for a job | 284 (2.7%) |
| None of these/other | 169 (1.6%) |
| Missing | 126 (1.2%) |
| **Religious service attendance** |  |
| >1/week | 305 (2.9%) |
| 1/week | 3,263 (31%) |
| 1-3/month | 2,081 (20%) |
| A few times a year | 3,064 (29%) |
| Never | 1,597 (15%) |
| Missing | 78 (0.8%) |
| **Education** |  |
| up to 8 years | 1,238 (12%) |
| 9-15 years | 6,130 (59%) |
| 16+years | 3,020 (29%) |
| Missing | 1 (<0.1%) |
| **Immigration** |  |
| Born in this country | 10,258 (99%) |
| Born in another country | 108 (1.0%) |
| Missing | 23 (0.2%) |
| **Religious affiliation** |  |
| Christianity | 9,378 (90%) |
| Islam | 2 (<0.1%) |
| Buddhism | 2 (<0.1%) |
| Sikhism | 1 (<0.1%) |
| Jainism | 3 (<0.1%) |
| Shinto | 1 (<0.1%) |
| Primal, Animist, or Folk religion | 11 (0.1%) |
| No religion/Atheist/Agnostic | 942 (9.1%) |
| Missing | 50 (0.5%) |
| **Race and ethnicity** |  |
| Belarussian | 2 (<0.1%) |
| German | 4 (<0.1%) |
| Kashubians | 3 (<0.1%) |
| Other | 4 (<0.1%) |
| Polish | 10,309 (99%) |
| Silesia | 14 (0.1%) |
| Ukrainian | 38 (0.4%) |
| Missing | 14 (0.1%) |
| ^1^n (%) | |

Table S15b. Means by demographic category for Poland

| Variable | Category | Mean | SE | 95% CI | Global p-value |
| --- | --- | --- | --- | --- | --- |
| Age group | 18-24 | 7.05 | 0.12 | (6.82, 7.28) | <0.001 |
|  | 25-29 | 7.07 | 0.10 | (6.88, 7.26) |  |
|  | 30-39 | 7.32 | 0.07 | (7.18, 7.47) |  |
|  | 40-49 | 7.27 | 0.07 | (7.13, 7.41) |  |
|  | 50-59 | 7.31 | 0.07 | (7.16, 7.45) |  |
|  | 60-69 | 7.60 | 0.09 | (7.43, 7.77) |  |
|  | 70-79 | 7.82 | 0.11 | (7.60, 8.04) |  |
|  | 80 or older | 7.06 | 0.27 | (6.52, 7.59) |  |
| Gender | Male | 7.22 | 0.06 | (7.09, 7.34) | <0.001 |
|  | Female | 7.48 | 0.05 | (7.37, 7.59) |  |
|  | Other | 6.06 | 0.55 | (2.53, 9.59) |  |
| Marital status | Married | 7.52 | 0.05 | (7.42, 7.62) | <0.001 |
|  | Separated | 6.99 | 0.20 | (6.60, 7.38) |  |
|  | Divorced | 6.91 | 0.15 | (6.61, 7.20) |  |
|  | Widowed | 7.50 | 0.11 | (7.28, 7.71) |  |
|  | Never | 6.92 | 0.09 | (6.74, 7.11) |  |
|  | Domestic Partner | 7.14 | 0.10 | (6.94, 7.34) |  |
| Employment | Employed for an employer | 7.34 | 0.05 | (7.23, 7.44) | <0.001 |
|  | Self-employed | 7.07 | 0.13 | (6.81, 7.32) |  |
|  | Retired | 7.65 | 0.07 | (7.50, 7.79) |  |
|  | Student | 7.13 | 0.15 | (6.83, 7.44) |  |
|  | Homemaker | 7.30 | 0.17 | (6.97, 7.63) |  |
|  | Unemployed and looking for a job | 6.45 | 0.27 | (5.92, 6.97) |  |
|  | None of these/other | 7.15 | 0.24 | (6.67, 7.63) |  |
| Religious service attendance | >1/week | 8.03 | 0.24 | (7.55, 8.51) | <0.001 |
|  | 1/week | 7.63 | 0.07 | (7.50, 7.76) |  |
|  | 1-3/month | 7.30 | 0.08 | (7.14, 7.47) |  |
|  | A few times a year | 7.27 | 0.07 | (7.14, 7.40) |  |
|  | Never | 6.89 | 0.11 | (6.67, 7.11) |  |
| Education | up to 8 years | 6.99 | 0.18 | (6.63, 7.35) | <0.001 |
|  | 9-15 years | 7.34 | 0.05 | (7.24, 7.45) |  |
|  | 16+years | 7.52 | 0.06 | (7.40, 7.64) |  |
| Immigration status | Born in this country | 7.36 | 0.05 | (7.25, 7.46) | 0.720 |
|  | Born in another country | 7.13 | 0.34 | (6.45, 7.80) |  |
| Religious affiliation | Christianity | 7.38 | 0.05 | (7.27, 7.48) | 0.449 |
|  | Islam | 7.60 |  |  |  |
|  | Buddhism | 5.96 |  |  |  |
|  | Primal, Animist, or Folk religion | 7.59 | 0.56 | (6.31, 8.87) |  |
|  | No religion/Atheist/Agnostic | 7.12 | 0.15 | (6.83, 7.40) |  |
| Race and ethnicity | Other | 6.21 | 1.30 | (1.11, 11.30) | <0.001 |
|  | Belarussian | 6.41 |  |  |  |
|  | German | 8.47 | 0.31 | (6.58, 10.35) |  |
|  | Kashubians | 8.49 | 0.53 | (4.06, 12.92) |  |
|  | Polish | 7.35 | 0.05 | (7.25, 7.45) |  |
|  | Silesia | 8.99 | 0.34 | (8.24, 9.74) |  |
|  | Ukrainian | 7.46 | 0.26 | (6.93, 7.98) |  |

Table S16a. Nationally representative descriptive statistics for South Africa

| **Characteristic** | **N = 2,651**^1^ |
| --- | --- |
| **Age group** |  |
| 18-24 | 461 (17%) |
| 25-29 | 364 (14%) |
| 30-39 | 655 (25%) |
| 40-49 | 522 (20%) |
| 50-59 | 309 (12%) |
| 60-69 | 195 (7.4%) |
| 70-79 | 120 (4.5%) |
| 80 or older | 17 (0.6%) |
| Missing | 9 (0.3%) |
| **Gender** |  |
| Male | 1,288 (49%) |
| Female | 1,356 (51%) |
| Other | 2 (<0.1%) |
| Missing | 4 (0.2%) |
| **Marital status** |  |
| Married | 539 (20%) |
| Separated | 76 (2.9%) |
| Divorced | 51 (1.9%) |
| Widowed | 133 (5.0%) |
| Never | 1,561 (59%) |
| Domestic Partner | 264 (10.0%) |
| Missing | 28 (1.0%) |
| **Employment** |  |
| Employed for an employer | 569 (21%) |
| Self-employed | 412 (16%) |
| Retired | 243 (9.2%) |
| Student | 204 (7.7%) |
| Homemaker | 137 (5.2%) |
| Unemployed and looking for a job | 1,008 (38%) |
| None of these/other | 74 (2.8%) |
| Missing | 3 (0.1%) |
| **Religious service attendance** |  |
| >1/week | 414 (16%) |
| 1/week | 891 (34%) |
| 1-3/month | 574 (22%) |
| A few times a year | 431 (16%) |
| Never | 334 (13%) |
| Missing | 7 (0.3%) |
| **Education** |  |
| up to 8 years | 668 (25%) |
| 9-15 years | 1,796 (68%) |
| 16+years | 183 (6.9%) |
| Missing | 4 (0.2%) |
| **Immigration** |  |
| Born in this country | 2,511 (95%) |
| Born in another country | 139 (5.2%) |
| Missing | 1 (<0.1%) |
| **Religious affiliation** |  |
| Christianity | 2,163 (82%) |
| Islam | 62 (2.3%) |
| Hinduism | 1 (<0.1%) |
| Buddhism | 12 (0.5%) |
| Jainism | 2 (<0.1%) |
| Shinto | 2 (<0.1%) |
| Taoism | 1 (<0.1%) |
| Primal, Animist, or Folk religion | 127 (4.8%) |
| Some other religion | 5 (0.2%) |
| No religion/Atheist/Agnostic | 253 (9.6%) |
| Missing | 23 (0.9%) |
| **Race and ethnicity** |  |
| Asian/Indian | 6 (0.2%) |
| Black | 2,381 (90%) |
| Colored | 252 (9.5%) |
| Other | 1 (<0.1%) |
| White | 8 (0.3%) |
| Missing | 3 (0.1%) |
| ^1^n (%) | |

Table S16b. Means by demographic category for South Africa

| Variable | Category | Mean | SE | 95% CI | Global p-value |
| --- | --- | --- | --- | --- | --- |
| Age group | 18-24 | 7.92 | 0.14 | (7.66, 8.19) | 0.609 |
|  | 25-29 | 7.66 | 0.14 | (7.39, 7.93) |  |
|  | 30-39 | 7.94 | 0.10 | (7.73, 8.14) |  |
|  | 40-49 | 7.82 | 0.13 | (7.56, 8.09) |  |
|  | 50-59 | 8.07 | 0.19 | (7.70, 8.43) |  |
|  | 60-69 | 7.64 | 0.34 | (6.97, 8.32) |  |
|  | 70-79 | 7.45 | 0.55 | (6.35, 8.54) |  |
|  | 80 or older | 8.43 | 0.48 | (7.41, 9.46) |  |
| Gender | Male | 7.85 | 0.10 | (7.66, 8.04) | 0.098 |
|  | Female | 7.85 | 0.09 | (7.68, 8.02) |  |
|  | Other | 6.33 |  |  |  |
| Marital status | Married | 8.05 | 0.15 | (7.75, 8.34) | 0.074 |
|  | Separated | 7.40 | 0.32 | (6.75, 8.04) |  |
|  | Divorced | 7.22 | 0.51 | (6.20, 8.24) |  |
|  | Widowed | 8.13 | 0.39 | (7.36, 8.90) |  |
|  | Never | 7.79 | 0.08 | (7.64, 7.94) |  |
|  | Domestic Partner | 7.89 | 0.17 | (7.56, 8.22) |  |
| Employment | Employed for an employer | 7.91 | 0.12 | (7.67, 8.16) | 0.318 |
|  | Self-employed | 8.12 | 0.15 | (7.83, 8.42) |  |
|  | Retired | 7.70 | 0.37 | (6.98, 8.42) |  |
|  | Student | 7.94 | 0.19 | (7.56, 8.32) |  |
|  | Homemaker | 7.27 | 0.32 | (6.64, 7.90) |  |
|  | Unemployed and looking for a job | 7.79 | 0.10 | (7.59, 7.99) |  |
|  | None of these/other | 7.90 | 0.50 | (6.91, 8.89) |  |
| Religious service attendance | >1/week | 8.15 | 0.17 | (7.82, 8.48) | 0.264 |
|  | 1/week | 7.79 | 0.12 | (7.55, 8.04) |  |
|  | 1-3/month | 7.87 | 0.13 | (7.62, 8.12) |  |
|  | A few times a year | 7.78 | 0.15 | (7.47, 8.08) |  |
|  | Never | 7.67 | 0.17 | (7.34, 8.00) |  |
| Education | up to 8 years | 7.85 | 0.19 | (7.48, 8.21) | 1.000 |
|  | 9-15 years | 7.84 | 0.07 | (7.71, 7.98) |  |
|  | 16+years | 7.91 | 0.16 | (7.61, 8.22) |  |
| Immigration status | Born in this country | 7.86 | 0.07 | (7.72, 8.00) | 0.687 |
|  | Born in another country | 7.67 | 0.26 | (7.15, 8.19) |  |
| Religious affiliation | Christianity | 7.91 | 0.07 | (7.76, 8.05) | <0.001 |
|  | Islam | 7.76 | 0.46 | (6.85, 8.68) |  |
|  | Buddhism | 8.49 | 0.32 | (7.78, 9.19) |  |
|  | Shinto | 9.11 |  |  |  |
|  | Taoism | 10.00 |  |  |  |
|  | Primal, Animist, or Folk religion | 7.07 | 0.33 | (6.41, 7.73) |  |
|  | Some other religion | 7.79 | 0.95 | (4.57, 11.00) |  |
|  | No religion/Atheist/Agnostic | 7.67 | 0.20 | (7.28, 8.06) |  |
| Race and ethnicity | Black | 7.82 | 0.07 | (7.68, 7.95) | <0.001 |
|  | White | 8.58 | 1.07 | (5.95, 11.20) |  |
|  | Asian/Indian | 9.71 | 0.35 | (8.68, 10.74) |  |
|  | Colored | 8.08 | 0.26 | (7.56, 8.60) |  |

Table S17a. Nationally representative descriptive statistics for Spain

| **Characteristic** | **N = 6,290**^1^ |
| --- | --- |
| **Age group** |  |
| 18-24 | 594 (9.4%) |
| 25-29 | 450 (7.2%) |
| 30-39 | 1,111 (18%) |
| 40-49 | 1,396 (22%) |
| 50-59 | 1,252 (20%) |
| 60-69 | 977 (16%) |
| 70-79 | 467 (7.4%) |
| 80 or older | 43 (0.7%) |
| Missing | 0 (0%) |
| **Gender** |  |
| Male | 3,142 (50%) |
| Female | 3,119 (50%) |
| Other | 6 (0.1%) |
| Missing | 22 (0.4%) |
| **Marital status** |  |
| Married | 2,947 (47%) |
| Separated | 237 (3.8%) |
| Divorced | 518 (8.2%) |
| Widowed | 189 (3.0%) |
| Never | 1,742 (28%) |
| Domestic Partner | 589 (9.4%) |
| Missing | 67 (1.1%) |
| **Employment** |  |
| Employed for an employer | 2,862 (45%) |
| Self-employed | 576 (9.2%) |
| Retired | 1,278 (20%) |
| Student | 448 (7.1%) |
| Homemaker | 345 (5.5%) |
| Unemployed and looking for a job | 646 (10%) |
| None of these/other | 123 (2.0%) |
| Missing | 11 (0.2%) |
| **Religious service attendance** |  |
| >1/week | 317 (5.0%) |
| 1/week | 662 (11%) |
| 1-3/month | 437 (6.9%) |
| A few times a year | 1,972 (31%) |
| Never | 2,875 (46%) |
| Missing | 27 (0.4%) |
| **Education** |  |
| up to 8 years | 802 (13%) |
| 9-15 years | 4,145 (66%) |
| 16+years | 1,341 (21%) |
| Missing | 2 (<0.1%) |
| **Immigration** |  |
| Born in this country | 5,479 (87%) |
| Born in another country | 788 (13%) |
| Missing | 23 (0.4%) |
| **Religious affiliation** |  |
| Christianity | 4,074 (65%) |
| Islam | 135 (2.1%) |
| Hinduism | 7 (0.1%) |
| Buddhism | 36 (0.6%) |
| Judaism | 4 (<0.1%) |
| Sikhism | 3 (<0.1%) |
| Baha’i | 2 (<0.1%) |
| Jainism | 1 (<0.1%) |
| Taoism | 5 (<0.1%) |
| Confucianism | 3 (<0.1%) |
| Primal, Animist, or Folk religion | 7 (0.1%) |
| Some other religion | 27 (0.4%) |
| No religion/Atheist/Agnostic | 1,932 (31%) |
| Missing | 55 (0.9%) |
| **Race and ethnicity** |  |
| Missing | 6,290 (100%) |
| ^1^n (%) | |

Table S17b. Means by demographic category for Spain

| Variable | Category | Mean | SE | 95% CI | Global p-value |
| --- | --- | --- | --- | --- | --- |
| Age group | 18-24 | 7.68 | 0.11 | (7.46, 7.90) | 0.008 |
|  | 25-29 | 7.72 | 0.12 | (7.49, 7.96) |  |
|  | 30-39 | 7.66 | 0.07 | (7.52, 7.80) |  |
|  | 40-49 | 7.84 | 0.06 | (7.72, 7.96) |  |
|  | 50-59 | 7.86 | 0.07 | (7.71, 8.00) |  |
|  | 60-69 | 7.78 | 0.11 | (7.57, 7.99) |  |
|  | 70-79 | 7.93 | 0.18 | (7.57, 8.28) |  |
|  | 80 or older | 8.53 | 0.32 | (7.88, 9.17) |  |
| Gender | Male | 7.76 | 0.05 | (7.67, 7.85) | 0.002 |
|  | Female | 7.82 | 0.05 | (7.72, 7.92) |  |
|  | Other | 8.42 | 0.29 | (7.63, 9.22) |  |
| Marital status | Married | 7.90 | 0.05 | (7.81, 8.00) | <0.001 |
|  | Separated | 7.58 | 0.19 | (7.21, 7.95) |  |
|  | Divorced | 7.73 | 0.15 | (7.45, 8.02) |  |
|  | Widowed | 7.38 | 0.27 | (6.84, 7.91) |  |
|  | Never | 7.68 | 0.06 | (7.56, 7.80) |  |
|  | Domestic Partner | 7.81 | 0.10 | (7.62, 8.00) |  |
| Employment | Employed for an employer | 7.79 | 0.04 | (7.71, 7.88) | <0.001 |
|  | Self-employed | 8.04 | 0.09 | (7.87, 8.22) |  |
|  | Retired | 7.73 | 0.10 | (7.52, 7.93) |  |
|  | Student | 7.64 | 0.13 | (7.38, 7.91) |  |
|  | Homemaker | 8.00 | 0.15 | (7.72, 8.29) |  |
|  | Unemployed and looking for a job | 7.67 | 0.10 | (7.48, 7.86) |  |
|  | None of these/other | 7.77 | 0.21 | (7.35, 8.19) |  |
| Religious service attendance | >1/week | 8.34 | 0.15 | (8.05, 8.62) | <0.001 |
|  | 1/week | 8.16 | 0.09 | (7.99, 8.34) |  |
|  | 1-3/month | 7.87 | 0.12 | (7.63, 8.11) |  |
|  | A few times a year | 7.96 | 0.06 | (7.84, 8.07) |  |
|  | Never | 7.52 | 0.05 | (7.41, 7.62) |  |
| Education | up to 8 years | 7.77 | 0.11 | (7.55, 7.99) | <0.001 |
|  | 9-15 years | 7.71 | 0.04 | (7.63, 7.79) |  |
|  | 16+years | 8.04 | 0.07 | (7.90, 8.18) |  |
| Immigration status | Born in this country | 7.68 | 0.04 | (7.61, 7.76) | <0.001 |
|  | Born in another country | 8.53 | 0.07 | (8.39, 8.67) |  |
| Religious affiliation | Christianity | 7.94 | 0.04 | (7.86, 8.02) | <0.001 |
|  | Islam | 7.26 | 0.30 | (6.67, 7.85) |  |
|  | Hinduism | 7.30 | 0.81 | (5.11, 9.49) |  |
|  | Buddhism | 7.75 | 0.55 | (6.63, 8.87) |  |
|  | Judaism | 6.31 | 1.64 | (0.09, 12.54) |  |
|  | Sikhism | 7.67 | 1.26 | (-2.34, 17.69) |  |
|  | Baha’i | 7.71 |  |  |  |
|  | Taoism | 5.29 | 1.88 | (-1.39, 11.98) |  |
|  | Confucianism | 3.20 |  |  |  |
|  | Primal, Animist, or Folk religion | 8.74 | 0.38 | (7.76, 9.72) |  |
|  | Some other religion | 8.50 | 0.38 | (7.71, 9.28) |  |
|  | No religion/Atheist/Agnostic | 7.51 | 0.07 | (7.39, 7.64) |  |

Table S18a. Nationally representative descriptive statistics for Sweden

| **Characteristic** | **N = 15,068**^1^ |
| --- | --- |
| **Age group** |  |
| 18-24 | 1,515 (10%) |
| 25-29 | 1,399 (9.3%) |
| 30-39 | 2,398 (16%) |
| 40-49 | 2,221 (15%) |
| 50-59 | 2,493 (17%) |
| 60-69 | 2,168 (14%) |
| 70-79 | 2,253 (15%) |
| 80 or older | 621 (4.1%) |
| Missing | 0 (0%) |
| **Gender** |  |
| Male | 7,536 (50%) |
| Female | 7,493 (50%) |
| Other | 27 (0.2%) |
| Missing | 12 (<0.1%) |
| **Marital status** |  |
| Married | 6,408 (43%) |
| Separated | 426 (2.8%) |
| Divorced | 801 (5.3%) |
| Widowed | 433 (2.9%) |
| Never | 3,854 (26%) |
| Domestic Partner | 3,073 (20%) |
| Missing | 72 (0.5%) |
| **Employment** |  |
| Employed for an employer | 7,907 (52%) |
| Self-employed | 1,243 (8.3%) |
| Retired | 3,832 (25%) |
| Student | 1,332 (8.8%) |
| Homemaker | 75 (0.5%) |
| Unemployed and looking for a job | 324 (2.2%) |
| None of these/other | 337 (2.2%) |
| Missing | 18 (0.1%) |
| **Religious service attendance** |  |
| >1/week | 236 (1.6%) |
| 1/week | 434 (2.9%) |
| 1-3/month | 486 (3.2%) |
| A few times a year | 3,950 (26%) |
| Never | 9,918 (66%) |
| Missing | 45 (0.3%) |
| **Education** |  |
| up to 8 years | 252 (1.7%) |
| 9-15 years | 10,790 (72%) |
| 16+years | 4,026 (27%) |
| Missing | 0 (0%) |
| **Immigration** |  |
| Born in this country | 13,922 (92%) |
| Born in another country | 1,052 (7.0%) |
| Missing | 94 (0.6%) |
| **Religious affiliation** |  |
| Christianity | 8,346 (55%) |
| Islam | 470 (3.1%) |
| Hinduism | 22 (0.1%) |
| Buddhism | 110 (0.7%) |
| Judaism | 54 (0.4%) |
| Sikhism | 4 (<0.1%) |
| Baha’i | 6 (<0.1%) |
| Shinto | 0 (<0.1%) |
| Taoism | 4 (<0.1%) |
| Primal, Animist, or Folk religion | 83 (0.5%) |
| Some other religion | 198 (1.3%) |
| No religion/Atheist/Agnostic | 5,697 (38%) |
| Missing | 74 (0.5%) |
| **Race and ethnicity** |  |
| Missing | 15,068 (100%) |
| ^1^n (%) | |

Table S18b. Means by demographic category for Sweden

| Variable | Category | Mean | SE | 95% CI | Global p-value |
| --- | --- | --- | --- | --- | --- |
| Age group | 18-24 | 7.23 | 0.07 | (7.09, 7.37) | <0.001 |
|  | 25-29 | 7.14 | 0.08 | (6.98, 7.30) |  |
|  | 30-39 | 7.27 | 0.06 | (7.16, 7.38) |  |
|  | 40-49 | 7.31 | 0.06 | (7.19, 7.43) |  |
|  | 50-59 | 7.46 | 0.05 | (7.35, 7.56) |  |
|  | 60-69 | 7.68 | 0.05 | (7.58, 7.78) |  |
|  | 70-79 | 7.96 | 0.05 | (7.86, 8.05) |  |
|  | 80 or older | 8.05 | 0.09 | (7.88, 8.23) |  |
| Gender | Male | 7.23 | 0.03 | (7.17, 7.29) | <0.001 |
|  | Female | 7.74 | 0.03 | (7.68, 7.80) |  |
|  | Other | 5.78 | 0.60 | (4.53, 7.02) |  |
| Marital status | Married | 7.90 | 0.03 | (7.84, 7.96) | <0.001 |
|  | Separated | 7.20 | 0.13 | (6.94, 7.46) |  |
|  | Divorced | 7.51 | 0.08 | (7.34, 7.68) |  |
|  | Widowed | 7.86 | 0.11 | (7.64, 8.08) |  |
|  | Never | 6.76 | 0.05 | (6.67, 6.86) |  |
|  | Domestic Partner | 7.49 | 0.04 | (7.41, 7.58) |  |
| Employment | Employed for an employer | 7.38 | 0.03 | (7.32, 7.44) | <0.001 |
|  | Self-employed | 7.90 | 0.09 | (7.73, 8.07) |  |
|  | Retired | 7.83 | 0.04 | (7.76, 7.91) |  |
|  | Student | 7.32 | 0.07 | (7.18, 7.47) |  |
|  | Homemaker | 7.45 | 0.34 | (6.78, 8.12) |  |
|  | Unemployed and looking for a job | 6.17 | 0.17 | (5.84, 6.50) |  |
|  | None of these/other | 6.40 | 0.18 | (6.04, 6.76) |  |
| Religious service attendance | >1/week | 8.59 | 0.20 | (8.19, 8.99) | <0.001 |
|  | 1/week | 8.08 | 0.14 | (7.81, 8.35) |  |
|  | 1-3/month | 7.91 | 0.11 | (7.69, 8.13) |  |
|  | A few times a year | 7.91 | 0.04 | (7.84, 7.99) |  |
|  | Never | 7.24 | 0.03 | (7.19, 7.29) |  |
| Education | up to 8 years | 7.64 | 0.17 | (7.30, 7.97) | <0.001 |
|  | 9-15 years | 7.35 | 0.03 | (7.30, 7.40) |  |
|  | 16+years | 7.83 | 0.04 | (7.76, 7.91) |  |
| Immigration status | Born in this country | 7.46 | 0.02 | (7.42, 7.51) | <0.001 |
|  | Born in another country | 7.78 | 0.08 | (7.62, 7.94) |  |
| Religious affiliation | Christianity | 7.77 | 0.03 | (7.71, 7.82) | <0.001 |
|  | Islam | 7.92 | 0.15 | (7.62, 8.22) |  |
|  | Hinduism | 7.01 | 0.81 | (5.32, 8.70) |  |
|  | Buddhism | 7.55 | 0.28 | (6.98, 8.11) |  |
|  | Judaism | 7.58 | 0.52 | (6.53, 8.62) |  |
|  | Sikhism | 8.11 | 0.70 | (5.25, 10.97) |  |
|  | Baha’i | 7.53 | 1.33 | (3.79, 11.26) |  |
|  | Taoism | 5.60 | 0.59 | (2.91, 8.28) |  |
|  | Primal, Animist, or Folk religion | 6.28 | 0.45 | (5.40, 7.17) |  |
|  | Some other religion | 6.74 | 0.28 | (6.18, 7.30) |  |
|  | No religion/Atheist/Agnostic | 7.08 | 0.03 | (7.01, 7.14) |  |

Table S19a. Nationally representative descriptive statistics for Tanzania

| **Characteristic** | **N = 9,075**^1^ |
| --- | --- |
| **Age group** |  |
| 18-24 | 2,284 (25%) |
| 25-29 | 1,349 (15%) |
| 30-39 | 2,060 (23%) |
| 40-49 | 1,503 (17%) |
| 50-59 | 912 (10%) |
| 60-69 | 575 (6.3%) |
| 70-79 | 297 (3.3%) |
| 80 or older | 93 (1.0%) |
| Missing | 2 (<0.1%) |
| **Gender** |  |
| Male | 4,299 (47%) |
| Female | 4,776 (53%) |
| Other | 0 (0%) |
| Missing | 0 (0%) |
| **Marital status** |  |
| Married | 5,577 (61%) |
| Separated | 404 (4.5%) |
| Divorced | 103 (1.1%) |
| Widowed | 450 (5.0%) |
| Never | 2,260 (25%) |
| Domestic Partner | 275 (3.0%) |
| Missing | 7 (<0.1%) |
| **Employment** |  |
| Employed for an employer | 513 (5.6%) |
| Self-employed | 4,625 (51%) |
| Retired | 139 (1.5%) |
| Student | 319 (3.5%) |
| Homemaker | 1,796 (20%) |
| Unemployed and looking for a job | 1,491 (16%) |
| None of these/other | 186 (2.1%) |
| Missing | 6 (<0.1%) |
| **Religious service attendance** |  |
| >1/week | 2,622 (29%) |
| 1/week | 4,268 (47%) |
| 1-3/month | 1,082 (12%) |
| A few times a year | 814 (9.0%) |
| Never | 288 (3.2%) |
| Missing | 1 (<0.1%) |
| **Education** |  |
| up to 8 years | 6,699 (74%) |
| 9-15 years | 2,252 (25%) |
| 16+years | 122 (1.3%) |
| Missing | 2 (<0.1%) |
| **Immigration** |  |
| Born in this country | 9,048 (100%) |
| Born in another country | 25 (0.3%) |
| Missing | 1 (<0.1%) |
| **Religious affiliation** |  |
| Christianity | 5,647 (62%) |
| Islam | 3,189 (35%) |
| Taoism | 1 (<0.1%) |
| Primal, Animist, or Folk religion | 12 (0.1%) |
| No religion/Atheist/Agnostic | 216 (2.4%) |
| Missing | 10 (0.1%) |
| **Race and ethnicity** |  |
| African | 9,060 (100%) |
| Arab | 11 (0.1%) |
| Indian | 3 (<0.1%) |
| Missing | 2 (<0.1%) |
| ^1^n (%) | |

Table S19b. Means by demographic category for Tanzania

| Variable | Category | Mean | SE | 95% CI | Global p-value |
| --- | --- | --- | --- | --- | --- |
| Age group | 18-24 | 7.95 | 0.09 | (7.78, 8.12) | 0.085 |
|  | 25-29 | 8.06 | 0.10 | (7.87, 8.26) |  |
|  | 30-39 | 7.83 | 0.11 | (7.62, 8.05) |  |
|  | 40-49 | 7.71 | 0.12 | (7.47, 7.94) |  |
|  | 50-59 | 7.75 | 0.13 | (7.50, 8.00) |  |
|  | 60-69 | 7.79 | 0.18 | (7.45, 8.14) |  |
|  | 70-79 | 7.51 | 0.34 | (6.85, 8.18) |  |
|  | 80 or older | 7.59 | 0.49 | (6.60, 8.57) |  |
| Gender | Male | 7.82 | 0.09 | (7.64, 8.00) | 0.712 |
|  | Female | 7.88 | 0.07 | (7.74, 8.02) |  |
| Marital status | Married | 7.81 | 0.09 | (7.64, 7.98) | 0.274 |
|  | Separated | 7.71 | 0.20 | (7.31, 8.11) |  |
|  | Divorced | 8.19 | 0.29 | (7.62, 8.77) |  |
|  | Widowed | 7.82 | 0.22 | (7.40, 8.24) |  |
|  | Never | 7.99 | 0.09 | (7.81, 8.17) |  |
|  | Domestic Partner | 7.64 | 0.22 | (7.22, 8.07) |  |
| Employment | Employed for an employer | 8.29 | 0.15 | (7.99, 8.59) | <0.001 |
|  | Self-employed | 7.81 | 0.09 | (7.64, 7.98) |  |
|  | Retired | 8.33 | 0.20 | (7.93, 8.73) |  |
|  | Student | 8.29 | 0.16 | (7.97, 8.61) |  |
|  | Homemaker | 7.72 | 0.12 | (7.49, 7.95) |  |
|  | Unemployed and looking for a job | 7.96 | 0.11 | (7.74, 8.17) |  |
|  | None of these/other | 7.11 | 0.40 | (6.32, 7.90) |  |
| Religious service attendance | >1/week | 8.01 | 0.09 | (7.82, 8.19) | <0.001 |
|  | 1/week | 7.86 | 0.09 | (7.69, 8.02) |  |
|  | 1-3/month | 7.66 | 0.13 | (7.40, 7.92) |  |
|  | A few times a year | 7.71 | 0.15 | (7.42, 8.01) |  |
|  | Never | 7.52 | 0.25 | (7.02, 8.02) |  |
| Education | up to 8 years | 7.74 | 0.08 | (7.58, 7.90) | <0.001 |
|  | 9-15 years | 8.17 | 0.07 | (8.03, 8.31) |  |
|  | 16+years | 8.33 | 0.23 | (7.88, 8.78) |  |
| Immigration status | Born in this country | 7.85 | 0.07 | (7.71, 7.99) | 0.969 |
|  | Born in another country | 8.12 | 0.73 | (6.62, 9.63) |  |
| Religious affiliation | Christianity | 7.85 | 0.09 | (7.68, 8.02) | <0.001 |
|  | Islam | 7.90 | 0.10 | (7.70, 8.09) |  |
|  | Primal, Animist, or Folk religion | 5.90 | 1.69 | (2.12, 9.69) |  |
|  | No religion/Atheist/Agnostic | 7.28 | 0.23 | (6.83, 7.74) |  |
| Race and ethnicity | Indian | 4.33 |  |  | 0.160 |
|  | Arab | 6.85 | 0.96 | (4.65, 9.04) |  |
|  | African | 7.85 | 0.07 | (7.72, 7.99) |  |

Table S20a. Nationally representative descriptive statistics for Türkiye

| **Characteristic** | **N = 1,473**^1^ |
| --- | --- |
| **Age group** |  |
| 18-24 | 222 (15%) |
| 25-29 | 152 (10%) |
| 30-39 | 315 (21%) |
| 40-49 | 312 (21%) |
| 50-59 | 225 (15%) |
| 60-69 | 164 (11%) |
| 70-79 | 65 (4.4%) |
| 80 or older | 18 (1.2%) |
| Missing | 0 (0%) |
| **Gender** |  |
| Male | 754 (51%) |
| Female | 719 (49%) |
| Other | 0 (0%) |
| Missing | 0 (0%) |
| **Marital status** |  |
| Married | 936 (64%) |
| Separated | 13 (0.9%) |
| Divorced | 64 (4.3%) |
| Widowed | 64 (4.3%) |
| Never | 379 (26%) |
| Domestic Partner | 0 (0%) |
| Missing | 17 (1.1%) |
| **Employment** |  |
| Employed for an employer | 413 (28%) |
| Self-employed | 255 (17%) |
| Retired | 205 (14%) |
| Student | 107 (7.3%) |
| Homemaker | 347 (24%) |
| Unemployed and looking for a job | 87 (5.9%) |
| None of these/other | 59 (4.0%) |
| Missing | 0 (0%) |
| **Religious service attendance** |  |
| >1/week | 493 (33%) |
| 1/week | 271 (18%) |
| 1-3/month | 174 (12%) |
| A few times a year | 255 (17%) |
| Never | 274 (19%) |
| Missing | 6 (0.4%) |
| **Education** |  |
| up to 8 years | 436 (30%) |
| 9-15 years | 711 (48%) |
| 16+years | 326 (22%) |
| Missing | 0 (0%) |
| **Immigration** |  |
| Born in this country | 1,415 (96%) |
| Born in another country | 58 (4.0%) |
| Missing | 0 (0%) |
| **Religious affiliation** |  |
| Christianity | 2 (0.1%) |
| Islam | 1,381 (94%) |
| Buddhism | 1 (<0.1%) |
| Judaism | 1 (<0.1%) |
| Sikhism | 1 (<0.1%) |
| Primal, Animist, or Folk religion | 1 (<0.1%) |
| Some other religion | 1 (<0.1%) |
| No religion/Atheist/Agnostic | 66 (4.5%) |
| Missing | 19 (1.3%) |
| **Race and ethnicity** |  |
| Albanian | 8 (0.5%) |
| Arab | 51 (3.5%) |
| Armenian | 1 (<0.1%) |
| Azeri | 9 (0.6%) |
| Bosnian | 5 (0.3%) |
| Circassian | 19 (1.3%) |
| Georgian | 4 (0.3%) |
| Greek | 1 (<0.1%) |
| Kurdish/Zaza | 252 (17%) |
| Laz | 25 (1.7%) |
| Other | 58 (3.9%) |
| Turkish | 1,030 (70%) |
| Uyghur | 1 (<0.1%) |
| Missing | 9 (0.6%) |
| ^1^n (%) | |

Table S20b. Means by demographic category for Türkiye

| Variable | Category | Mean | SE | 95% CI | Global p-value |
| --- | --- | --- | --- | --- | --- |
| Age group | 18-24 | 6.58 | 0.19 | (6.21, 6.95) | 0.008 |
|  | 25-29 | 7.19 | 0.26 | (6.68, 7.70) |  |
|  | 30-39 | 7.02 | 0.18 | (6.67, 7.37) |  |
|  | 40-49 | 7.04 | 0.17 | (6.69, 7.38) |  |
|  | 50-59 | 6.96 | 0.25 | (6.47, 7.44) |  |
|  | 60-69 | 6.84 | 0.34 | (6.16, 7.53) |  |
|  | 70-79 | 6.97 | 0.61 | (5.75, 8.20) |  |
|  | 80 or older | 8.80 | 0.84 | (6.95, 10.65) |  |
| Gender | Male | 6.86 | 0.11 | (6.64, 7.07) | 0.109 |
|  | Female | 7.08 | 0.14 | (6.80, 7.35) |  |
| Marital status | Married | 7.00 | 0.12 | (6.77, 7.24) | 0.560 |
|  | Separated | 6.73 | 0.94 | (4.44, 9.02) |  |
|  | Divorced | 6.95 | 0.38 | (6.20, 7.71) |  |
|  | Widowed | 7.61 | 0.57 | (6.45, 8.76) |  |
|  | Never | 6.77 | 0.14 | (6.49, 7.05) |  |
| Employment | Employed for an employer | 7.05 | 0.15 | (6.76, 7.34) | <0.001 |
|  | Self-employed | 6.65 | 0.21 | (6.24, 7.06) |  |
|  | Retired | 7.25 | 0.23 | (6.79, 7.70) |  |
|  | Student | 6.48 | 0.26 | (5.97, 6.99) |  |
|  | Homemaker | 7.16 | 0.24 | (6.69, 7.62) |  |
|  | Unemployed and looking for a job | 7.12 | 0.33 | (6.45, 7.78) |  |
|  | None of these/other | 6.31 | 0.33 | (5.66, 6.96) |  |
| Religious service attendance | >1/week | 7.35 | 0.16 | (7.05, 7.66) | <0.001 |
|  | 1/week | 7.19 | 0.21 | (6.78, 7.60) |  |
|  | 1-3/month | 6.89 | 0.26 | (6.39, 7.40) |  |
|  | A few times a year | 6.61 | 0.19 | (6.24, 6.98) |  |
|  | Never | 6.42 | 0.23 | (5.96, 6.87) |  |
| Education | up to 8 years | 7.31 | 0.21 | (6.89, 7.73) | <0.001 |
|  | 9-15 years | 6.96 | 0.12 | (6.72, 7.21) |  |
|  | 16+years | 6.51 | 0.12 | (6.27, 6.74) |  |
| Immigration status | Born in this country | 6.96 | 0.09 | (6.77, 7.14) | 0.935 |
|  | Born in another country | 7.15 | 0.42 | (6.30, 8.00) |  |
| Religious affiliation | Christianity | 5.37 |  |  | <0.001 |
|  | Islam | 7.02 | 0.10 | (6.83, 7.20) |  |
|  | Primal, Animist, or Folk religion | 5.66 |  |  |  |
|  | Some other religion | 8.62 |  |  |  |
|  | No religion/Atheist/Agnostic | 5.93 | 0.33 | (5.27, 6.59) |  |
| Race and ethnicity | Other | 7.42 | 0.39 | (6.63, 8.21) | <0.001 |
|  | Arab | 7.69 | 0.40 | (6.88, 8.49) |  |
|  | Greek | 6.71 |  |  |  |
|  | Turkish | 6.87 | 0.10 | (6.67, 7.08) |  |
|  | Albanian | 5.18 | 1.78 | (0.66, 9.71) |  |
|  | Armenian | 5.43 |  |  |  |
|  | Azeri | 8.50 | 0.77 | (6.65, 10.35) |  |
|  | Bosnian | 7.22 | 0.52 | (5.44, 9.00) |  |
|  | Circassian | 5.94 | 1.08 | (3.57, 8.32) |  |
|  | Georgian | 6.26 | 1.32 | (0.81, 11.70) |  |
|  | Kurdish/Zaza | 7.19 | 0.23 | (6.73, 7.64) |  |
|  | Laz | 7.21 | 0.56 | (6.04, 8.38) |  |

Table S21a. Nationally representative descriptive statistics for United Kingdom

| **Characteristic** | **N = 5,368**^1^ |
| --- | --- |
| **Age group** |  |
| 18-24 | 490 (9.1%) |
| 25-29 | 391 (7.3%) |
| 30-39 | 946 (18%) |
| 40-49 | 827 (15%) |
| 50-59 | 949 (18%) |
| 60-69 | 889 (17%) |
| 70-79 | 711 (13%) |
| 80 or older | 163 (3.0%) |
| Missing | 1 (<0.1%) |
| **Gender** |  |
| Male | 2,557 (48%) |
| Female | 2,789 (52%) |
| Other | 14 (0.3%) |
| Missing | 9 (0.2%) |
| **Marital status** |  |
| Married | 2,510 (47%) |
| Separated | 114 (2.1%) |
| Divorced | 435 (8.1%) |
| Widowed | 294 (5.5%) |
| Never | 1,456 (27%) |
| Domestic Partner | 512 (9.5%) |
| Missing | 48 (0.9%) |
| **Employment** |  |
| Employed for an employer | 2,798 (52%) |
| Self-employed | 469 (8.7%) |
| Retired | 1,262 (24%) |
| Student | 229 (4.3%) |
| Homemaker | 184 (3.4%) |
| Unemployed and looking for a job | 215 (4.0%) |
| None of these/other | 201 (3.7%) |
| Missing | 11 (0.2%) |
| **Religious service attendance** |  |
| >1/week | 291 (5.4%) |
| 1/week | 499 (9.3%) |
| 1-3/month | 293 (5.5%) |
| A few times a year | 1,165 (22%) |
| Never | 3,110 (58%) |
| Missing | 10 (0.2%) |
| **Education** |  |
| up to 8 years | 1,314 (24%) |
| 9-15 years | 2,072 (39%) |
| 16+years | 1,974 (37%) |
| Missing | 8 (0.2%) |
| **Immigration** |  |
| Born in this country | 4,659 (87%) |
| Born in another country | 682 (13%) |
| Missing | 27 (0.5%) |
| **Religious affiliation** |  |
| Christianity | 2,750 (51%) |
| Islam | 218 (4.1%) |
| Hinduism | 61 (1.1%) |
| Buddhism | 30 (0.6%) |
| Judaism | 44 (0.8%) |
| Sikhism | 29 (0.5%) |
| Baha’i | 6 (0.1%) |
| Jainism | 4 (<0.1%) |
| Taoism | 4 (<0.1%) |
| Confucianism | 2 (<0.1%) |
| Primal, Animist, or Folk religion | 36 (0.7%) |
| Some other religion | 61 (1.1%) |
| No religion/Atheist/Agnostic | 2,099 (39%) |
| Missing | 25 (0.5%) |
| **Race and ethnicity** |  |
| Asian | 426 (7.9%) |
| Black | 152 (2.8%) |
| Other | 96 (1.8%) |
| White | 4,647 (87%) |
| Missing | 47 (0.9%) |
| ^1^n (%) | |

Table S21b. Means by demographic category for United Kingdom

| Variable | Category | Mean | SE | 95% CI | Global p-value |
| --- | --- | --- | --- | --- | --- |
| Age group | 18-24 | 7.22 | 0.17 | (6.88, 7.55) | <0.001 |
|  | 25-29 | 7.65 | 0.16 | (7.34, 7.96) |  |
|  | 30-39 | 7.45 | 0.11 | (7.24, 7.66) |  |
|  | 40-49 | 7.24 | 0.12 | (7.01, 7.47) |  |
|  | 50-59 | 7.25 | 0.10 | (7.05, 7.45) |  |
|  | 60-69 | 7.38 | 0.11 | (7.17, 7.60) |  |
|  | 70-79 | 7.67 | 0.13 | (7.41, 7.92) |  |
|  | 80 or older | 7.88 | 0.29 | (7.30, 8.46) |  |
| Gender | Male | 7.36 | 0.07 | (7.23, 7.49) | 0.015 |
|  | Female | 7.46 | 0.06 | (7.33, 7.58) |  |
|  | Other | 5.80 | 0.89 | (3.84, 7.76) |  |
| Marital status | Married | 7.82 | 0.06 | (7.71, 7.93) | <0.001 |
|  | Separated | 6.69 | 0.27 | (6.15, 7.22) |  |
|  | Divorced | 6.88 | 0.16 | (6.56, 7.20) |  |
|  | Widowed | 7.50 | 0.24 | (7.02, 7.97) |  |
|  | Never | 6.87 | 0.10 | (6.68, 7.07) |  |
|  | Domestic Partner | 7.44 | 0.14 | (7.16, 7.71) |  |
| Employment | Employed for an employer | 7.44 | 0.06 | (7.33, 7.56) | <0.001 |
|  | Self-employed | 7.58 | 0.14 | (7.30, 7.86) |  |
|  | Retired | 7.70 | 0.09 | (7.51, 7.88) |  |
|  | Student | 7.47 | 0.21 | (7.05, 7.88) |  |
|  | Homemaker | 7.48 | 0.31 | (6.86, 8.09) |  |
|  | Unemployed and looking for a job | 6.50 | 0.27 | (5.98, 7.02) |  |
|  | None of these/other | 5.48 | 0.26 | (4.96, 5.99) |  |
| Religious service attendance | >1/week | 8.87 | 0.11 | (8.66, 9.08) | <0.001 |
|  | 1/week | 8.23 | 0.12 | (8.00, 8.47) |  |
|  | 1-3/month | 7.97 | 0.17 | (7.63, 8.30) |  |
|  | A few times a year | 7.77 | 0.09 | (7.60, 7.94) |  |
|  | Never | 6.95 | 0.06 | (6.82, 7.07) |  |
| Education | up to 8 years | 7.13 | 0.13 | (6.87, 7.39) | <0.001 |
|  | 9-15 years | 7.25 | 0.06 | (7.13, 7.38) |  |
|  | 16+years | 7.75 | 0.06 | (7.64, 7.87) |  |
| Immigration status | Born in this country | 7.37 | 0.05 | (7.27, 7.46) | <0.001 |
|  | Born in another country | 7.69 | 0.12 | (7.44, 7.93) |  |
| Religious affiliation | Christianity | 7.81 | 0.06 | (7.70, 7.92) | <0.001 |
|  | Islam | 8.36 | 0.17 | (8.03, 8.69) |  |
|  | Hinduism | 7.22 | 0.52 | (6.18, 8.26) |  |
|  | Buddhism | 8.24 | 0.32 | (7.58, 8.90) |  |
|  | Judaism | 6.95 | 0.61 | (5.71, 8.20) |  |
|  | Sikhism | 7.41 | 0.35 | (6.69, 8.13) |  |
|  | Baha’i | 1.99 | 1.84 | (-3.57, 7.56) |  |
|  | Taoism | 9.24 | 0.59 | (6.43, 12.04) |  |
|  | Primal, Animist, or Folk religion | 6.51 | 0.48 | (5.54, 7.48) |  |
|  | Some other religion | 7.11 | 0.29 | (6.52, 7.70) |  |
|  | No religion/Atheist/Agnostic | 6.82 | 0.08 | (6.67, 6.97) |  |
| Race and ethnicity | Asian | 7.72 | 0.16 | (7.41, 8.04) | <0.001 |
|  | Black | 8.22 | 0.21 | (7.80, 8.64) |  |
|  | Other | 7.45 | 0.38 | (6.71, 8.20) |  |
|  | White | 7.35 | 0.05 | (7.25, 7.45) |  |

Table S22a. Nationally representative descriptive statistics for United States

| **Characteristic** | **N = 38,312**^1^ |
| --- | --- |
| **Age group** |  |
| 18-24 | 2,682 (7.0%) |
| 25-29 | 3,540 (9.2%) |
| 30-39 | 7,284 (19%) |
| 40-49 | 5,649 (15%) |
| 50-59 | 6,745 (18%) |
| 60-69 | 6,832 (18%) |
| 70-79 | 4,054 (11%) |
| 80 or older | 1,525 (4.0%) |
| Missing | 0 (0%) |
| **Gender** |  |
| Male | 18,222 (48%) |
| Female | 19,562 (51%) |
| Other | 392 (1.0%) |
| Missing | 136 (0.4%) |
| **Marital status** |  |
| Married | 20,360 (53%) |
| Separated | 727 (1.9%) |
| Divorced | 3,636 (9.5%) |
| Widowed | 1,978 (5.2%) |
| Never | 9,431 (25%) |
| Domestic Partner | 1,971 (5.1%) |
| Missing | 207 (0.5%) |
| **Employment** |  |
| Employed for an employer | 19,502 (51%) |
| Self-employed | 3,445 (9.0%) |
| Retired | 9,016 (24%) |
| Student | 1,145 (3.0%) |
| Homemaker | 2,049 (5.3%) |
| Unemployed and looking for a job | 1,777 (4.6%) |
| None of these/other | 1,292 (3.4%) |
| Missing | 87 (0.2%) |
| **Religious service attendance** |  |
| >1/week | 2,633 (6.9%) |
| 1/week | 5,887 (15%) |
| 1-3/month | 2,819 (7.4%) |
| A few times a year | 8,870 (23%) |
| Never | 17,975 (47%) |
| Missing | 128 (0.3%) |
| **Education** |  |
| up to 8 years | 210 (0.5%) |
| 9-15 years | 25,322 (66%) |
| 16+years | 12,705 (33%) |
| Missing | 75 (0.2%) |
| **Immigration** |  |
| Born in this country | 34,865 (91%) |
| Born in another country | 3,020 (7.9%) |
| Missing | 427 (1.1%) |
| **Religious affiliation** |  |
| Christianity | 22,954 (60%) |
| Islam | 205 (0.5%) |
| Hinduism | 167 (0.4%) |
| Buddhism | 336 (0.9%) |
| Judaism | 638 (1.7%) |
| Sikhism | 24 (<0.1%) |
| Baha’i | 13 (<0.1%) |
| Jainism | 18 (<0.1%) |
| Shinto | 12 (<0.1%) |
| Taoism | 93 (0.2%) |
| Confucianism | 8 (<0.1%) |
| Primal, Animist, or Folk religion | 240 (0.6%) |
| Some other religion | 1,267 (3.3%) |
| No religion/Atheist/Agnostic | 11,870 (31%) |
| Missing | 467 (1.2%) |
| **Race and ethnicity** |  |
| Asian | 2,466 (6.4%) |
| Black | 4,501 (12%) |
| Hispanic | 6,724 (18%) |
| Other | 997 (2.6%) |
| White | 23,605 (62%) |
| Missing | 20 (<0.1%) |
| ^1^n (%) | |

Table S22b. Means by demographic category for United States

| Variable | Category | Mean | SE | 95% CI | Global p-value |
| --- | --- | --- | --- | --- | --- |
| Age group | 18-24 | 7.38 | 0.23 | (6.93, 7.83) | <0.001 |
|  | 25-29 | 7.46 | 0.15 | (7.16, 7.75) |  |
|  | 30-39 | 7.76 | 0.08 | (7.60, 7.92) |  |
|  | 40-49 | 7.97 | 0.07 | (7.84, 8.11) |  |
|  | 50-59 | 8.31 | 0.04 | (8.22, 8.40) |  |
|  | 60-69 | 8.60 | 0.03 | (8.54, 8.66) |  |
|  | 70-79 | 8.80 | 0.04 | (8.72, 8.87) |  |
|  | 80 or older | 8.98 | 0.08 | (8.83, 9.13) |  |
| Gender | Male | 7.96 | 0.05 | (7.87, 8.05) | <0.001 |
|  | Female | 8.34 | 0.04 | (8.26, 8.42) |  |
|  | Other | 6.59 | 0.44 | (5.73, 7.44) |  |
| Marital status | Married | 8.55 | 0.03 | (8.50, 8.60) | <0.001 |
|  | Separated | 7.53 | 0.35 | (6.84, 8.23) |  |
|  | Divorced | 8.17 | 0.06 | (8.05, 8.29) |  |
|  | Widowed | 8.68 | 0.07 | (8.56, 8.81) |  |
|  | Never | 7.31 | 0.09 | (7.13, 7.49) |  |
|  | Domestic Partner | 7.53 | 0.15 | (7.23, 7.82) |  |
| Employment | Employed for an employer | 8.05 | 0.04 | (7.98, 8.12) | <0.001 |
|  | Self-employed | 8.44 | 0.09 | (8.27, 8.61) |  |
|  | Retired | 8.70 | 0.03 | (8.63, 8.77) |  |
|  | Student | 7.11 | 0.31 | (6.50, 7.72) |  |
|  | Homemaker | 8.23 | 0.13 | (7.97, 8.48) |  |
|  | Unemployed and looking for a job | 6.82 | 0.30 | (6.23, 7.40) |  |
|  | None of these/other | 7.44 | 0.25 | (6.94, 7.93) |  |
| Religious service attendance | >1/week | 9.37 | 0.06 | (9.24, 9.49) | <0.001 |
|  | 1/week | 8.97 | 0.05 | (8.88, 9.05) |  |
|  | 1-3/month | 8.70 | 0.09 | (8.53, 8.88) |  |
|  | A few times a year | 8.34 | 0.05 | (8.25, 8.44) |  |
|  | Never | 7.50 | 0.05 | (7.40, 7.61) |  |
| Education | up to 8 years | 7.88 | 0.45 | (6.99, 8.77) | <0.001 |
|  | 9-15 years | 7.98 | 0.05 | (7.89, 8.07) |  |
|  | 16+years | 8.47 | 0.02 | (8.43, 8.52) |  |
| Immigration status | Born in this country | 8.13 | 0.03 | (8.07, 8.19) | 0.321 |
|  | Born in another country | 8.26 | 0.13 | (8.01, 8.52) |  |
| Religious affiliation | Christianity | 8.58 | 0.03 | (8.52, 8.64) | <0.001 |
|  | Islam | 7.76 | 0.48 | (6.81, 8.71) |  |
|  | Hinduism | 8.34 | 0.26 | (7.82, 8.86) |  |
|  | Buddhism | 7.85 | 0.30 | (7.25, 8.44) |  |
|  | Judaism | 8.54 | 0.10 | (8.35, 8.72) |  |
|  | Sikhism | 8.18 | 0.52 | (7.11, 9.25) |  |
|  | Baha’i | 8.55 | 0.75 | (6.89, 10.21) |  |
|  | Jainism | 7.05 | 0.76 | (5.44, 8.66) |  |
|  | Shinto | 8.95 | 0.59 | (7.61, 10.28) |  |
|  | Taoism | 7.00 | 1.38 | (4.25, 9.74) |  |
|  | Confucianism | 8.15 | 0.52 | (6.89, 9.41) |  |
|  | Primal, Animist, or Folk religion | 7.41 | 0.68 | (6.07, 8.75) |  |
|  | Some other religion | 7.68 | 0.20 | (7.29, 8.06) |  |
|  | No religion/Atheist/Agnostic | 7.36 | 0.07 | (7.23, 7.48) |  |
| Race and ethnicity | Asian | 7.97 | 0.13 | (7.72, 8.22) | <0.001 |
|  | Black | 8.20 | 0.11 | (7.99, 8.41) |  |
|  | Other | 8.29 | 0.11 | (8.08, 8.51) |  |
|  | White | 8.22 | 0.03 | (8.17, 8.27) |  |
|  | Hispanic | 7.88 | 0.12 | (7.64, 8.11) |  |

Table S23. Population weighted meta-analysis of results demographic group means.

| Variable | Category | Est | 95% CI | SE |
| --- | --- | --- | --- | --- |
| Age group |  |  |  |  |
|  | 18-24 | 7.80 | (7.71,7.88) | 0.043 |
|  | 25-29 | 7.86 | (7.77,7.94) | 0.044 |
|  | 30-39 | 7.86 | (7.80,7.92) | 0.032 |
|  | 40-49 | 7.91 | (7.85,7.98) | 0.033 |
|  | 50-59 | 7.95 | (7.87,8.03) | 0.041 |
|  | 60-69 | 7.85 | (7.74,7.96) | 0.057 |
|  | 70-79 | 7.85 | (7.65,8.05) | 0.100 |
|  | 80 or older | 7.99 | (7.50,8.48) | 0.249 |
| Gender |  |  |  |  |
|  | Male | 7.80 | (7.76,7.85) | 0.022 |
|  | Female | 8.03 | (7.98,8.08) | 0.025 |
|  | Other | 6.93 | (6.48,7.38) | 0.229 |
| Marital status |  |  |  |  |
|  | Married | 8.03 | (7.99,8.07) | 0.021 |
|  | Separated | 7.41 | (6.88,7.93) | 0.270 |
|  | Divorced | 7.56 | (6.81,8.32) | 0.384 |
|  | Widowed | 7.98 | (7.81,8.15) | 0.087 |
|  | Domestic partner | 7.99 | (7.72,8.25) | 0.134 |
|  | Single, never married | 7.72 | (7.65,7.80) | 0.037 |
| Employment status |  |  |  |  |
|  | Employed for an employer | 7.91 | (7.84,7.98) | 0.036 |
|  | Self-employed | 7.98 | (7.92,8.05) | 0.035 |
|  | Retired | 7.94 | (7.69,8.18) | 0.124 |
|  | Student | 7.91 | (7.78,8.04) | 0.067 |
|  | Homemaker | 7.98 | (7.91,8.04) | 0.033 |
|  | Unemployed and looking for a job | 7.46 | (7.32,7.59) | 0.070 |
|  | None of these/other | 7.67 | (7.53,7.80) | 0.069 |
| Education |  |  |  |  |
|  | Up to 8 years | 7.83 | (7.72,7.93) | 0.054 |
|  | 9-15 years | 8.02 | (7.95,8.10) | 0.036 |
|  | 16+ years | 8.20 | (8.05,8.36) | 0.080 |
| Religious service attendance |  |  |  |  |
|  | >1/week | 8.39 | (8.33,8.45) | 0.031 |
|  | 1/week | 8.15 | (8.08,8.21) | 0.033 |
|  | 1-3/month | 8.00 | (7.93,8.07) | 0.035 |
|  | A few times a year | 7.82 | (7.75,7.90) | 0.038 |
|  | Never | 7.60 | (7.50,7.69) | 0.049 |
| Immigration status |  |  |  |  |
|  | Born in this country | 7.91 | (7.87,7.95) | 0.019 |
|  | Born in another country | 7.88 | (7.62,8.13) | 0.130 |


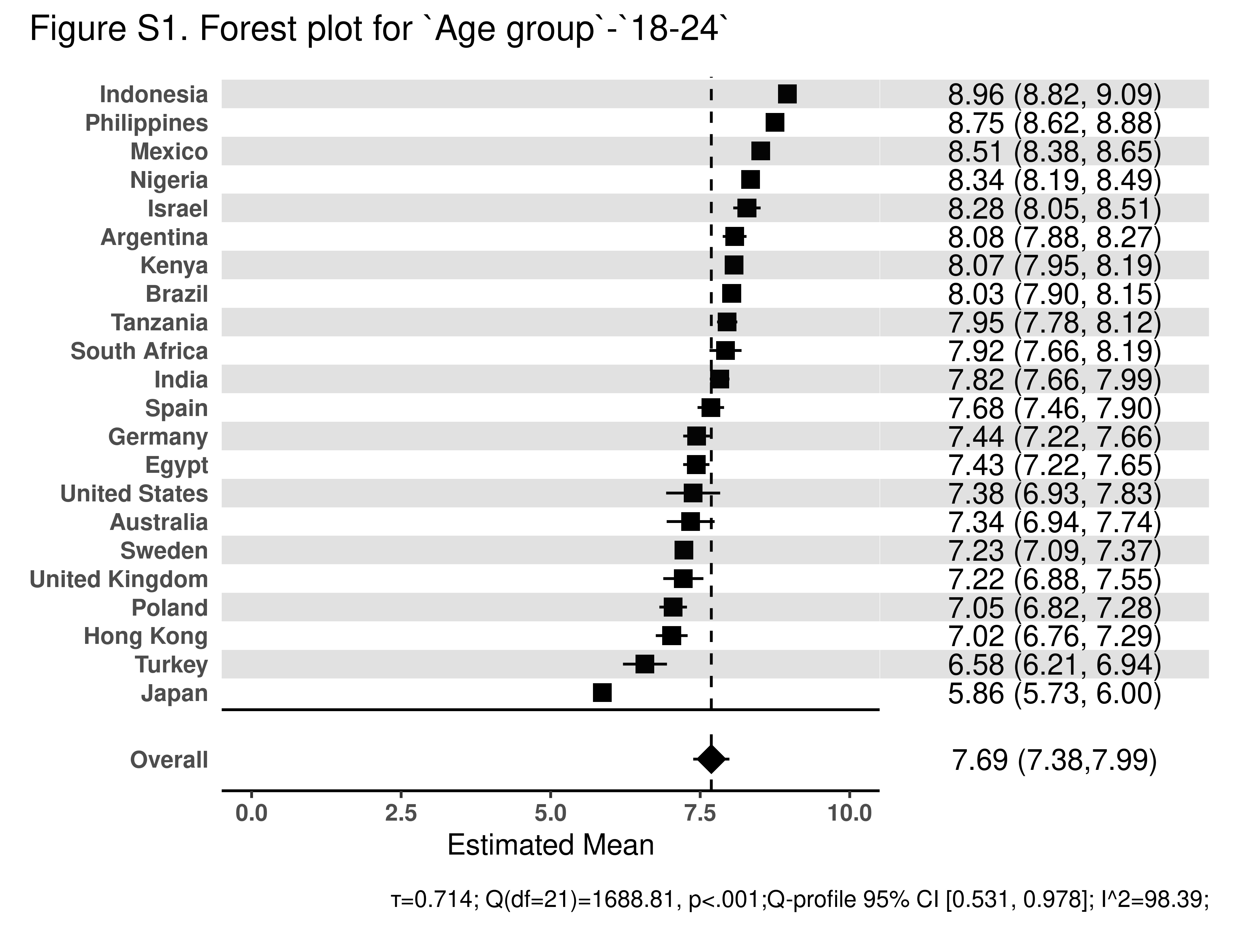

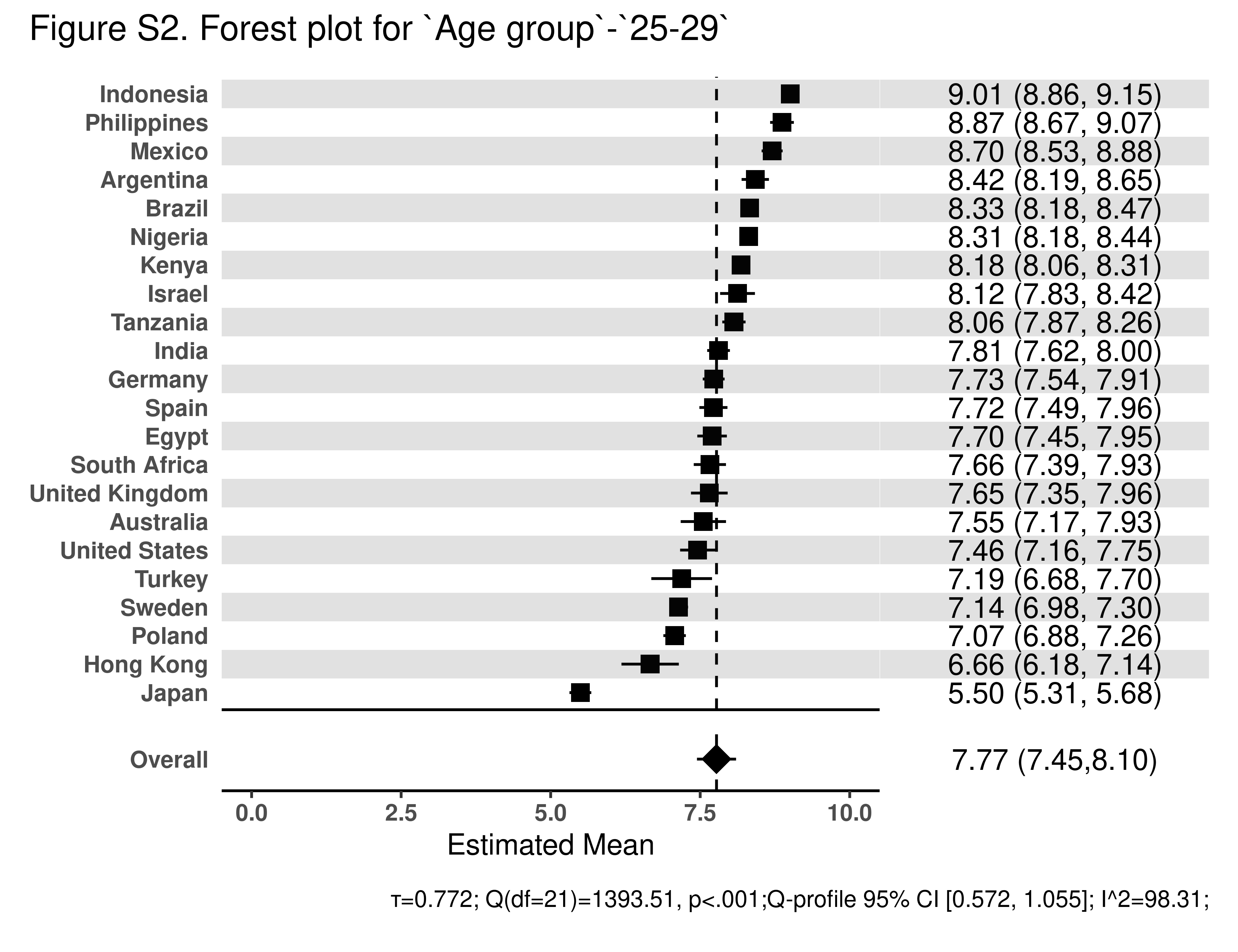

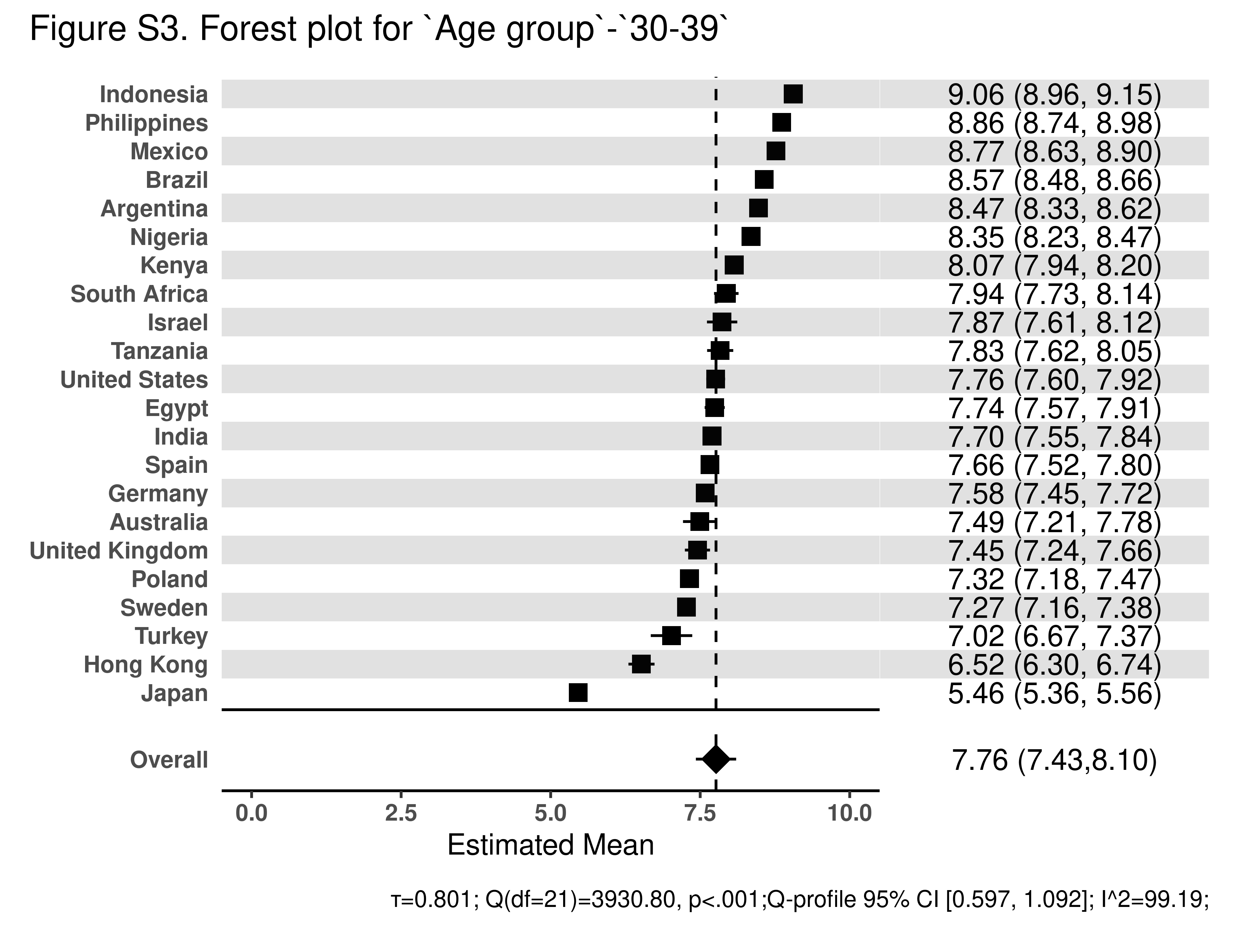

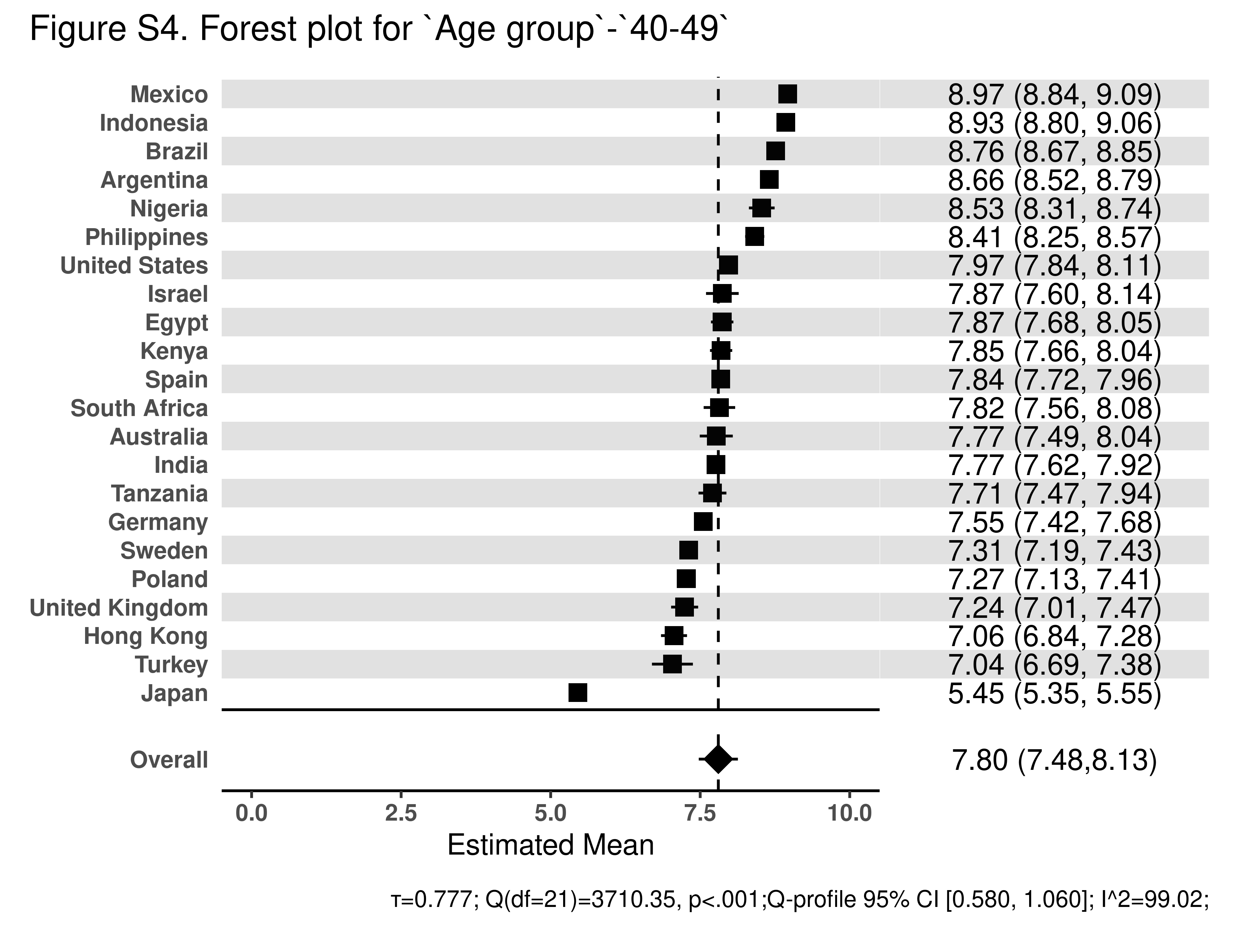

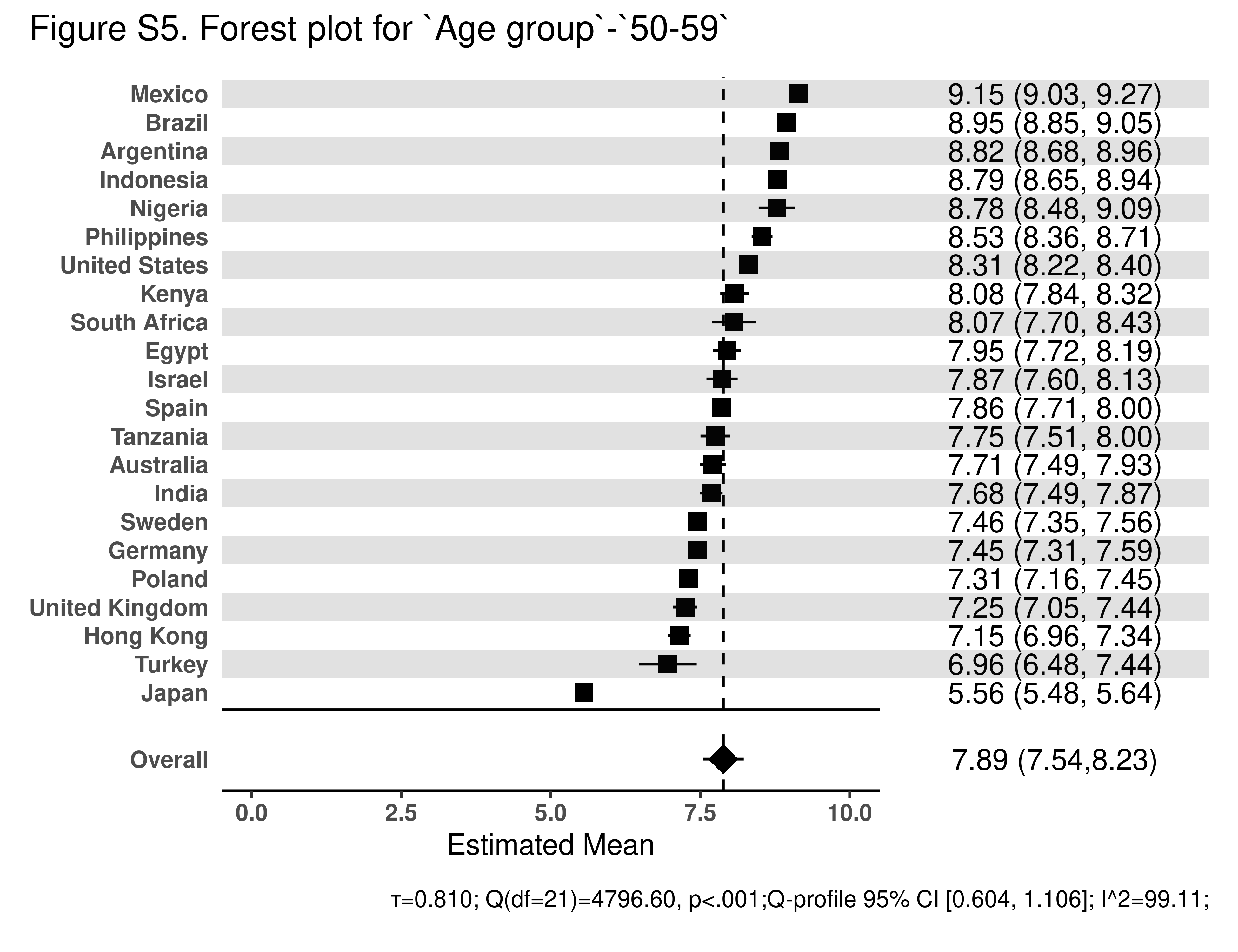

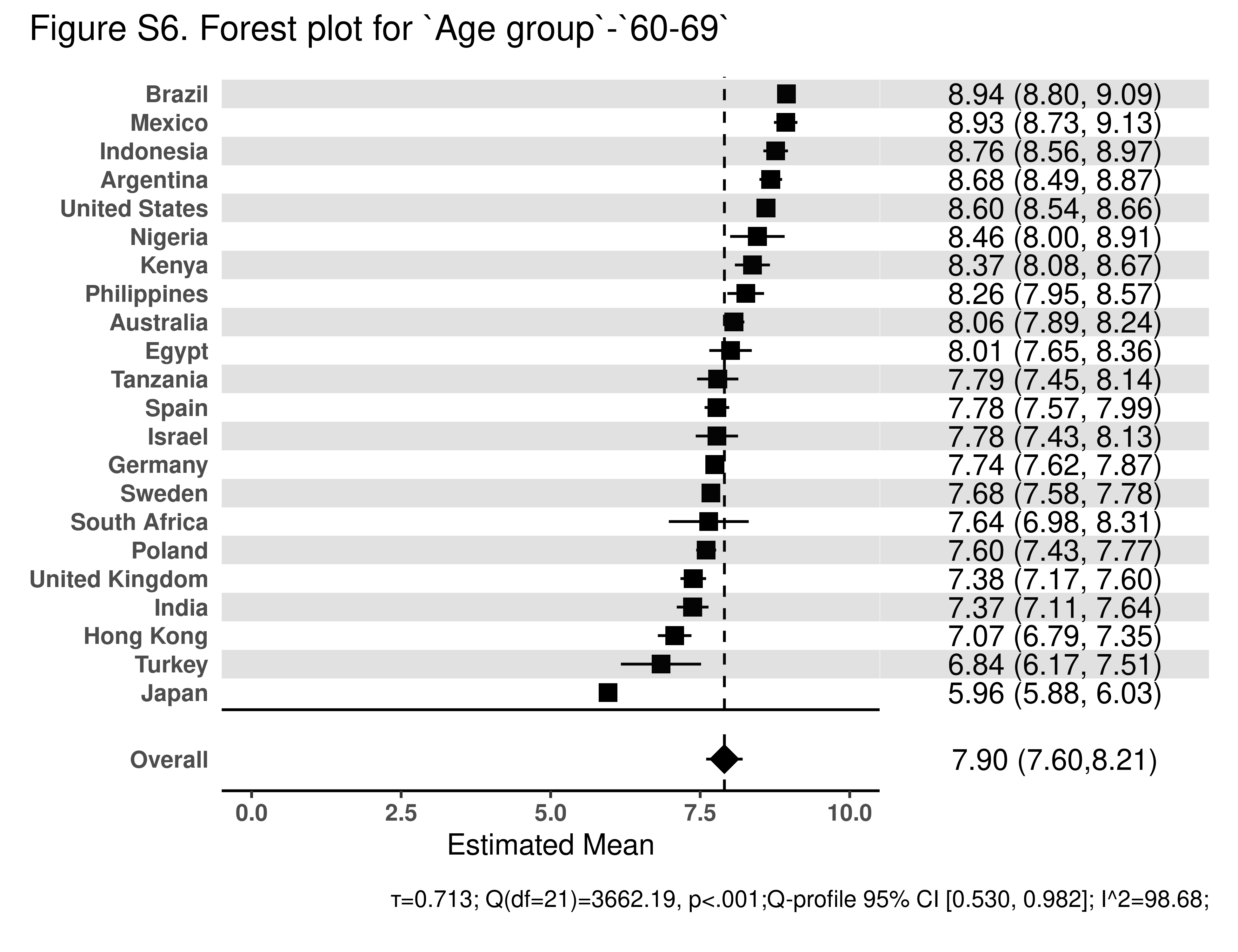

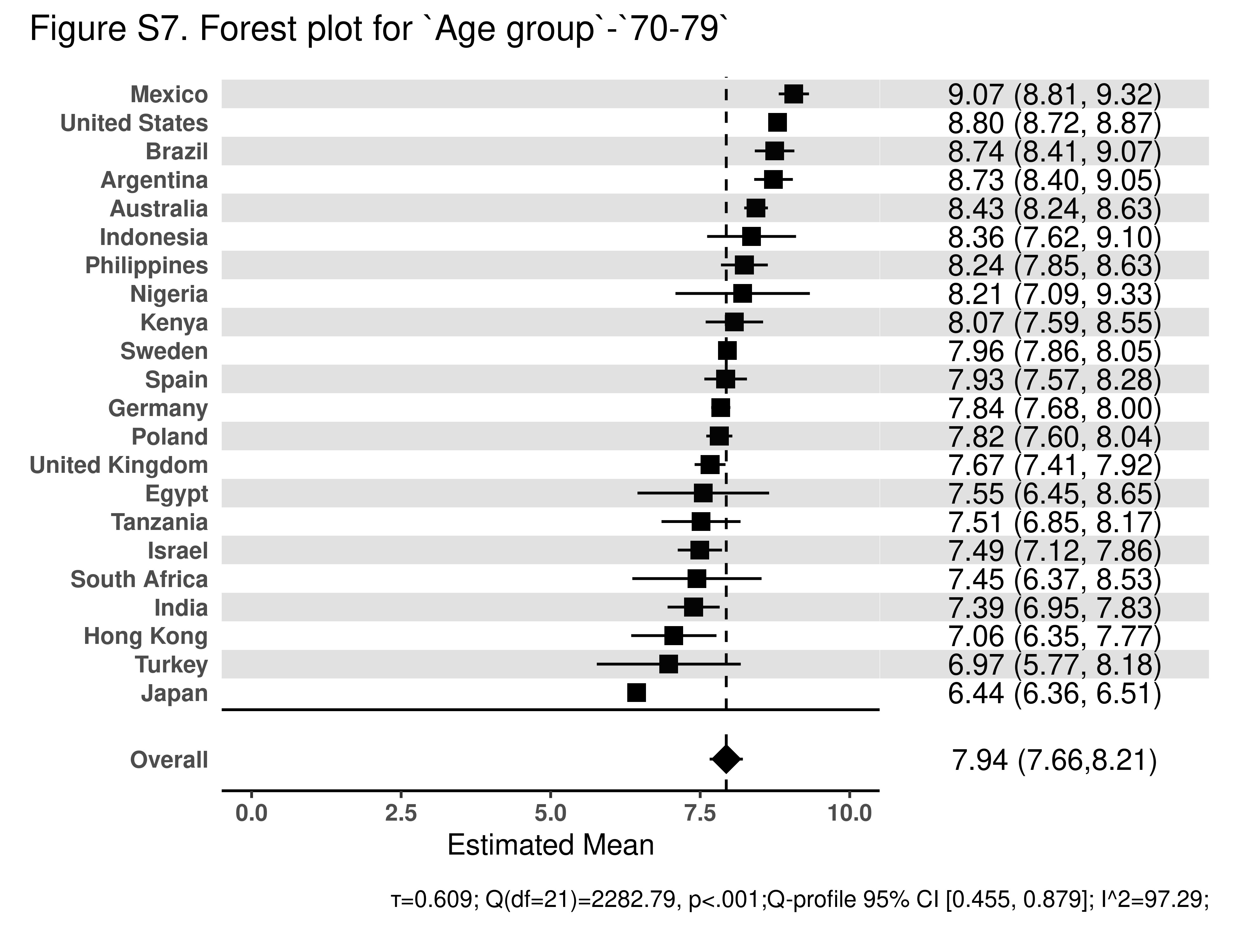

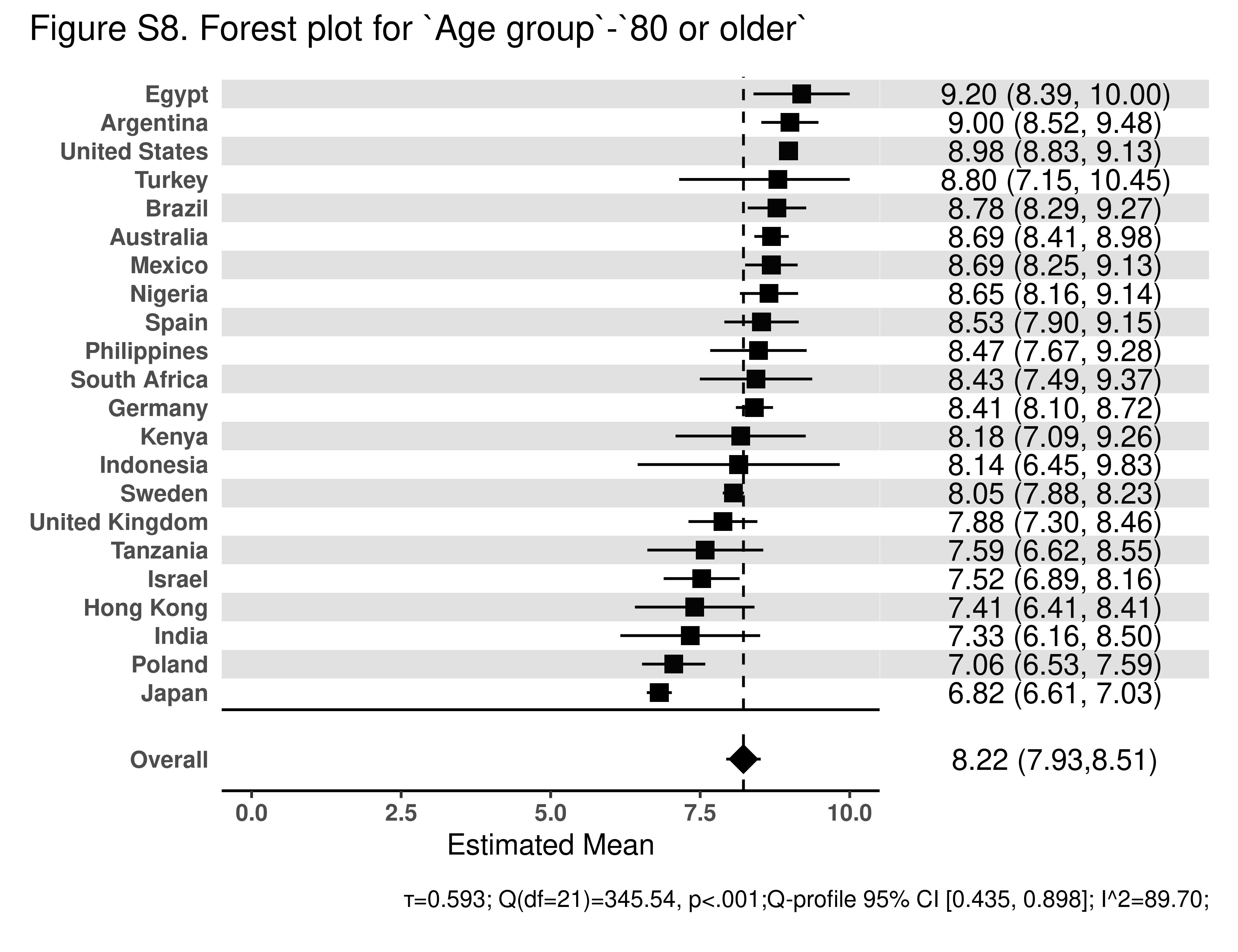

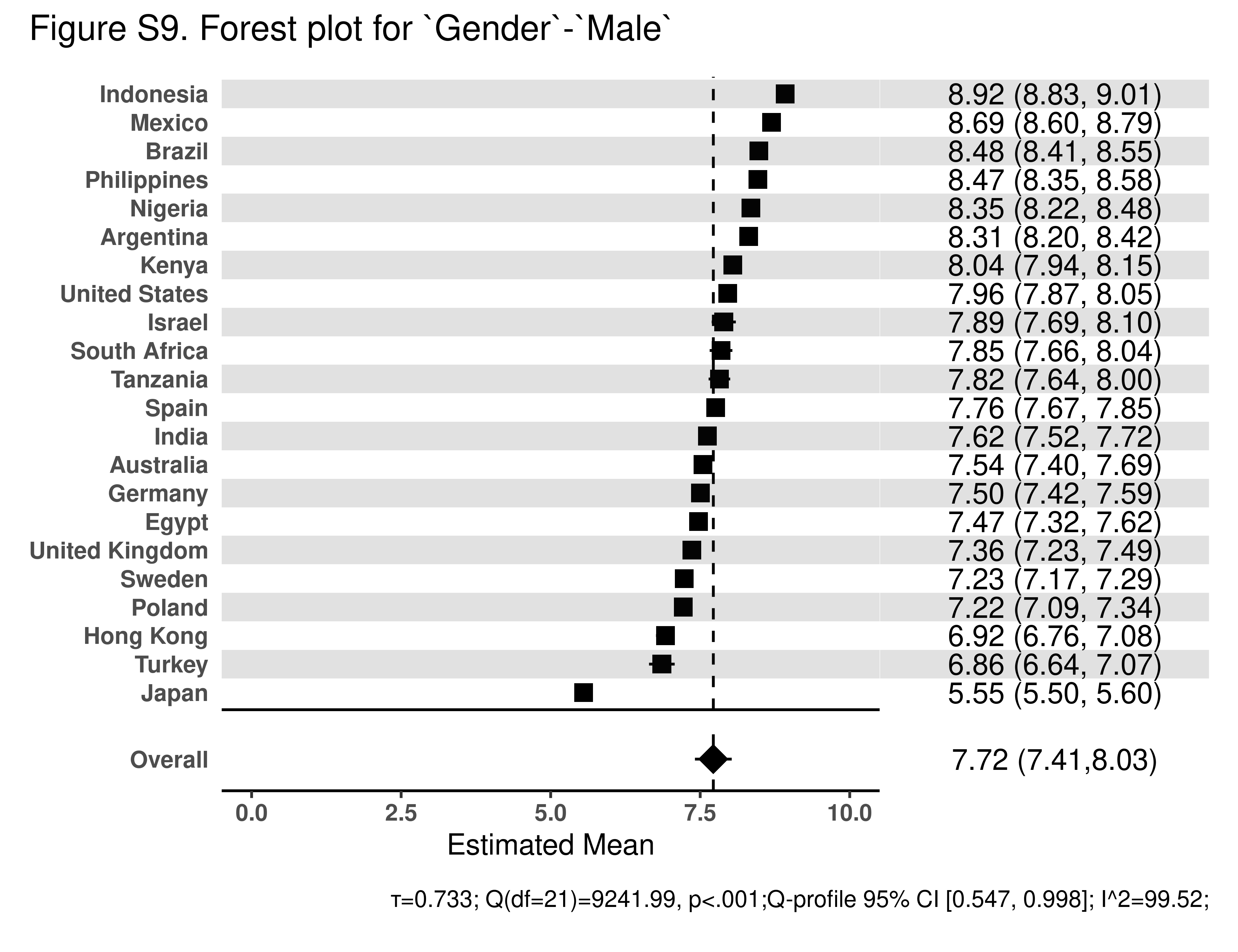

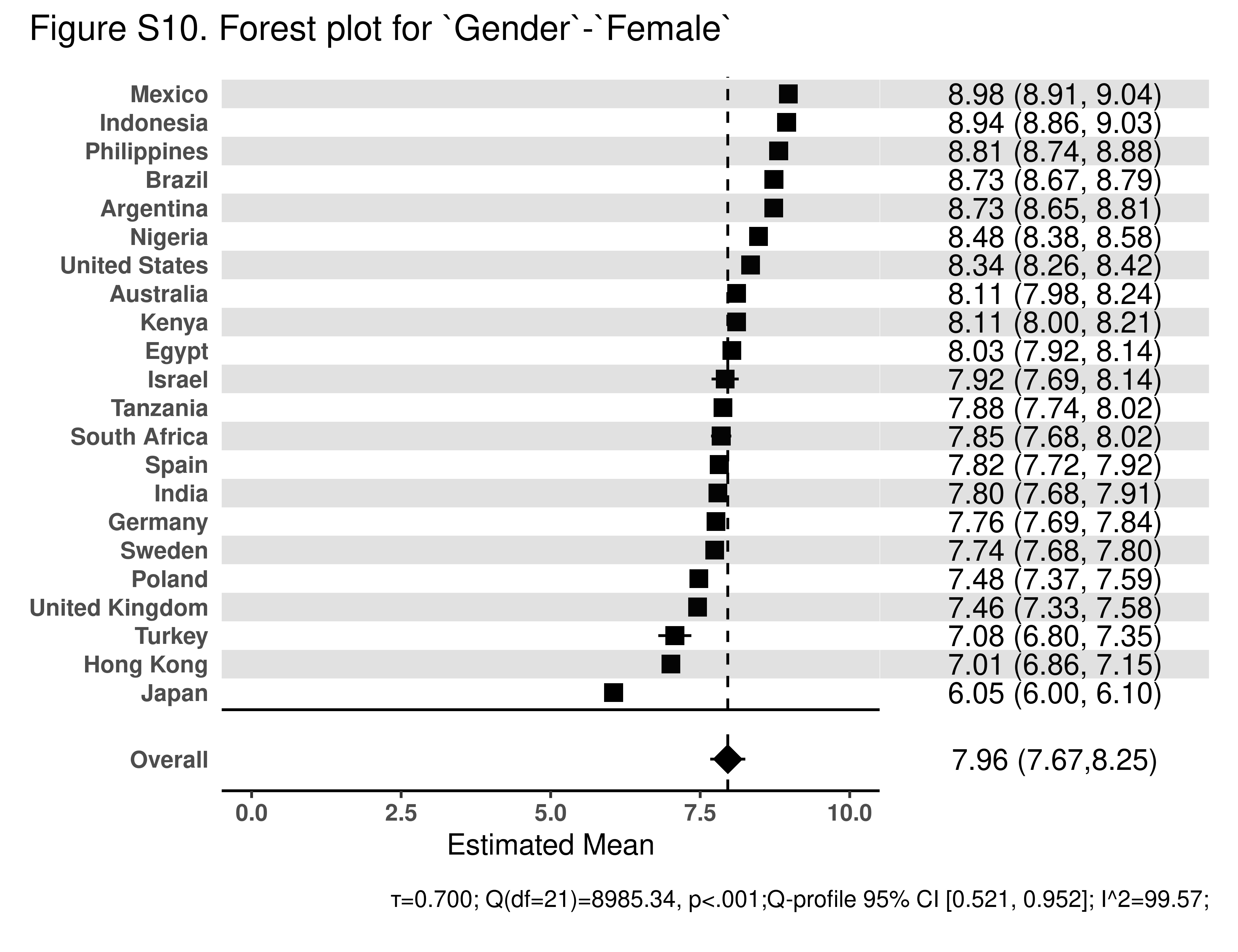

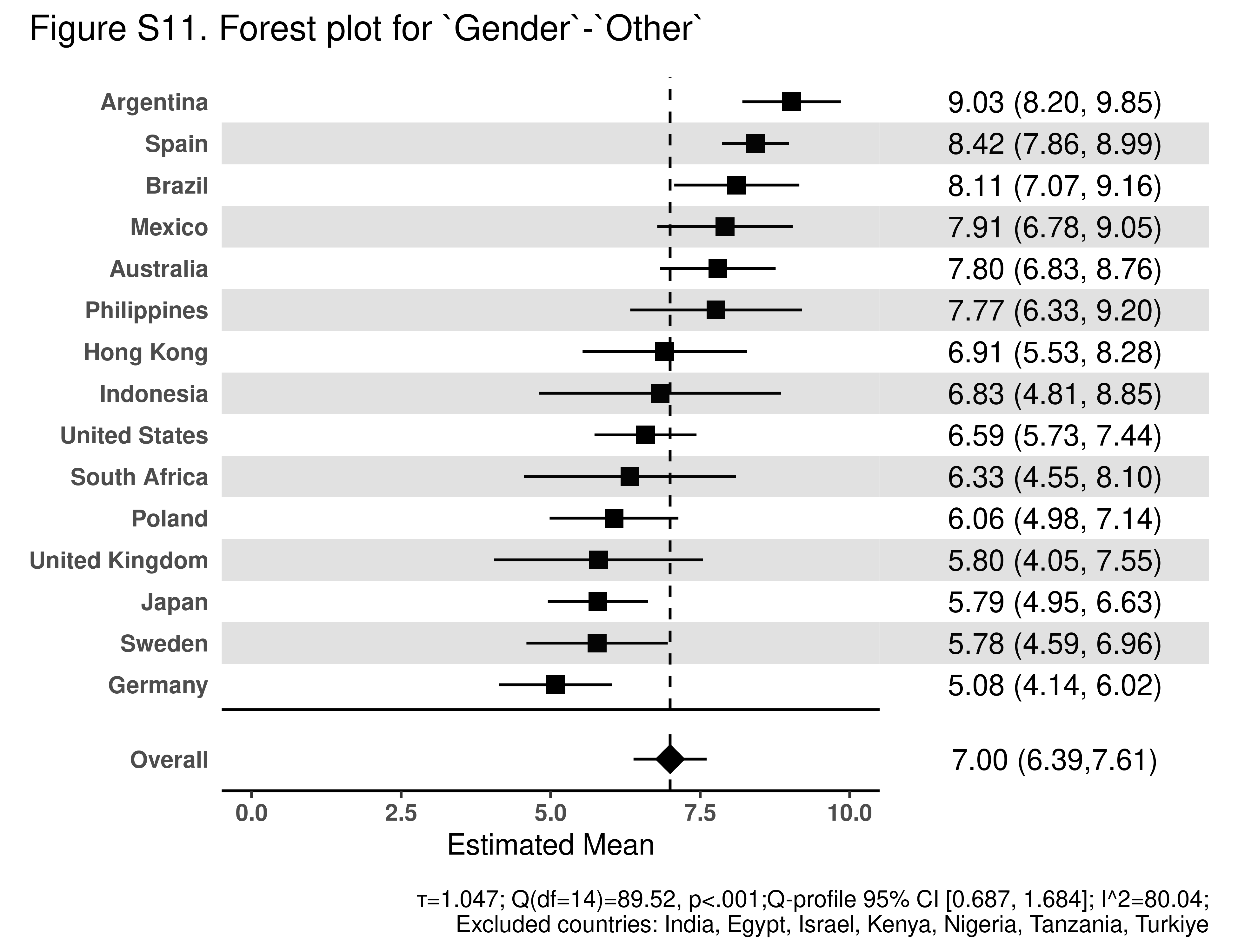

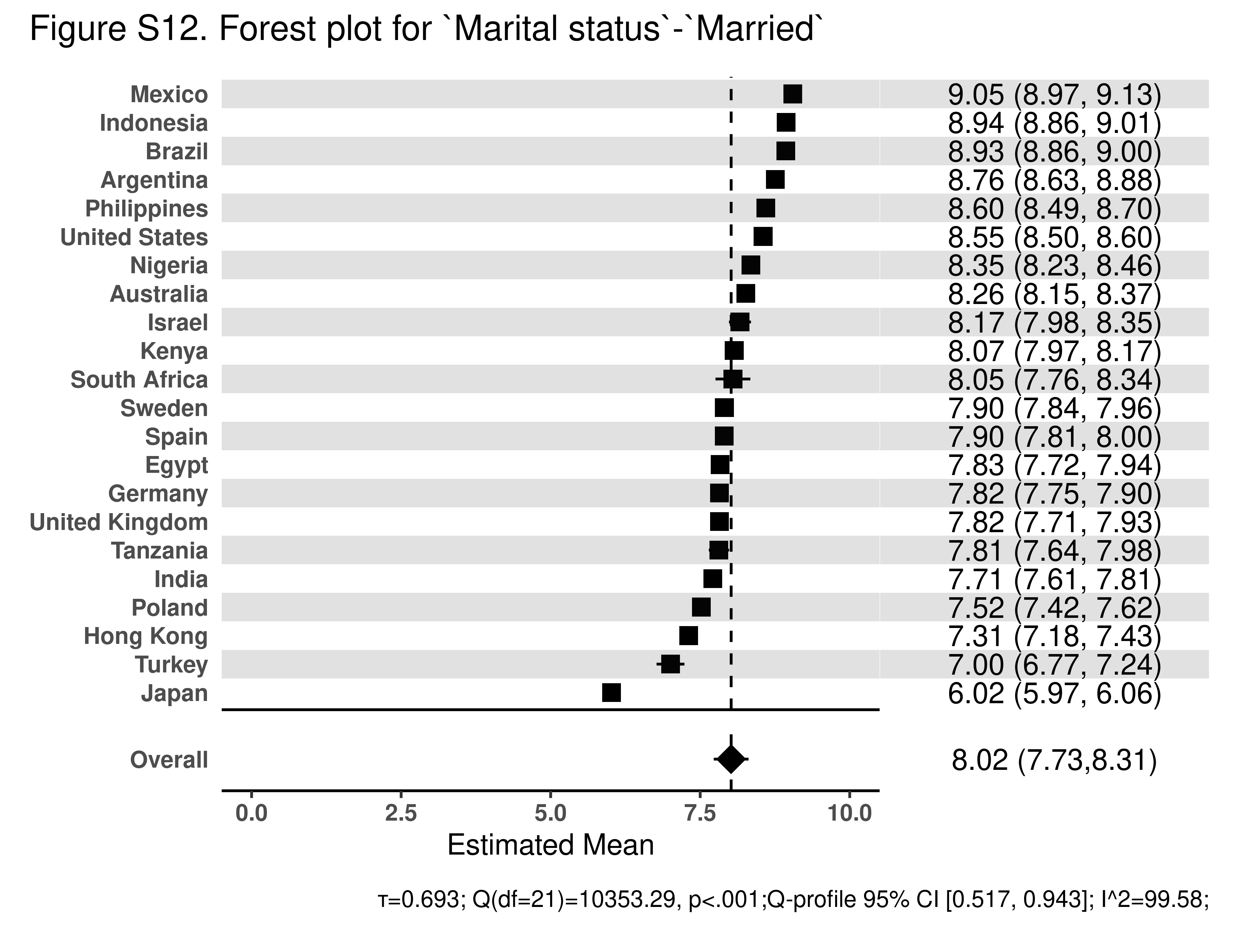

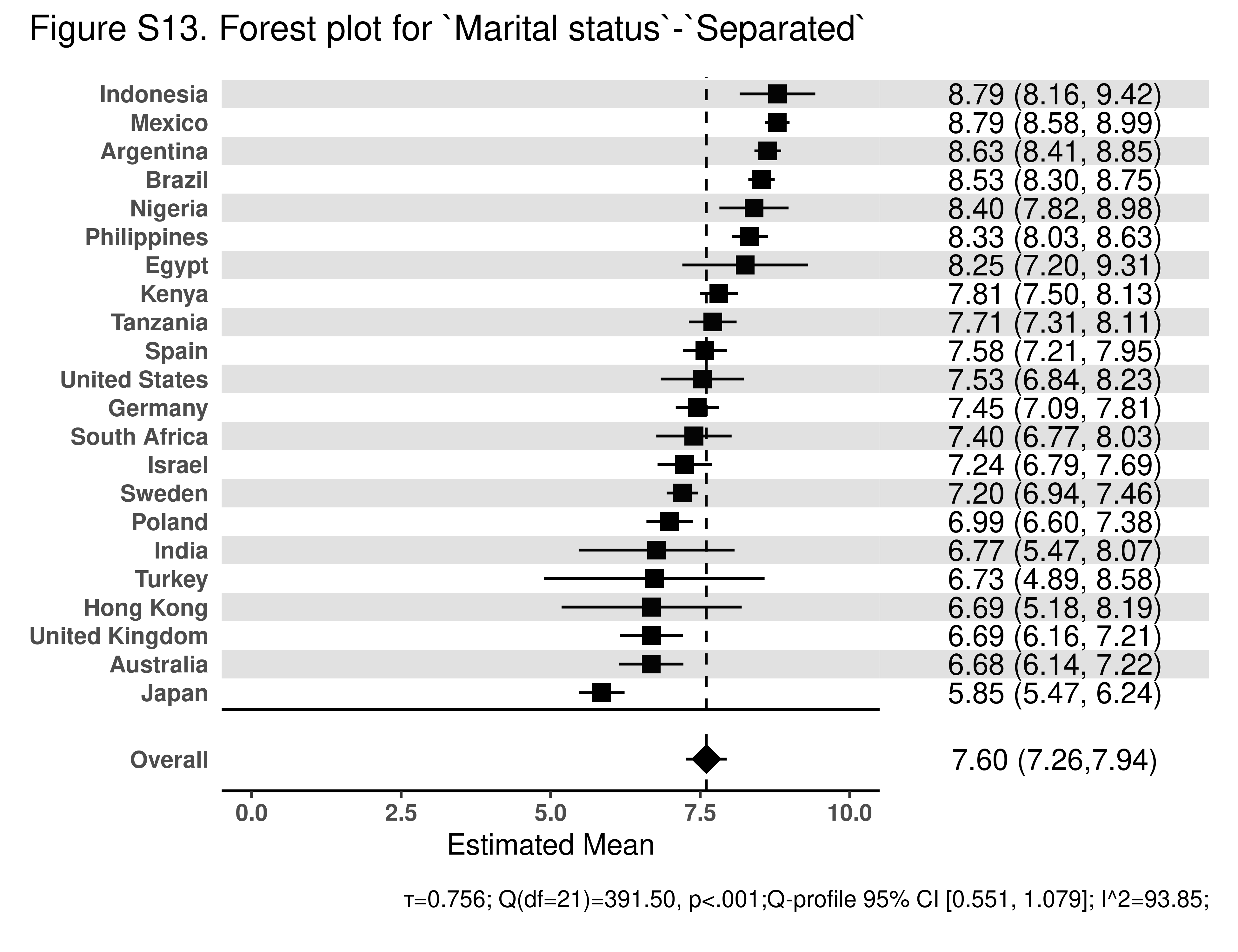

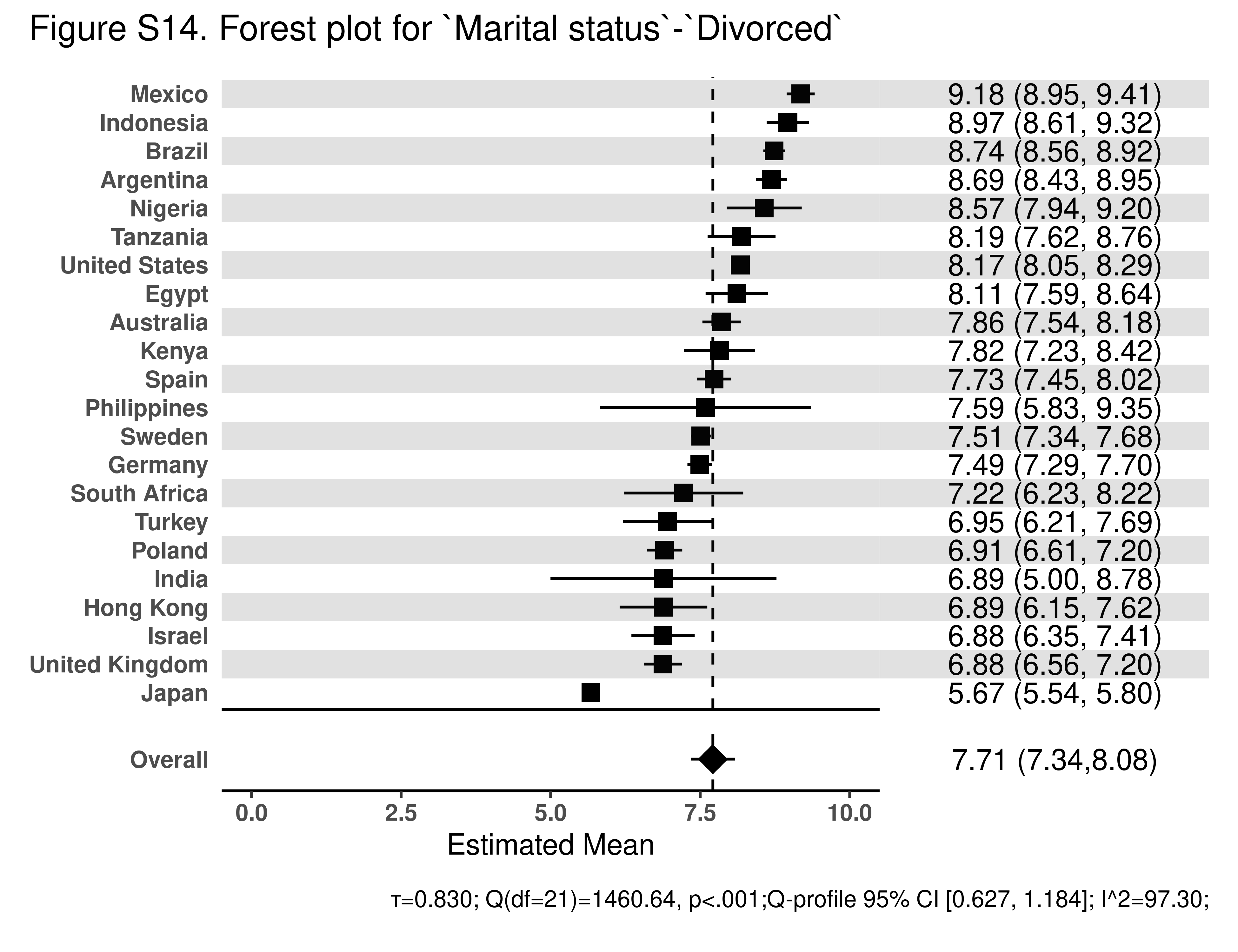

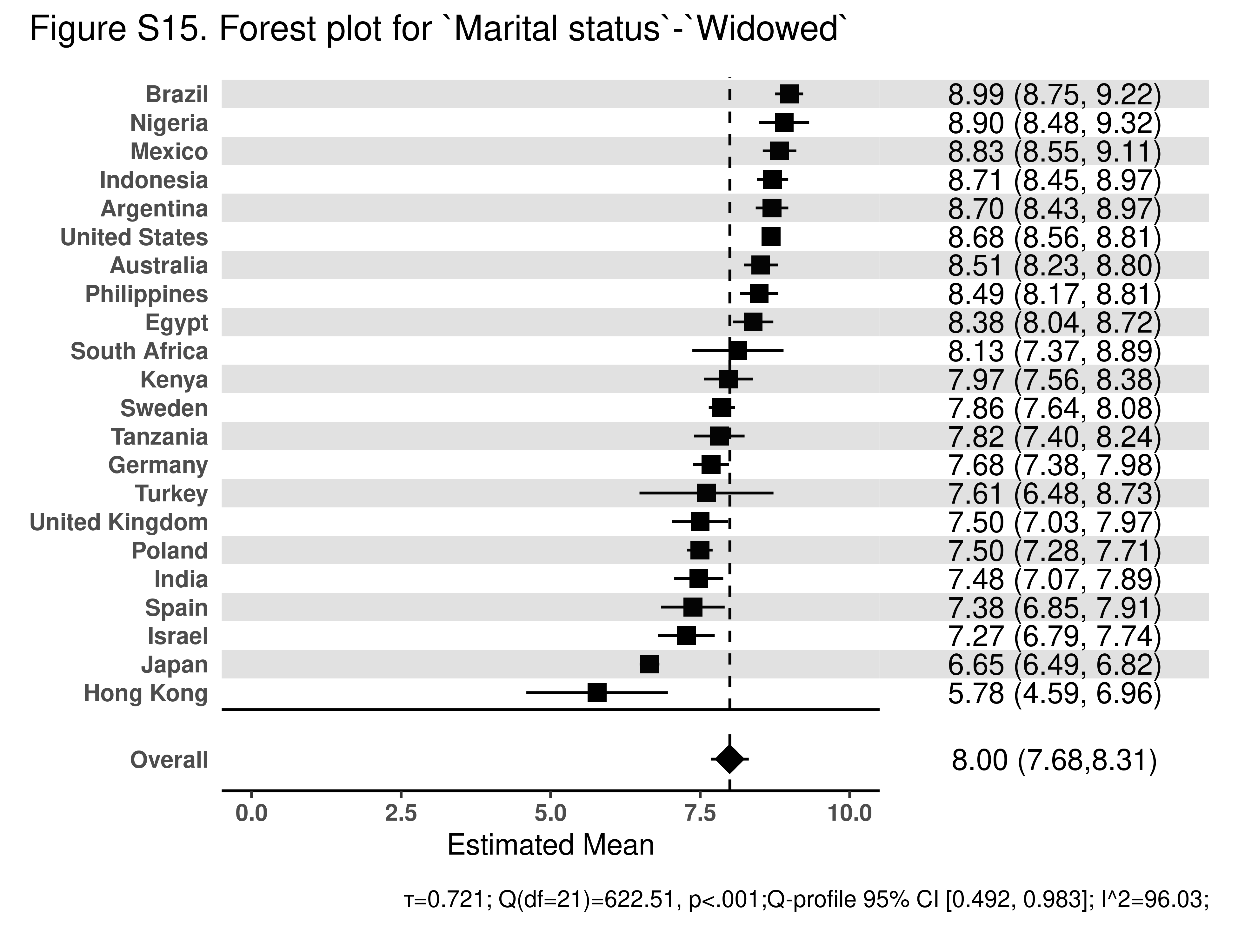

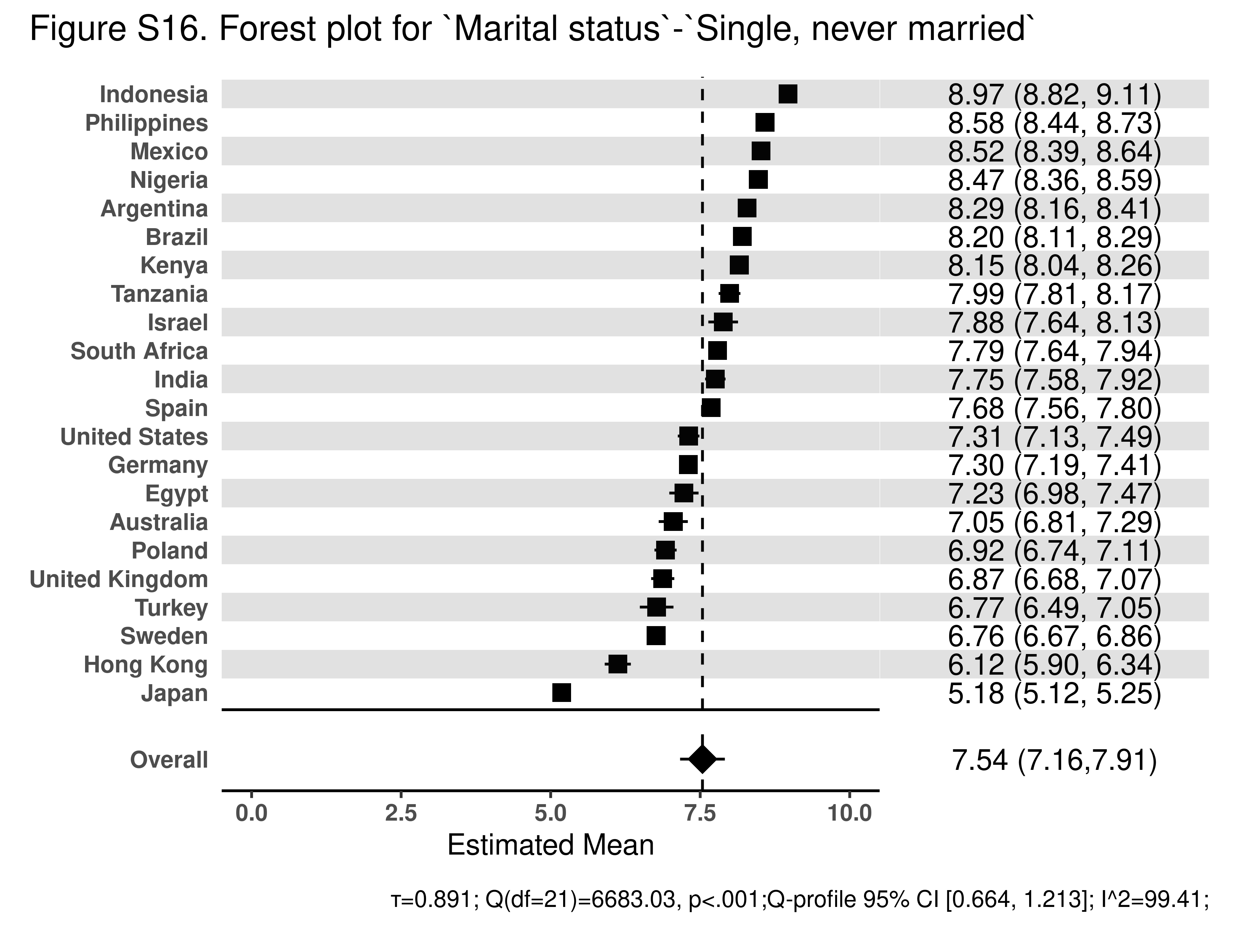

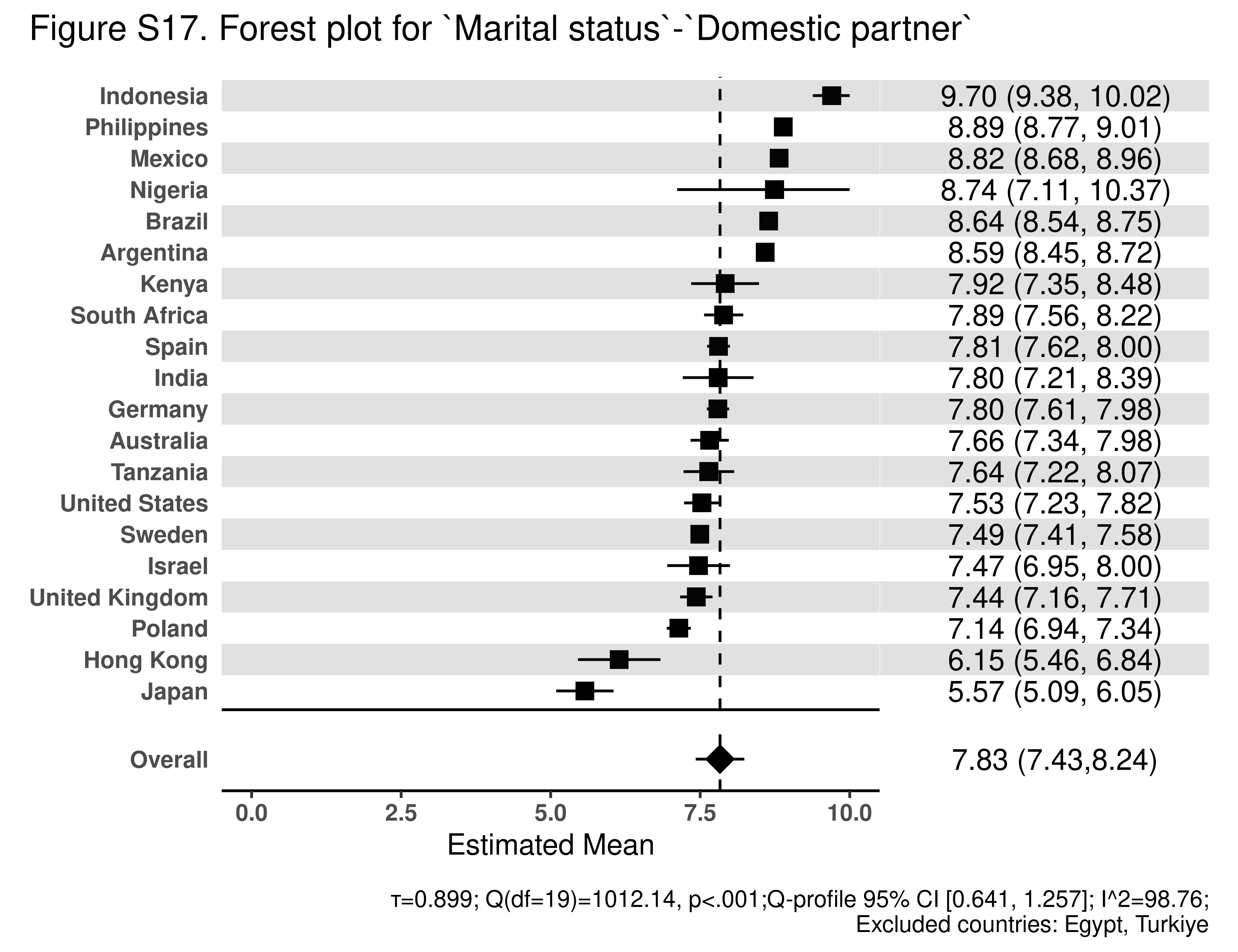

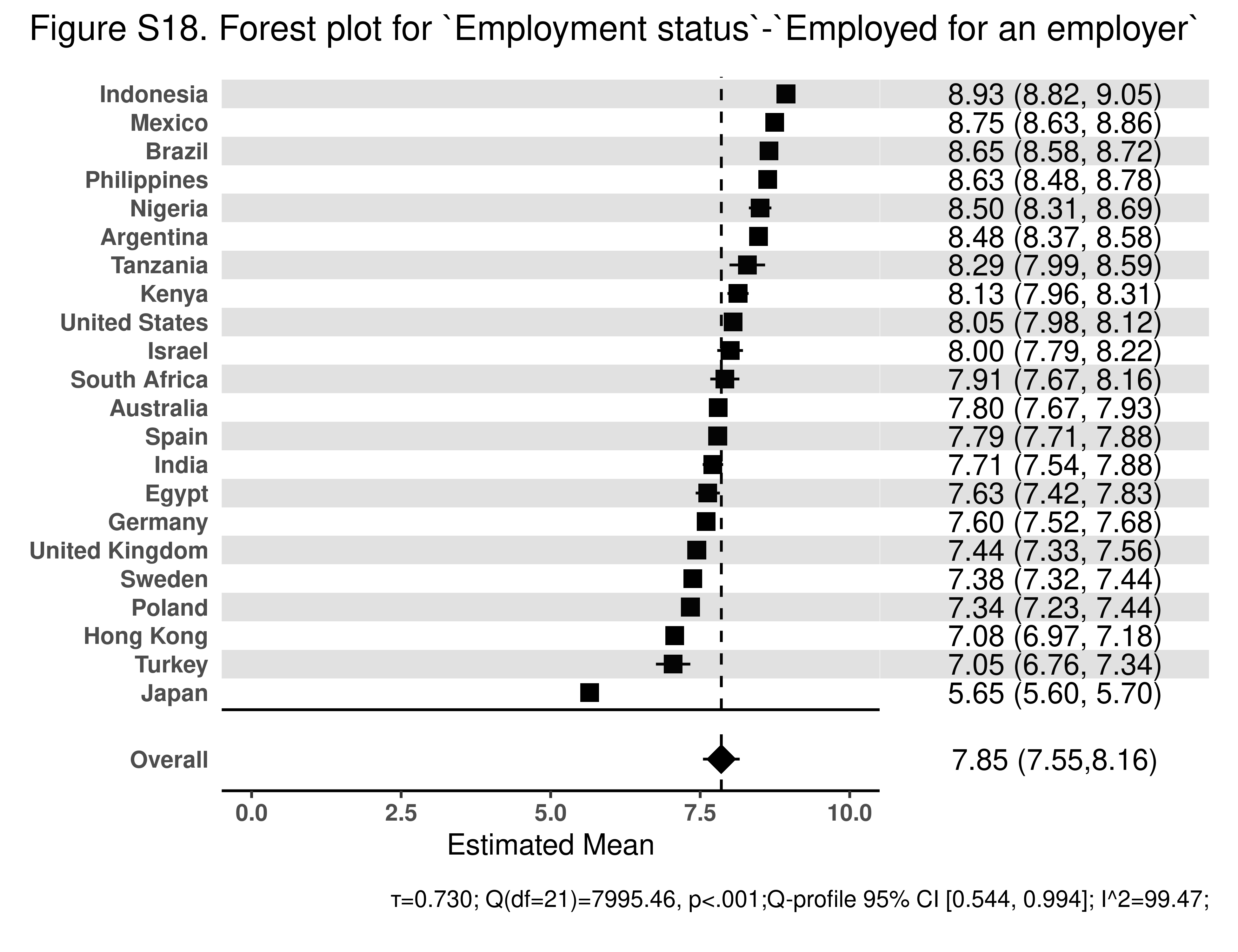

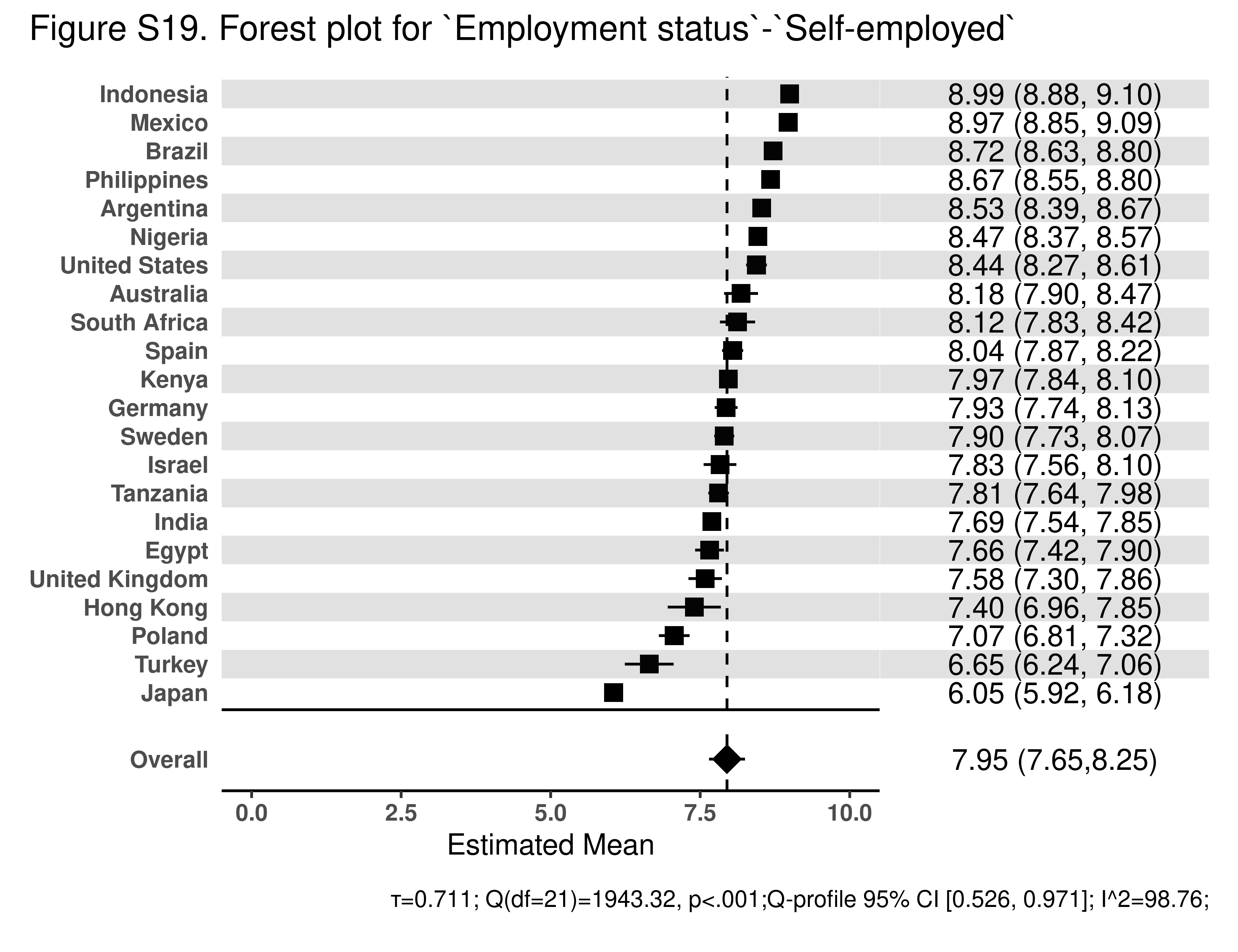

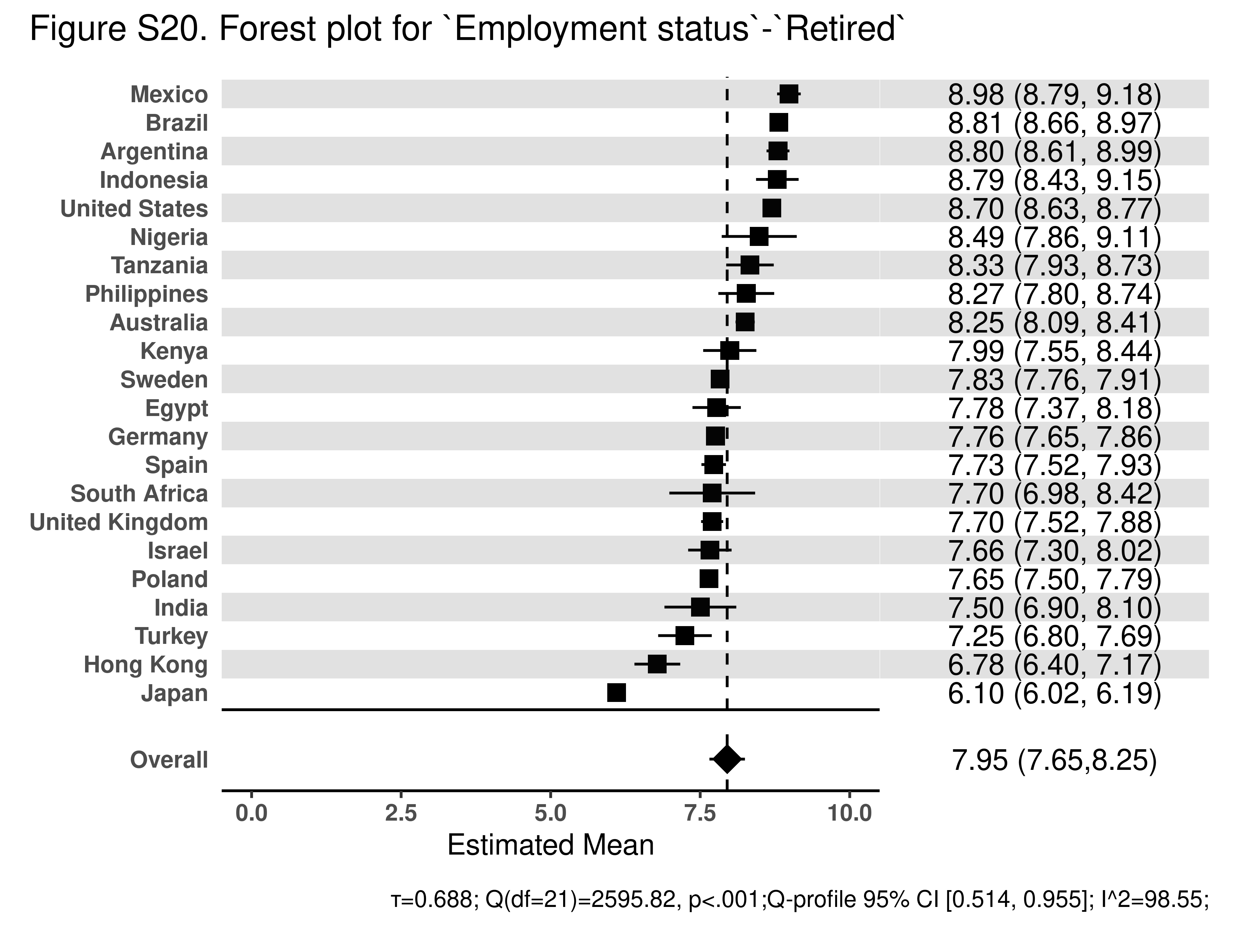

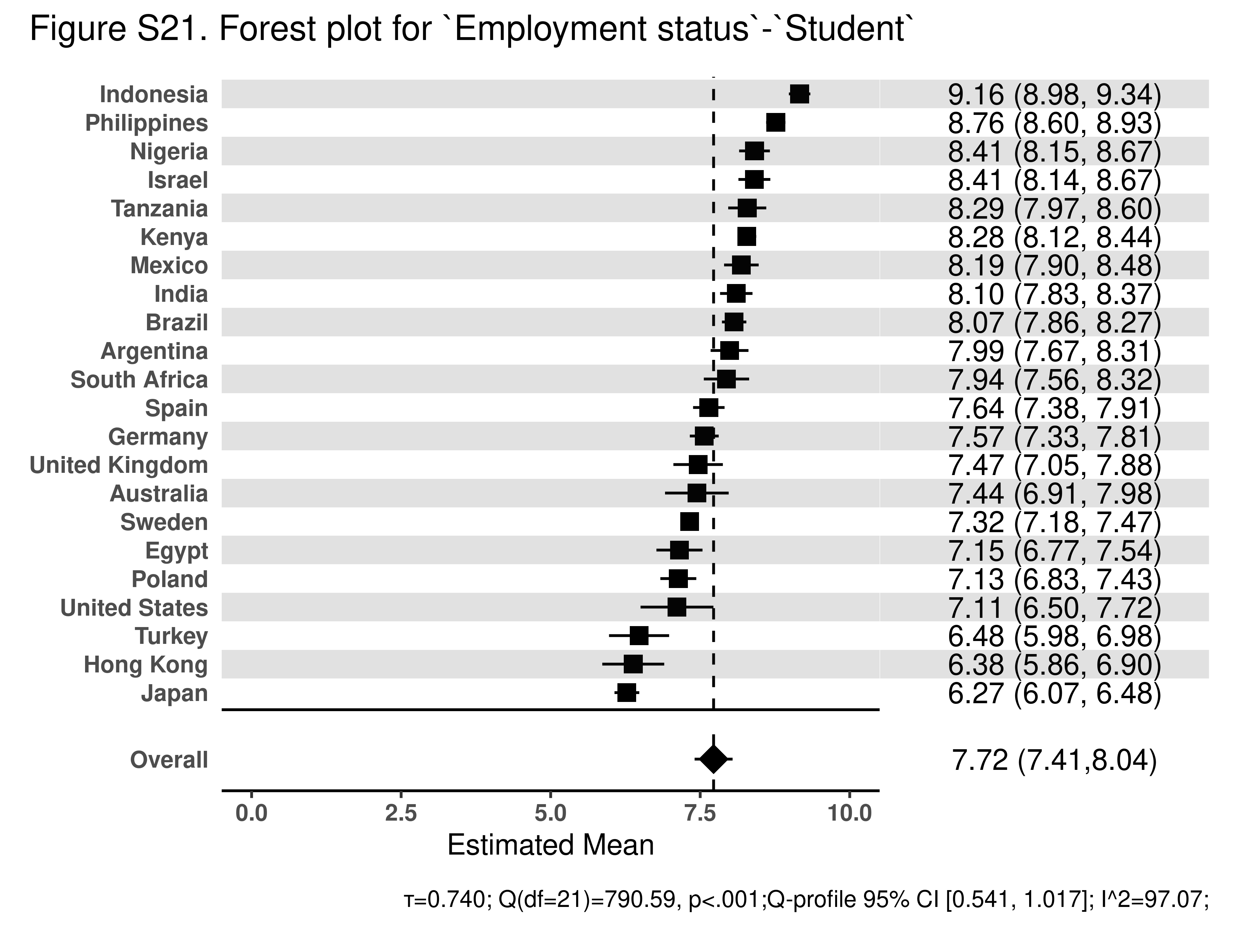

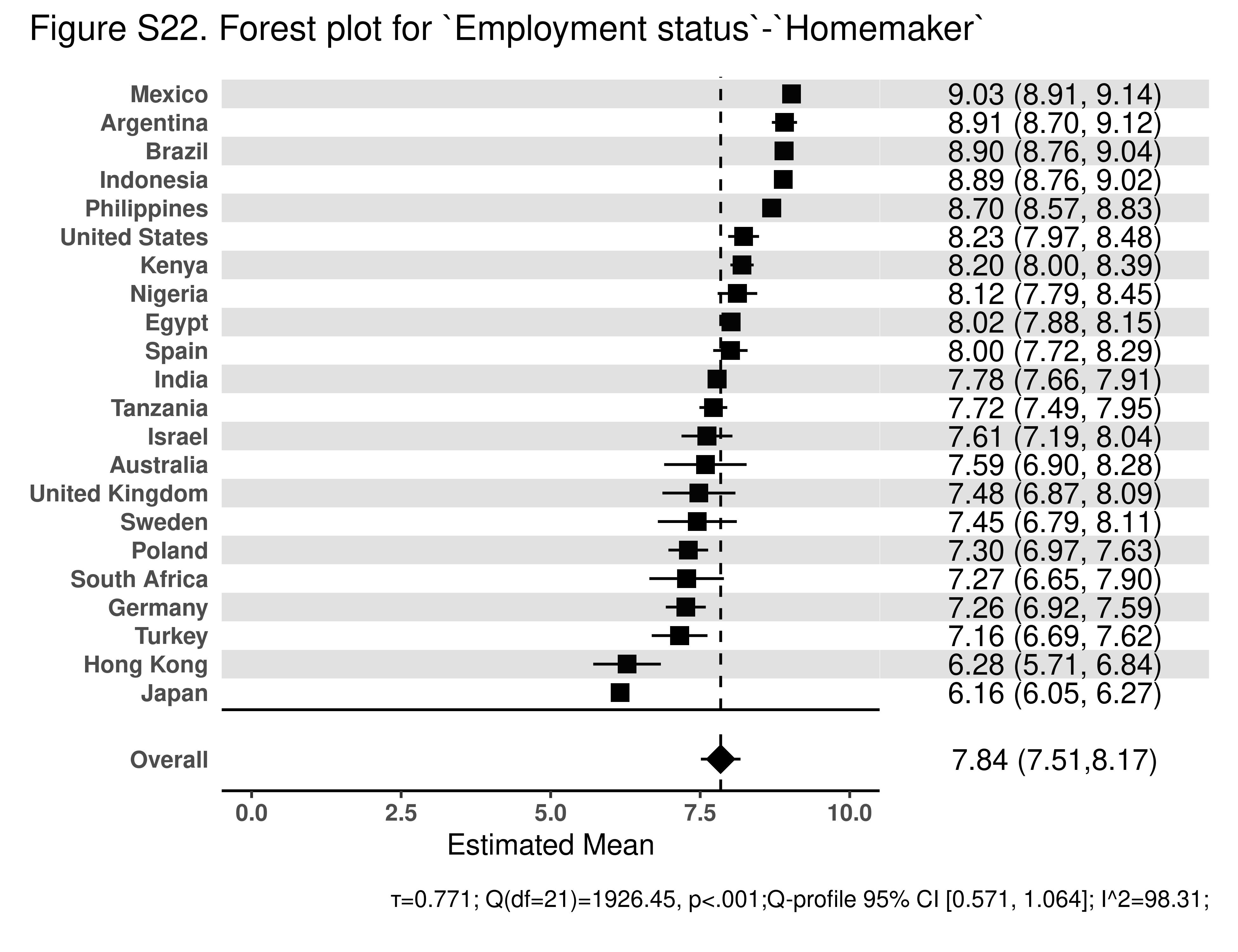

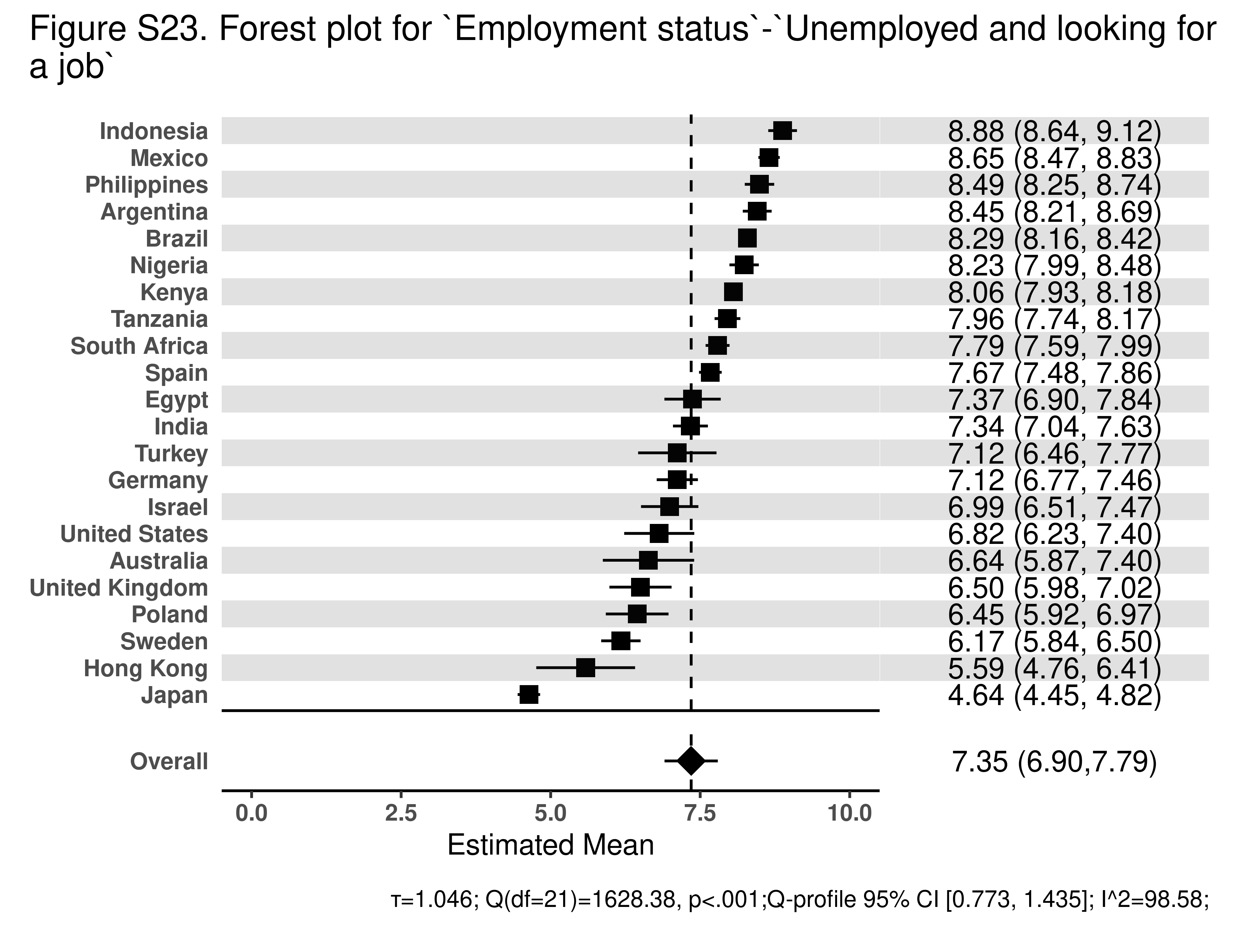

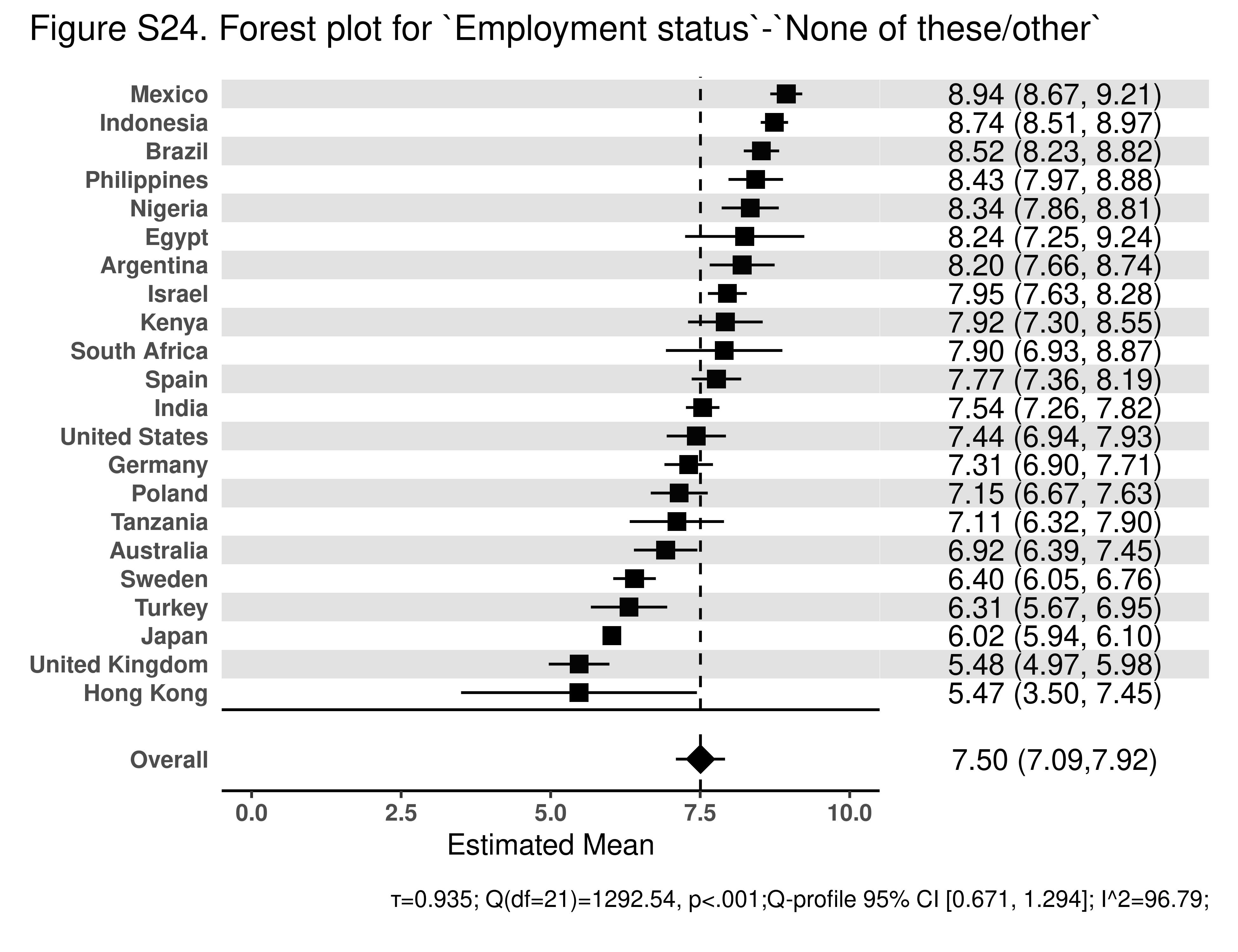

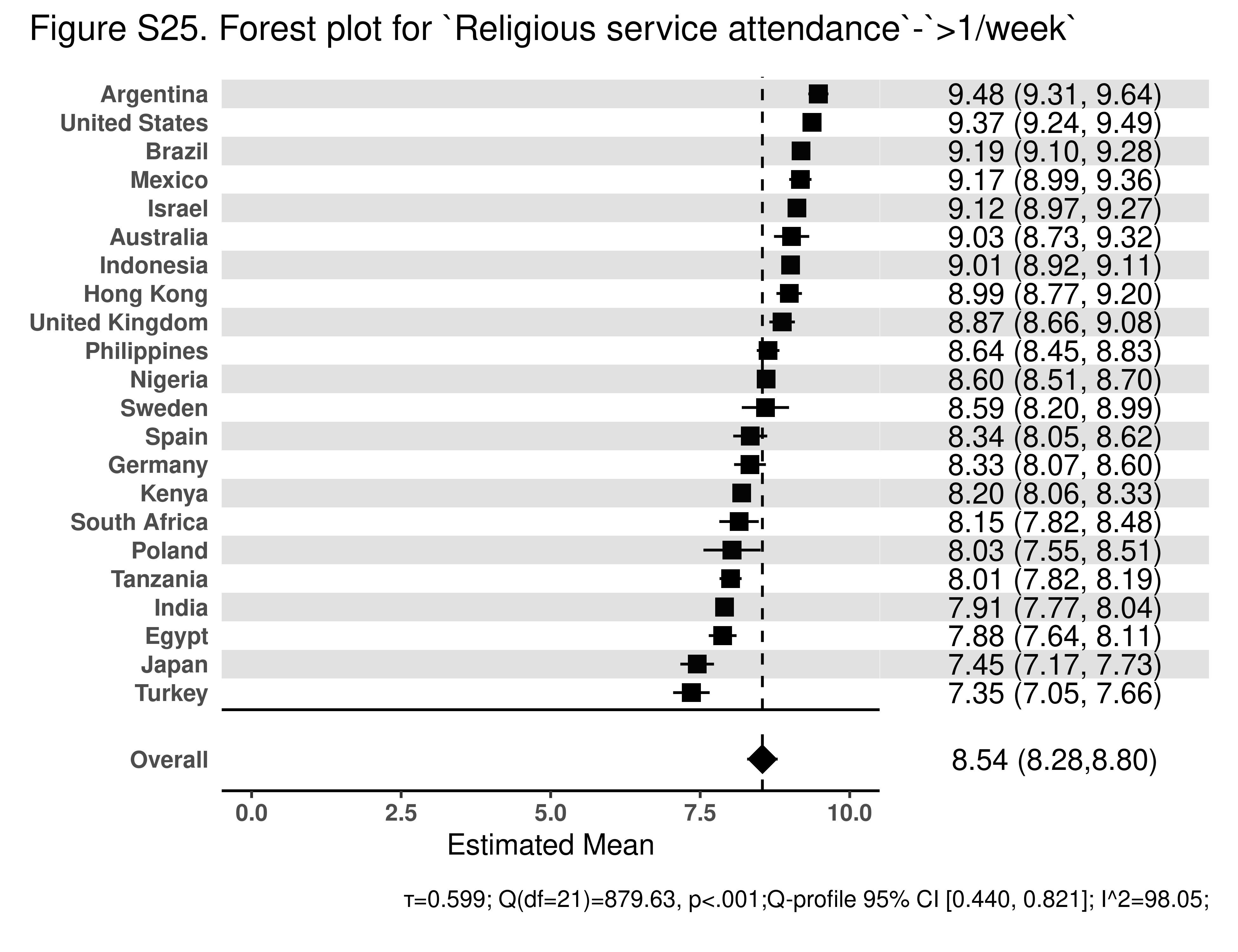

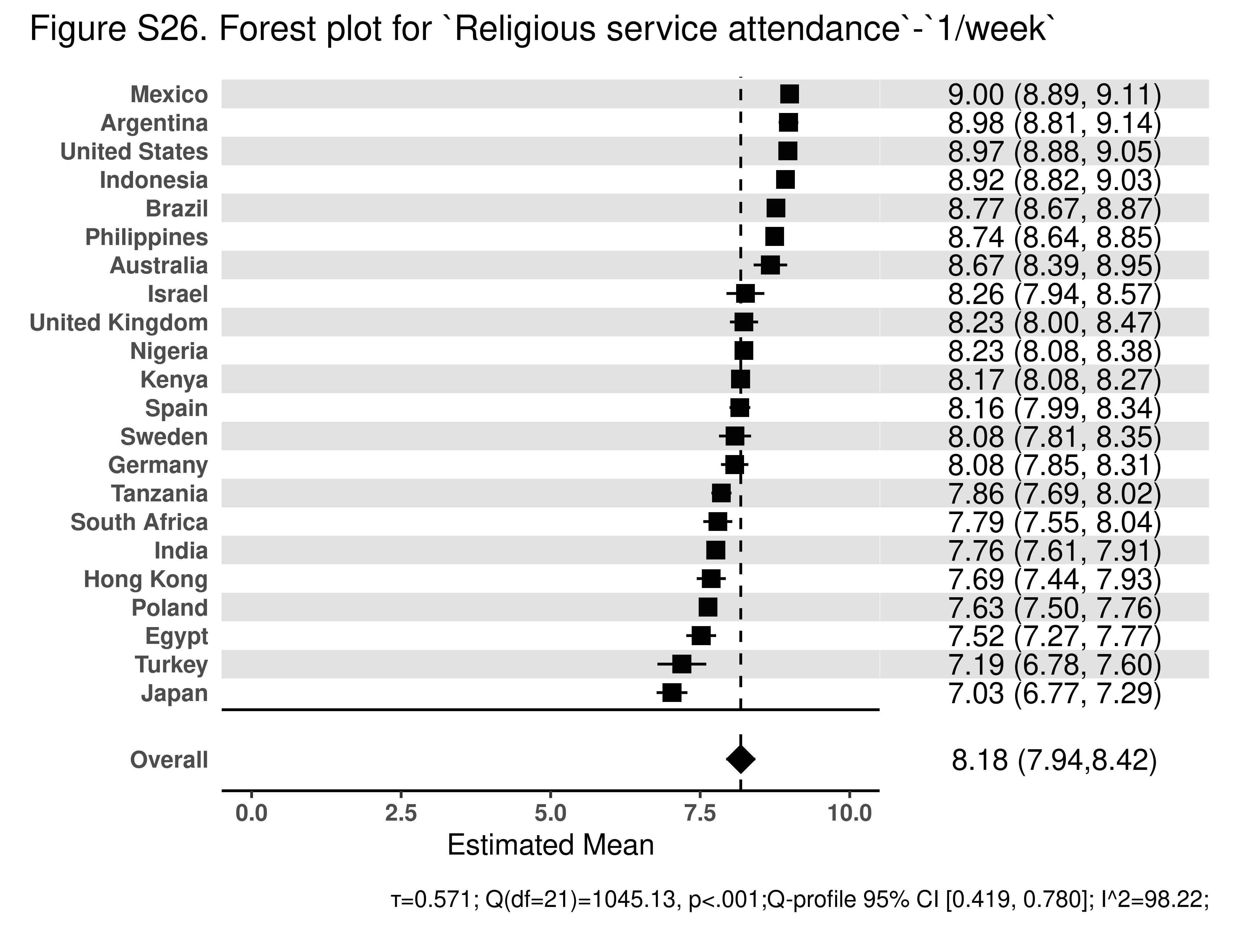

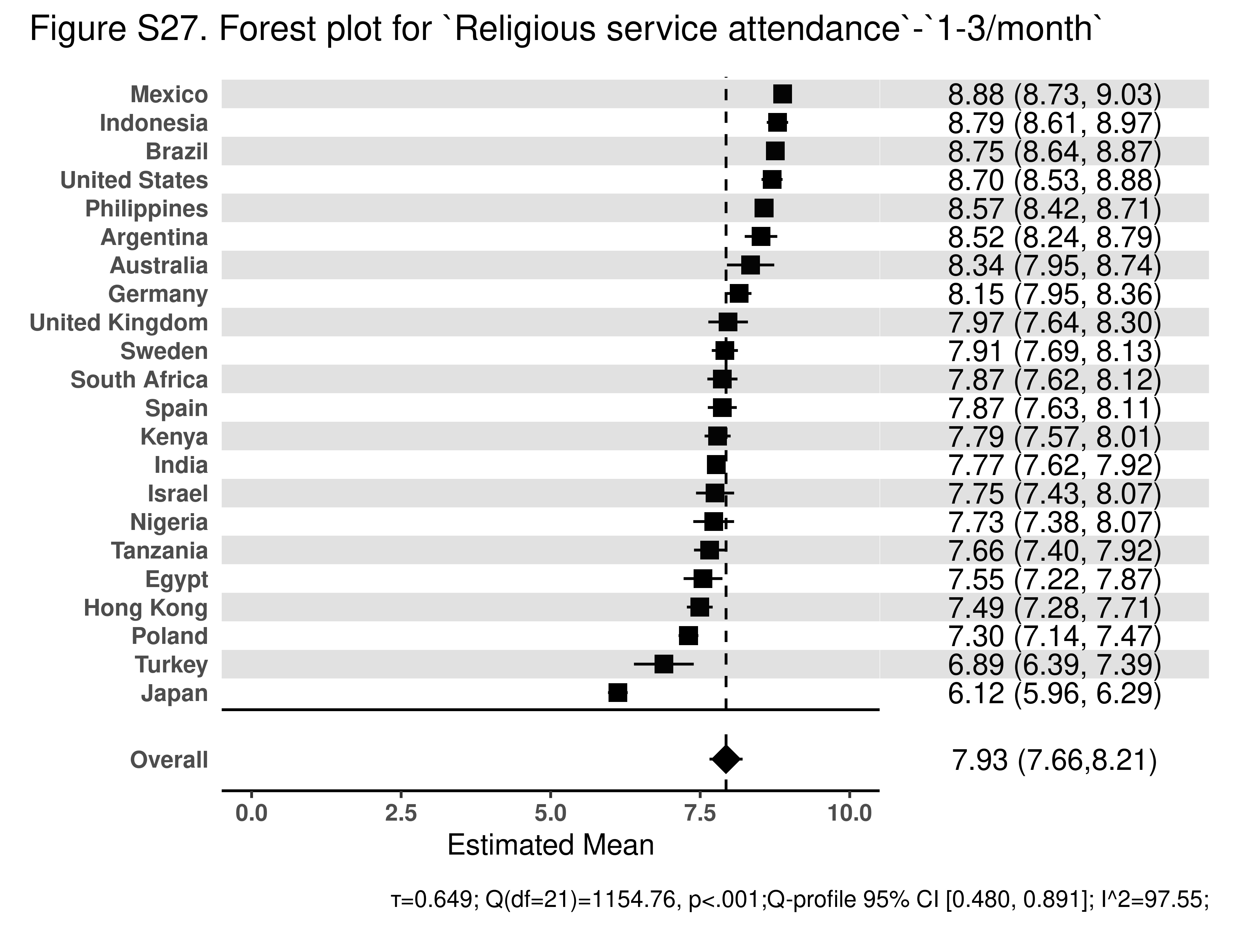

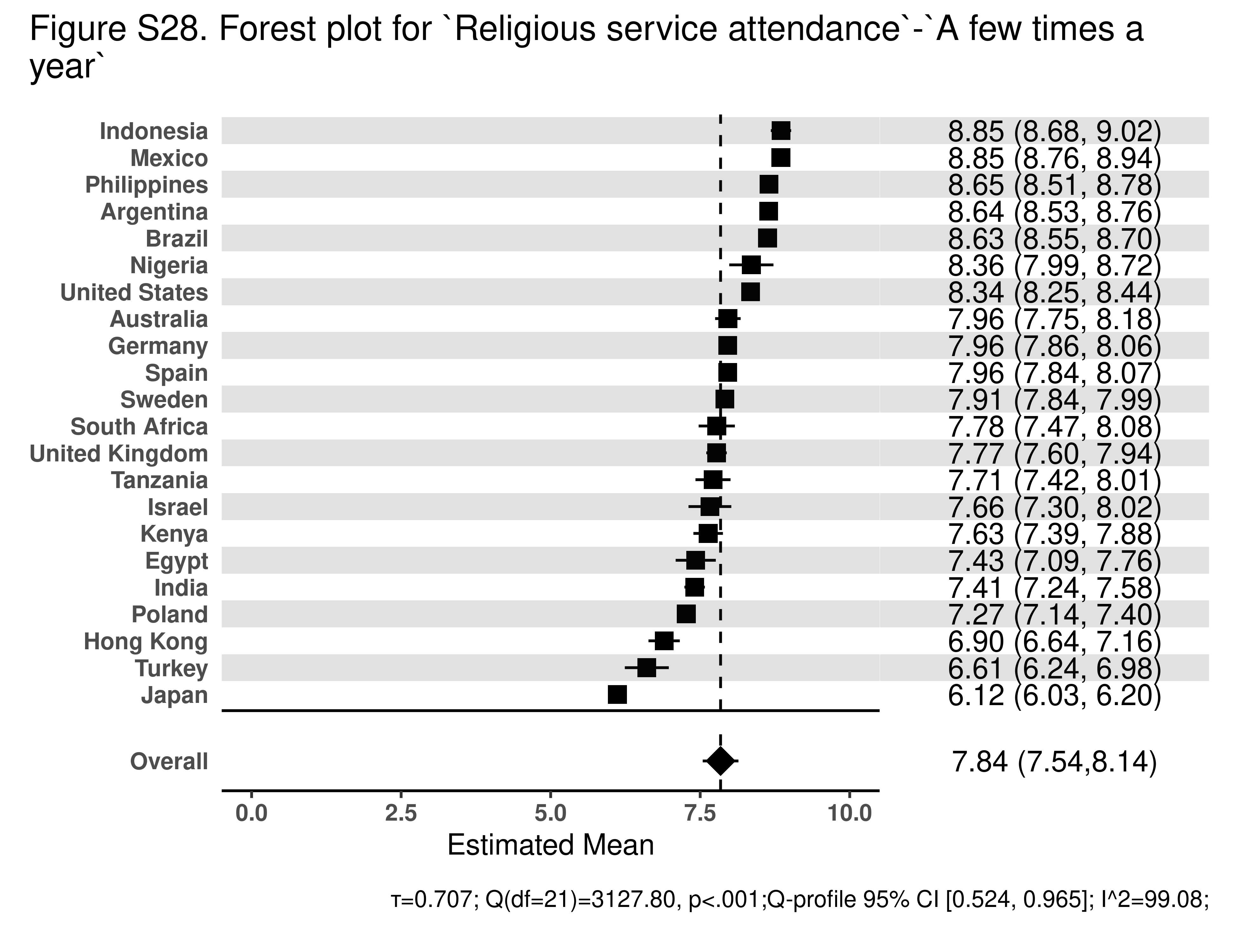

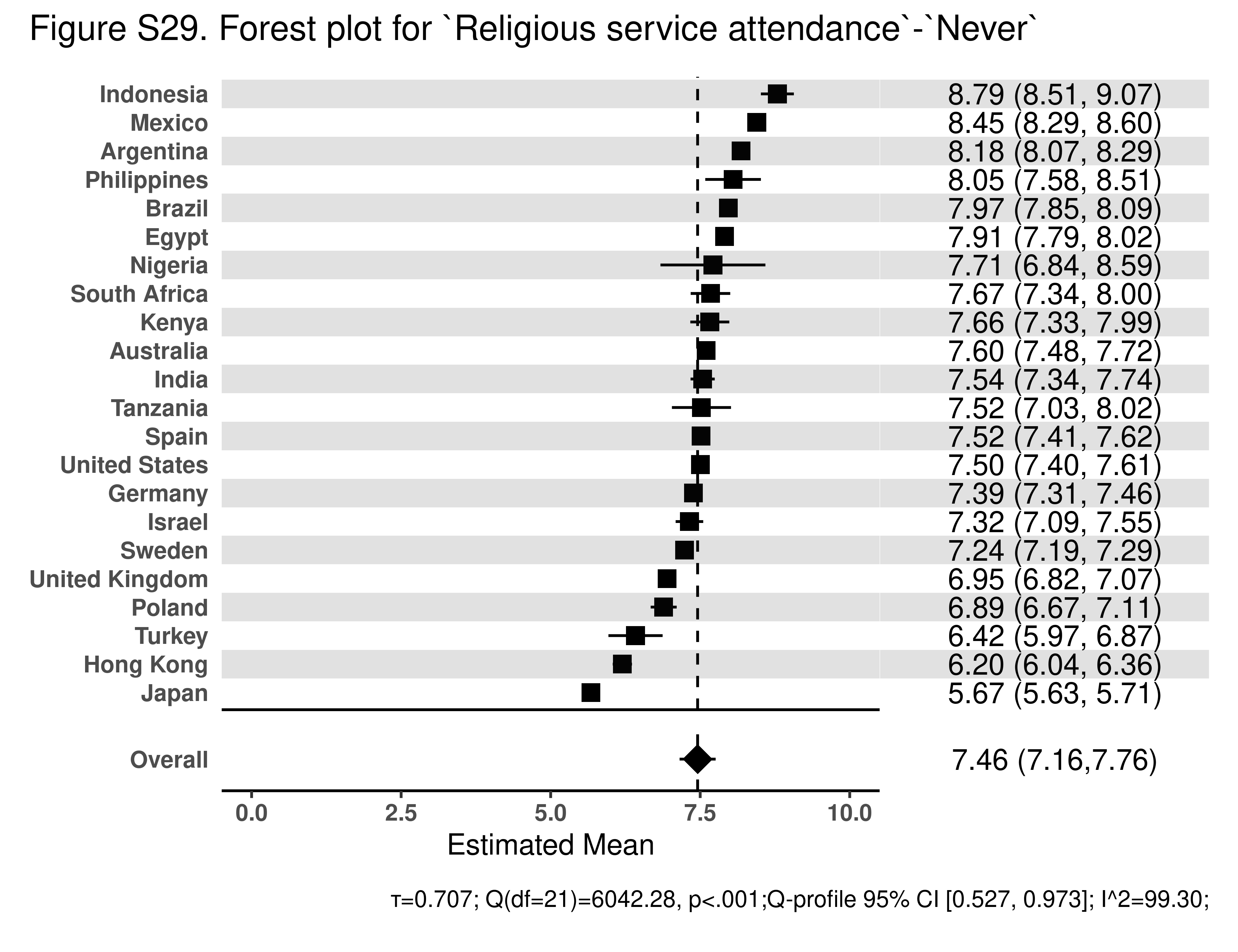

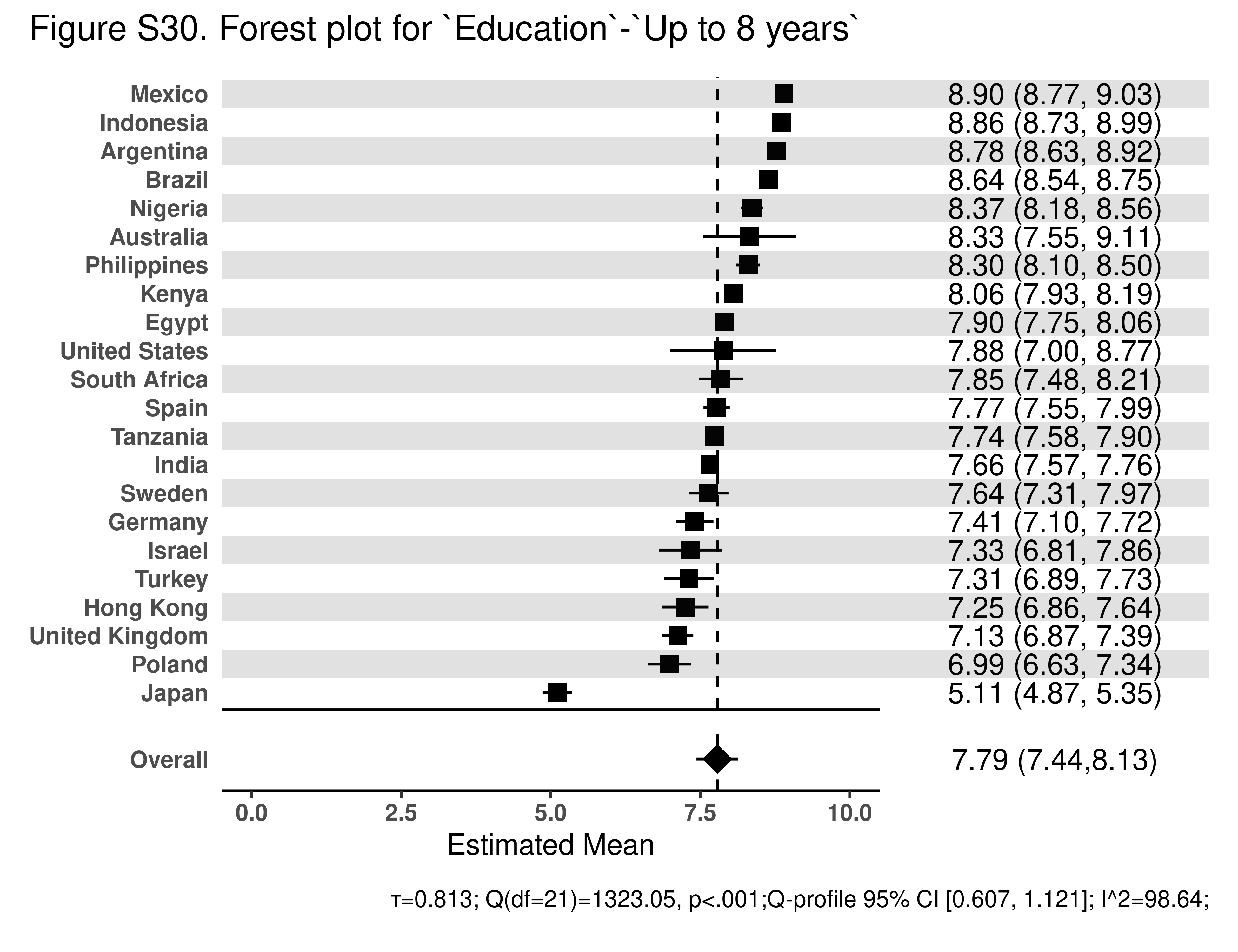

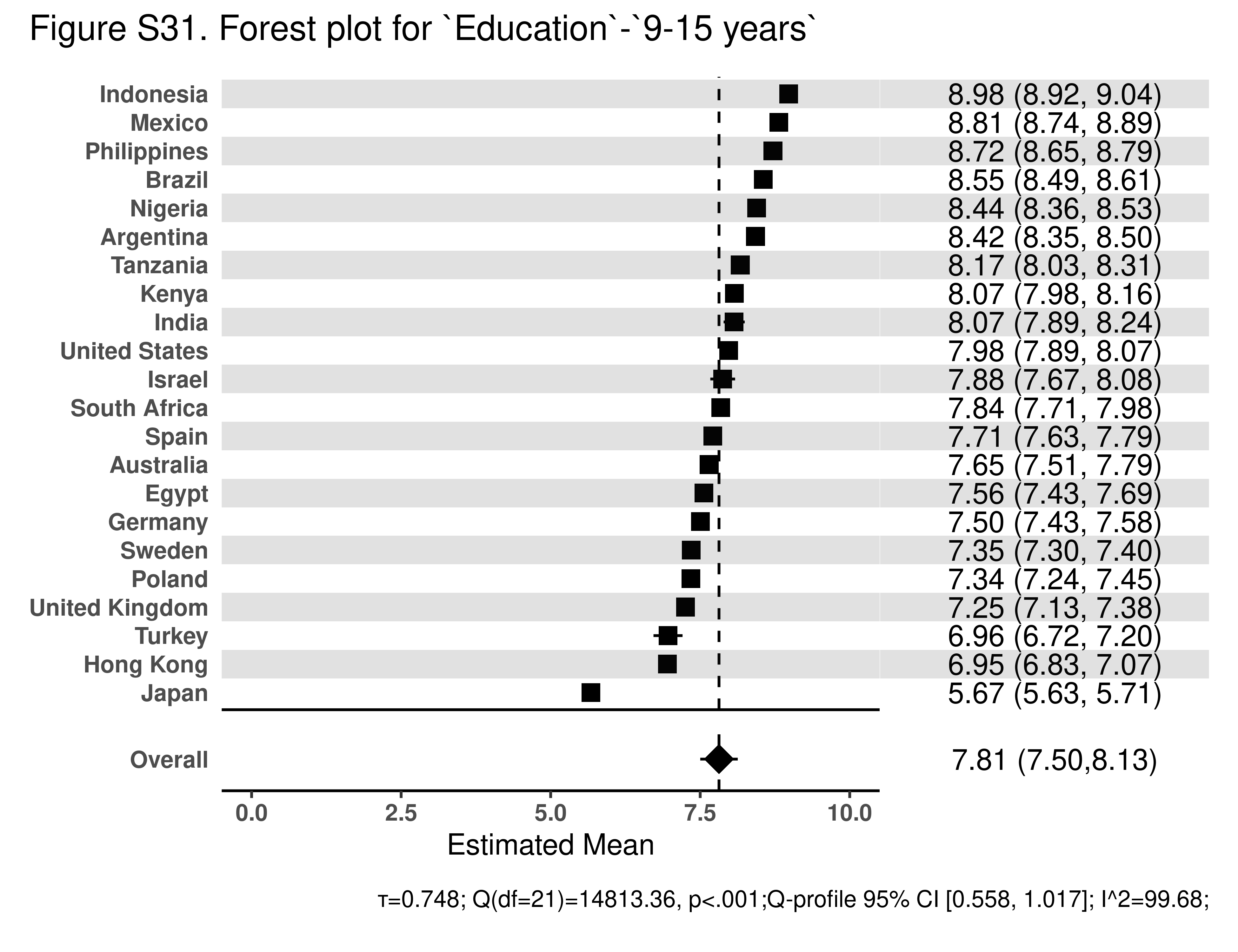

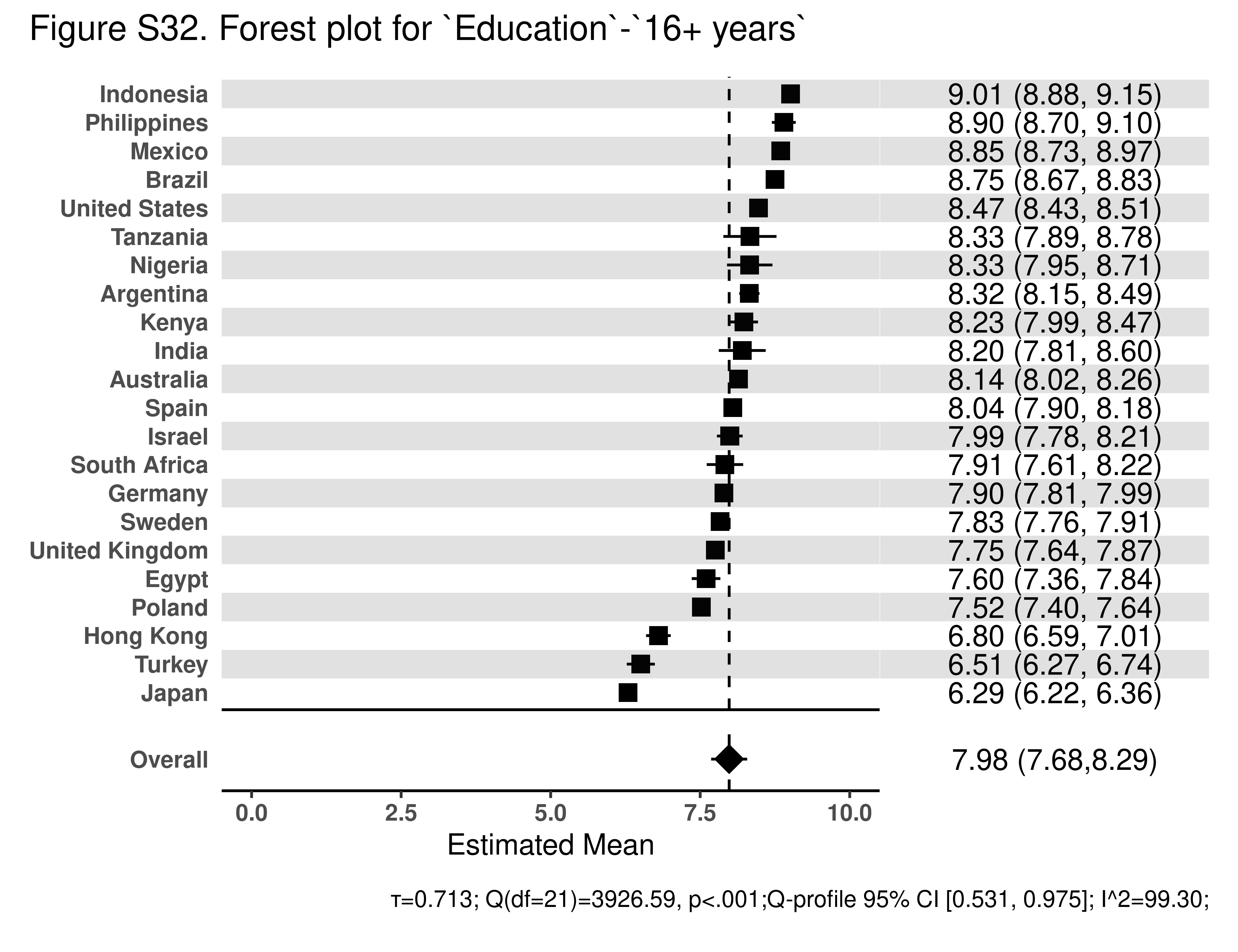

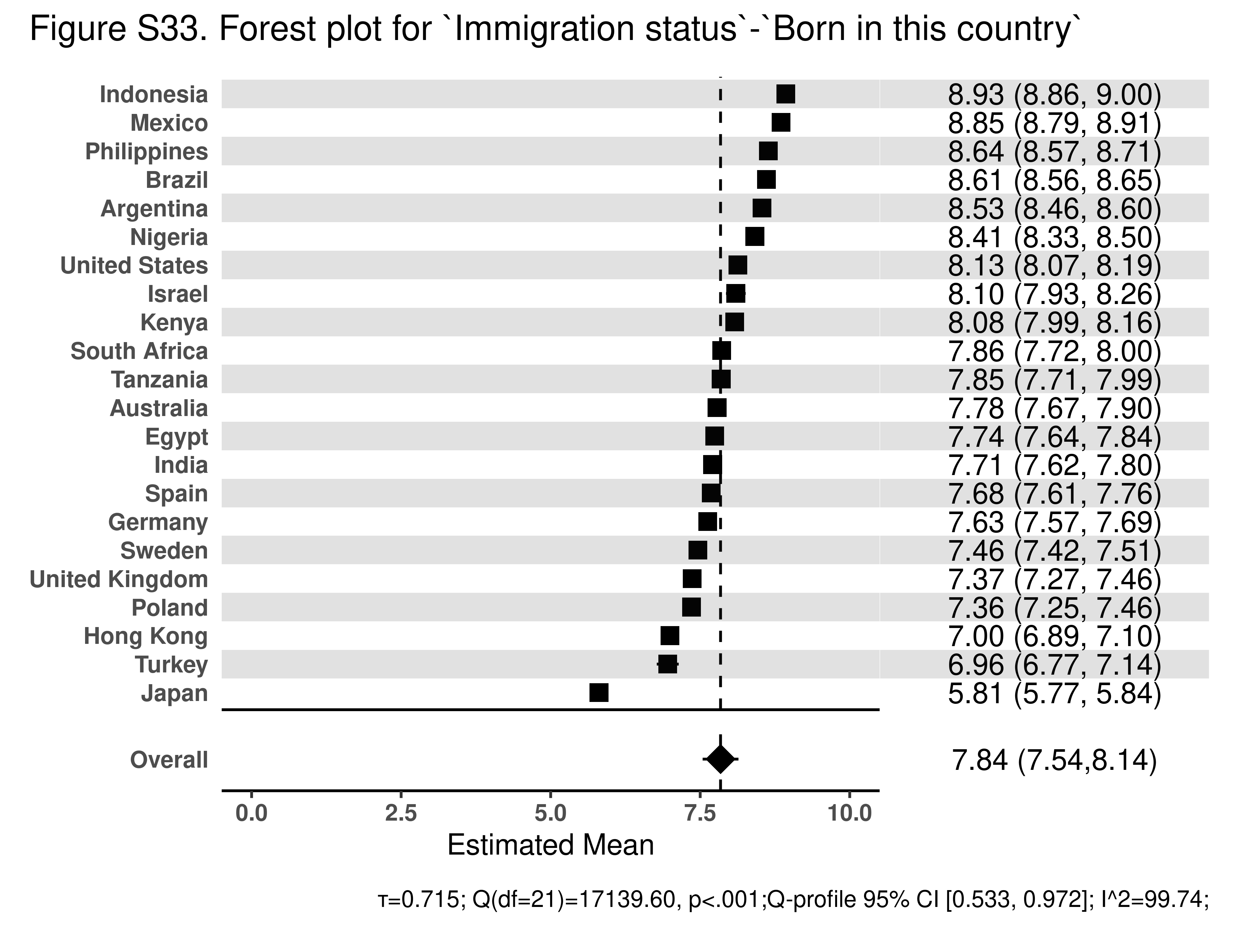

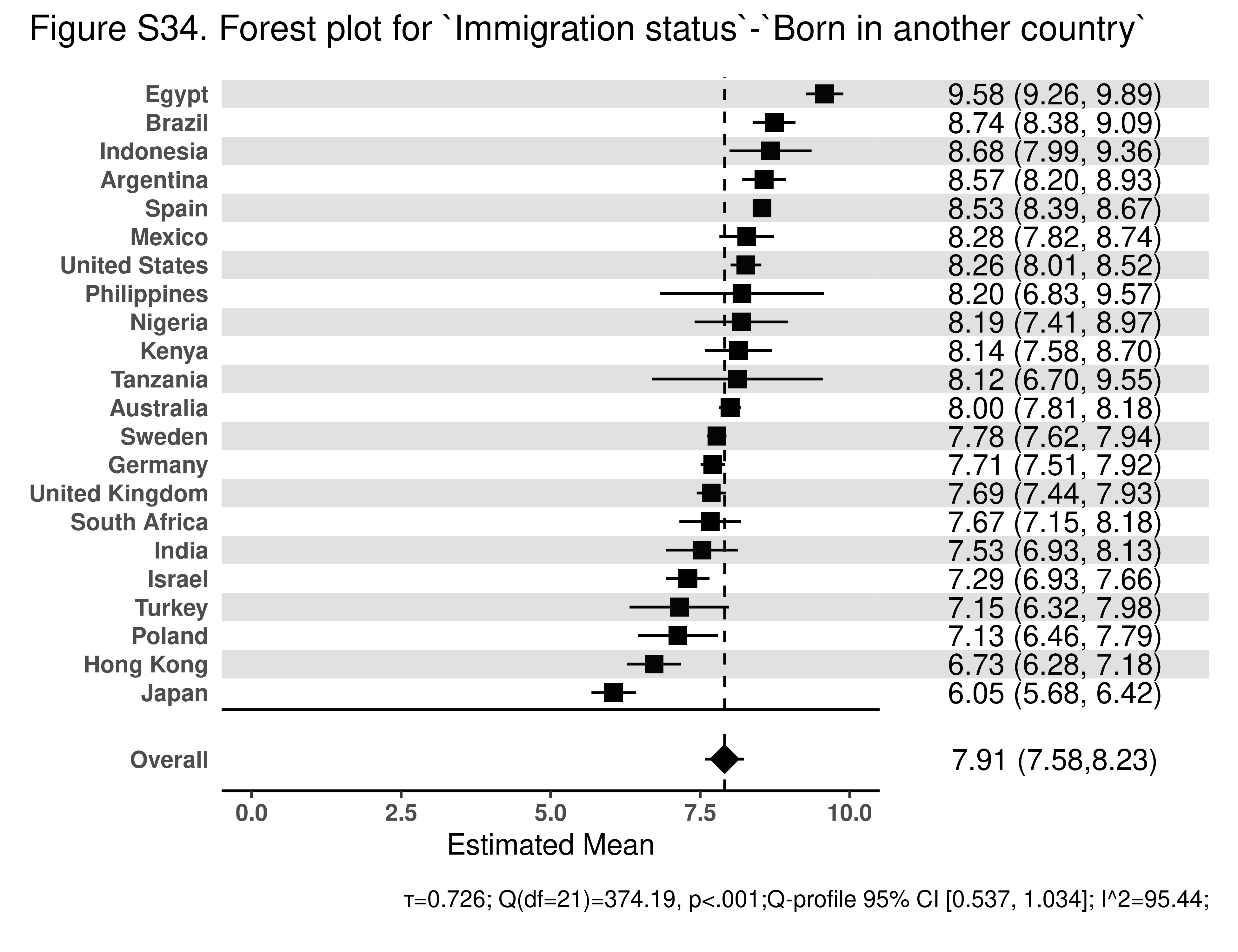


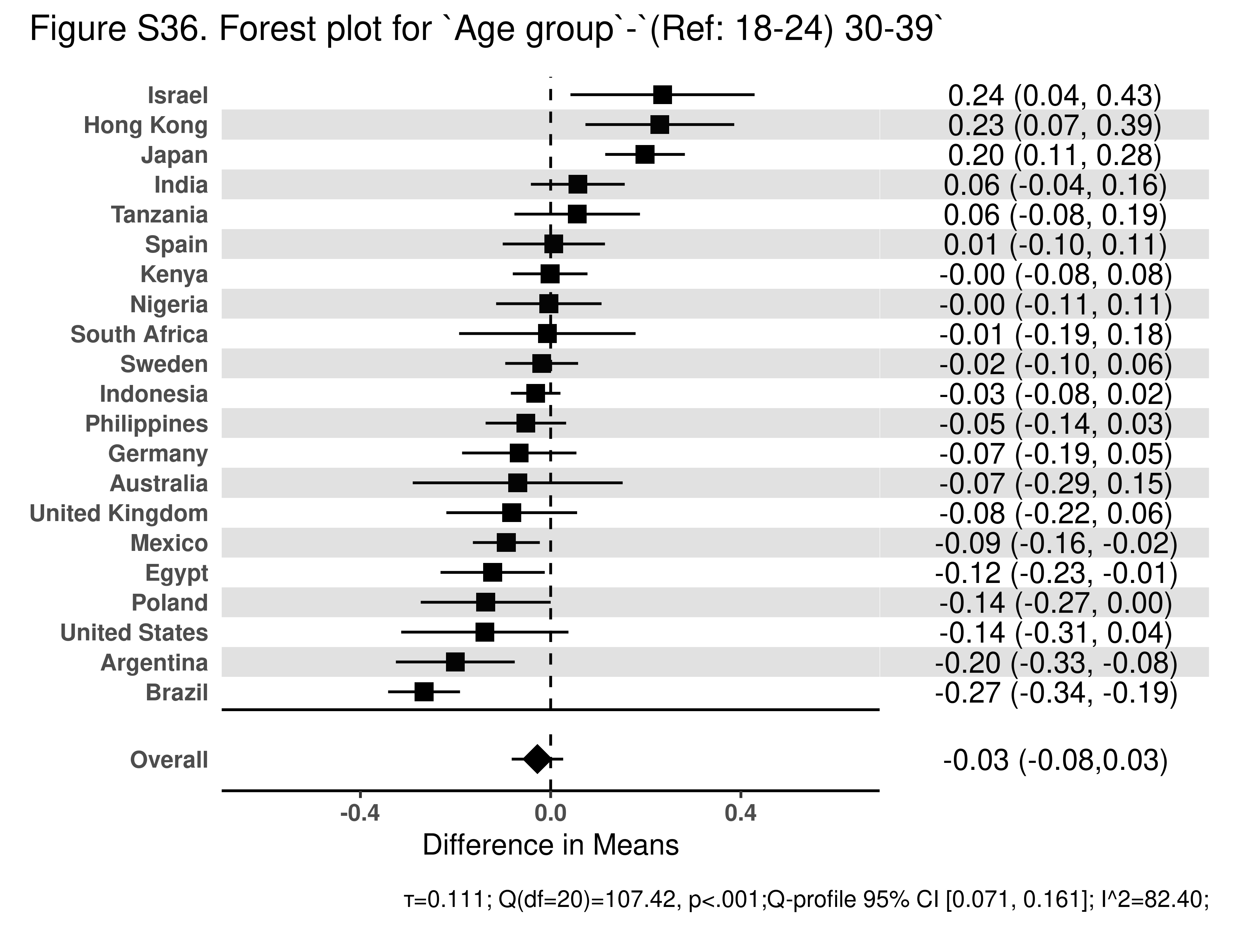

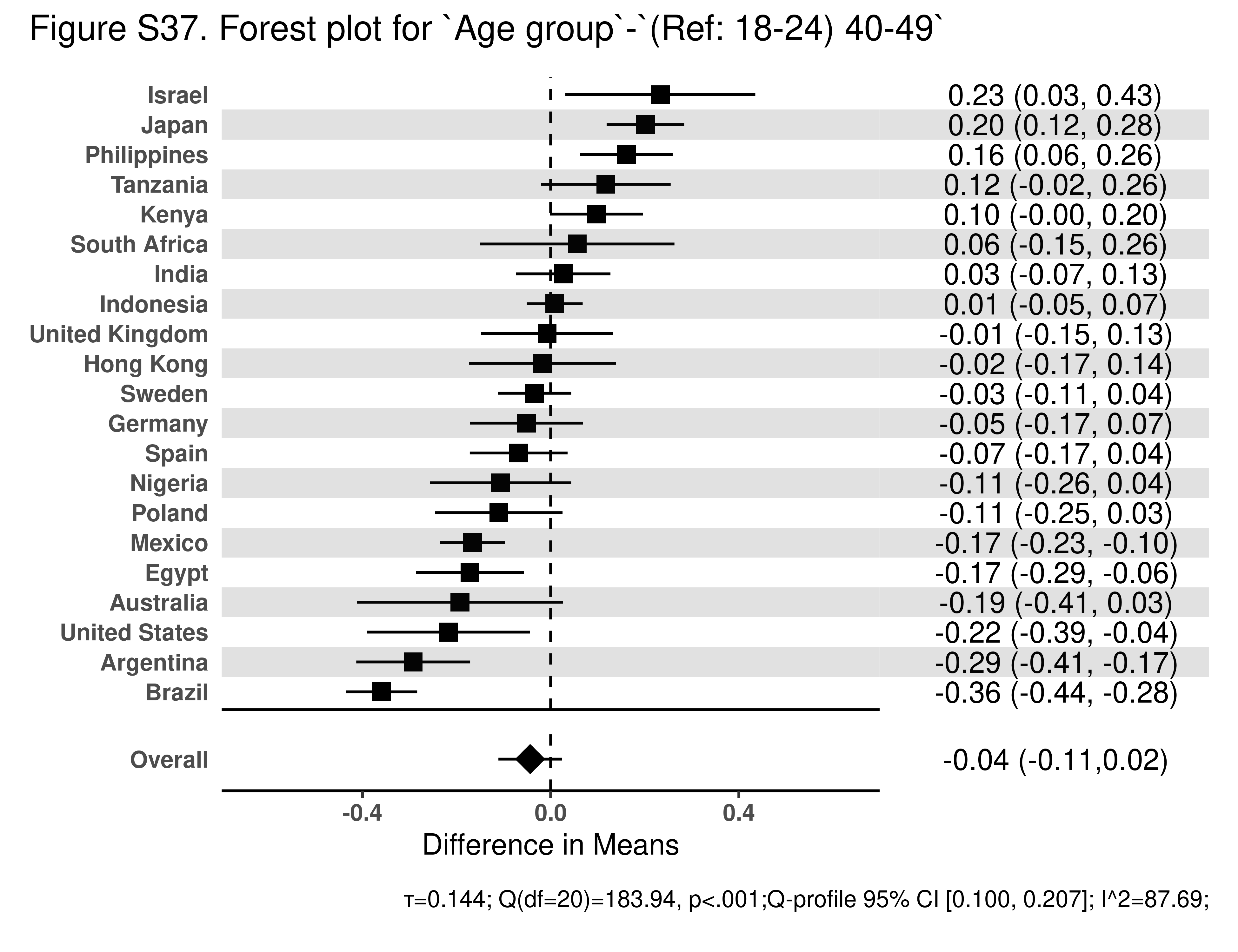

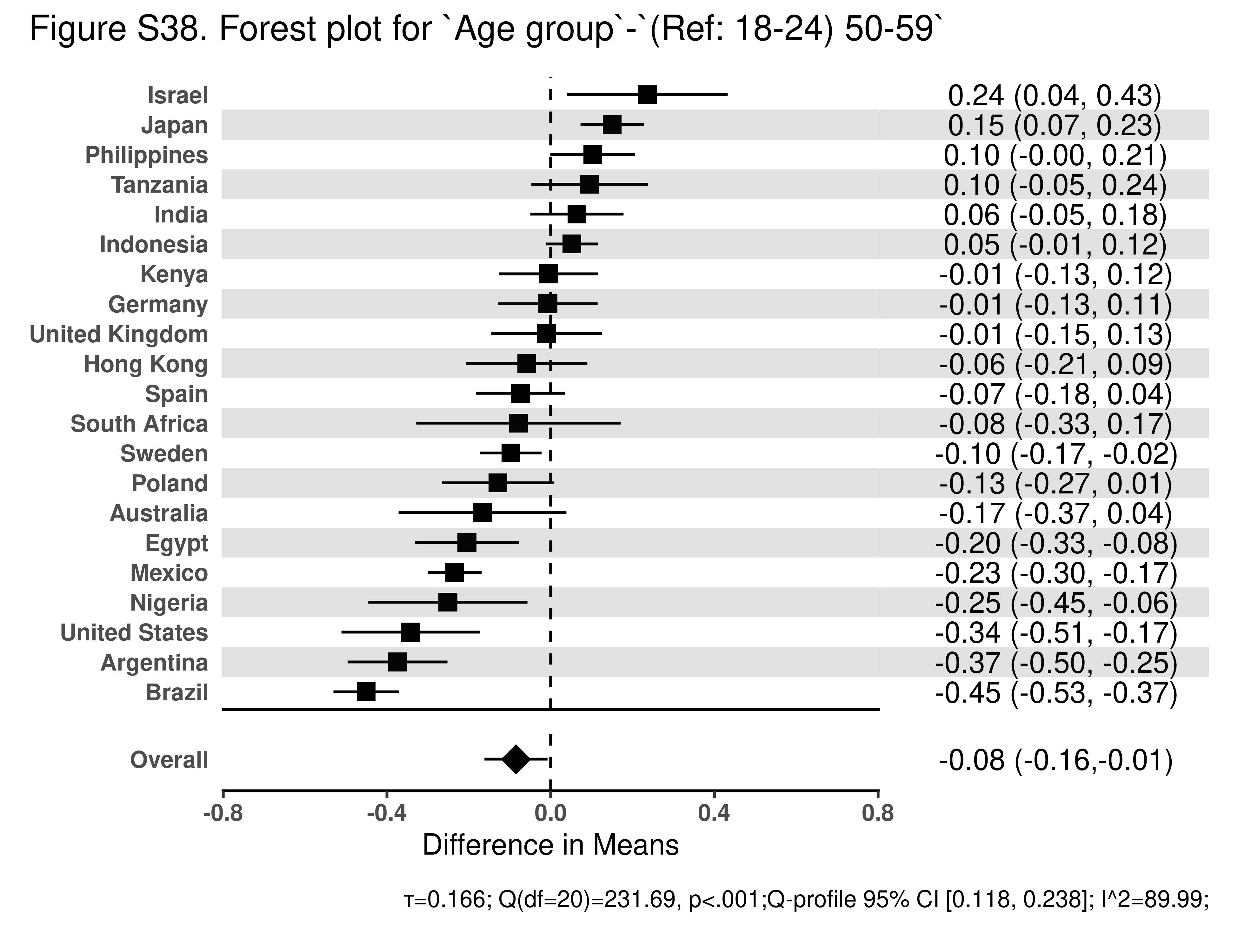

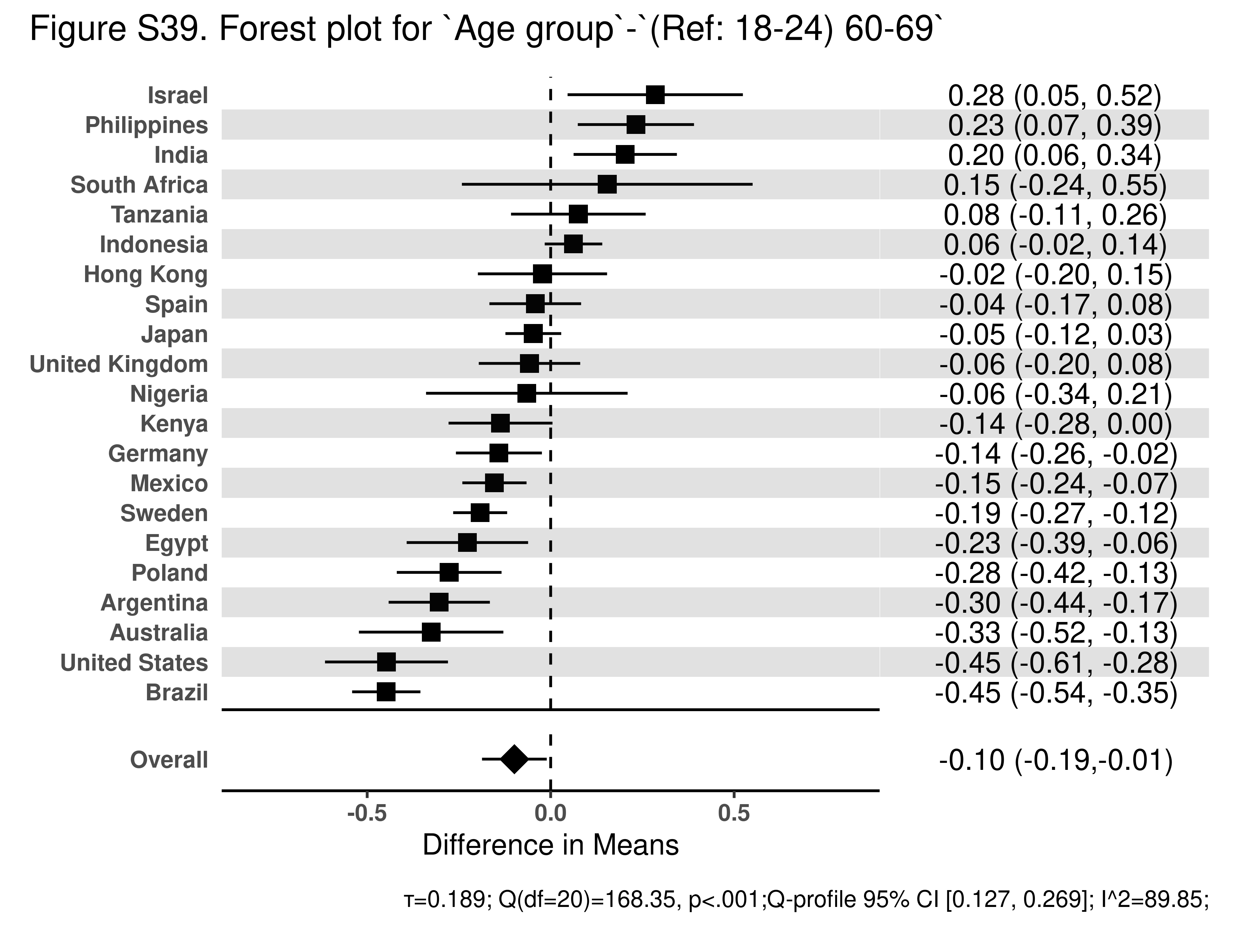

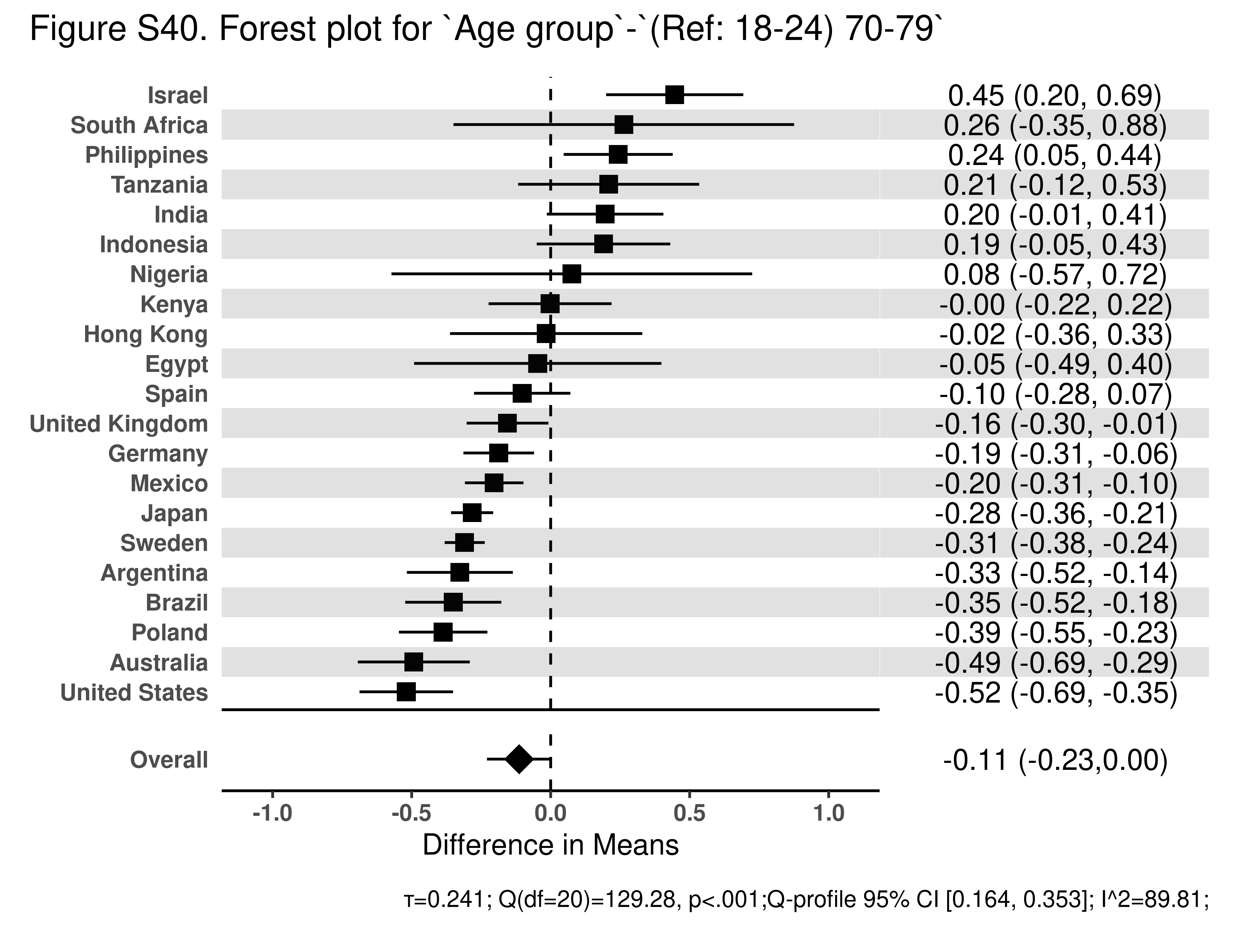

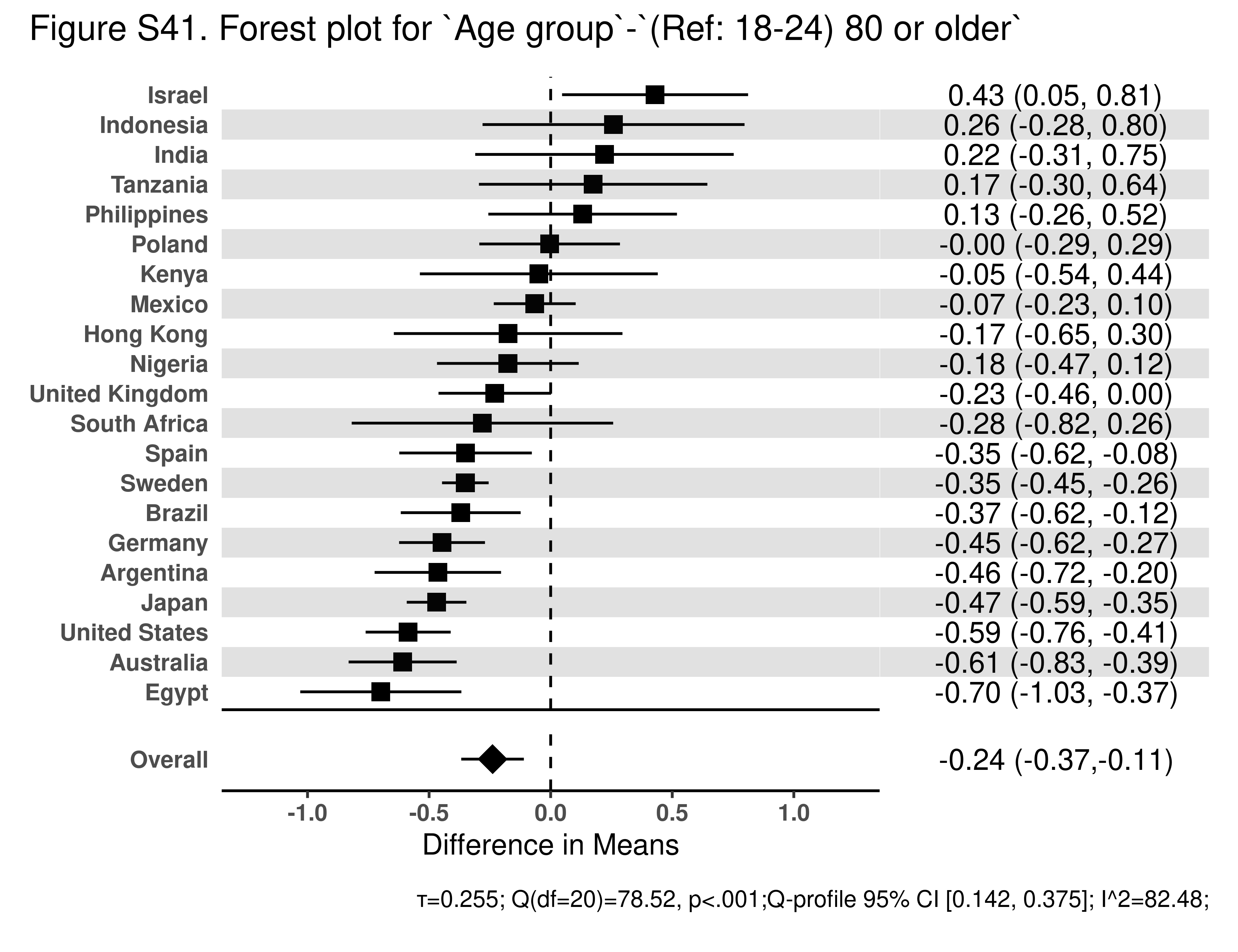

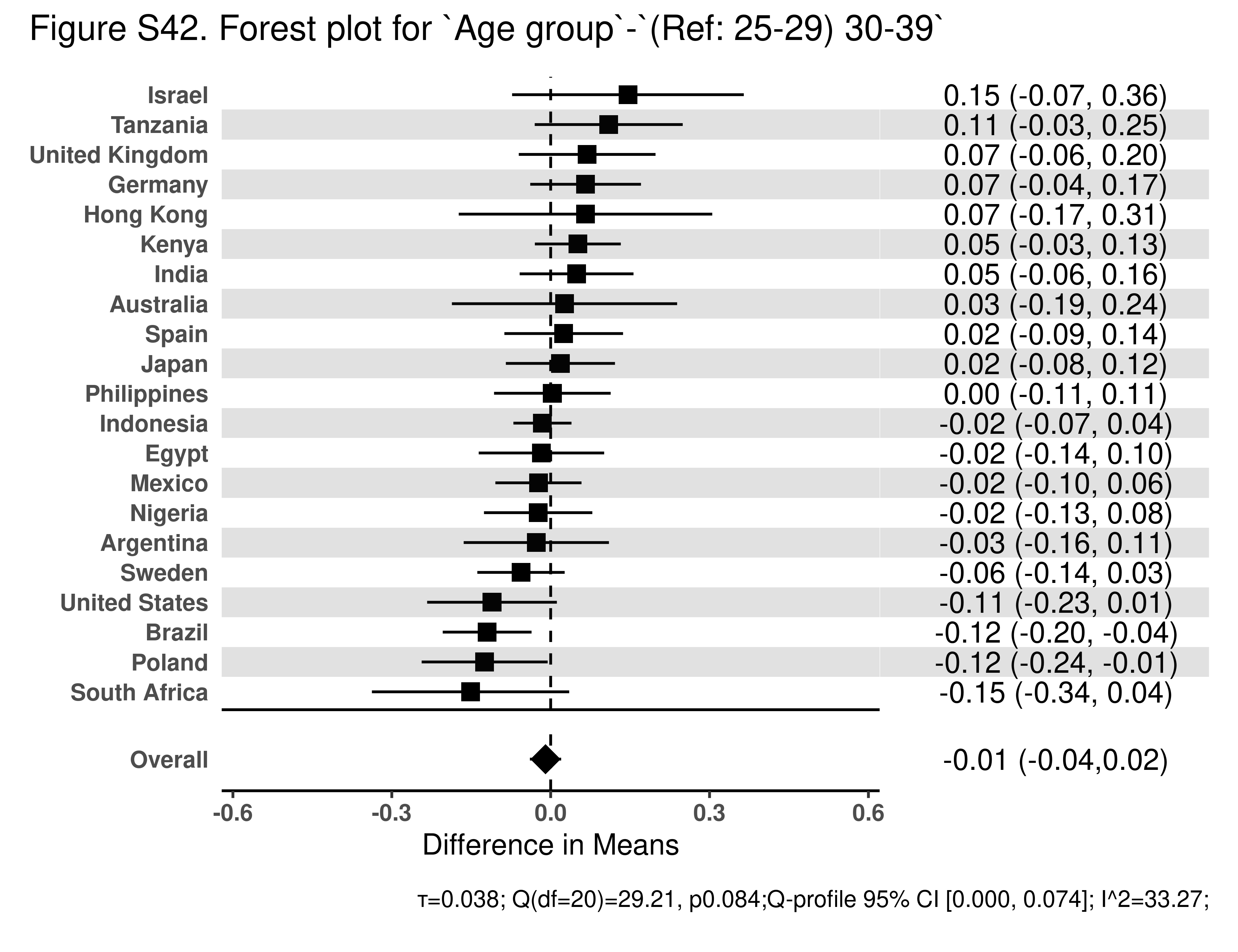

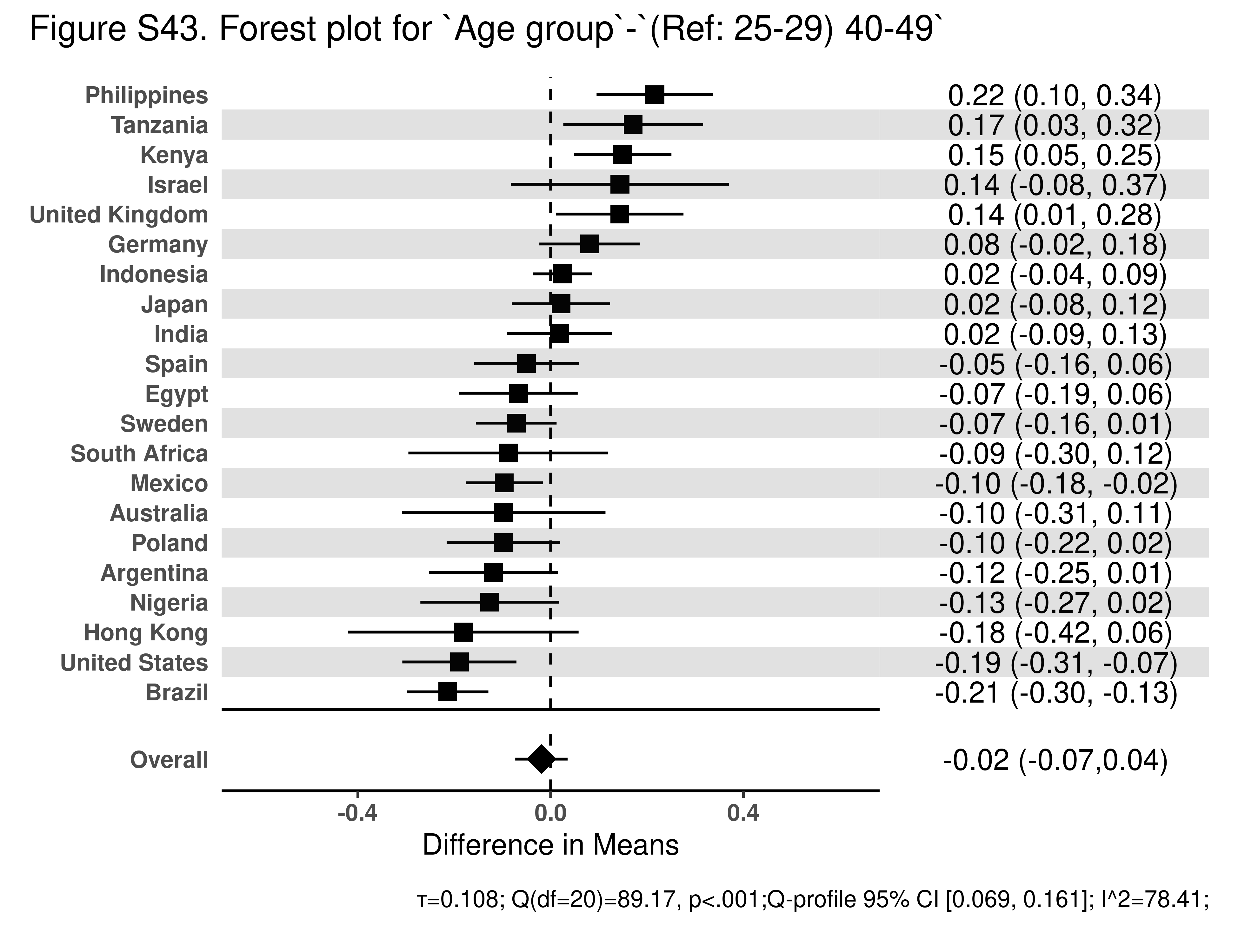

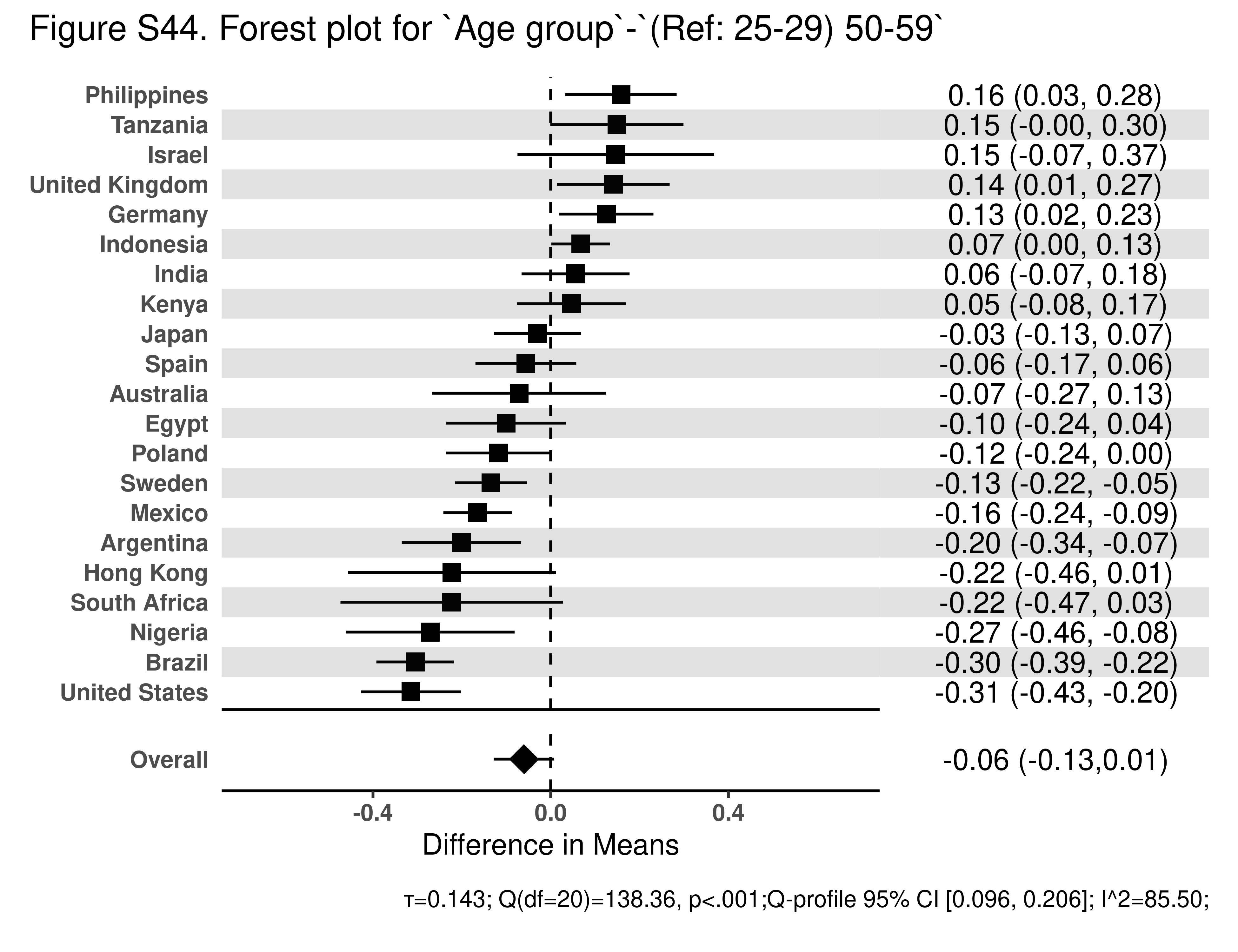

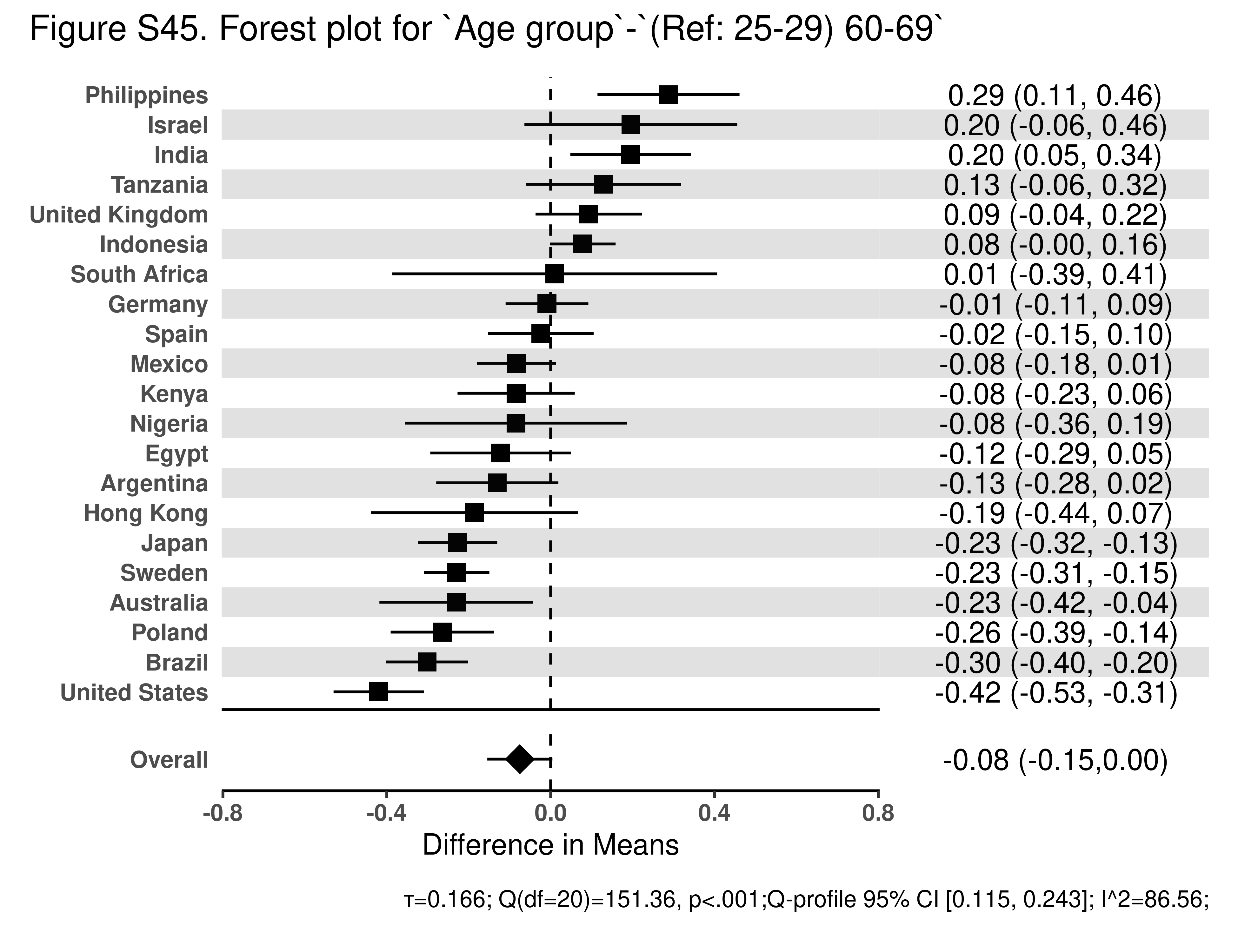

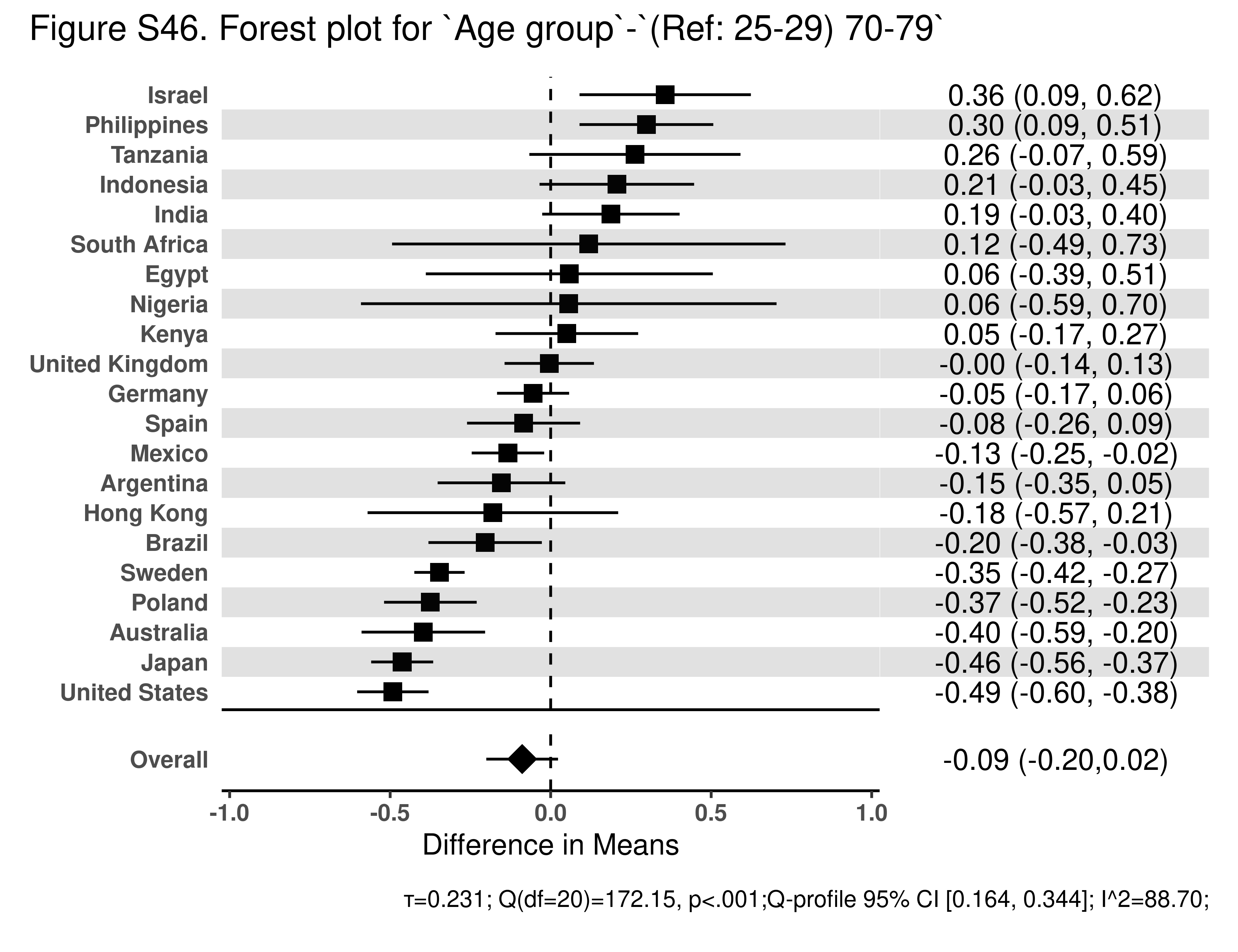

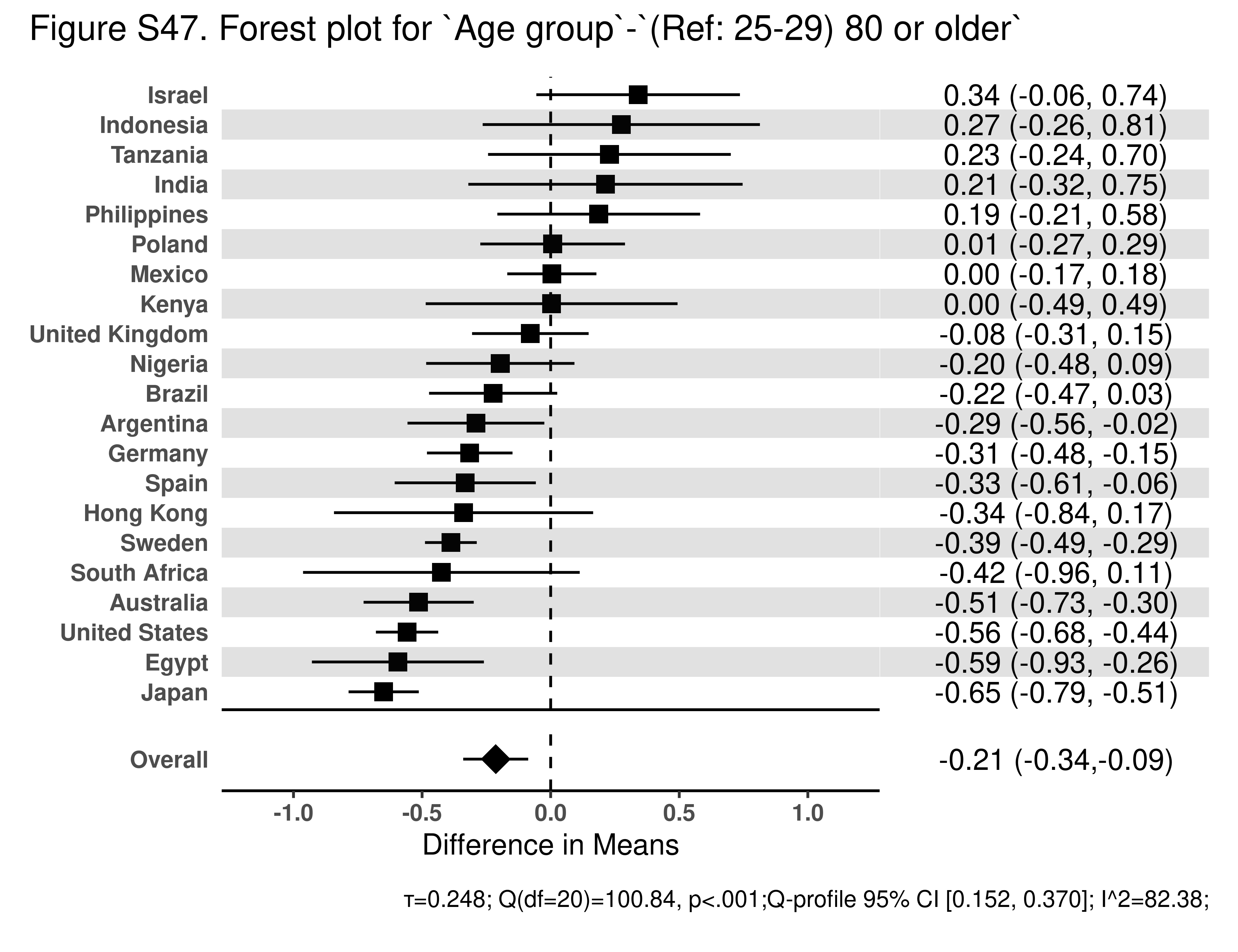

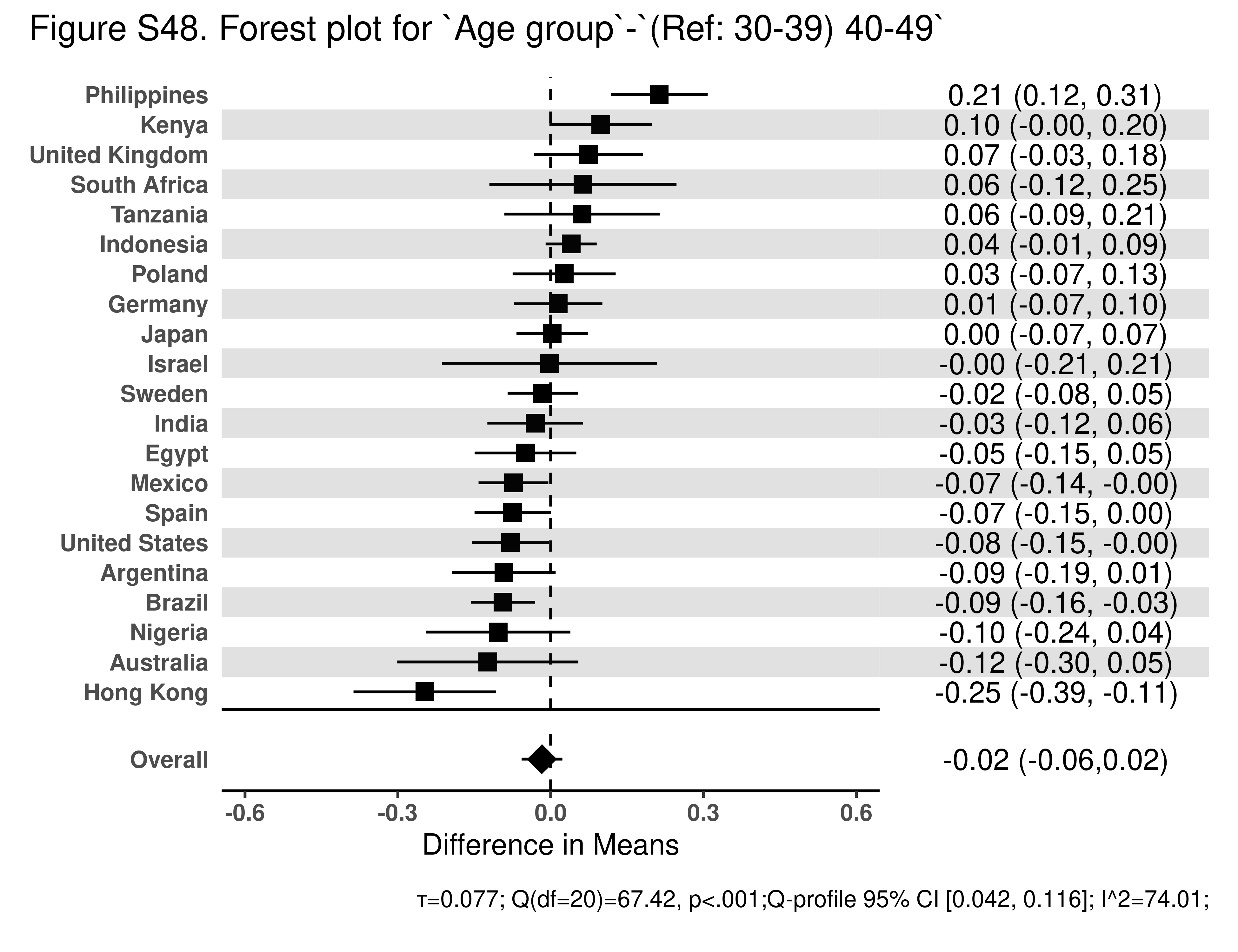

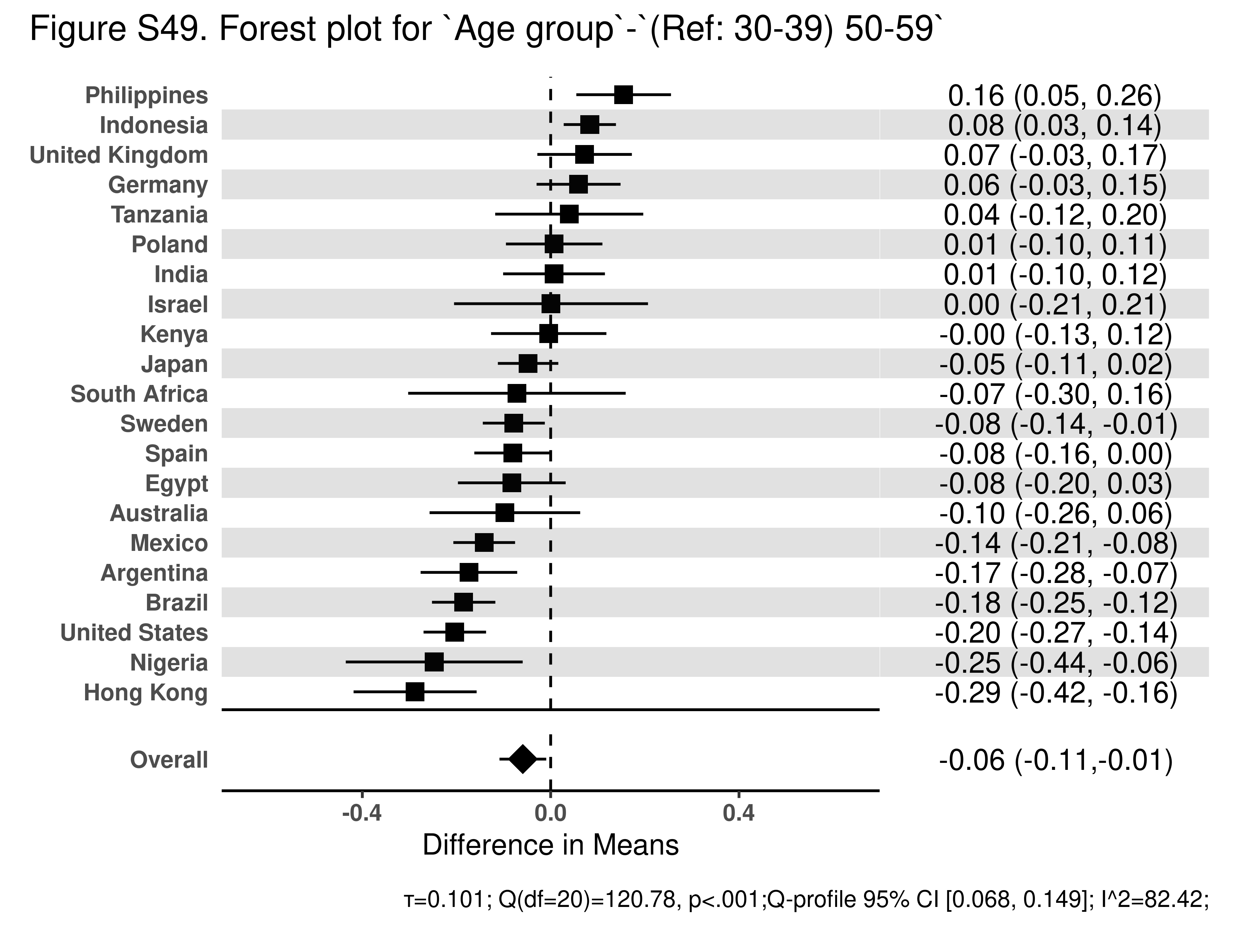

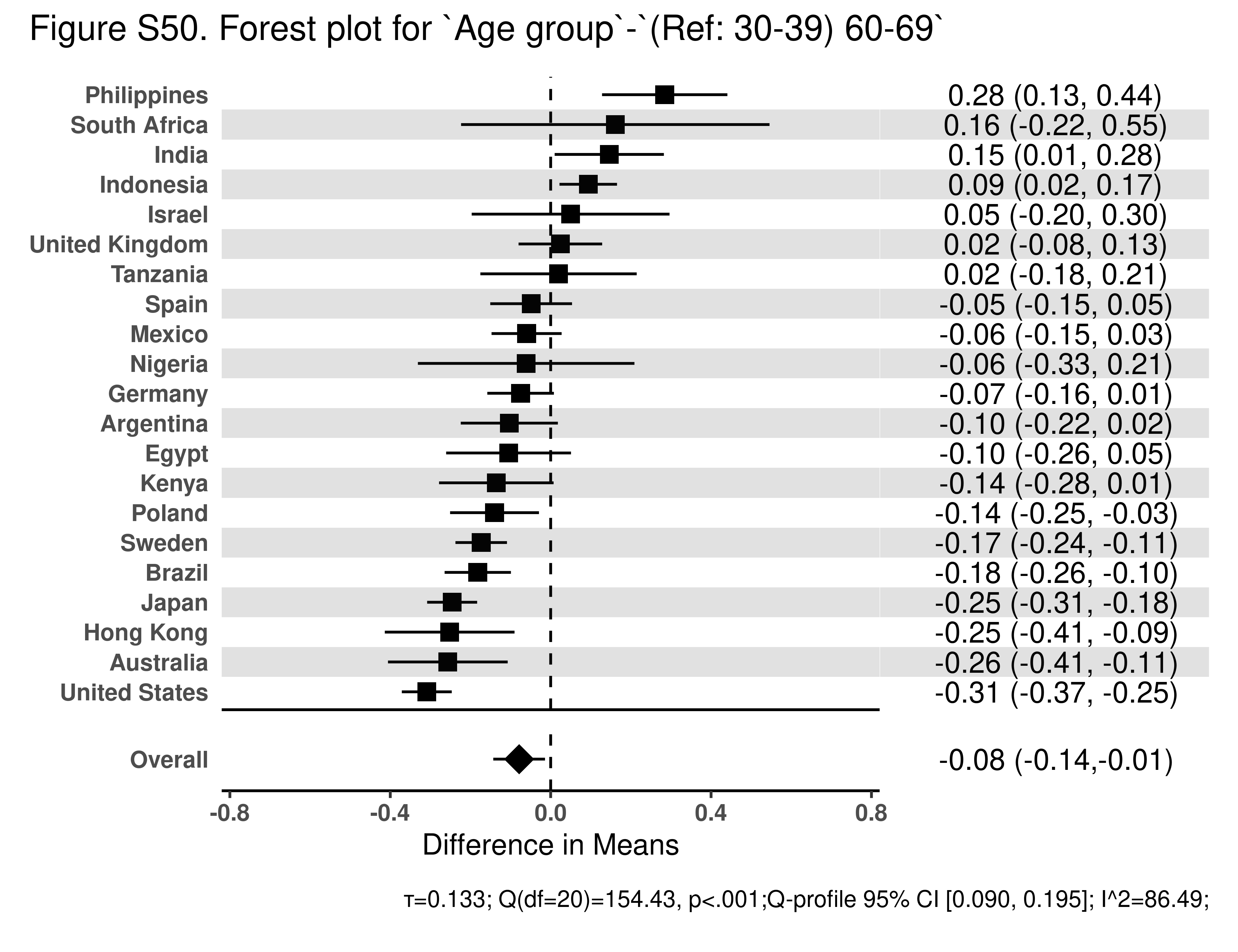

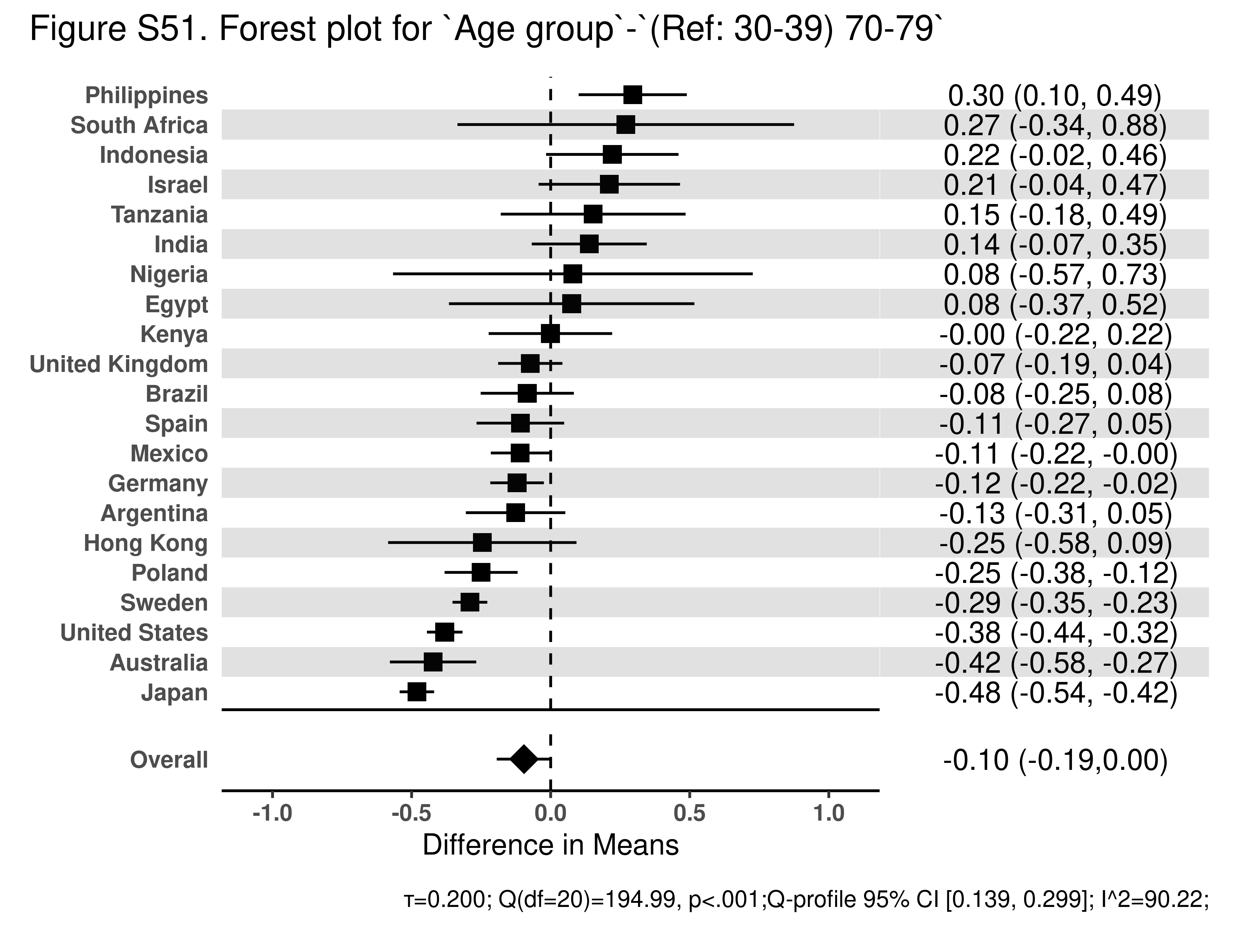

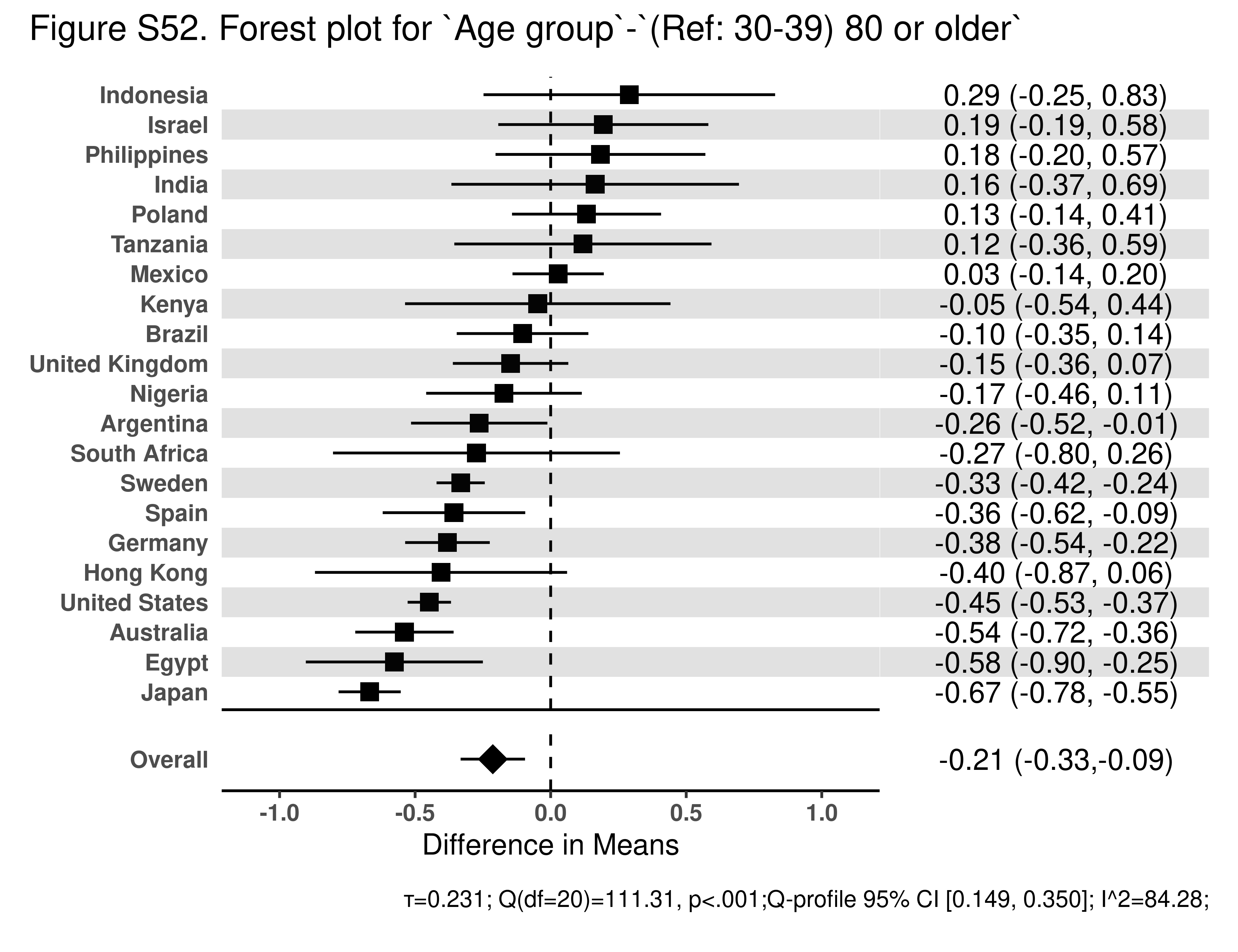

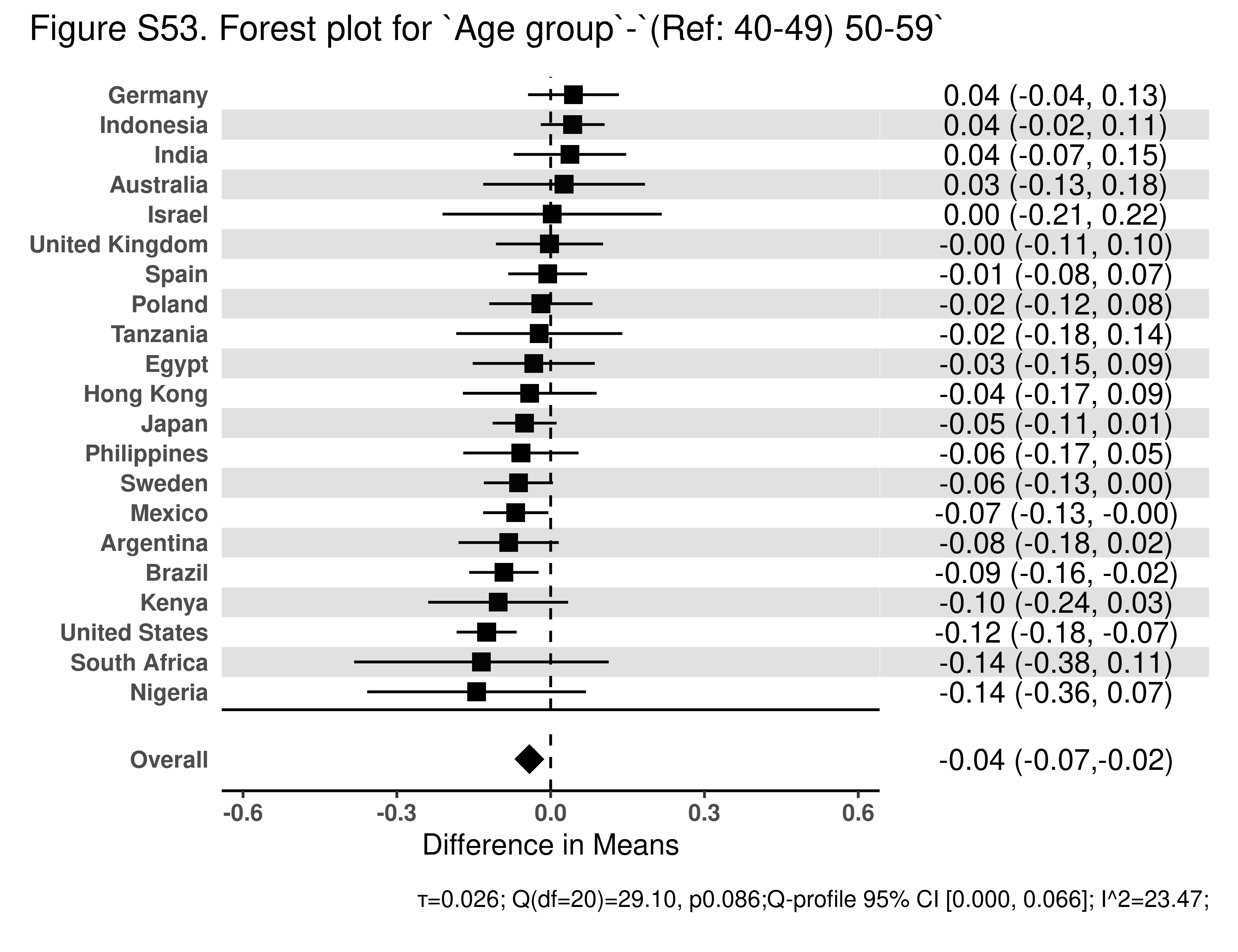

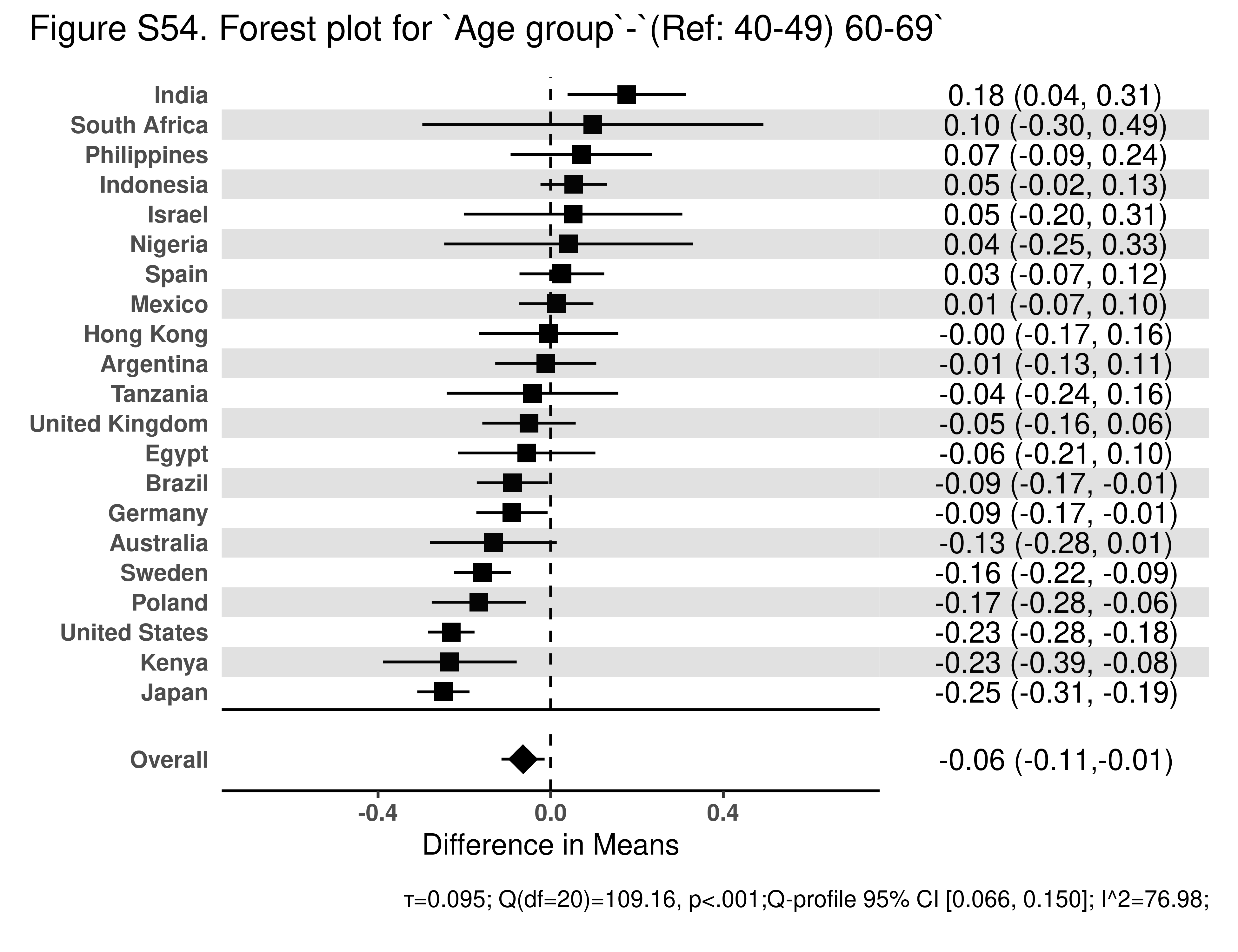

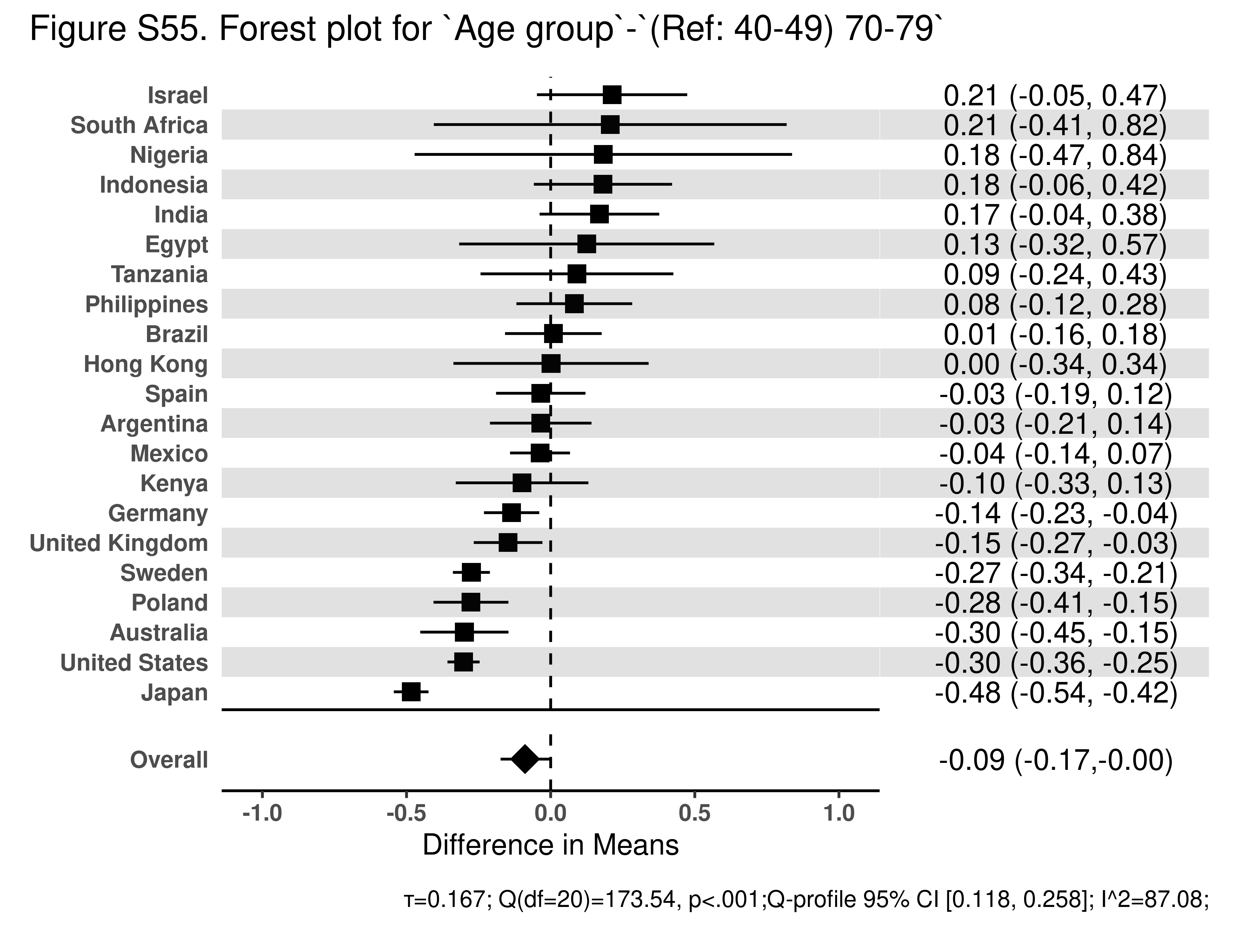

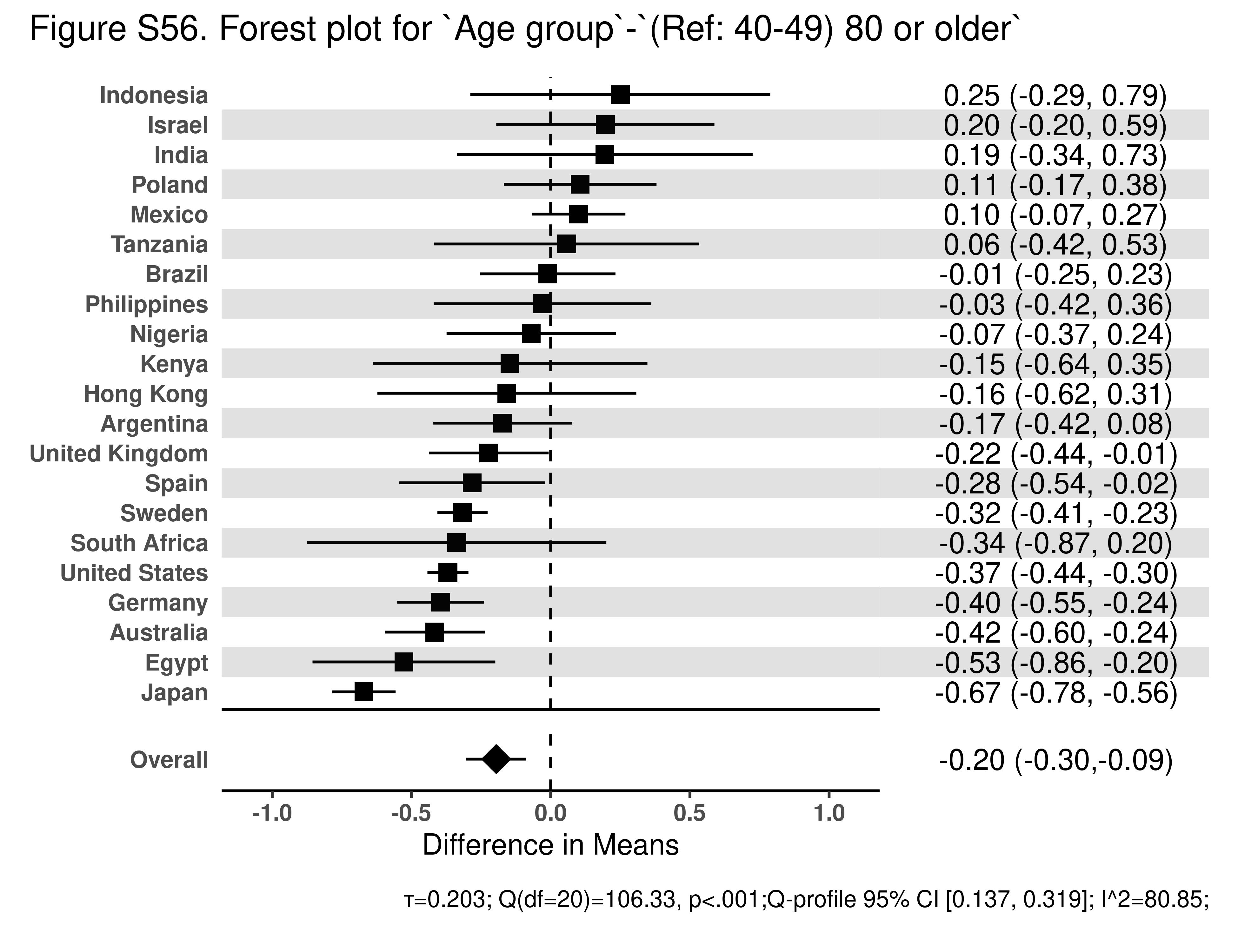

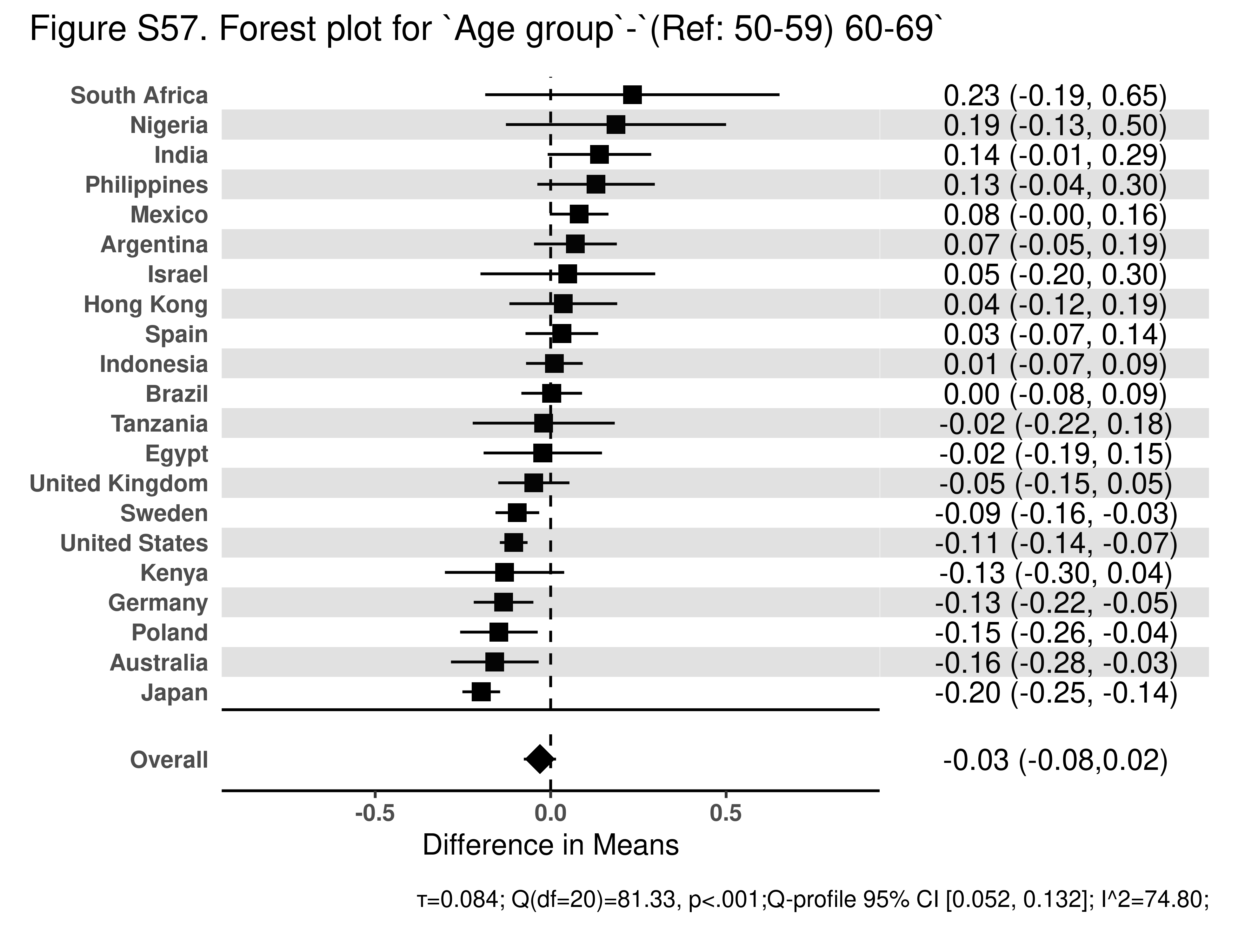

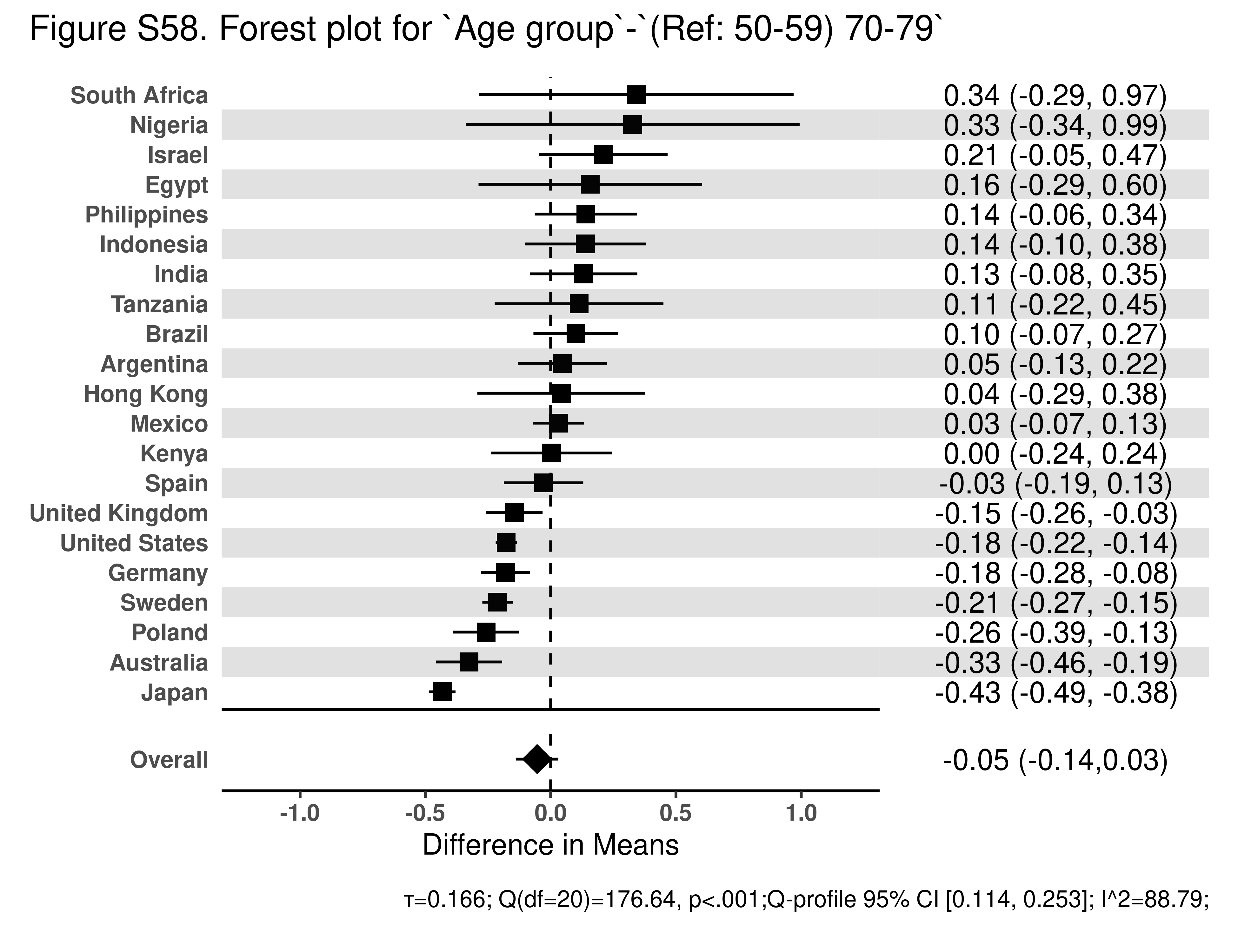

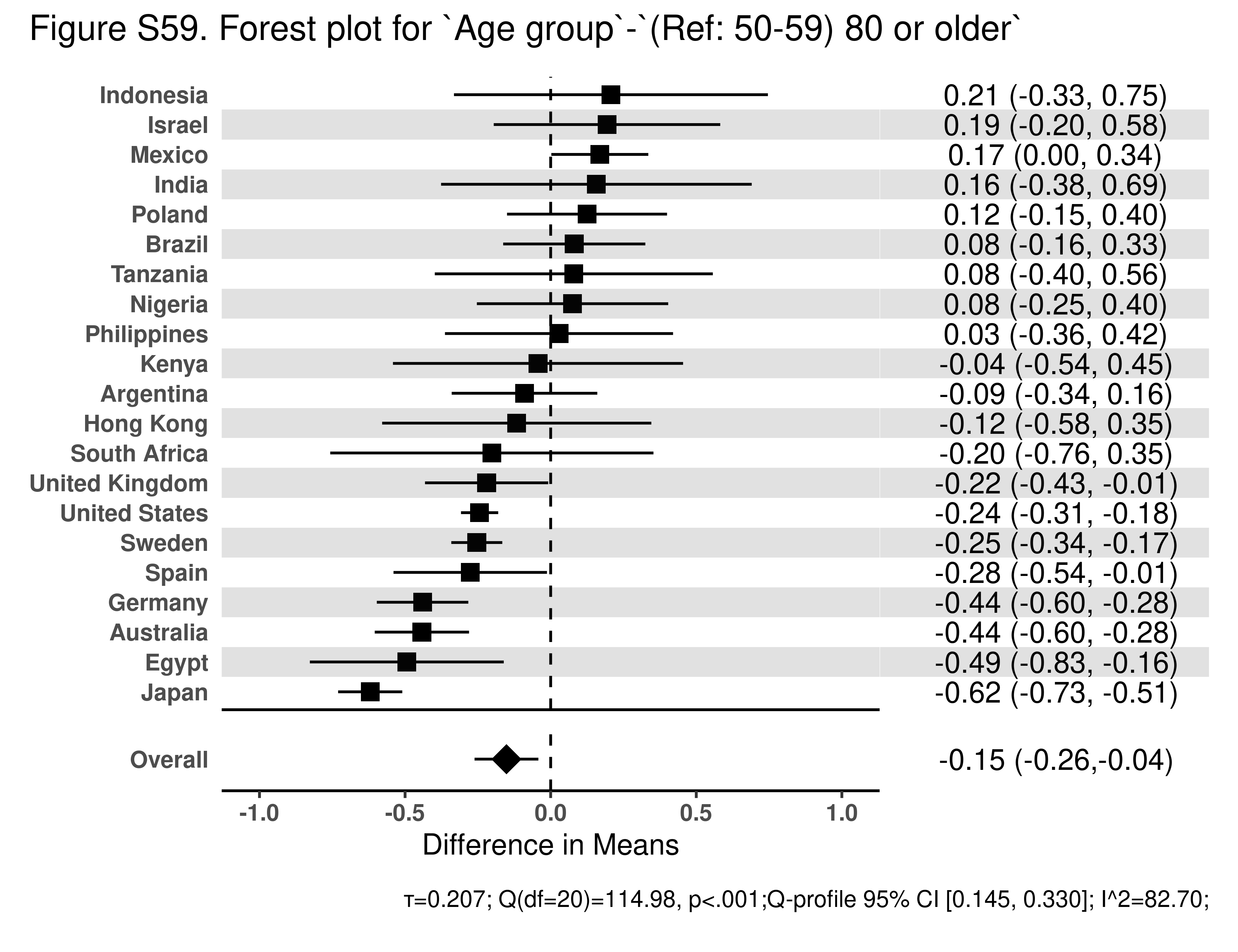

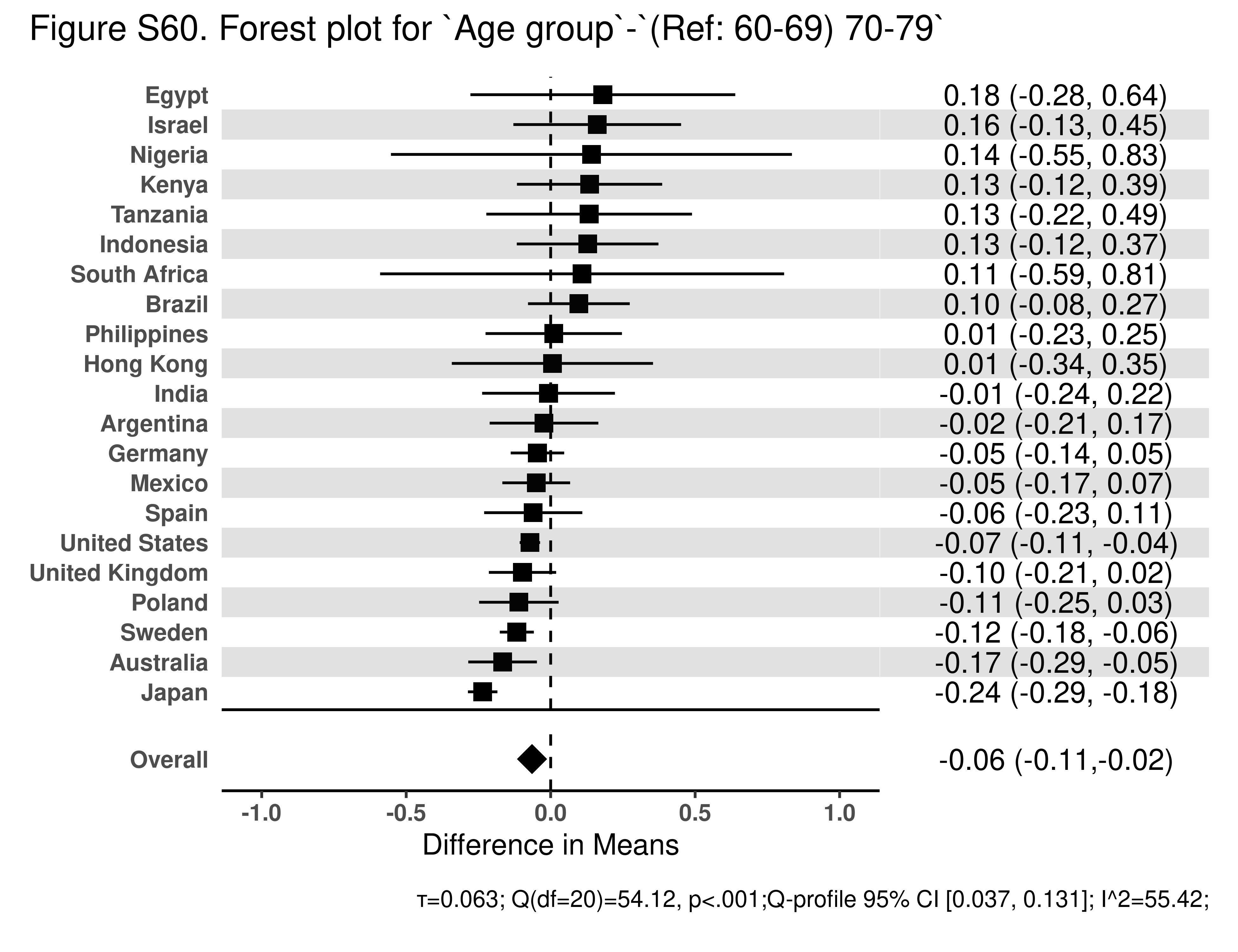

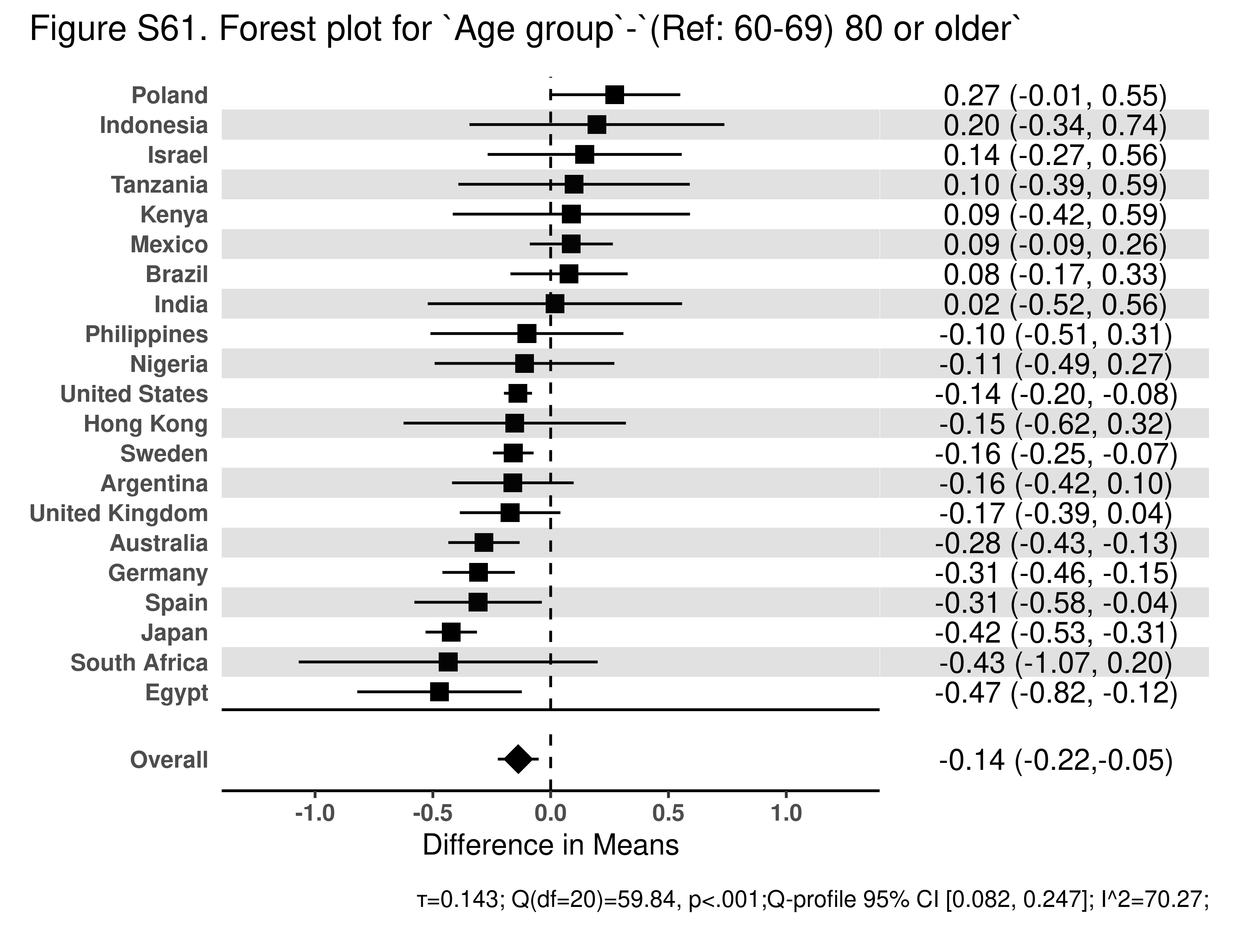

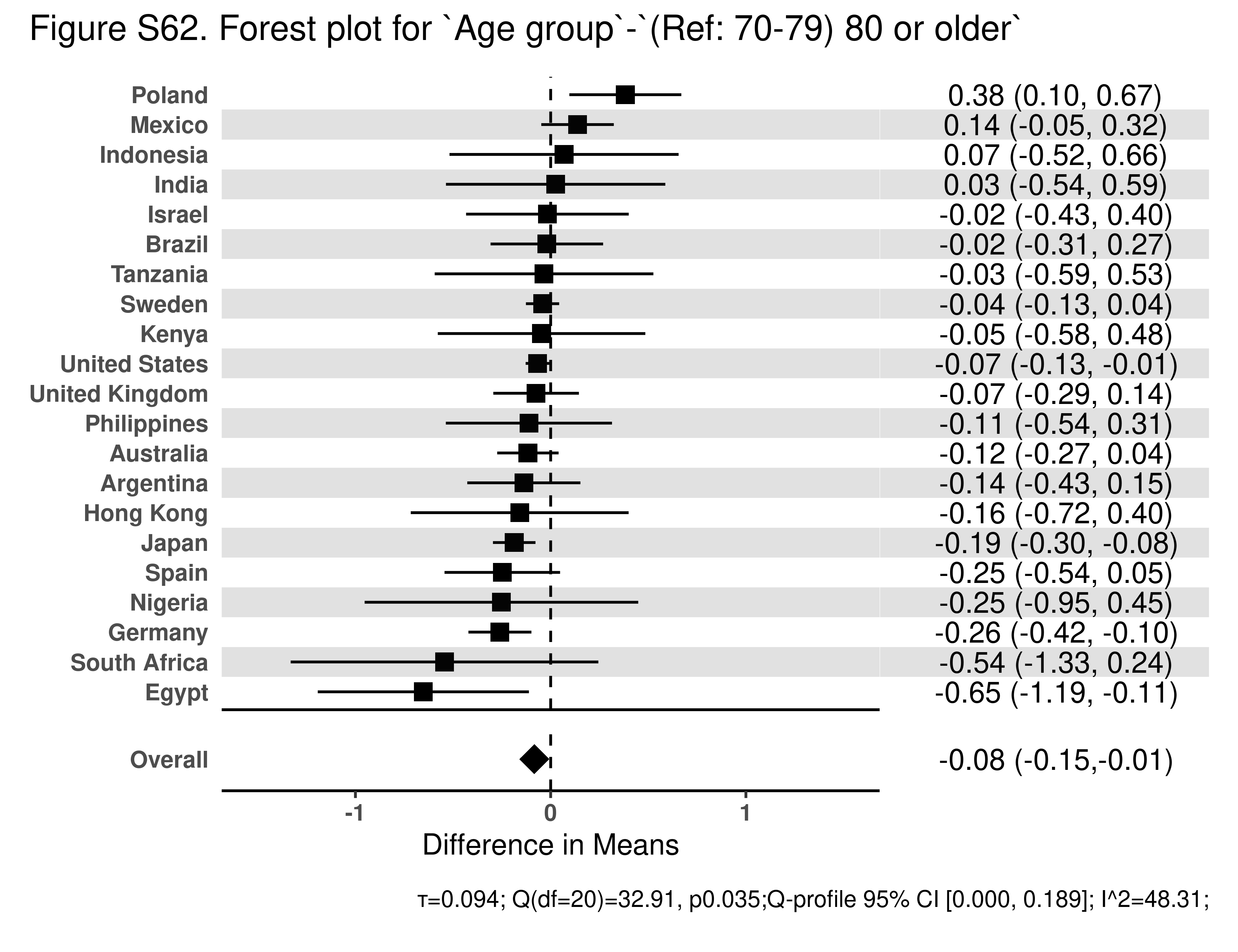

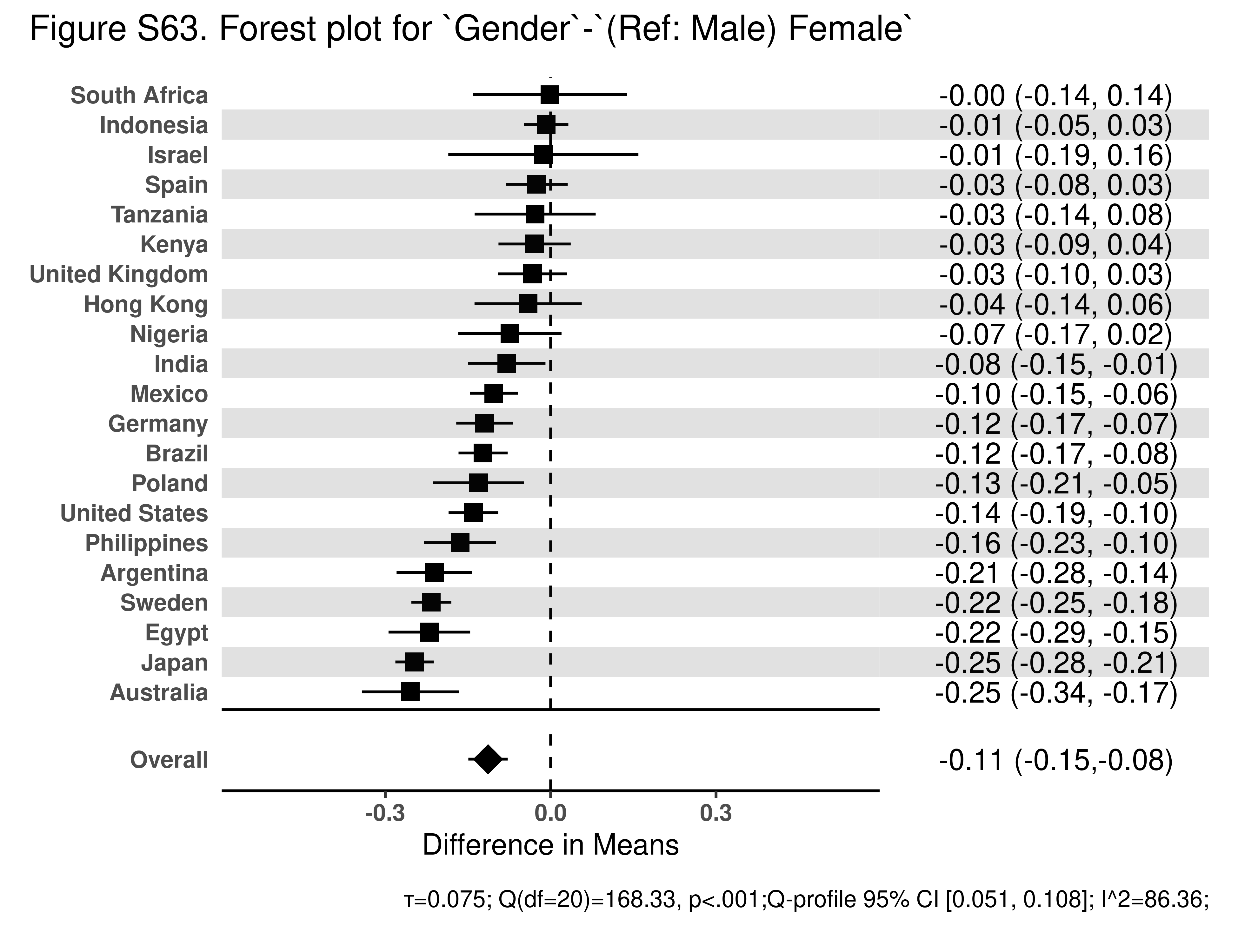

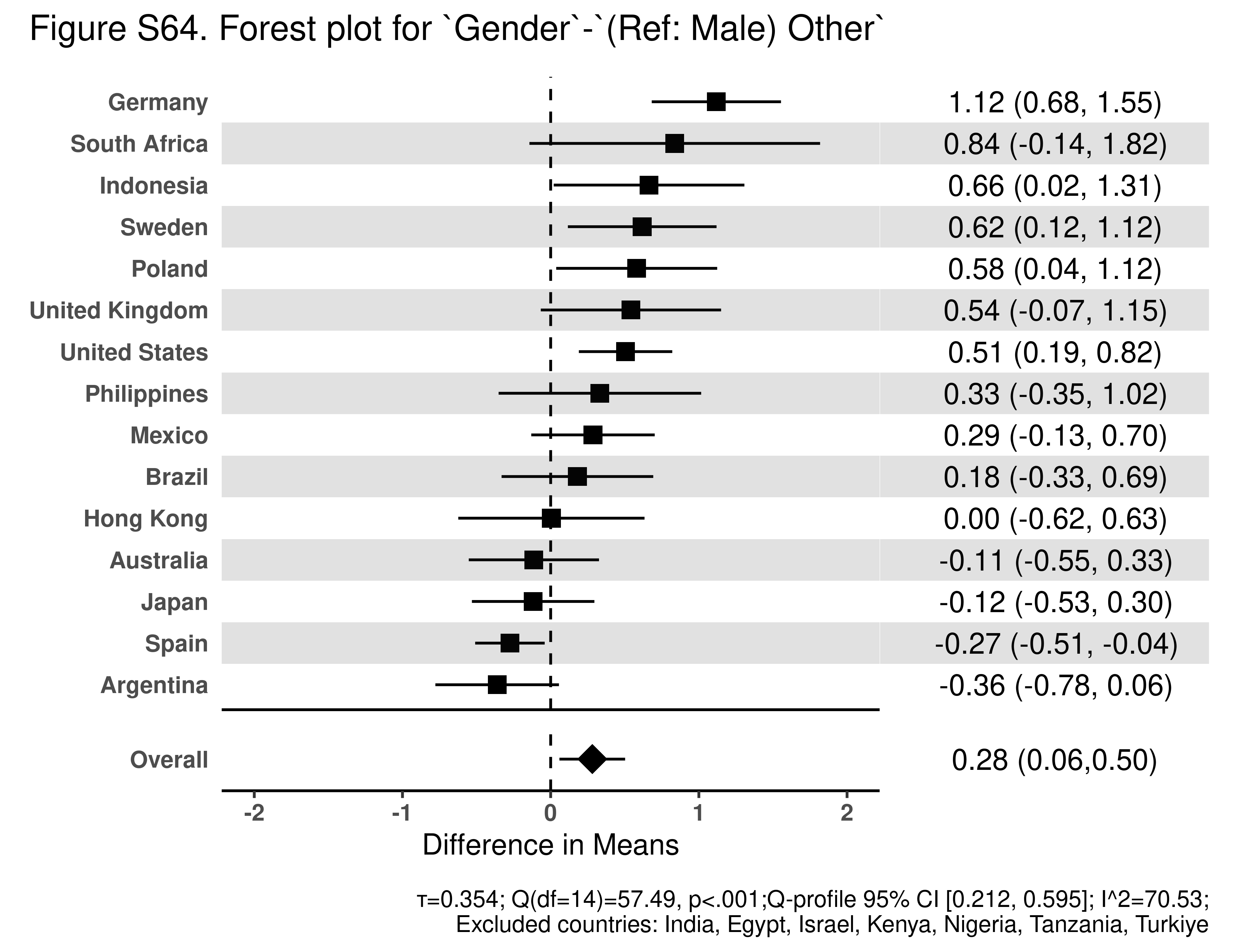

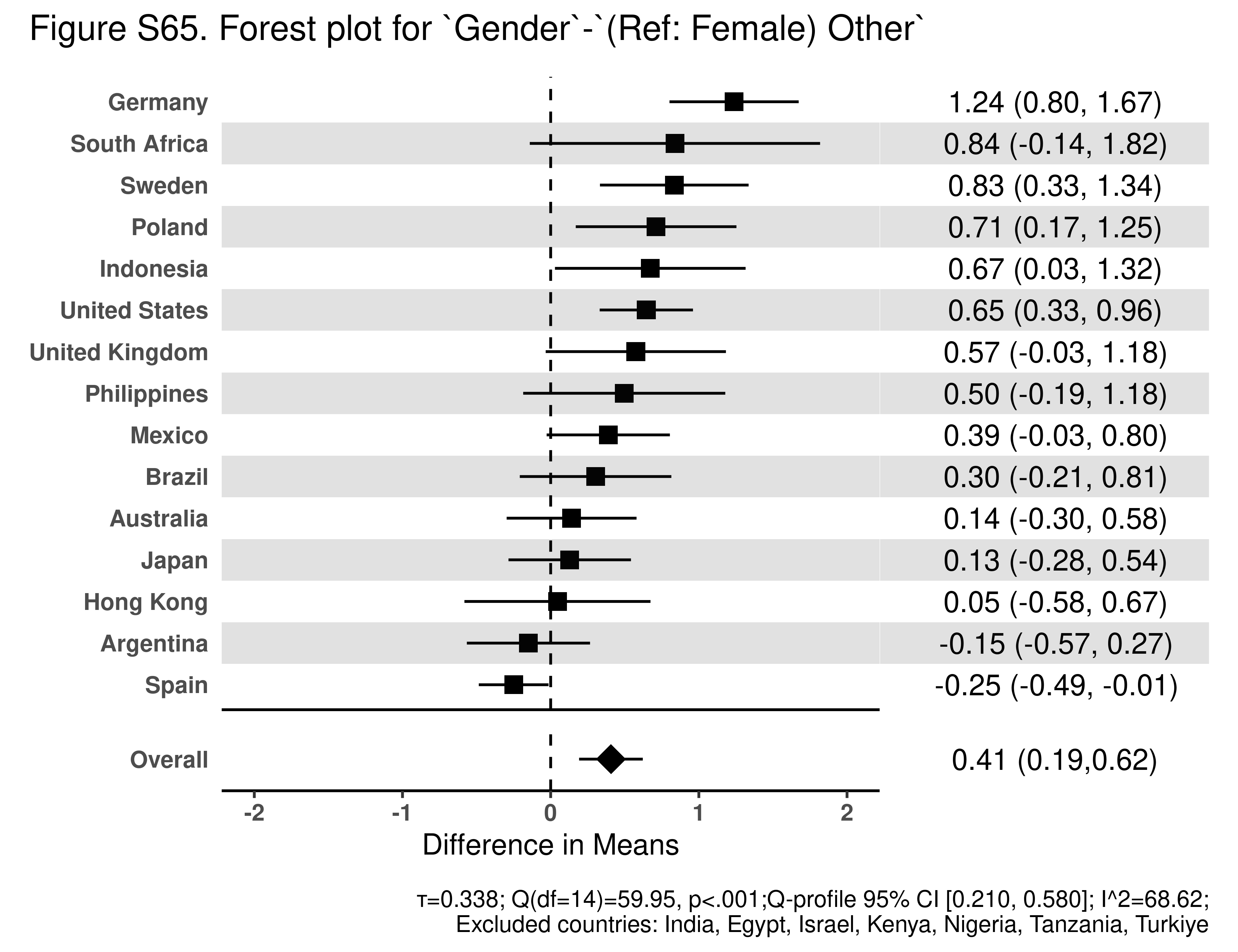

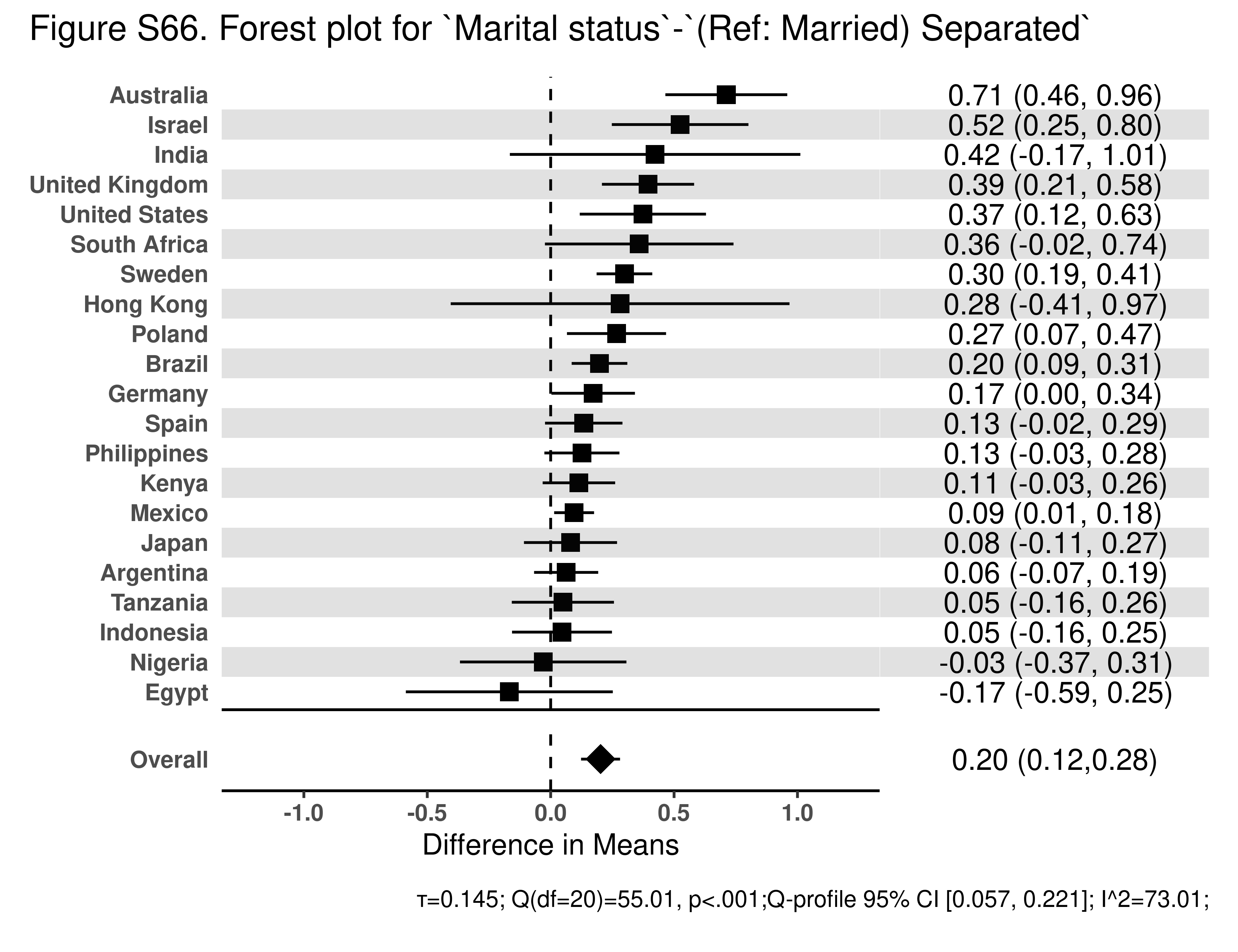

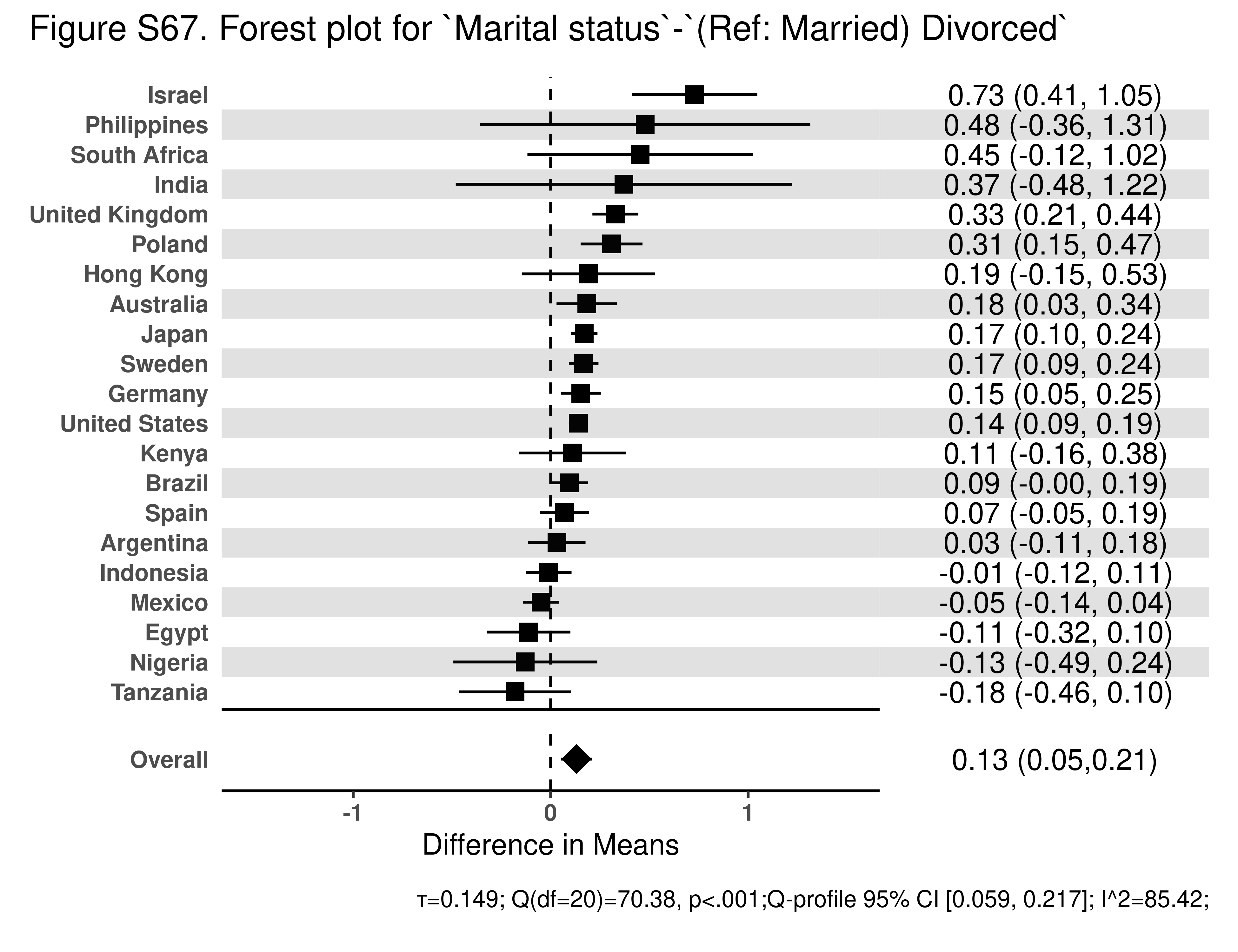

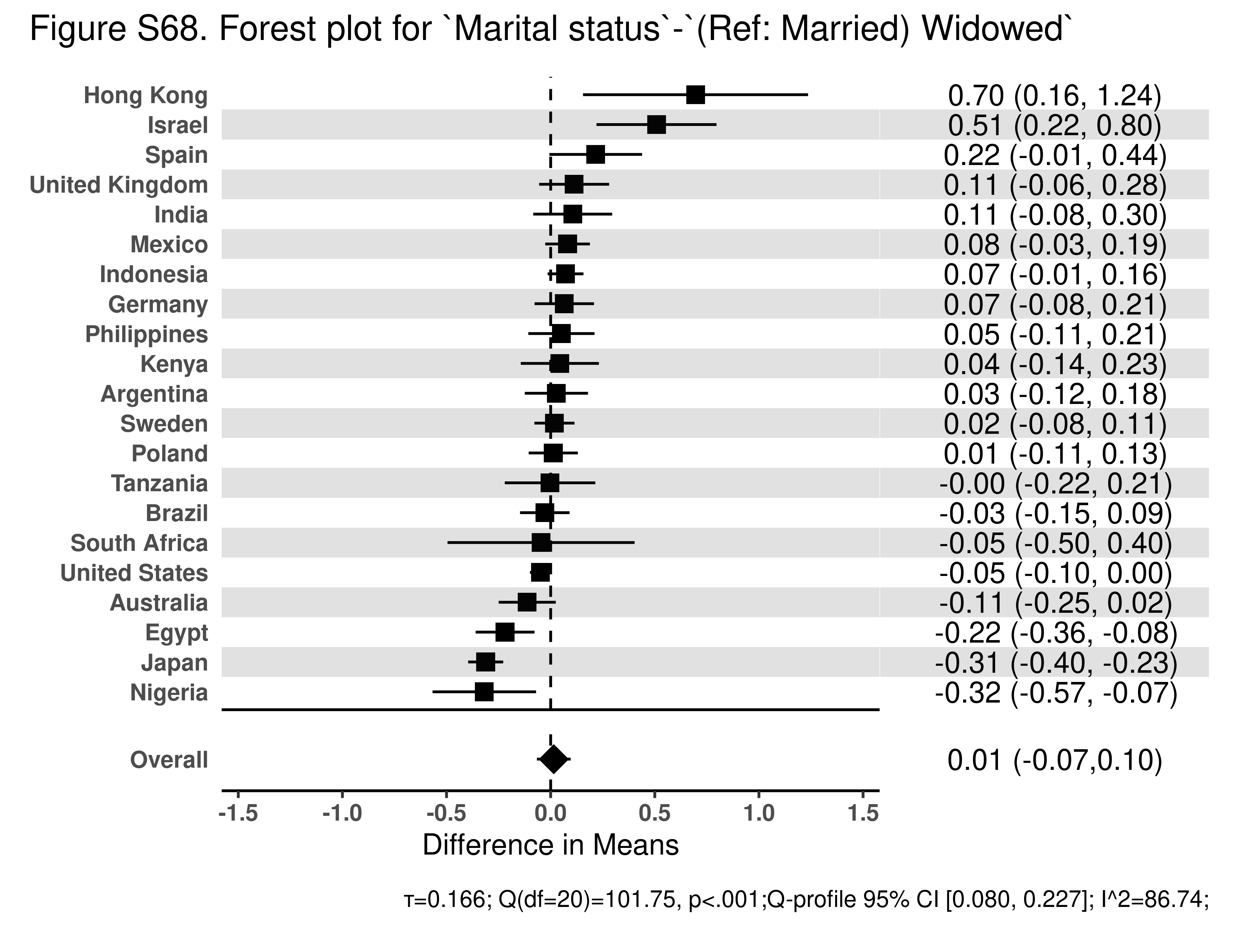

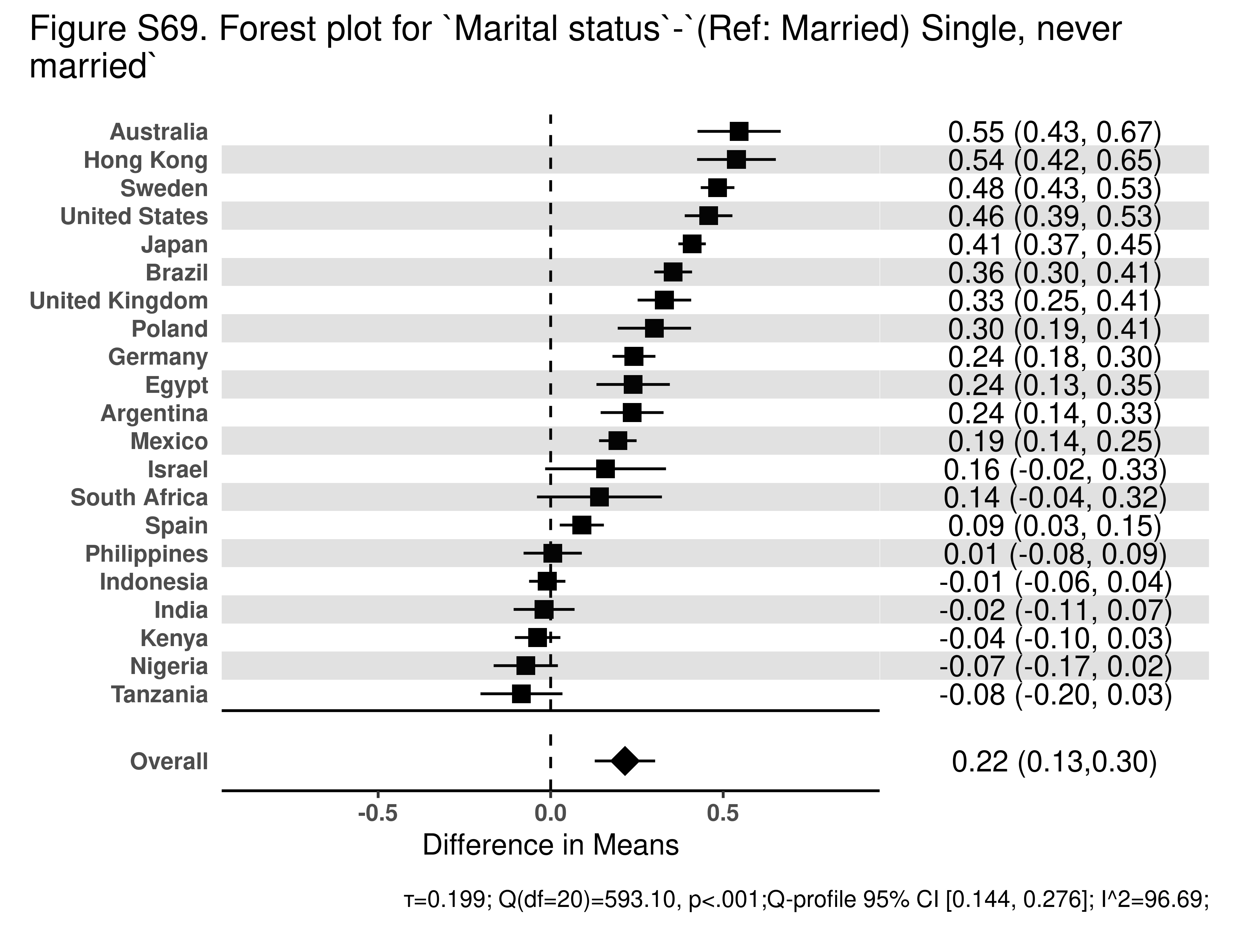

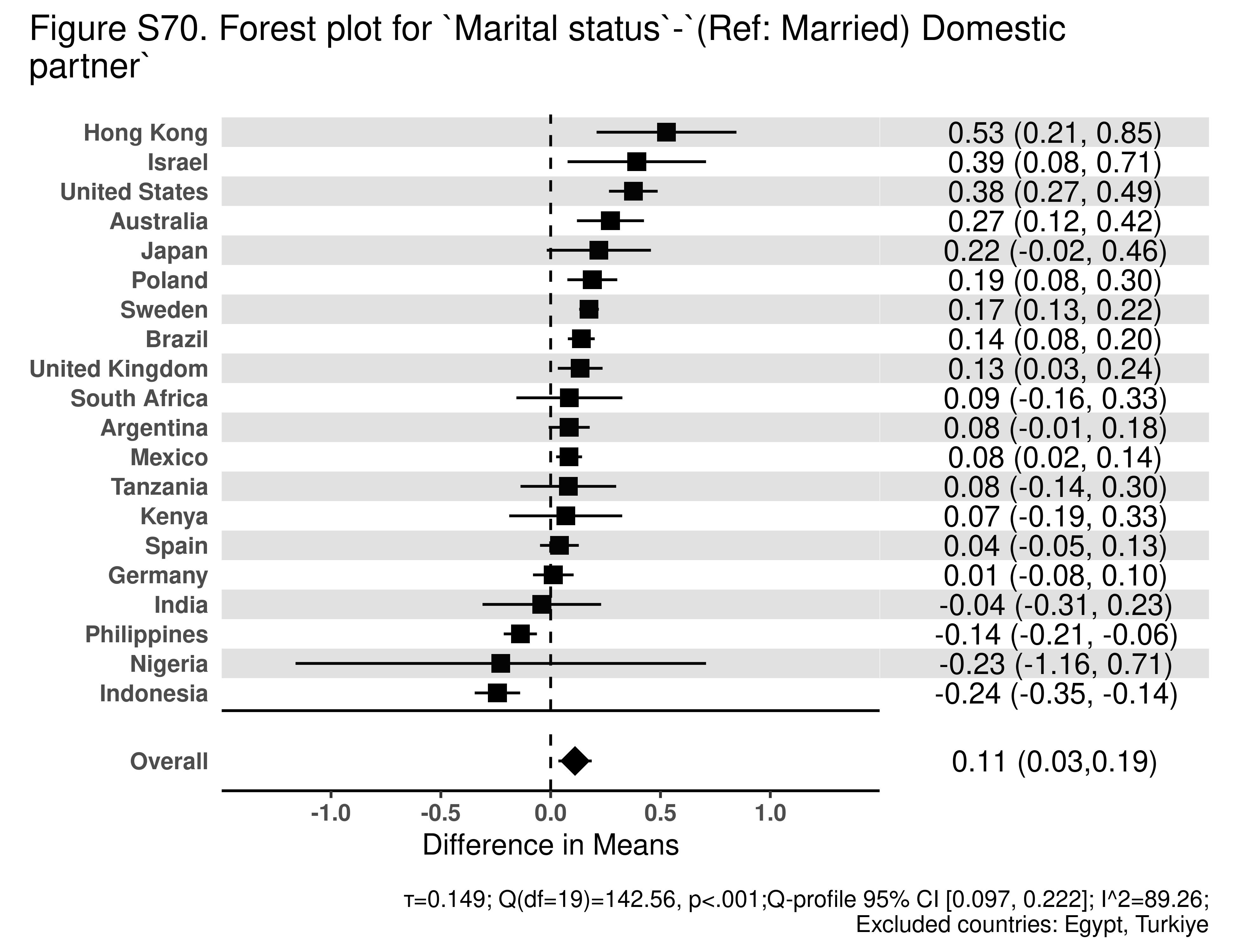

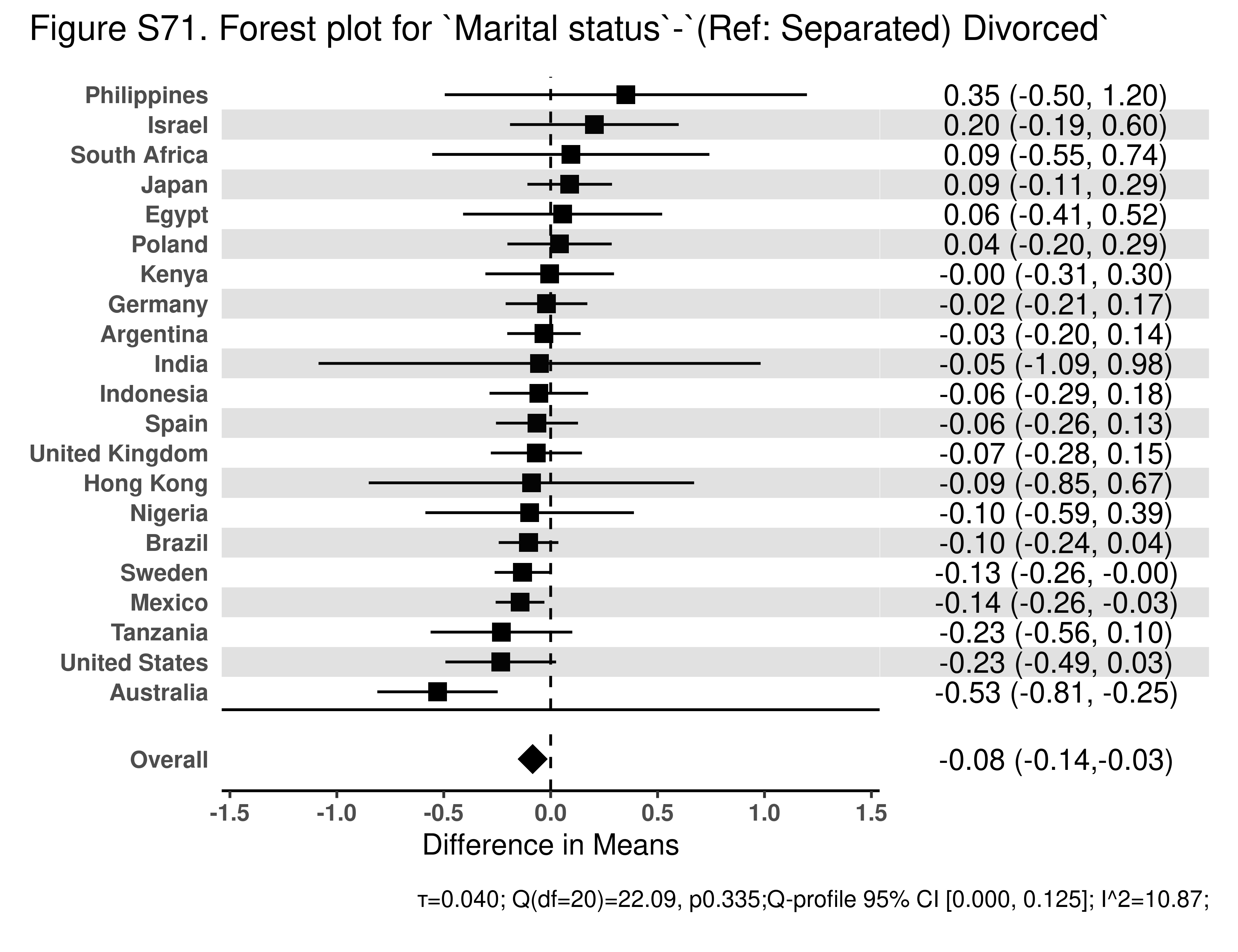

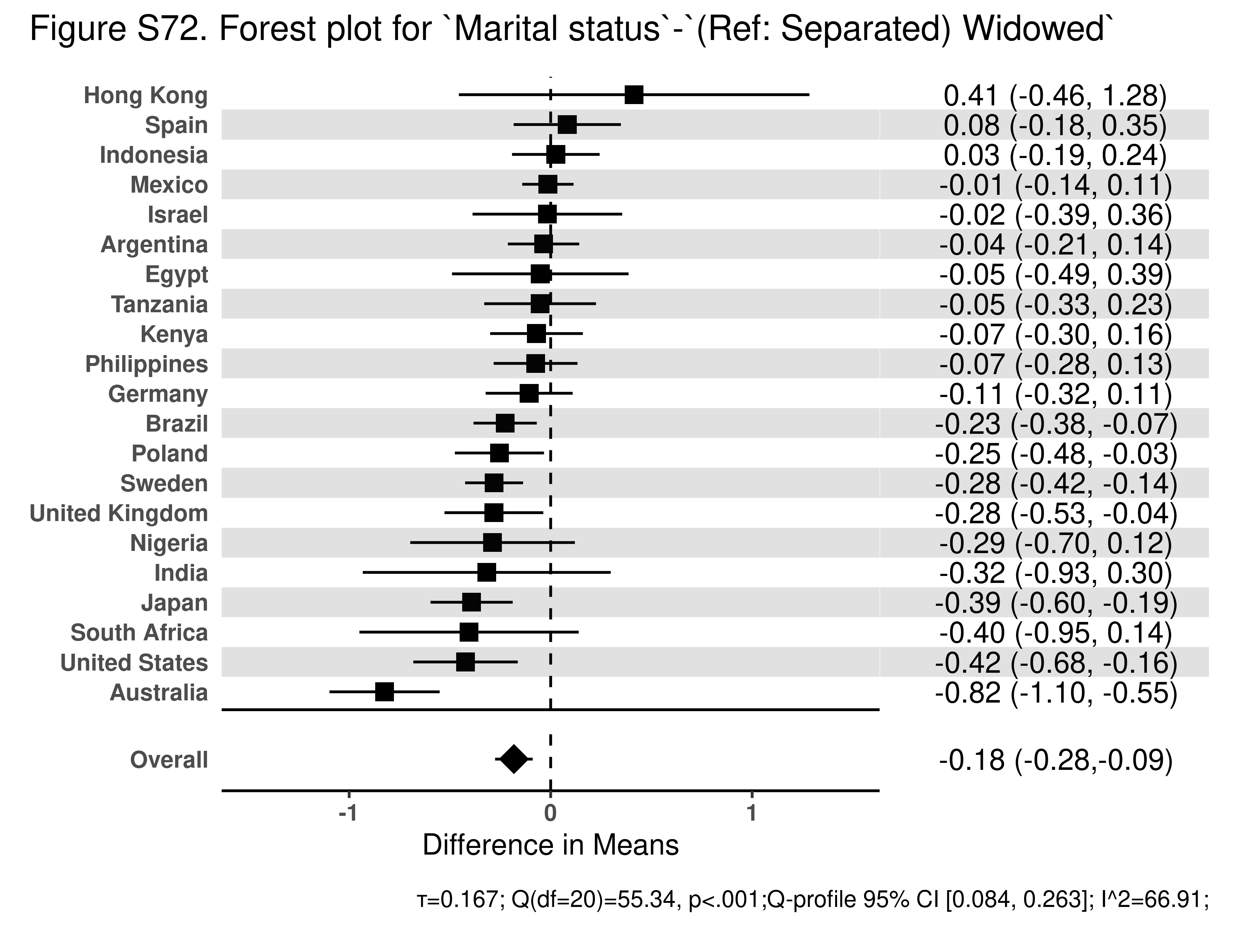

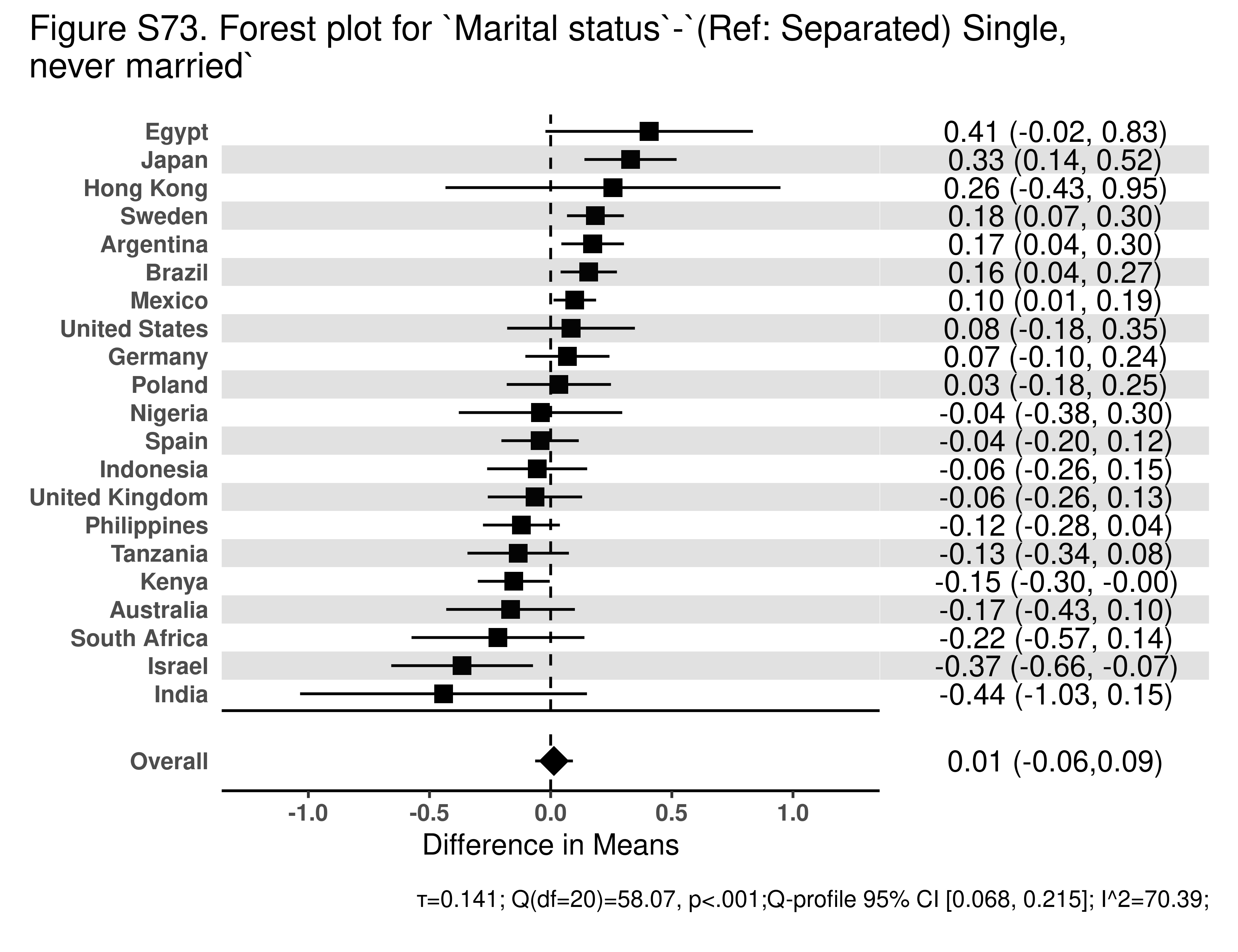

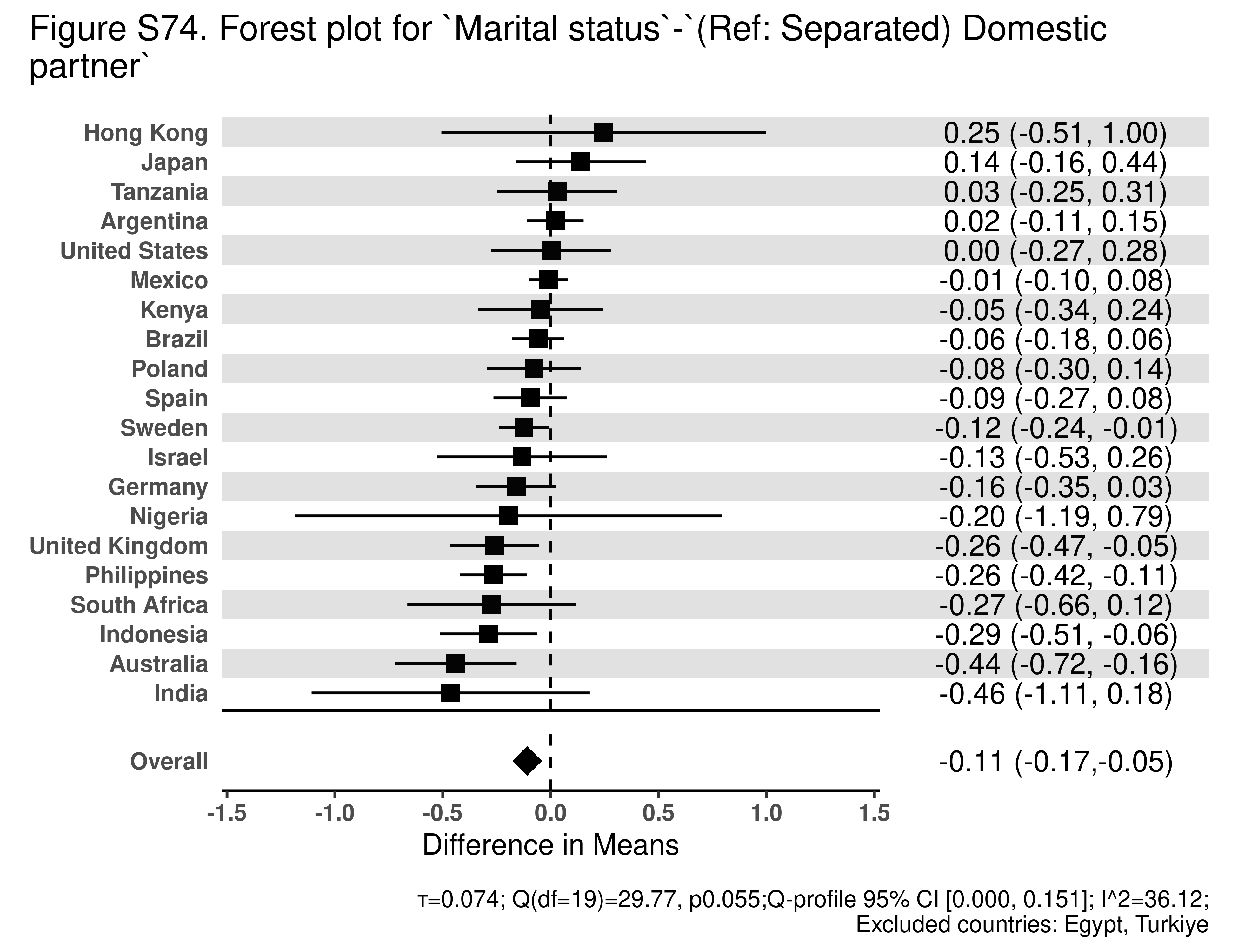

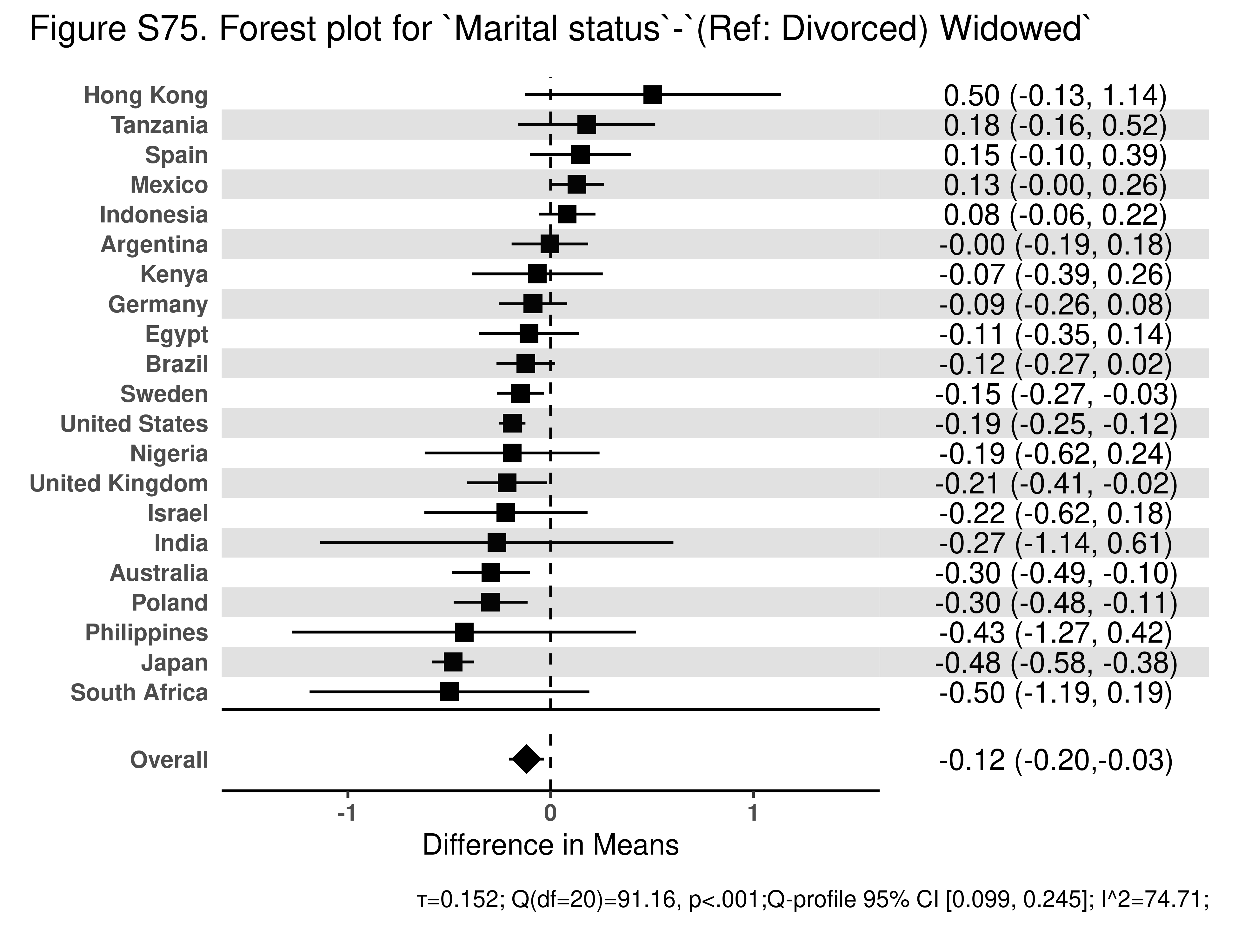

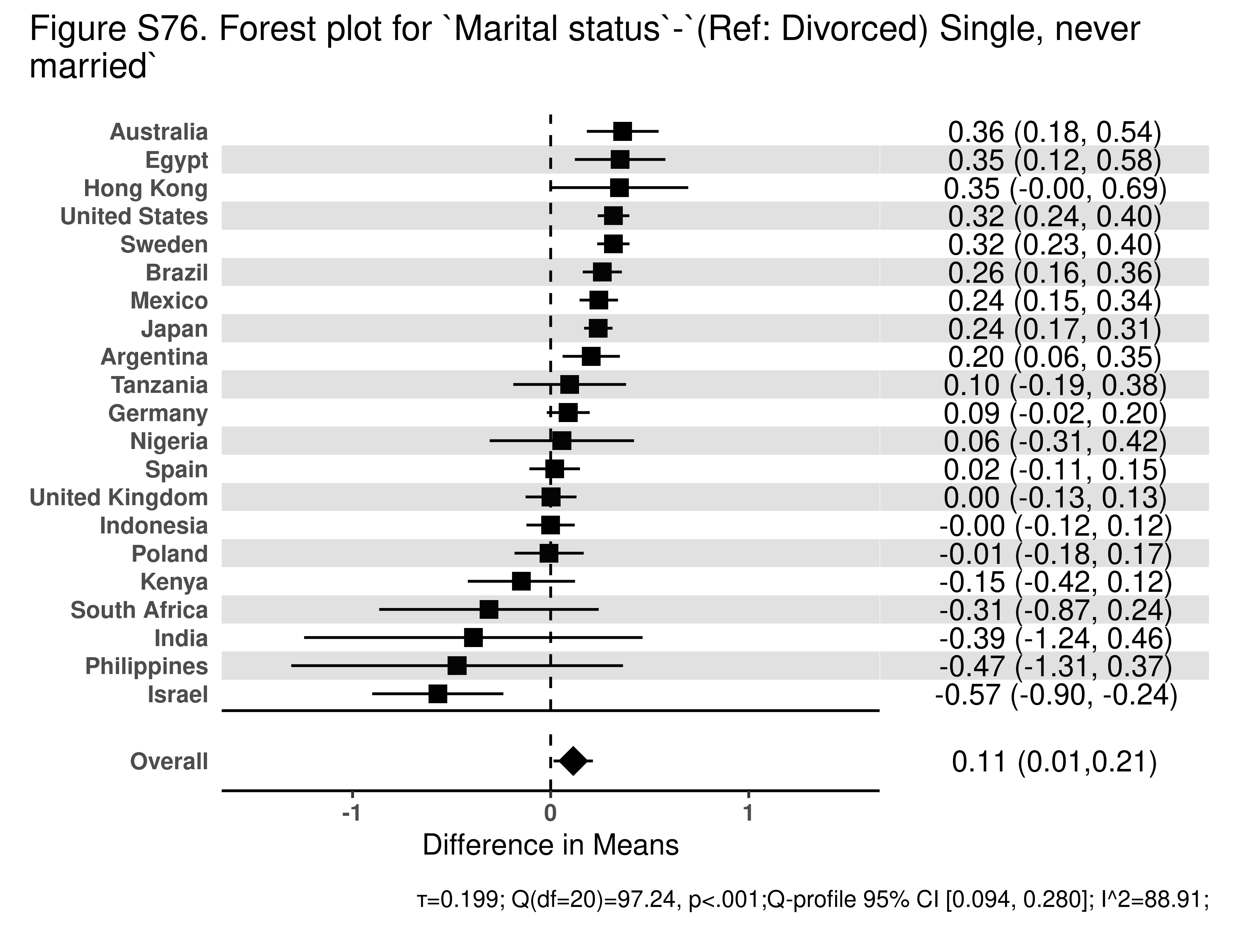

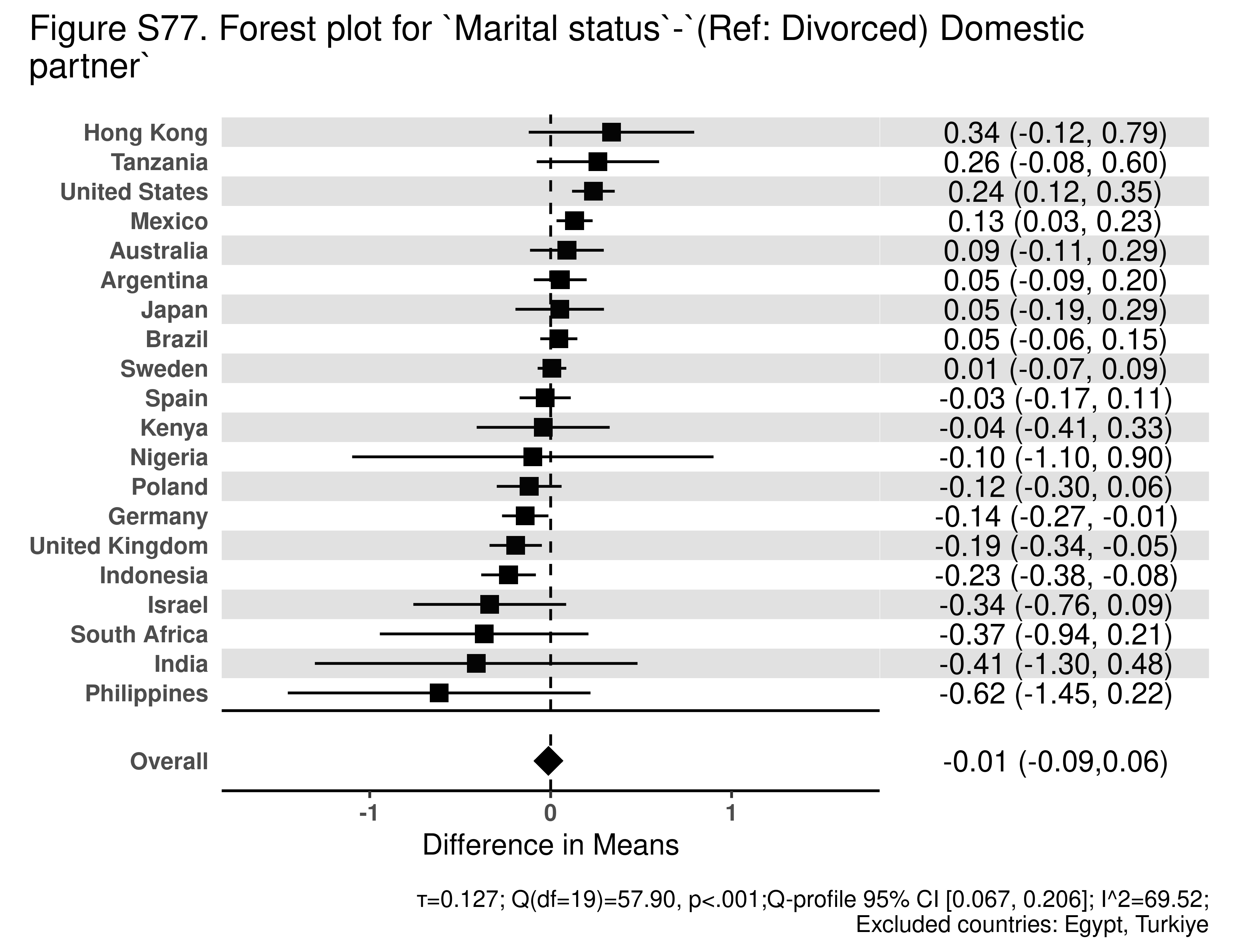

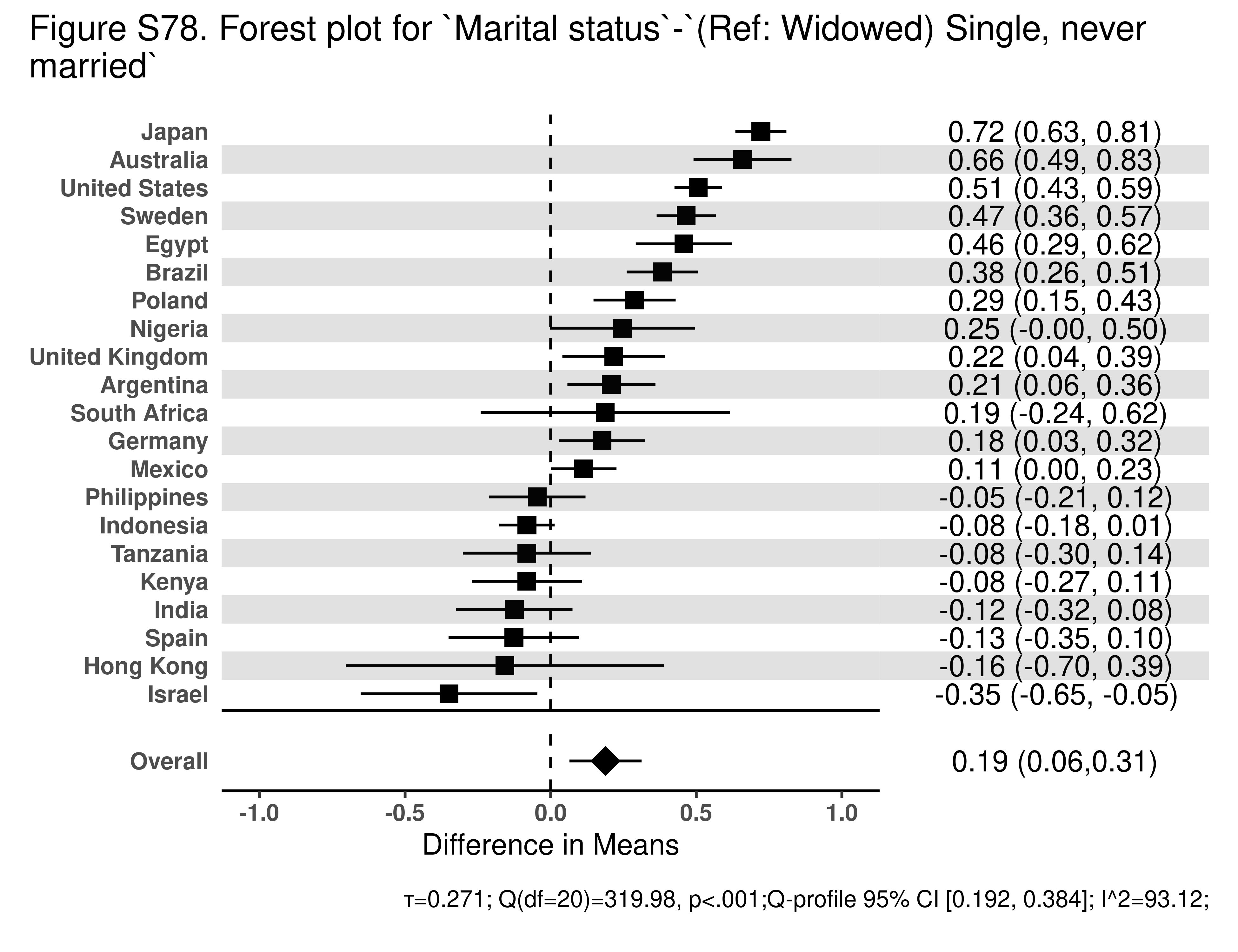

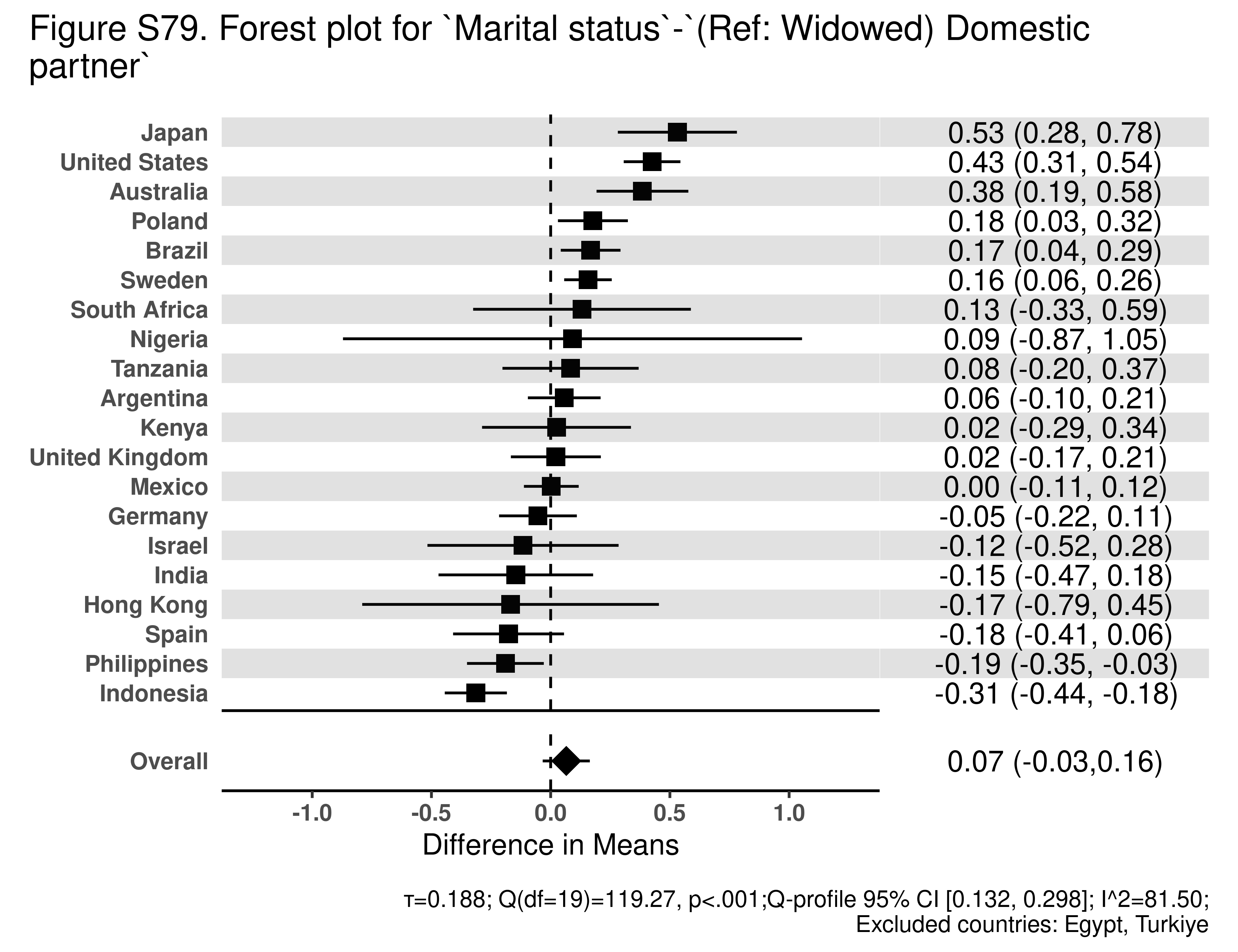

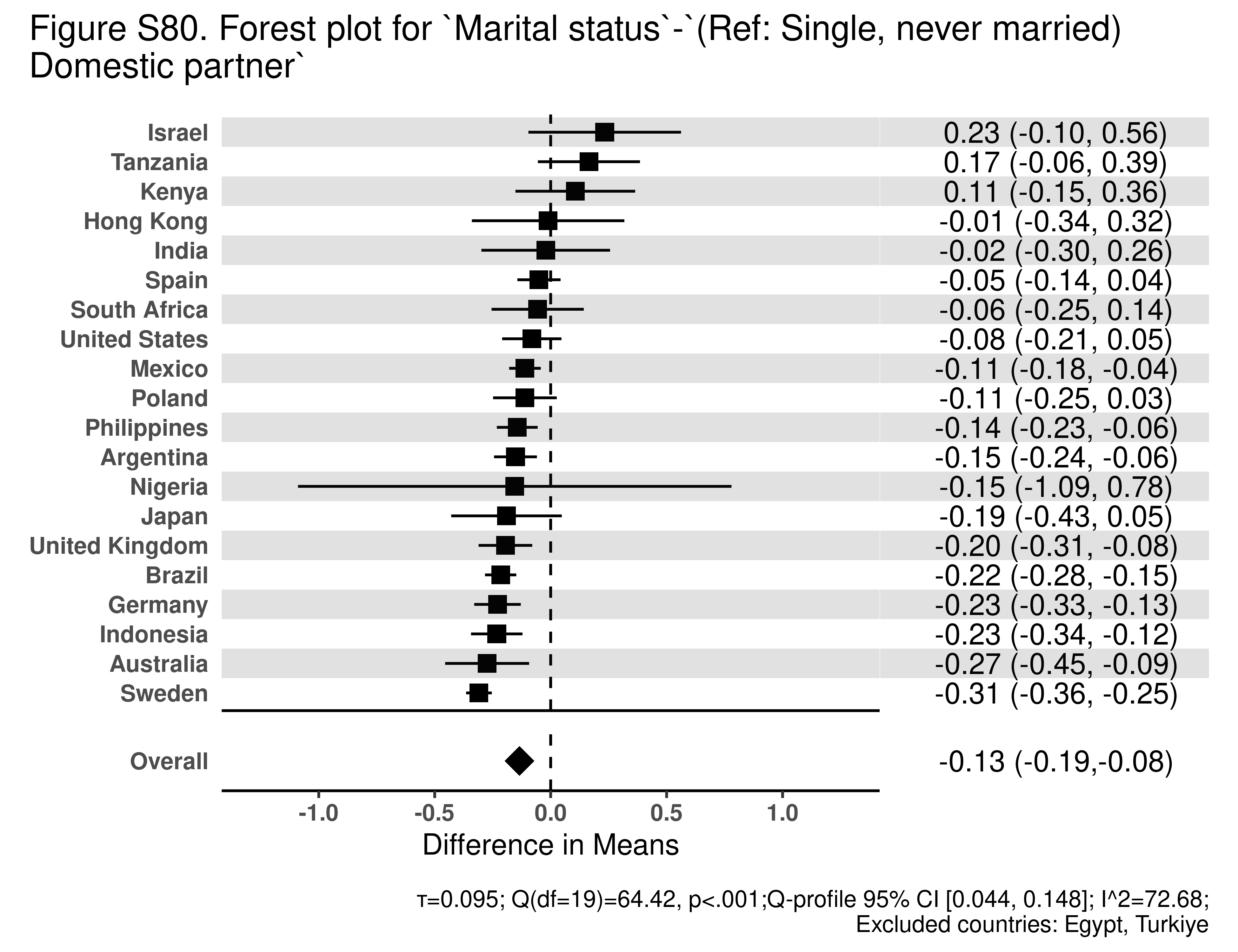

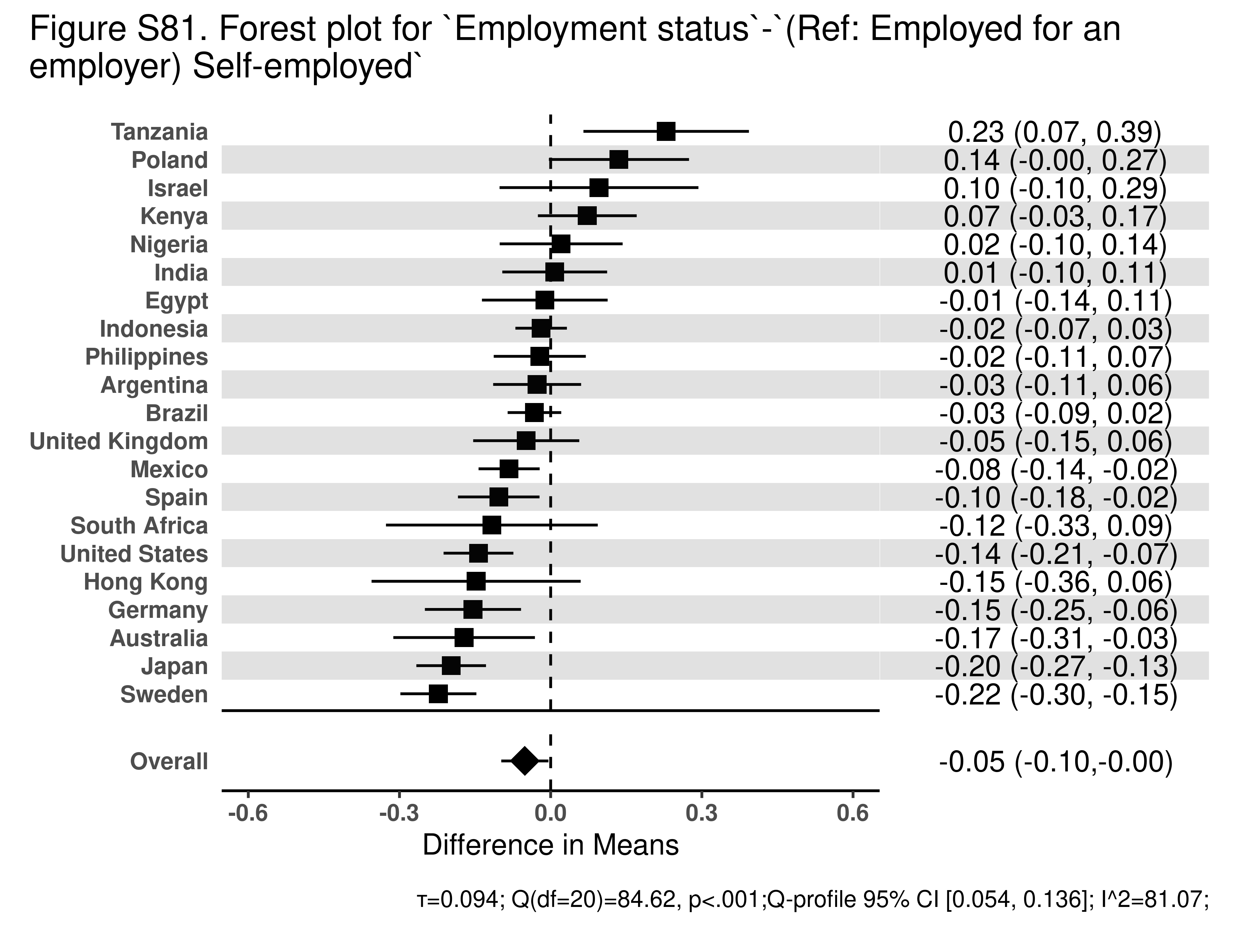

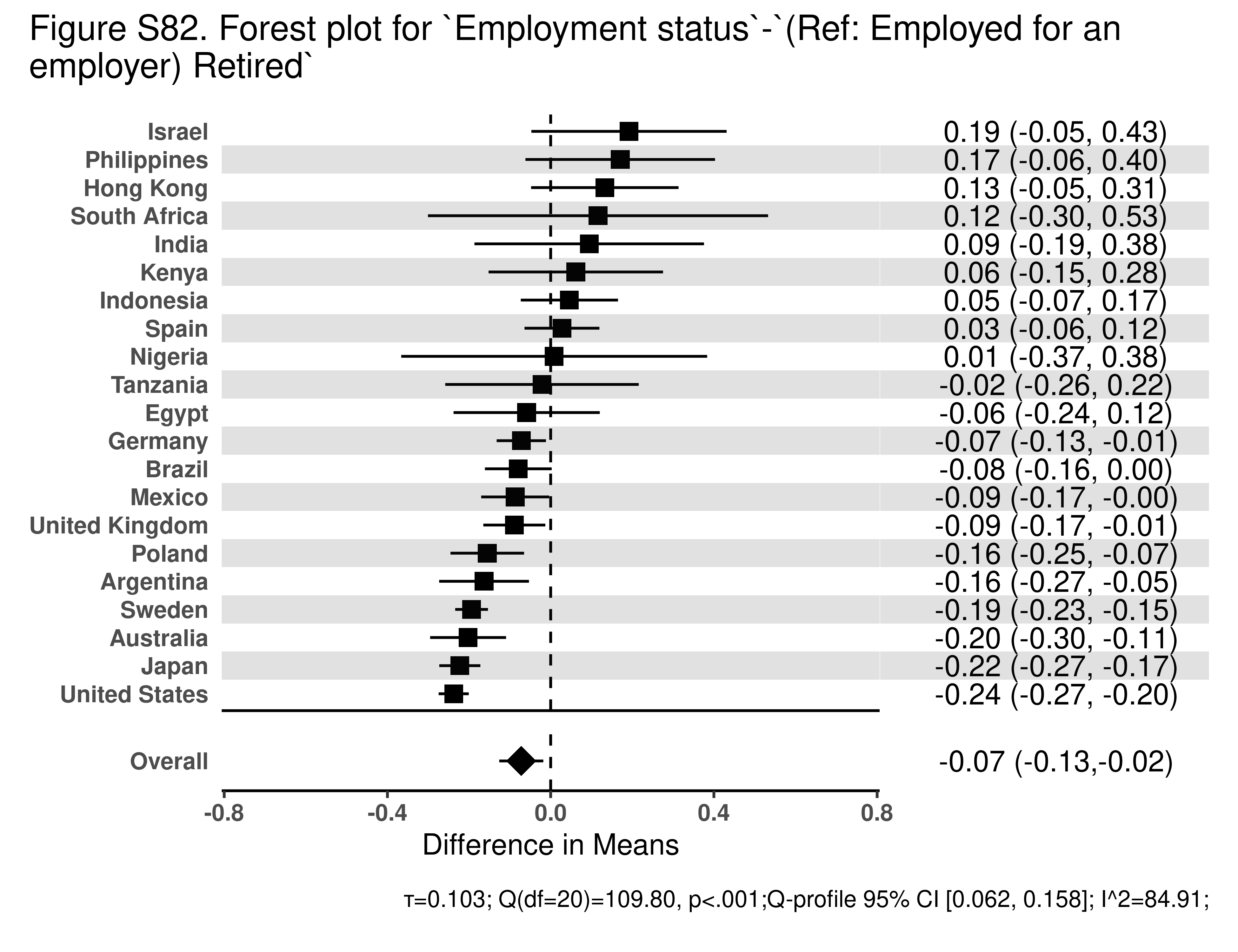

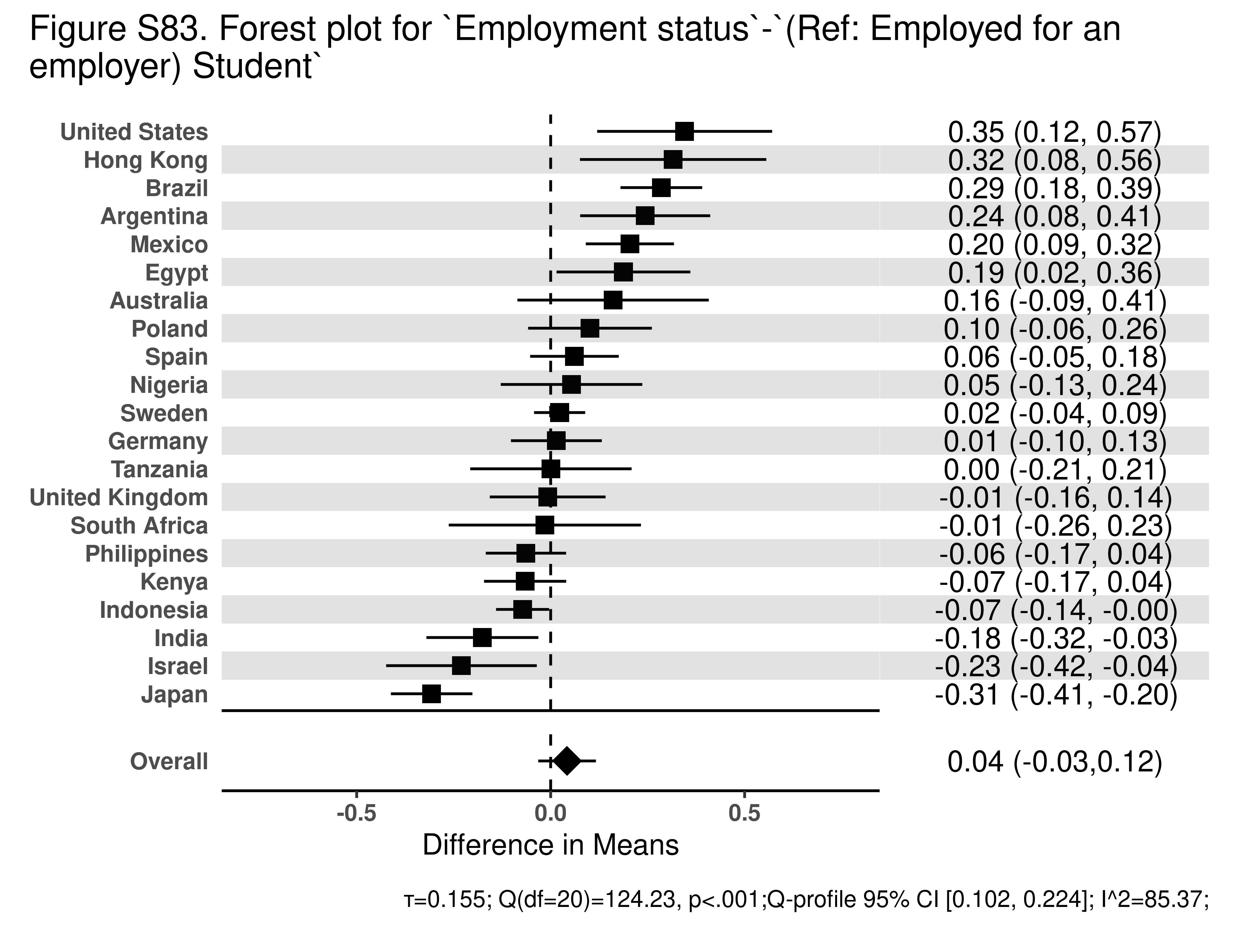

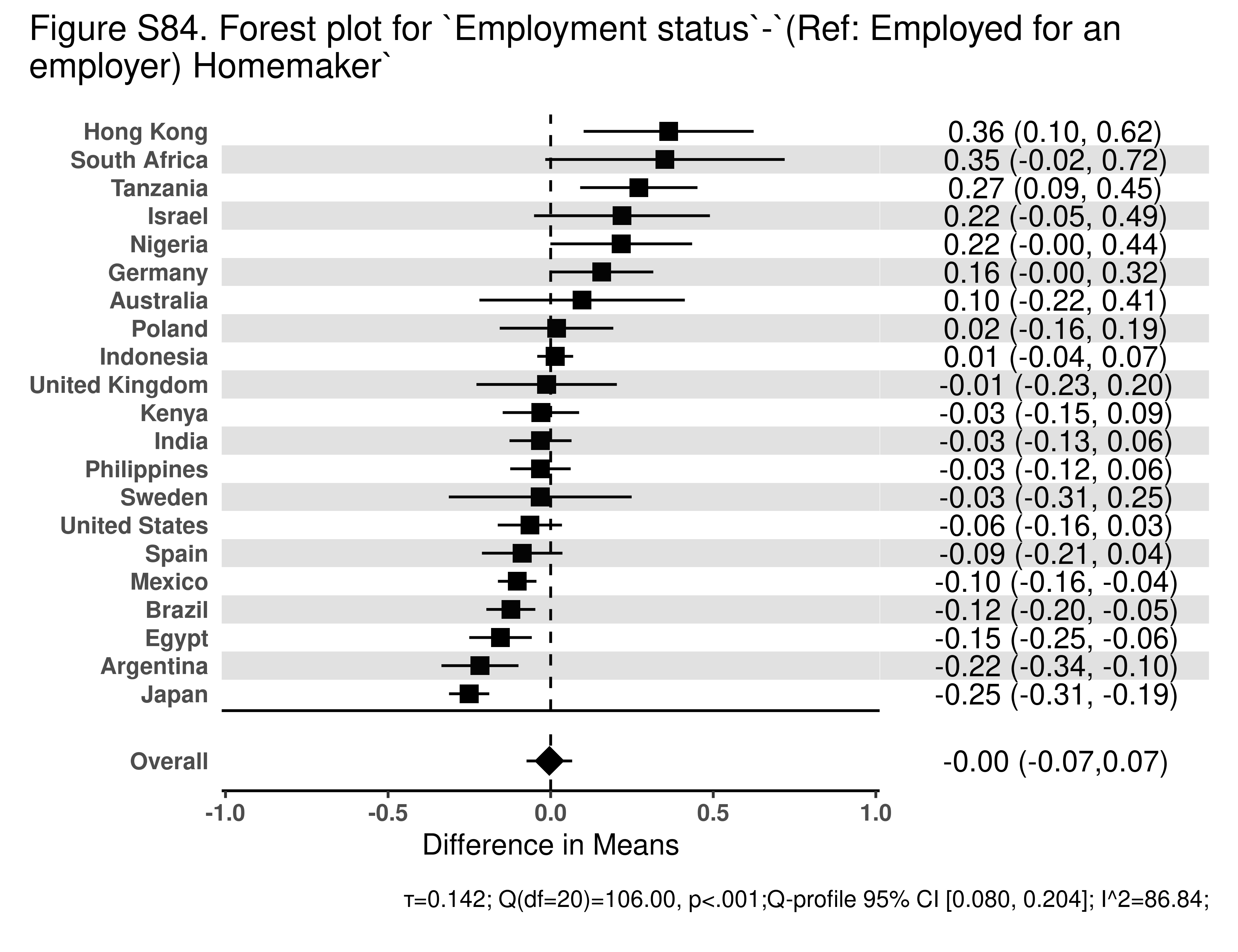

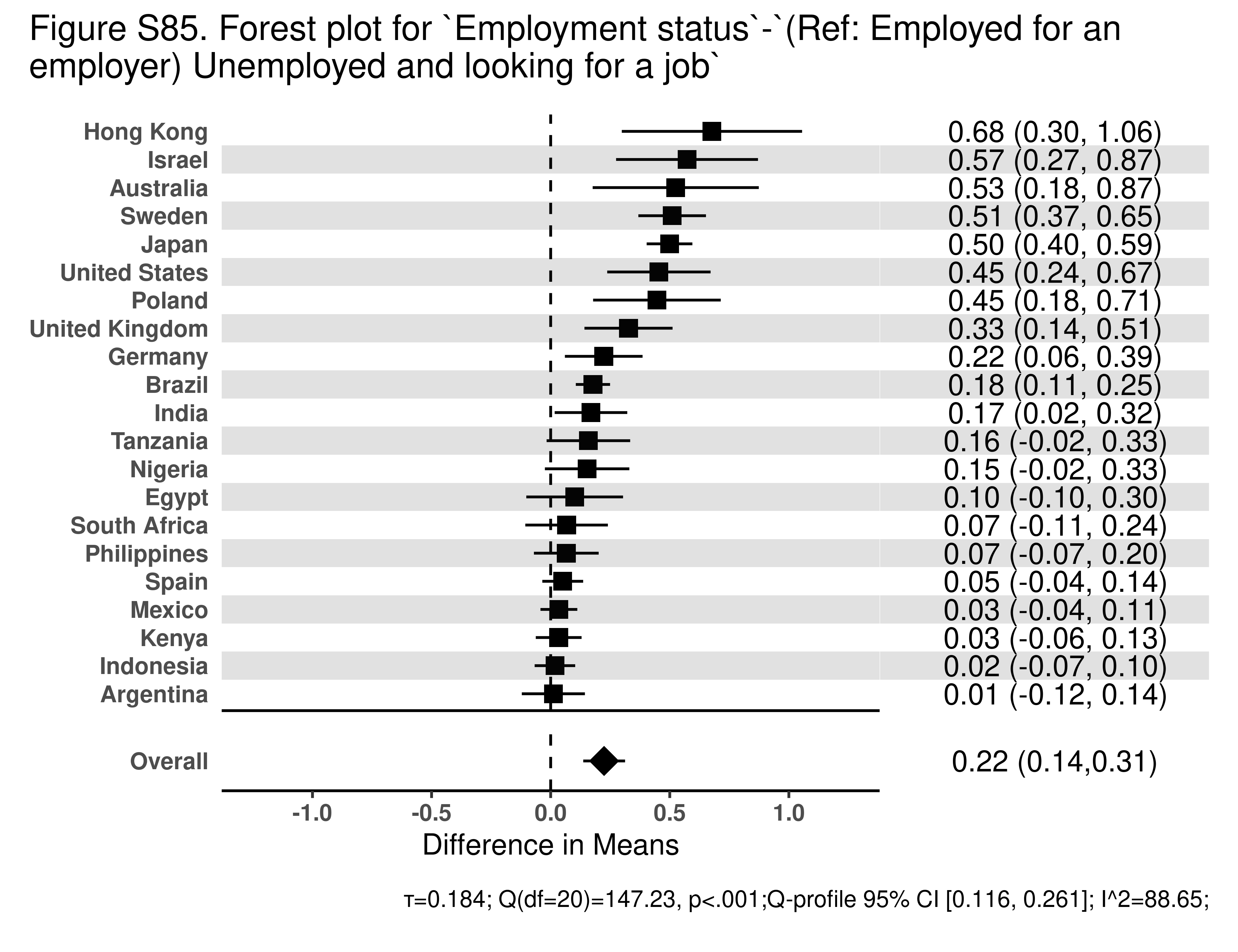

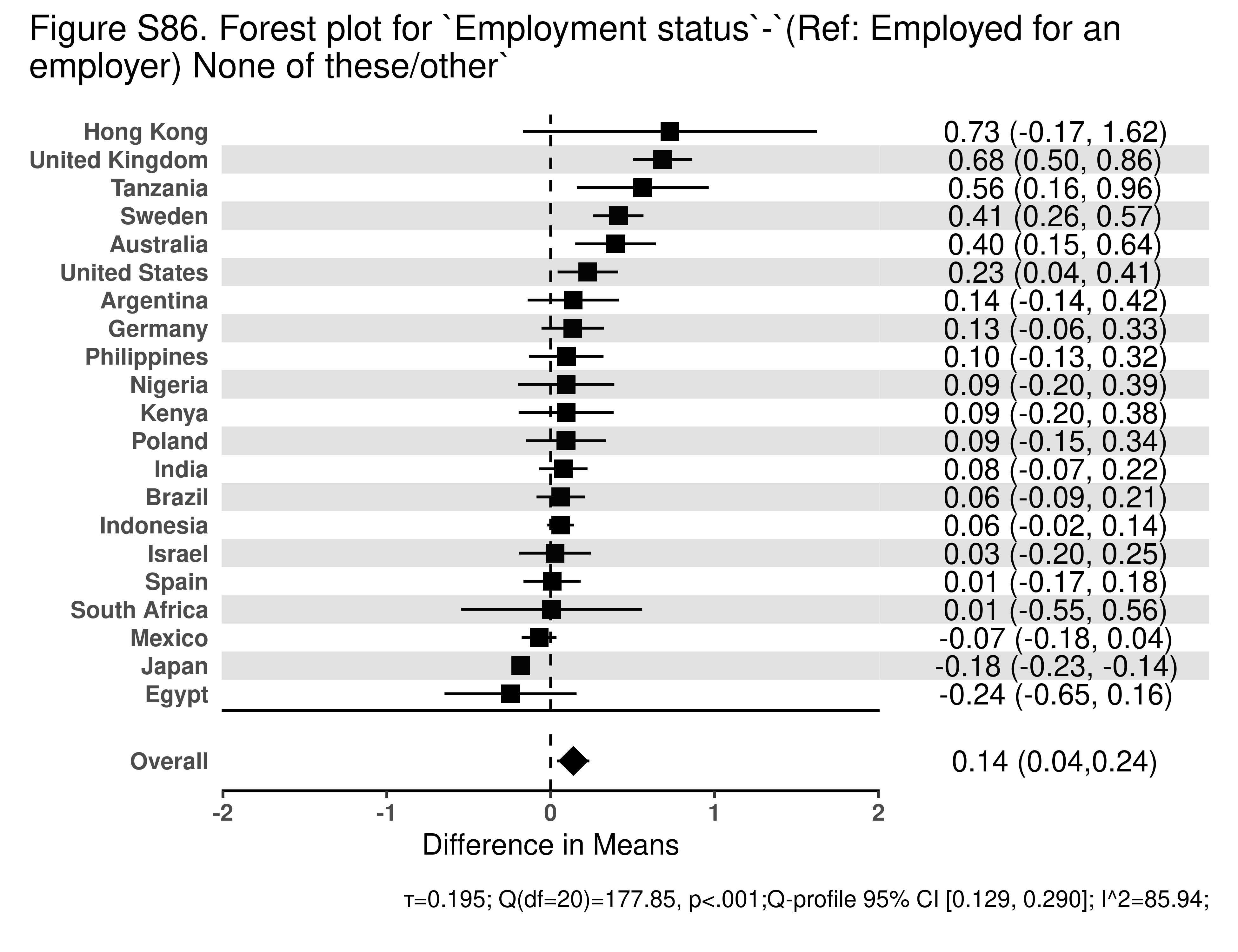

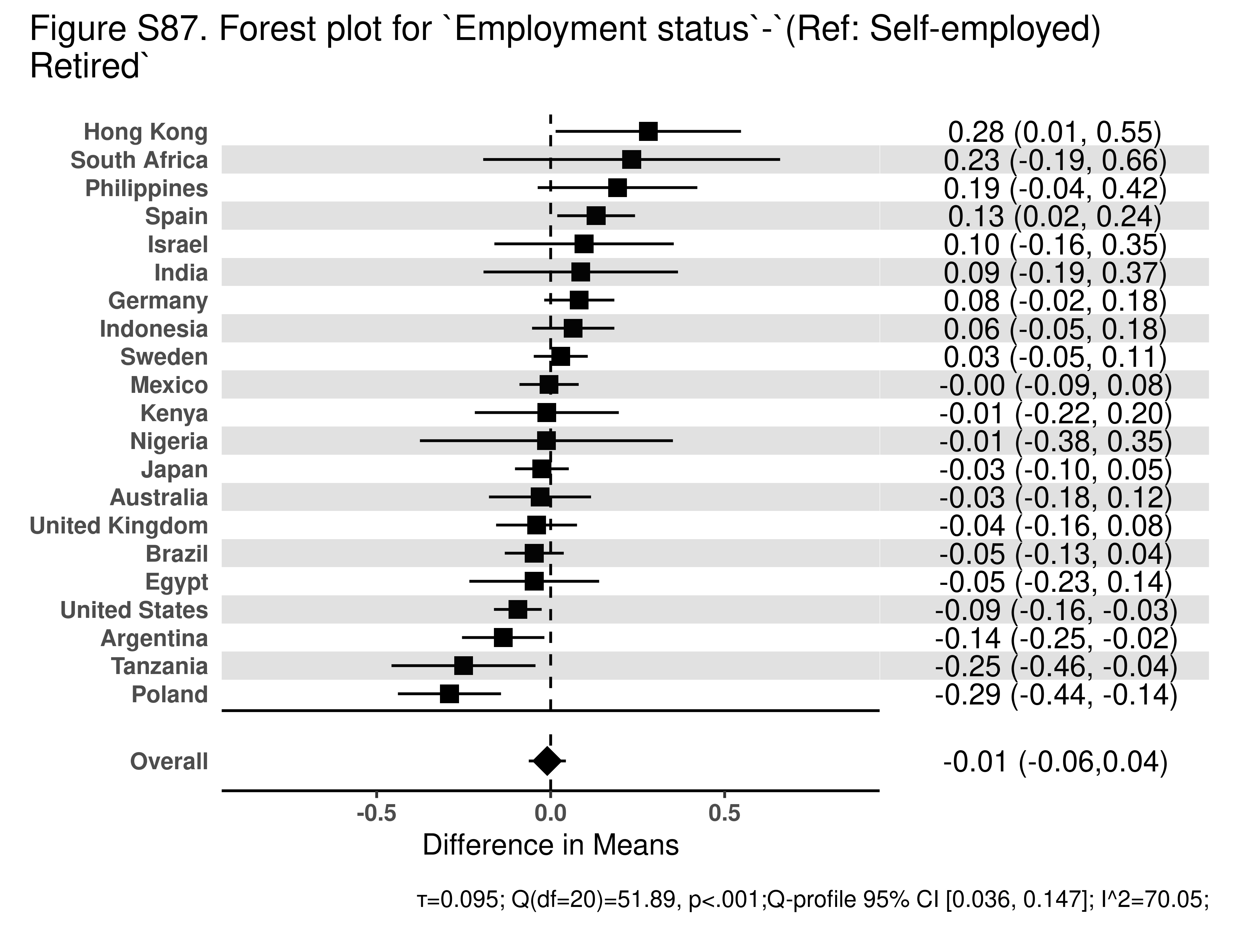

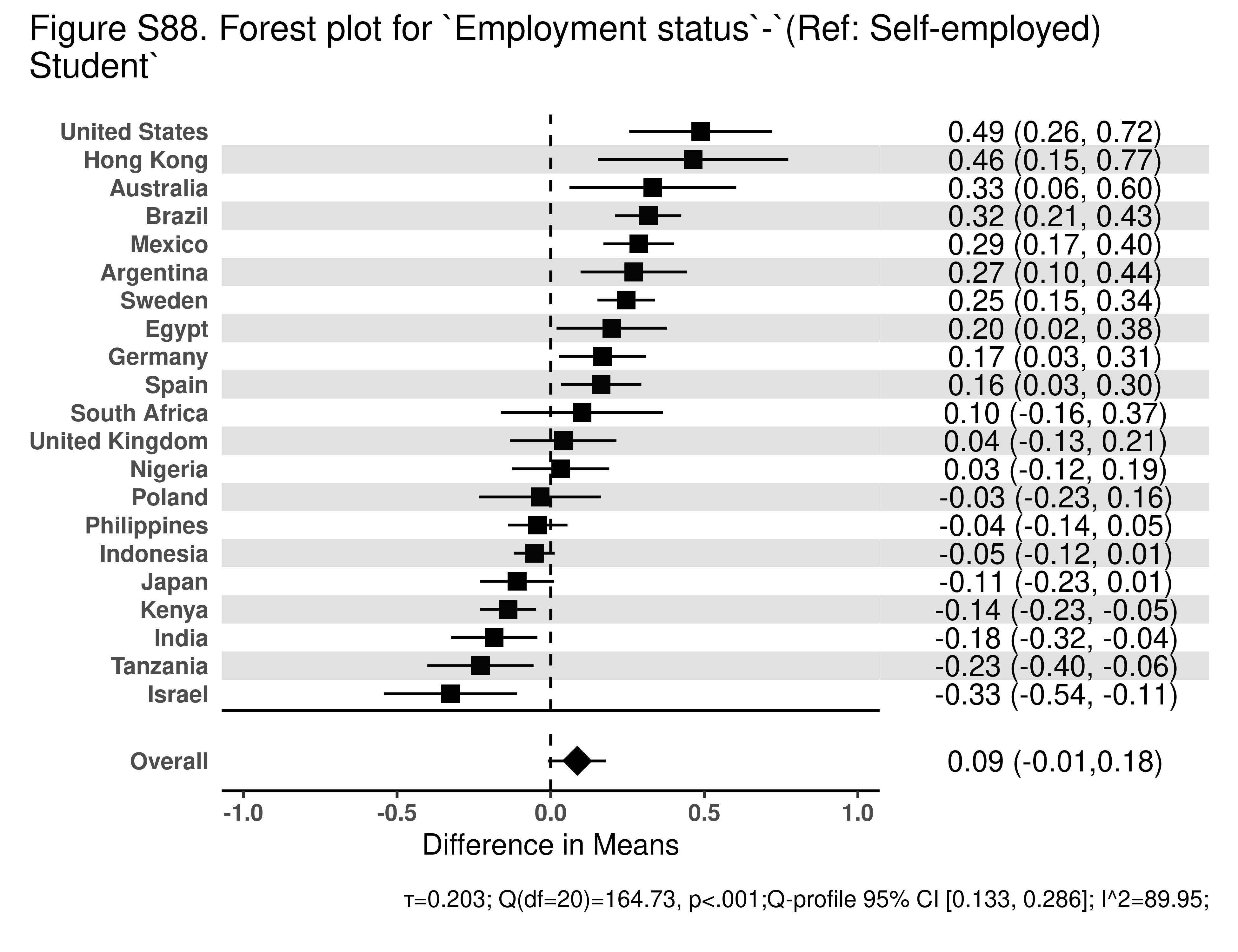

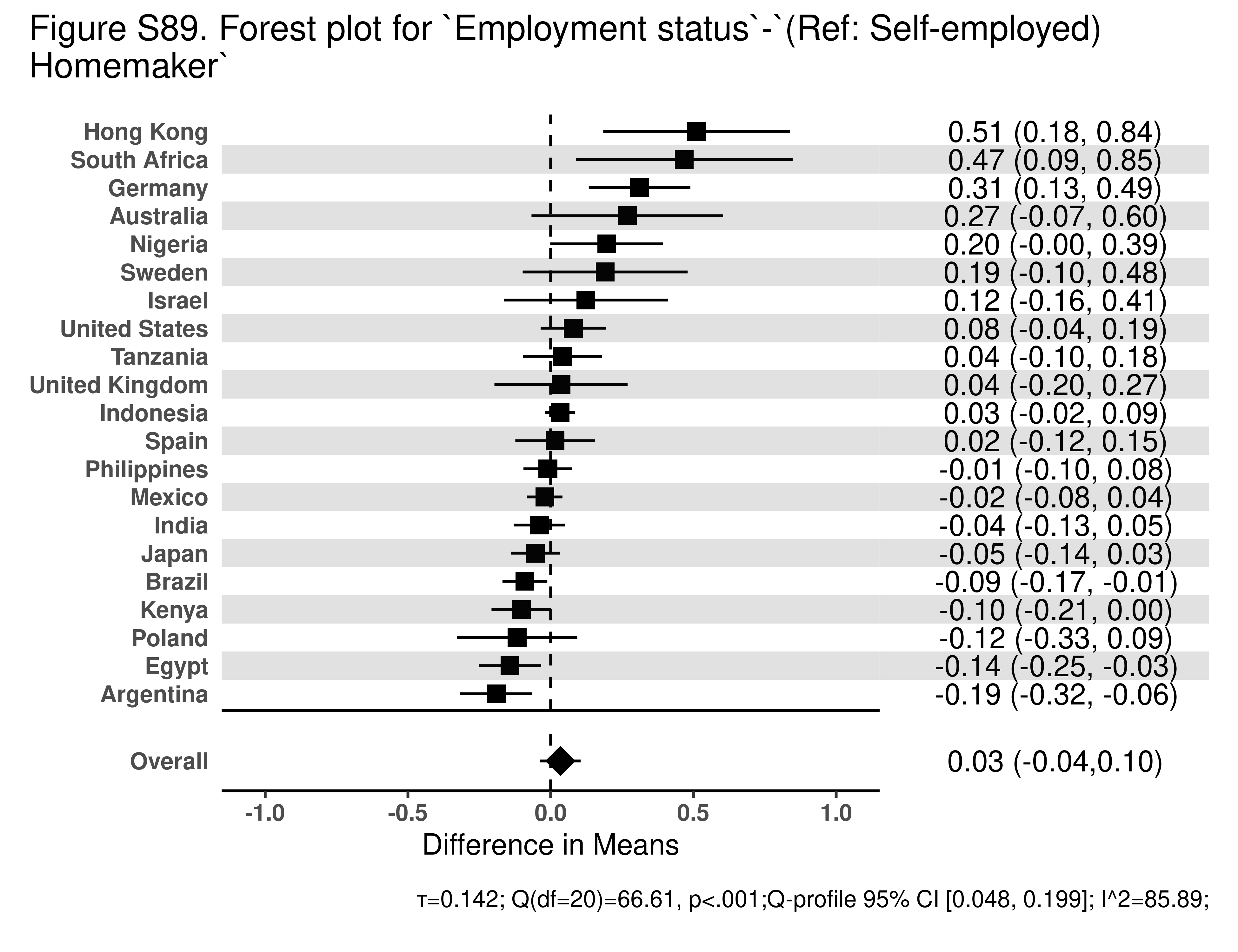

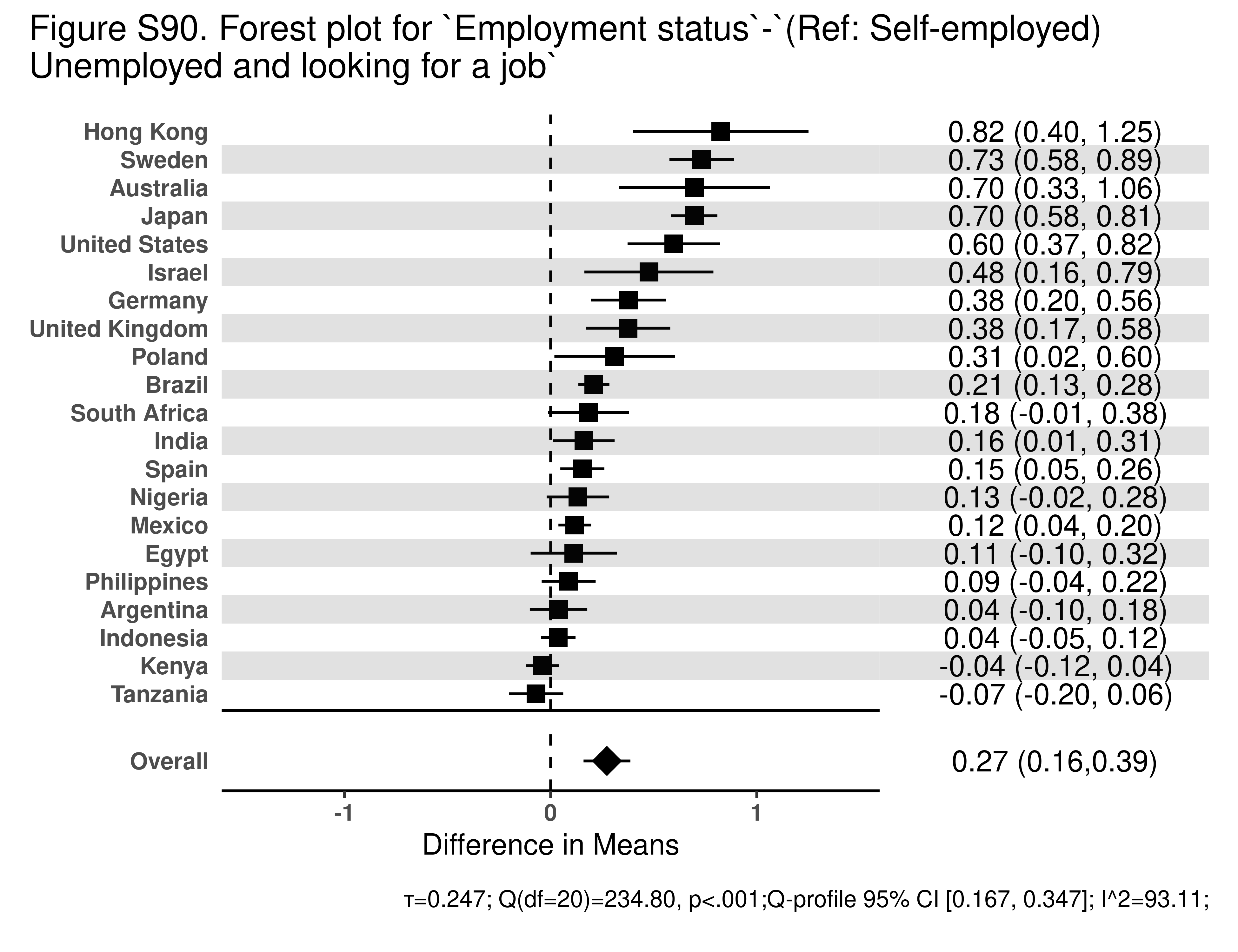

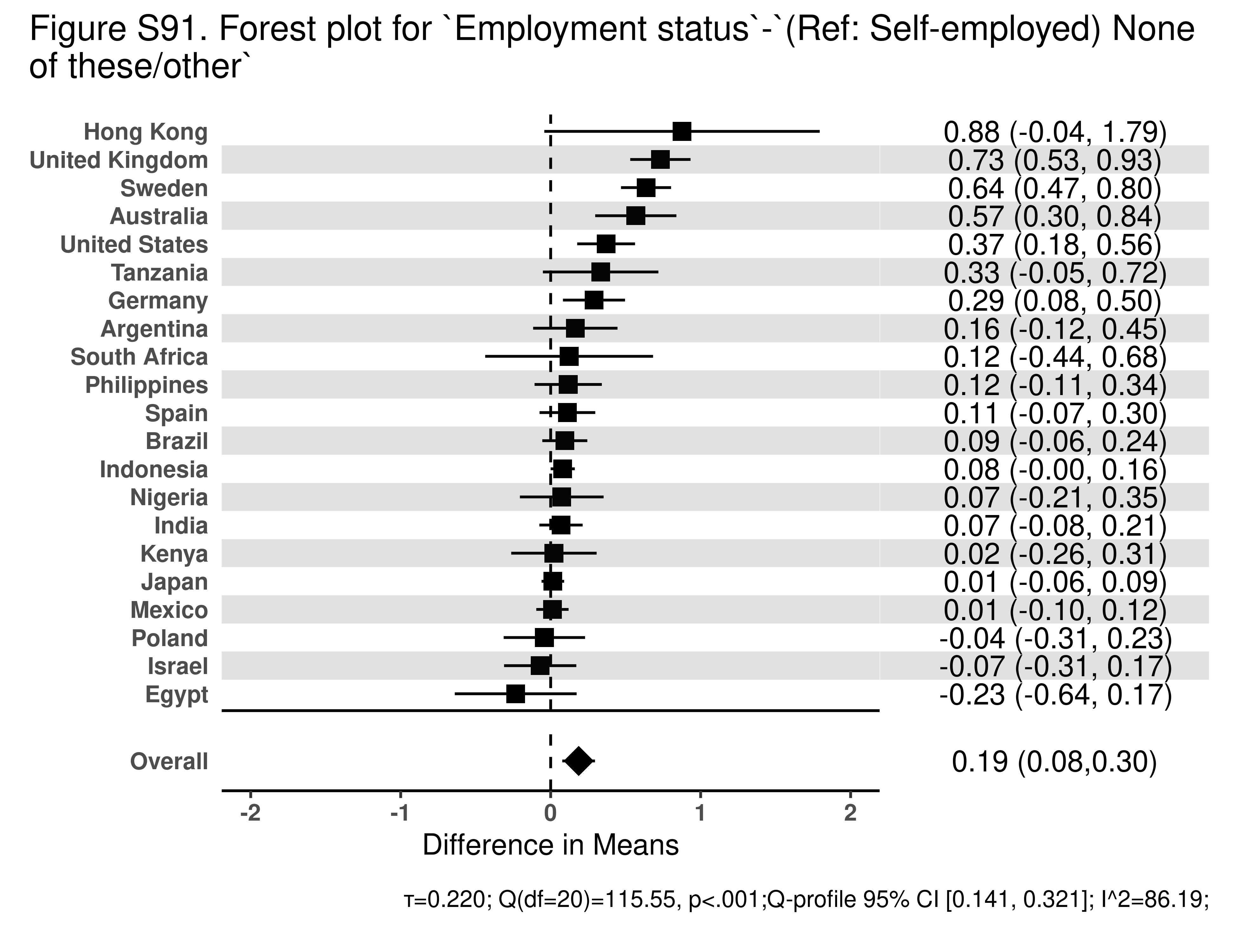

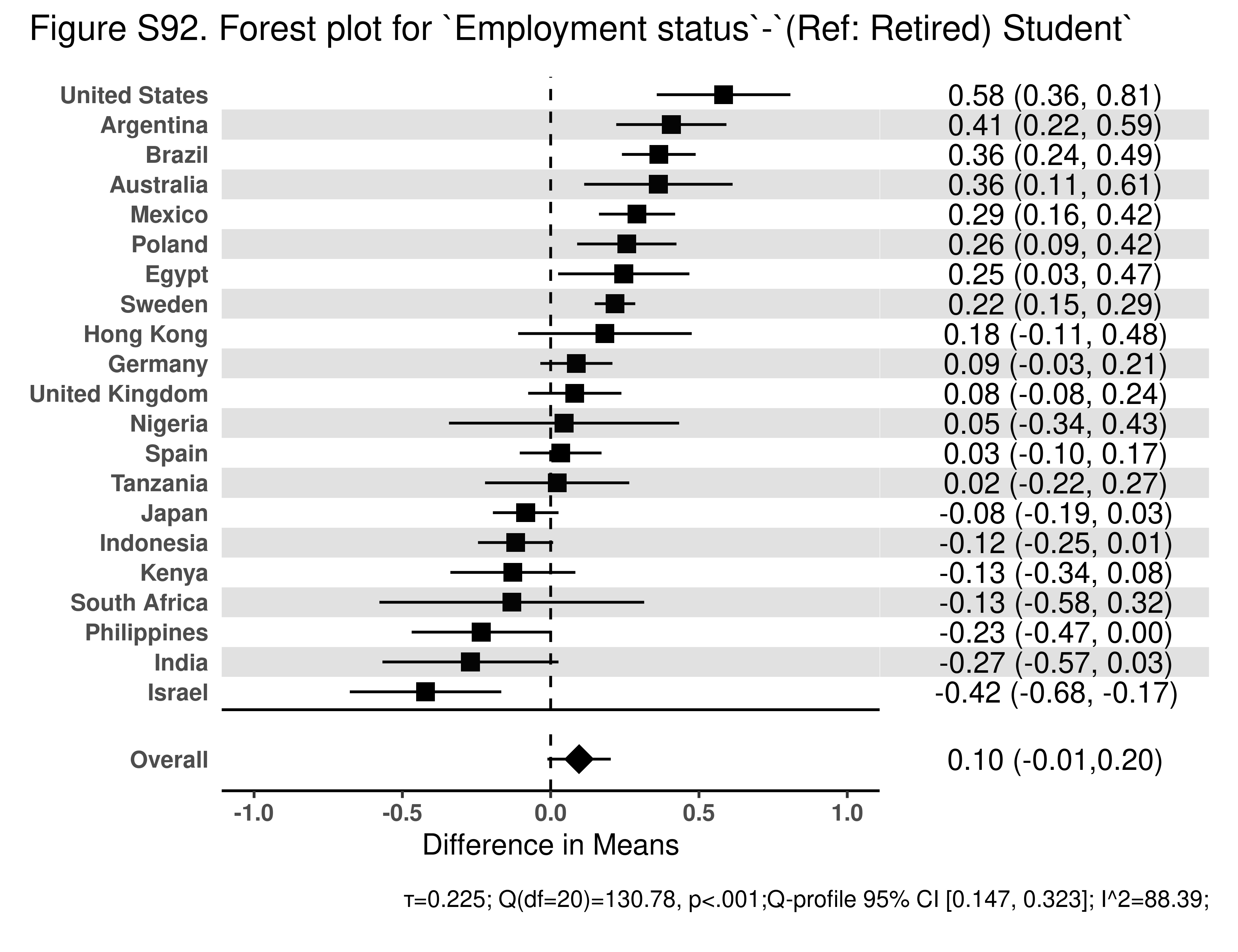

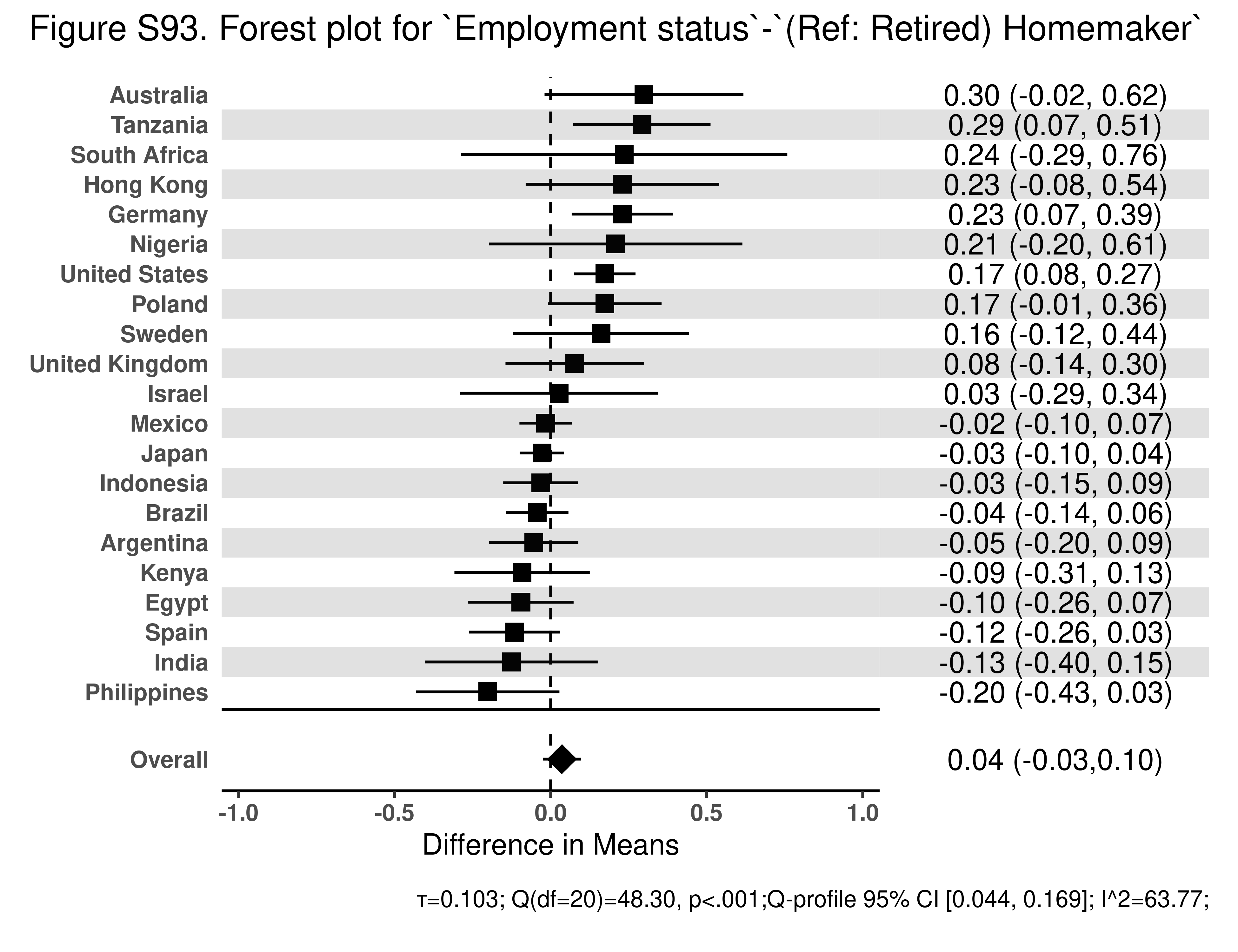

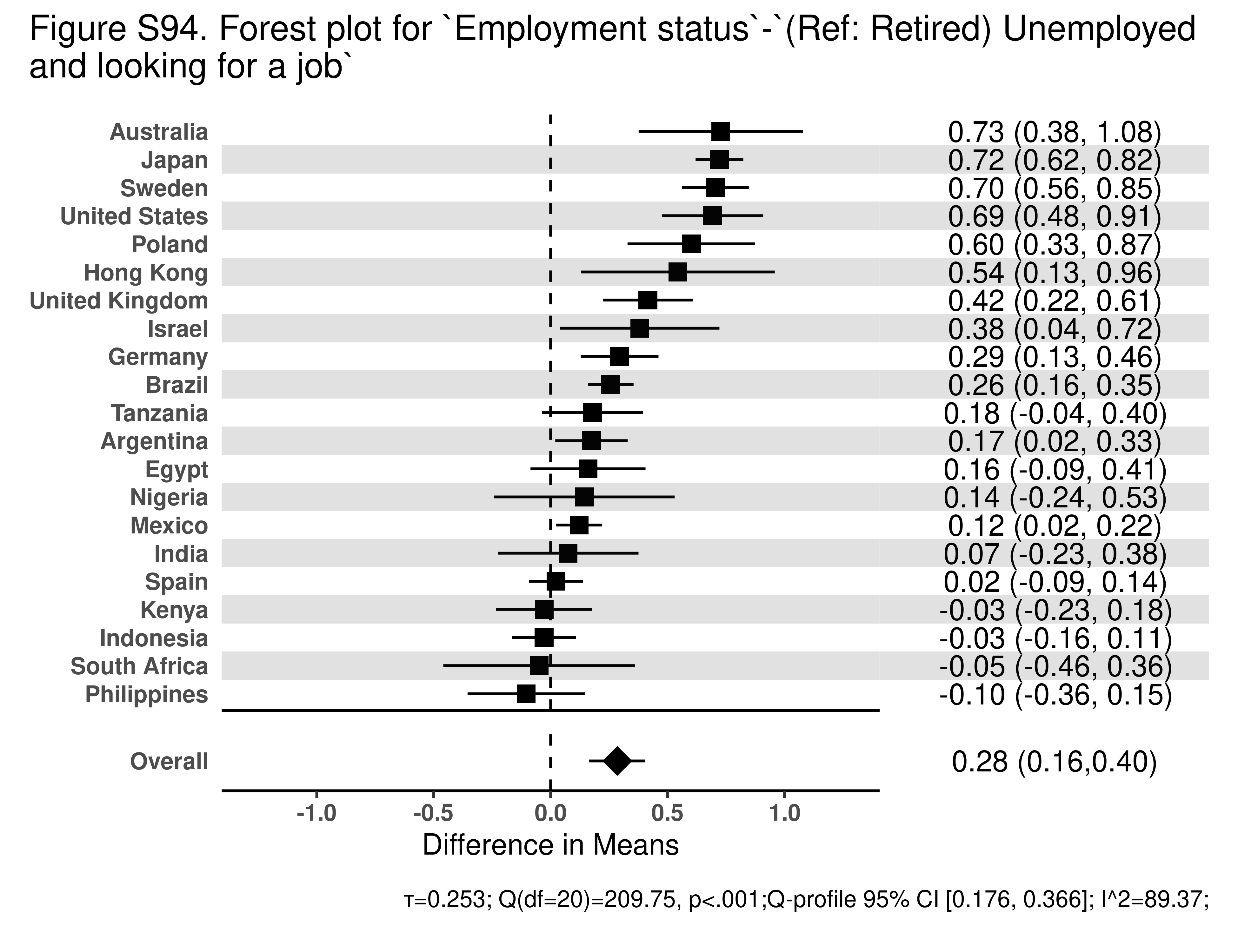

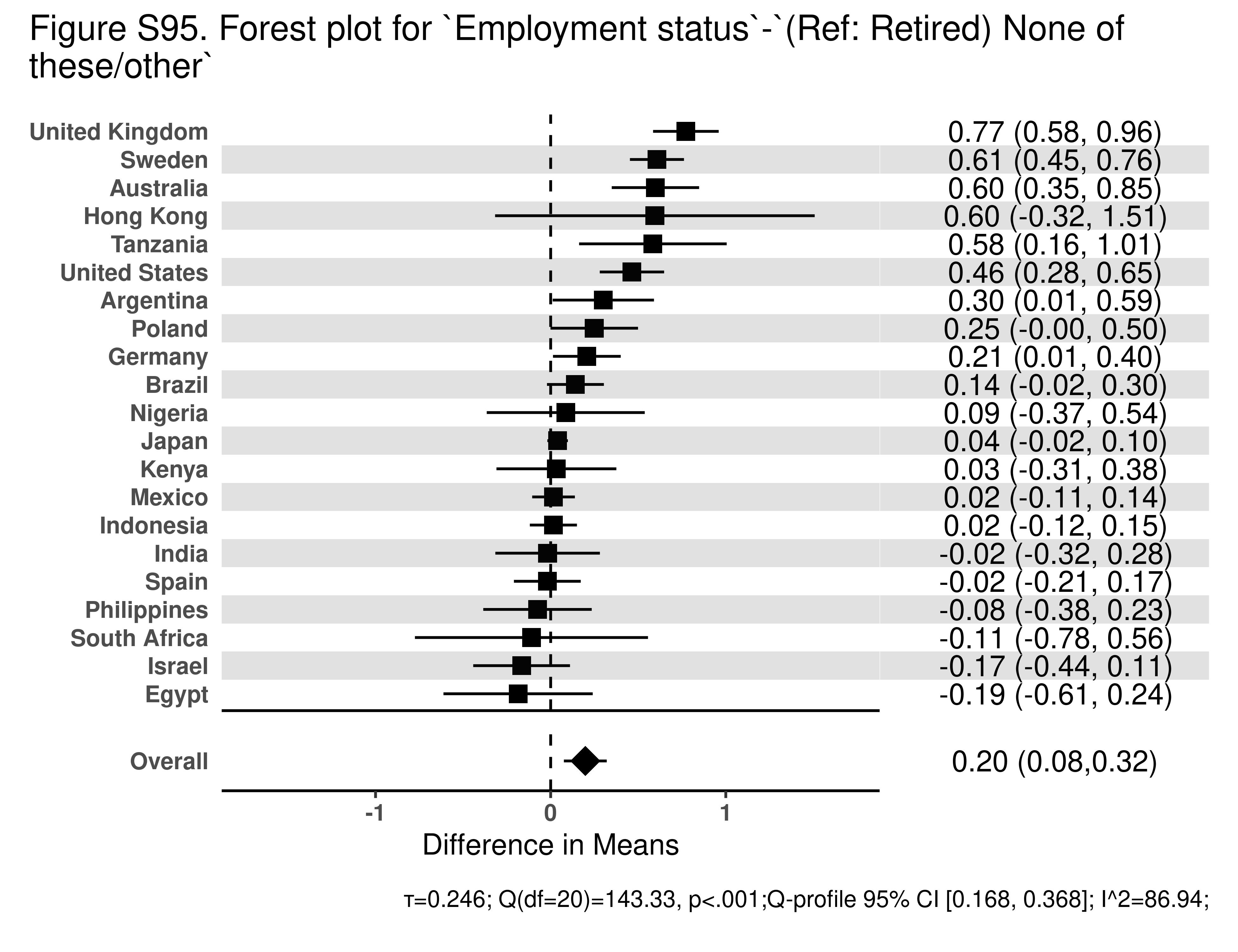

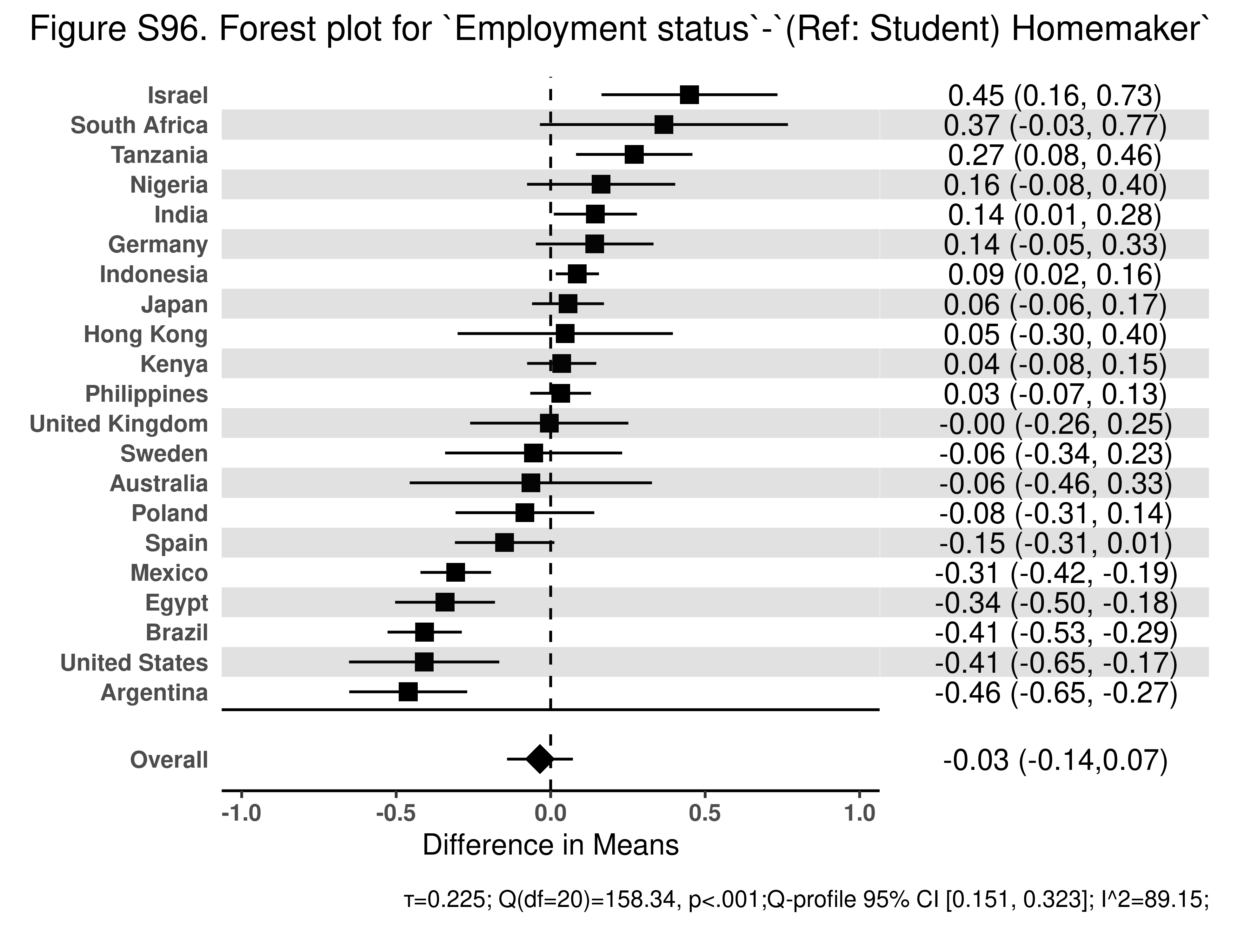

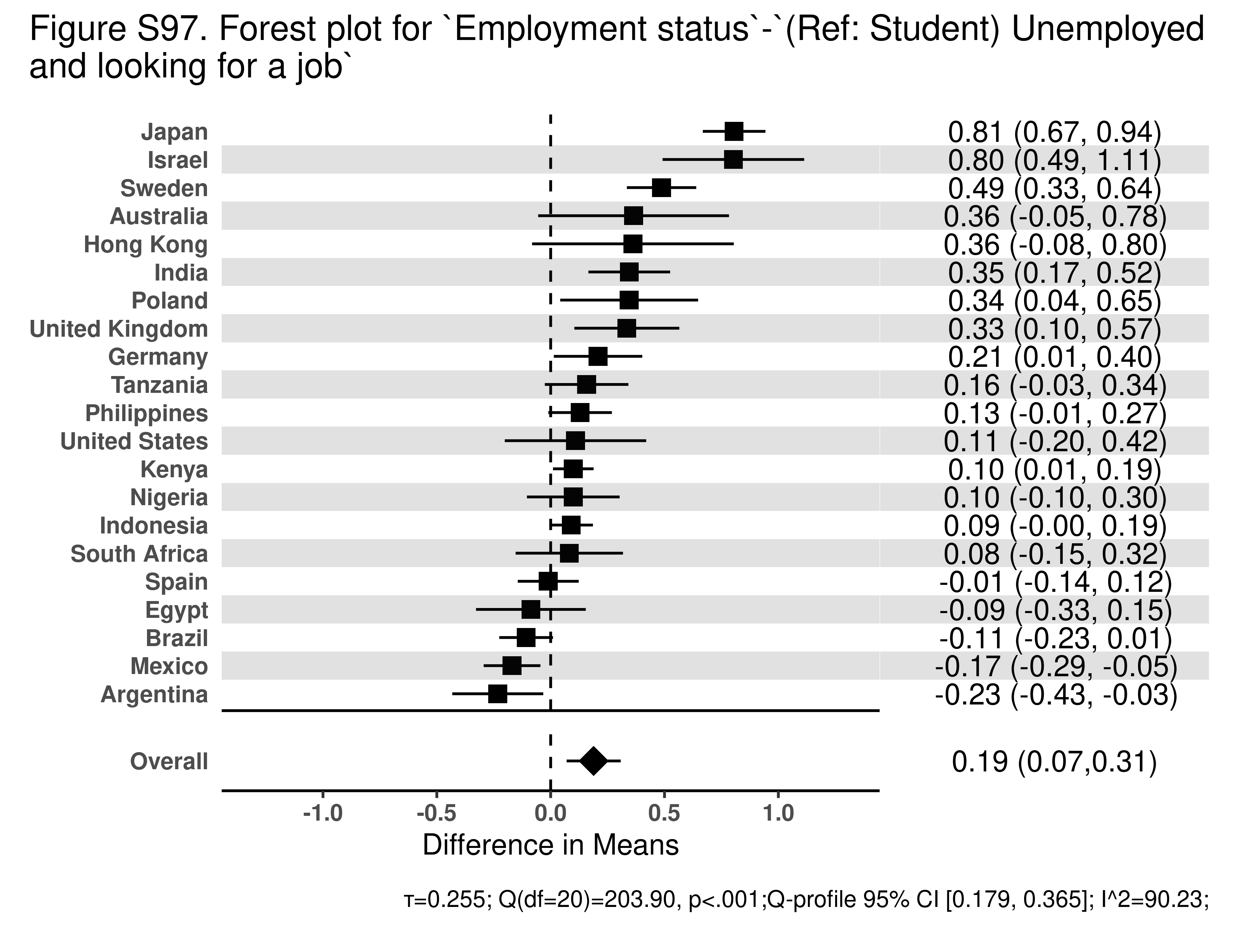

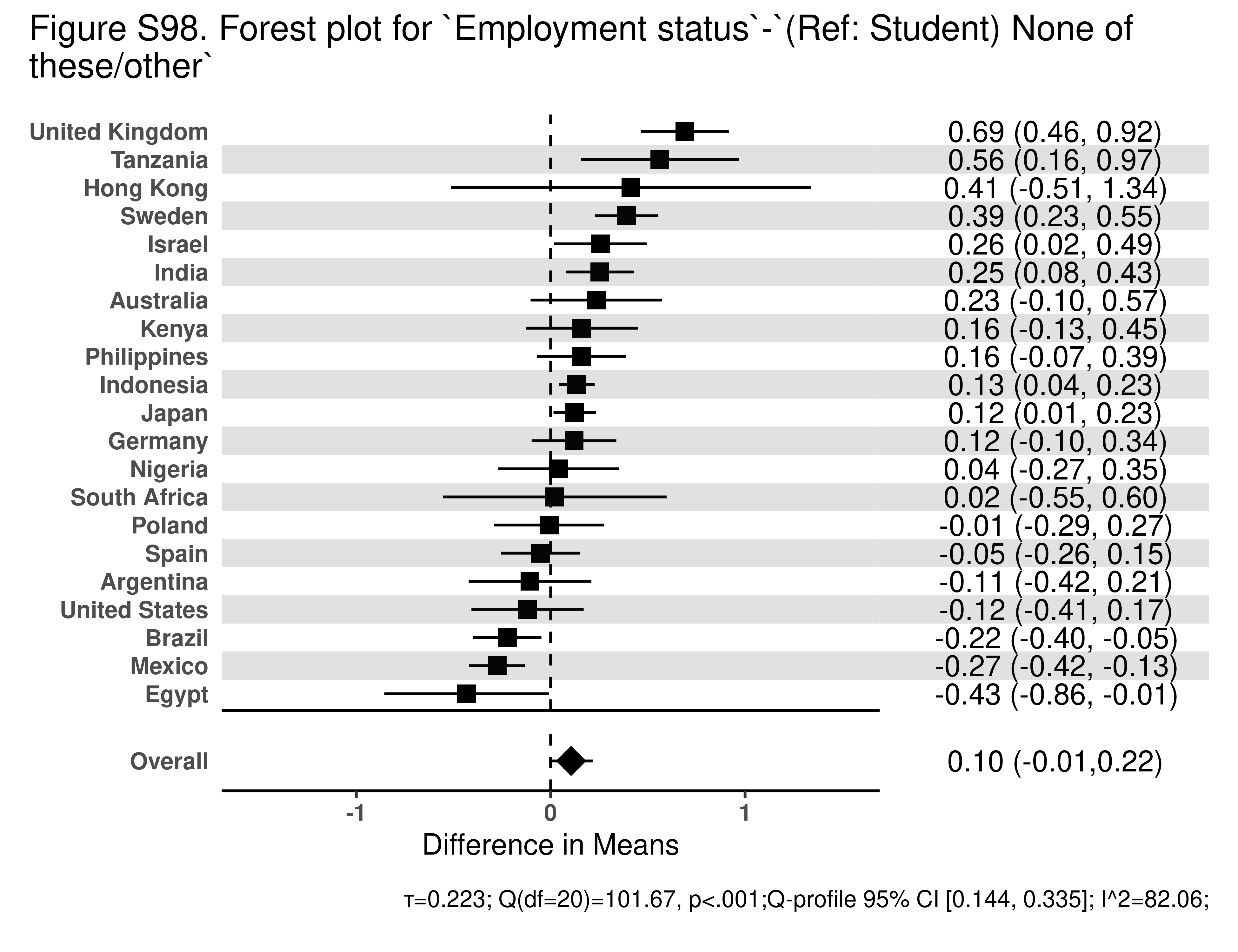

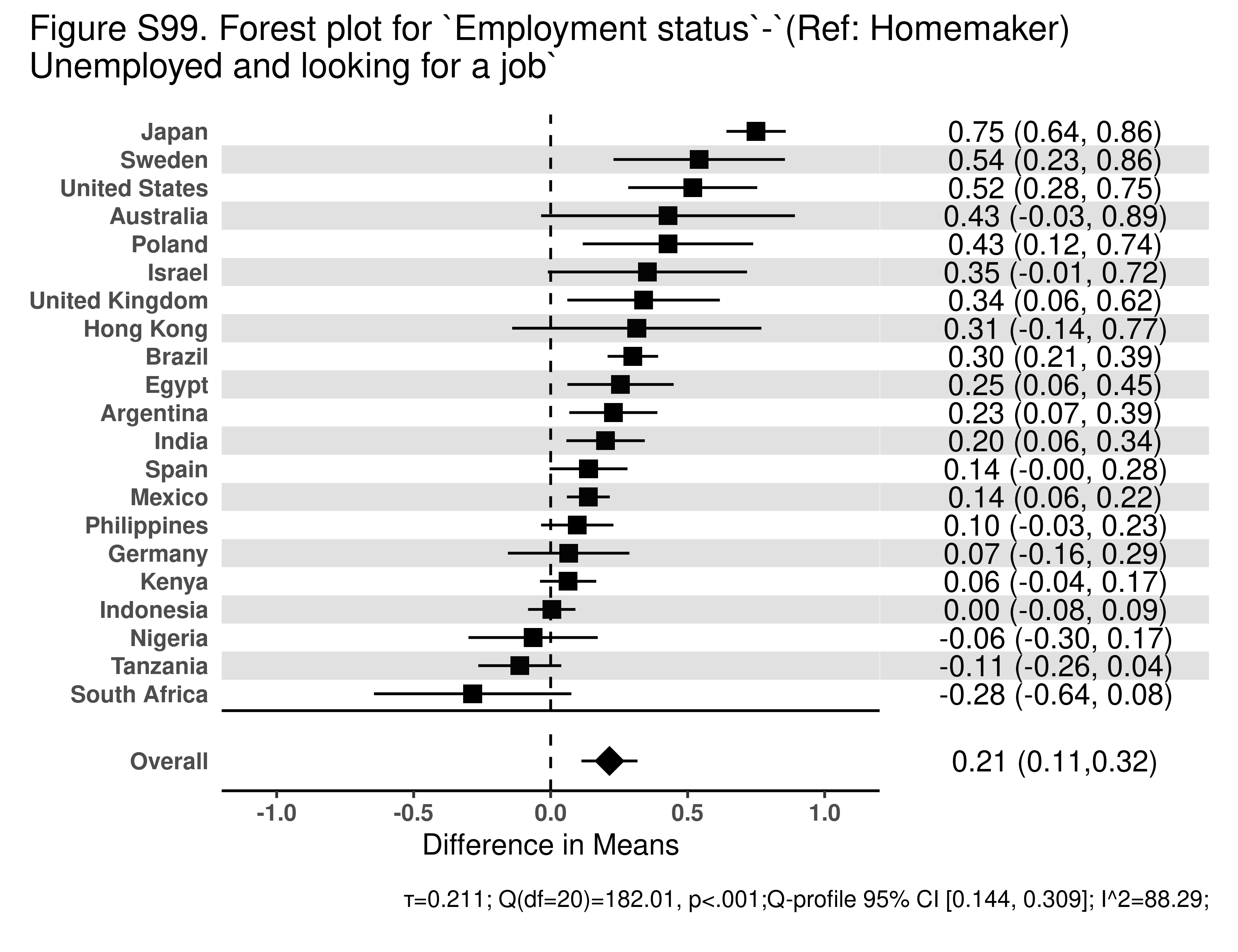

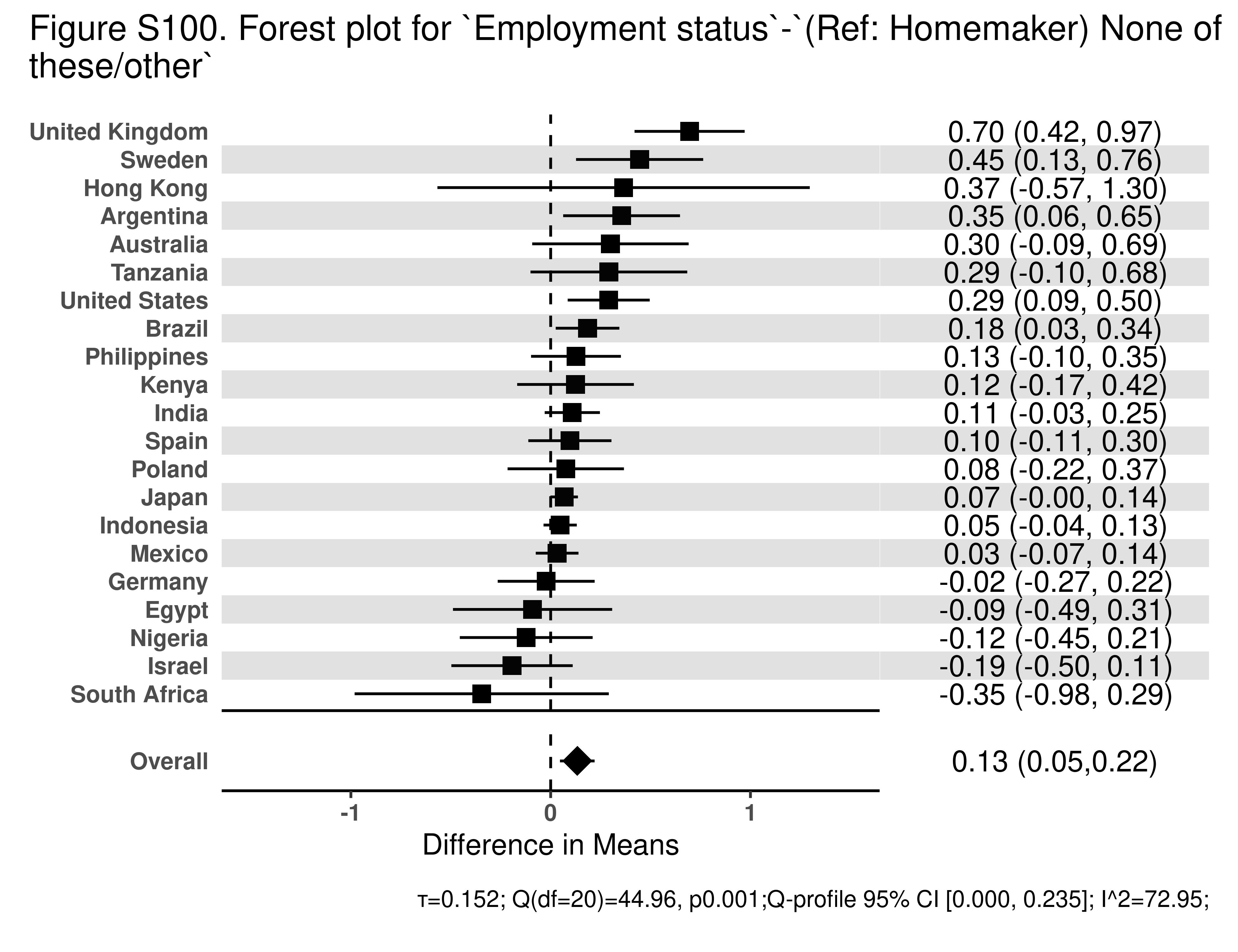

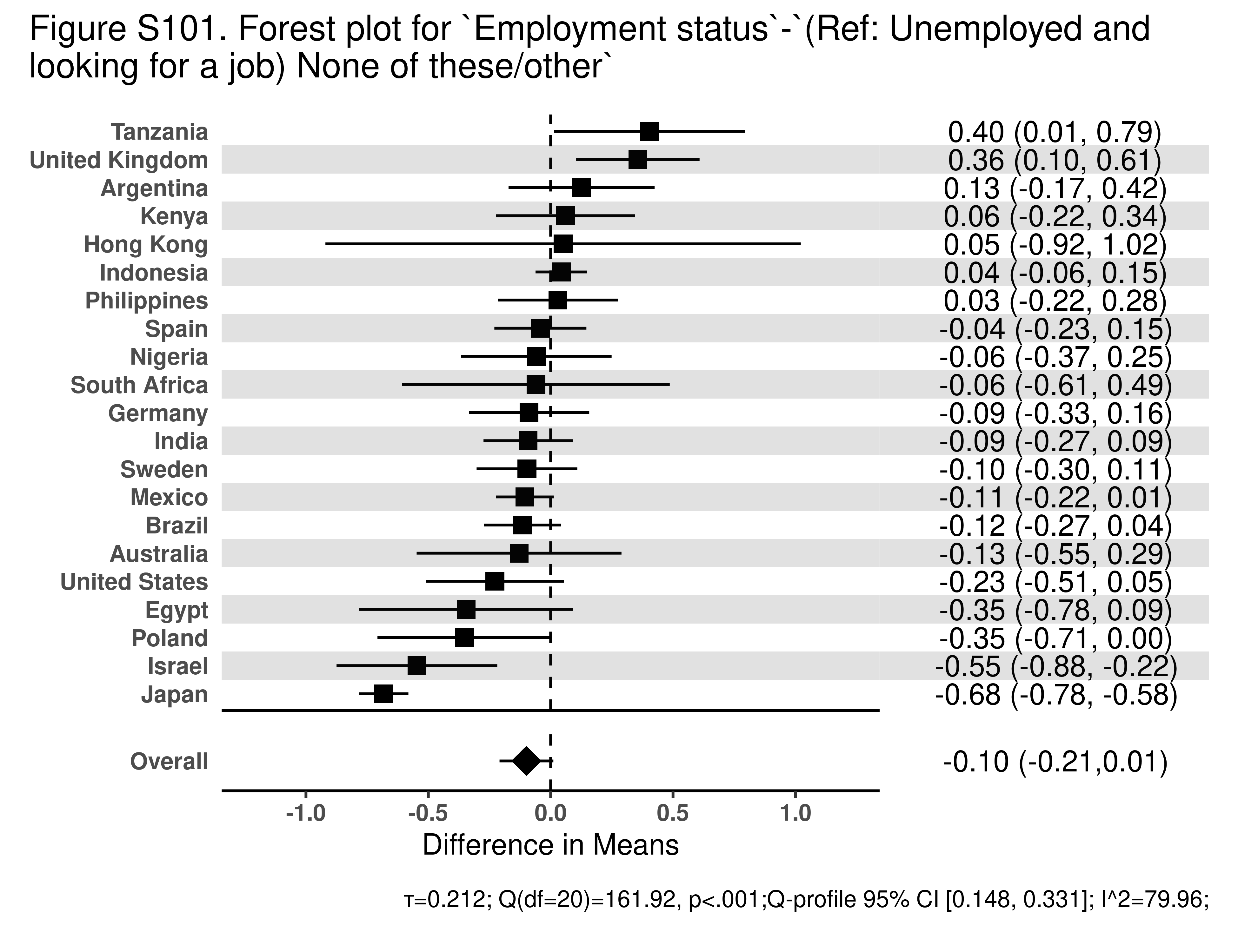

Supplement: Supplementary file 1 — Supplementary Material 1 [file 41042_2025_254_MOESM1_ESM.docx]
